# Putative antibiotic resistance genes present in extant *Bacillus licheniformis* and *Bacillus paralicheniformis* strains are probably intrinsic and part of the ancient resistome

**DOI:** 10.1371/journal.pone.0210363

**Published:** 2019-01-15

**Authors:** Yvonne Agersø, Karin Bjerre, Elke Brockmann, Eric Johansen, Bea Nielsen, Roland Siezen, Birgitte Stuer-Lauridsen, Michiel Wels, Ahmad A. Zeidan

**Affiliations:** 1 Chr. Hansen A/S, Hørsholm, Denmark; 2 Microbial Bioinformatics, Ede, The Netherlands; 3 NIZO, Ede, The Netherlands; Cornell University, UNITED STATES

## Abstract

Whole-genome sequencing and phenotypic testing of 104 strains of *Bacillus licheniformis* and *Bacillus paralicheniformis* from a variety of sources and time periods was used to characterize the genetic background and evolution of (putative) antimicrobial resistance mechanisms. Core proteins were identified in draft genomes and a phylogenetic analysis based on single amino acid polymorphisms allowed the species to be separated into two phylogenetically distinct clades with one outlier. Putative antimicrobial resistance genes were identified and mapped. A chromosomal *ermD* gene was found at the same location in all *B*. *paralichenformis* and in 27% of *B*. *licheniformis* genomes. Erythromycin resistance correlated very well with the presence of *ermD*. The putative streptomycin resistance genes, *aph* and *aadK*, were found in the chromosome of all strains as adjacent loci. Variations in amino acid sequence did not correlate with streptomycin susceptibility although the species were less susceptible than other *Bacillus* species. A putative chloramphenicol resistance gene (*cat*), encoding a novel chloramphenicol acetyltransferase protein was also found in the chromosome of all strains. Strains encoding a truncated CAT protein were sensitive to chloramphenicol. For all four resistance genes, the diversity and genetic context followed the overall phylogenetic relationship. No potentially mobile genetic elements were detected in their vicinity. Moreover, the genes were only distantly related to previously-described *cat*, *aph*, *aad* and *erm* genes present on mobile genetic elements or in other species. Thus, these genes are suggested to be intrinsic to *B*. *licheniformis* and *B*. *paralicheniformis* and part of their ancient resistomes. Since there is no evidence supporting horizontal transmission, these genes are not expected to add to the pool of antibiotic resistance elements considered to pose a risk to human or animal health. Whole-genome based phylogenetic and sequence analysis, combined with phenotypic testing, is proposed to be suitable for determining intrinsic resistance and evolutionary relationships.

## Introduction

The development and spread of antimicrobial resistance is considered a major threat to human health [1]. The excess consumption of antimicrobials by humans and their massive use in the agricultural sector are major contributing factors [2, 3]. However, antimicrobial resistance is also part of the natural microbial ecology and exists in bacteria from all environments [2, 4]. Antimicrobial resistance can either be intrinsic to a given species, or acquired through mutation or uptake of resistance genes via horizontal gene transfer [2, 5]. Antimicrobial resistance mechanisms have evolved over time as part of bacterial evolution; some mechanisms evolved to protect the bacteria from naturally occurring antimicrobials, while other mechanisms serve different purposes within the cell with antimicrobial resistance being an additional benefit. Such resistance mechanisms are referred to as intrinsic [5]. Intrinsic resistance does not normally spread horizontally between bacteria but spreads clonally and is often seen as a common trait within a bacterial species or subpopulation which share a common evolutionary history [5]. Several recent studies have used whole genome sequencing (WGS) to characterize intrinsic resistance in Gram-negative (*Pseudomonas aeruginosa* and *Acinetobacter*) and Gram-positive (*Staphylococcus aureus*) bacteria [6–8].

Antimicrobial resistance mechanisms can be present on mobile genetic elements such as plasmids or conjugative transposons and such elements may spread horizontally between bacteria from different environments and between different bacterial species [9]. Antimicrobial resistance obtained by horizontal gene transfer (conjugation, transduction or transformation) is referred to as acquired resistance [10]. Antimicrobial resistance can also be acquired by mutation, for example by changing the target site of the specific antimicrobial. Resistance acquired by mutation is not considered to be horizontally transmissible.

Antimicrobial resistance in pathogenic bacteria, whether intrinsic or acquired, can contribute to treatment failure due to ineffective antimicrobial therapy. Intrinsic resistance mechanisms, when present in non-pathogenic bacteria, do not add to the resistance pool in pathogenic bacteria as intrinsic resistance is not readily spread horizontally between bacteria. Resistance mechanisms linked to mobile genetic elements in non-pathogenic bacteria are, however, considered a risk [11, 12]. It is therefore necessary to ensure that viable microorganisms intentionally added to food and feed do not add to the pool of antimicrobial resistance genes and thereby cause a risk to human and animal health. For this reason, the European Food Safety Authority (EFSA) and other regulatory agencies have established guidelines and methodologies to limit this risk [13, 14]. Consequently, when dealing with non-pathogenic bacteria, acquired resistance mechanisms represent the major concern, as they could potentially spread horizontally to pathogenic bacteria. When using classical phenotypic susceptibility-testing assays, such as disc-diffusion and micro-dilution based methods, it is not possible to distinguish between intrinsic and acquired resistance [13]. In contrast, genome-based sequence analysis makes it possible to detect resistance mechanisms and to reveal the presence of mobile genetic elements carrying resistance genes. Whole genome sequence analysis is also very useful for studying the evolutionary relationships between bacterial species and sub-species and to investigate the evolution of resistance genes in bacteria including *Bacillus* species.

The genus *Bacillus* consists of Gram-positive spore-forming rods with the soil environment as their natural habitat [15, 16]. The genus contains several species of commercial significance. These are used for the production of enzymes for a variety of applications, bioprotection, as plant growth promoters, and as probiotic feed additives for domestic animals [17]. The species *B*. *licheniformis* and *B*. *paralicheniformis* are widely used commercially, for example as live organisms in feed applications, for plant protection and in aquaculture [17, 18].

With recent advances in genome sequencing technologies, draft genome sequences of several *B*. *licheniformis* strains have become publicly available. Recently, the genomes of 16 *B*. *licheniformis* strains were compared [19]. Based on phylogenetic and phenotypic analyses, these strains were separated into 2 distinct groups, of which one group was proposed to represent a novel species, designated *Bacillus paralicheniformis*. A recent study of more than 120 *Bacillus* strains including *B*. *licheniformis* and *B*. *paralicheniformis* found that the minimal inhibitory concentration (MIC) of erythromycin differed significantly between the tested *Bacillus* species; all *B*. *paralicheniformis* strains and 23% of the *B*. *licheniformis* strains were resistant to erythromycin, whereas all *B*. *megaterium*, *B*. *amyloliquenfaciens* and *B*. *velenzensis* isolates were susceptible[20]. Erythromycin resistance correlated with the presence of the *ermD* gene in the chromosome except for one *ermD*-positive strain which was susceptible to erythromycin [20]. This highlights the need to supplement phenotypic susceptibility testing with more detailed genomic analysis that could reveal intrinsic resistance when evaluating the risk posed by antimicrobial resistance in non-pathogenic bacteria. Erythromycin resistance encoded by *ermD* and intrinsic resistance to bacitracin encoded by an ABC transporter have been described previously [21, 22], but to our knowledge the correlation between the diversity of resistance genes and phylogenetic relationships of *B*. *licheniformis* and *B*. *paralicheniformis* strains has not been studied.

The objective of the present study was to assess the genetic background and evolutionary history of four putative resistance determinants, conferring resistance to erythromycin (*ermD*), streptomycin (*aadK* and *aph*) and chloramphenicol (*cat*), and to assess the potential for horizontal transfer of these putative resistance genes to other bacteria. We sequenced and compared the genomes of more than 100 *B*. *licheniformis* and *B*. *paralicheniformis* strains from different origins and time periods. The sequence composition of the putative resistance genes was analyzed and their flanking regions were assessed for the presence of mobile genetic elements. In addition, a phylogenetic analysis was performed at the whole-genome level as well as on the individual gene level to find evidence for lateral transfer of these putative antibiotic resistance genes in the strains. We integrated the results of both approaches to infer the evolutionary history of the antibiotic resistance genes in *B*. *licheniformis* and *B*. *paralicheniformis* strains and to assess whether the presence of these genes imposes a potential risk of transferring the resistance to other bacteria. Based on this analysis, we conclude that the putative *cat*, *aph*, *aadK* and *ermD* in *B*. *licheniformis* and *B*. *paralicheniformis* are most likely intrinsic and part of the ancient resistome and that no evidence supports the possibility of horizontal transfer of the genes to other bacteria.

## Materials and methods

### Bacterial strains

A collection of 104 strains, composed of *B*. *licheniformis* (73 strains), *B*. *paralicheniformis* (30 strains) and one strain (CHCC20375) which could not be definitively identified as either, was included in this study (S1 Table). The strains were chosen to represent a diverse collection based on source material, time span and geographic origin (Table 1). One strain, CHCC14814, was identified as *B*. *paralicheniformis*, but is more distantly related to the type strain than the other *B*. *paralicheniformis* strains in the collection. These strains were obtained from public strain collections, universities or isolated in different in-house projects at Chr. Hansen A/S. The strains originated from more than 15 different countries and more than 30 sources (e.g. food, animals, human, soil and other natural environments). Most strains were isolated over a timespan of seven decades (<1944–2014); one strain was isolated from a can of tinned veal from an arctic expedition, sealed around 1825. All strains were deposited in the Chr. Hansen Culture Collection (CHCC) [23].

**Table 1:** Origin of strains included in the study.

Species	Source	Year of isolation	Geographic origin
*Bacillus licheniformis*^a^^)^	Human faeces (2), Animal faeces (17), Animals other (8), Food (16), Feed (2), Soil (12), Plants (2), Environment (9), Not given (6)	1878 (1), 1917 (1), 1921 (1), <1944 (1), 1944 (1), <1950 (1), <1952 (1), <1956 (1), 1958 (1), <1963 (1), <1964 (1), <1976 (1), <1979^b^^)^ (3), <1978 (1), <1980 (1), 1984 (1), 1993 (4), 1994 (2), <1996 (4), <2000 (1), 2000 (1), 2005 (1), 2010 (2), 2011 (27), 2012 (1), Not given (13)	Denmark (16), France (1), Germany (12), United Kingdom (3), Spain (2), The Netherlands (2), Norway (2), Sweden (2), Sudan (4), Egypt (2), Philippines (2), Vietnam (3), Japan (1), Australia (2), USA (2), Not given (18)
*Bacillus paralicheniformis*	Animal faeces (5), Animals other (1), Food (5), Soil (10), Plants (1), Environment (2), Not given (6)	<1950 (1), <1951 (3), <1953 (1), <1954 (1), 1965 (1), <1977 (1), <1980 (1), <1986 (1), 2010 (1), 2011 (6), 2013 (2), 2014 (1), Not given (10)	Denmark (3), Germany (2), United Kingdom (1), Spain (2), Ghana (1), Sudan (2), China (1), Australia (1), USA (3), Not given (14)

Numbers in brackets represent the number of strains; < in front of year indicates that only the date of deposit is known;

a) CHCC20375 could not be definitively identified as either *B*. *licheniformis* or *B*. *paralicheniformis*, but is included in the *B*. *licheniformis* group here;

b) CHCC20323 was isolated from a can of tinned veal sealed around 1825, however, the strain was deposited in 1979 and hence the year of isolation is given here as <1979

Twenty-six of the *B*. *paralicheniformis* strains, 34 of the *B*. *licheniformis* strains and strain CHCC20375 were previously tested for susceptibility to chloramphenicol, erythromycin, clindamycin and streptomycin by use of a two-fold broth micro-dilution method allowing a determination of the MIC for each antimicrobial [20]. The additional strains used in the current study, two *B*. *paralicheniformis* and 38 *B*. *licheniformis*, were tested in the same way. Moreover, the following strains were also characterized: *B*. *licheniformis* DSM 13 (ATCC 14580), *B*. *paralicheniformis* ATCC 9945A and BL-09, *Bacillus cereus* DSM 7459 and *Bacillus sonorensis* DSM 12369.

Additional details on the strains and the relevant MIC values can be found in S1 Table.

### DNA extraction, sequencing and genome assembly

DNA sequencing was performed at BaseClear (Leiden, the Netherlands) for most strains, using Illumina Hiseq2500 sequencing with 125 bp paired-end read length. Cell pellets were provided, and DNA was extracted at BaseClear using ZR Fungal/Bacterial DNA kit (Zymo Research; D6005), according to the manufacturer’s protocol. For 17 strains, total DNA extraction was done in house using a QIAcube and the Qiagen blood and tissue kit (Hilden, Germany), according to the manufacturer’s protocol. The DNA samples were sent to BGI (Shenzhen, China) for Illumina Hiseq2500 sequencing with 125 bp paired-end read length.

The paired-end reads obtained were assembled *de novo* using CLC genomic workbench (version 7.3.3; Qiagen Bioinformatics, Aarhus, Denmark) as previously described [20]. The sequence quality was assessed and only *B*. *licheniformis* and *B*. *paralicheniformis* sequences with an average coverage of >45x and a total contig length between 4 and 4.8 MB were considered acceptable. The minimum contig size accepted was 200 bp.

### Genome sequences

The genome sequences of *Bacillus cereus* DSM 7459, *Bacillus sonorensis* DSM 12369 and the 104 *B*. *licheniformis and B*. *paralicheniformis* strains from CHCC were deposited in the NCBI/GenBank database under biosample numbers SAMN02603262, SAMN2603685, SAMN03272542, SAMN05583215 to SAMN05583319 as indicated in S1 Table. The complete circular genome sequences of *B*. *paralicheniformis* 9945A [24], *B*. *paralicheniformis* BL-09 (GenBank Accession CP010524), *B*. *licheniformis* DSM 13 [25] and *Bacillus subtilis* 168 (GenBank Accession NC000964.3) were retrieved from the NCBI GenBank sequence database, April 2015 (http://www.ncbi.nlm.nih.gov/) while *B*. *paralicheniformis* KJ-16 (LBMN02.1.fsa_nt.gz) [19] and *B*. *sonorensis* KCTC 13918 (AYTN01.1.fsa_nt.gz) were downloaded in October 2017. Nucleotide sequences of representative antibiotic resistance genes from other bacterial species and their translation products were also obtained from GenBank and are presented in the respective figures with species name and GenBank access number.

### Species identification and multi locus sequence typing (MLST)

Species identification was performed by whole genome average nucleotide identity (ANI) calculation with the help of the JSpecies software using default parameters [26]. ANI values of 96% relative to the type strains *B*. *licheniformis* DSM 13, B. *paralicheniformis* KJ-16 and *B*. *sonerensis* KCTC 13918, respectively, were used as species thresholds. MLST typing of whole genome sequences was performed using the microbial genomics module of the CLC software (version 2.5.1) employing the MLST scheme developed for *Bacillus licheniformis* [27]. Novel sequence types (STs) were submitted to the *B*. *licheniformis* MLST database to obtain an ST number.

### Genome annotation

The Rapid Annotation using Subsystems Technology (RAST) server [28] was used, with the default settings, to find open reading frames (ORFs) that could potentially code for proteins and to provide an automatic annotation of encoded functions. The three reference genomes of *B*. *licheniformis* and *B*. *paralicheniformis* were reannotated using RAST to allow for a better comparison of all genome sequences. The obtained contigs of draft genomes were re-oriented and ordered (scaffolded) by aligning to the circular reference genome templates using *ad hoc* python scripts.

The RAST annotations were queried to identify putative resistance genes by text searching the excel file which can be downloaded together with the annotated genome. Genes with an annotation containing words related to antibiotic resistance (e.g antimicrobial, resistance, antibiotic), and adjacent genes, were analyzed manually to improve the functional annotations by comparing the gene sequence to curated protein sequence databases using BLAST (http://blast.ncbi.nlm.nih.gov/), InterproScan (http://www.ebi.ac.uk/Tools/pfa/iprscan/) [29] and ClustalW (http://www.ebi.ac.uk/Tools/msa/clustalw2/) [30]. The genomic context of the genes of interest was examined using KEGG PATHWAY (http://www.genome.jp/kegg/pathway.html), and MGcV (Microbial Genome context Viewer; http://mgcv.cmbi.ru.nl) [31].

### Comparative genomics

All protein sequences potentially encoded in the genomes were compared using OrthoMCL [32]. OrthoMCL uses all-against-all protein BLAST, where it groups proteins which have higher sequence similarity within the species than with proteins outside the species. In this way, orthologs (genes in different species that evolved from a common ancestral gene by speciation) are separated from paralogs (genes related by duplication within a genome). In specific cases, when an orthologous group (OG) contained more than one gene per strain (*i*.*e*. highly similar genes), this OG was manually split into separate OGs containing only one gene per strain, and these separate OGs were positioned as indicated by contig assembly and template alignments.

The OrthoMCL output matrix containing OGs, *i*.*e*. gene families, was used as input to generate a database in MS Excel in which the information about the location (contig-level coordinates) and length of orthologous proteins of all *B*. *licheniformis* and *B*. *paralicheniformis* genomes were aligned. Multiple sequence alignment (MSA) files were made using Muscle [33] where the nucleotide and protein sequences within OGs were aligned, to facilitate identification of pseudogenes (encoding incomplete proteins), and to identify correct start codons. Color-coded multiple sequence alignments were made of the putative resistance proteins using Clustal Omega (https://www.ebi.ac.uk/Tools/msa/clustalo/).

### Phylogeny

All OGs with a single gene copy in each of the strains were selected based on the OrthoMCL output. The protein sequences in these OGs were aligned using Muscle [33, 34] and alignment positions with amino acid differences were isolated and stored in a single artificial protein sequence. This protein sequence was used as a basis to generate a whole-genome-based phylogenetic tree using FastTree [35]. Rerooting of the tree was performed using the most distant strain on the basis of species description or, if all strains were of the same species, on the basis of the longest branch length.

### Identification, characterization and phylogenetic relationship of (putative) antimicrobial resistance genes

The macrolide resistance gene *ermD* was previously identified in 36 out of 63 strains [20] by use of ResFinder (http://www.genomicepidemiology.org/) [36]. The remaining 41 strains were subsequently screened for resistance genes by use of ResFinder; of these, 15 strains were found to have the *ermD* gene.

To identify putative streptomycin or chloramphenicol resistance genes a text search of the RAST annotated function using the terms “chloramphenicol” and “aminoglycoside” as performed. The identified genes and adjacent genes were compared with sequences in NCBI database using BLAST in order to improve the functional annotation or qualify it.

ErmD and Putative resistance proteins were compared for diversity at the single protein level by aligning the sequences with Clustal 2.1, Clustal Omega or Muscle using default settings. Phylogenetic trees and manual assessment of SAP variations was used for determination of protein variant types and the variance types was compared to the whole-genome phylogenetic tree of the strains and to the MIC value of the antibiotic in question.

The flanking region of each of the putative resistance genes in all genomes were aligned and ordered based on contig alignments to the template genomes, to determine the location of the genes and their organization in the genomes. Finally, an NCBI GenBank BLAST search using the DNA or amino acid sequence of putative resistance genes as query sequence with default settings (Program settings: megablast; discontiguous megablast; and protein protein BLAST) was performed to evaluate similarity to known resistance genes in other bacteria.

### Horizontal gene transfer detection

HGTector (https://github.com/DittmarLab/HGTector) [37] was used to search for genes that may be acquired by horizontal gene transfer using standard settings and the NCBI non-redundant protein database (NRDB) of Jan 28, 2017. This tool identifies genes that fall beyond a series of statistically determined thresholds as not adhering to the typical vertical history of the organisms in question, but instead having a putative horizontal origin.

## Results and discussion

### Sequencing and assembly of contigs

The published genome sequences of *B*. *licheniformis* DSM 13 (4.22 Mb, 46.2% GC) and *B*. *paralicheniformis* strains BL-09 (4.39 Mb, 45.9% GC) and ATCC9945A (4.38, 45.9% GC) consist of a single circular contig, representing the chromosome, and no plasmids. We have sequenced an additional 101 strains of which 28 were identified by ANI as *B*. *paralicheniformis*, and 72 as *B*. *licheniformis*; one strain, CHCC20375, was closely related to both species, but could not be assigned to either species, so is designated *B*. *paralicheniformis/licheniformis* (S1 Table). The 72 draft genomes of *B*. *licheniformis* ranged in size from 4.08–4.73 Mb, with a GC content of 45.3–46.3%; the 28 *B*. *paralicheniformis* draft genomes ranged in size from 4.25–4.55 Mb, with a GC content of 45.5–46.0%; while the *B*. *paralicheniformis/licheniformis* strain had a genome size of 4.16 Mb, and a GC content of 45.9%. (S2 Table). The true genome sizes are slightly larger since repeat sequences longer than the read length (e.g. IS elements, rRNA genes) are assembled into single contigs in draft genomes and hence are only counted once. The size and GC content are as expected for the species. We conclude that the sequences obtained are free of contamination and of a sufficient quality for the phylogenetic analysis.

### Representativeness of the strain collection

The strains analysed comprise a diverse collection of strains collected over a time span of more than seven decades representing very different origins and geographic areas. MLST analysis and a comparison to known MLST types of both species showed the collection to represent most known sequence types (STs). Moreover, 18 novel STs were found among 23 strains (see S1 Fig and S1 Table). *B*. *paralicheniformis/licheniformis* strain CHCC20375 did not share alleles with any of the other strains, confirming the strain is different from both species.

### Orthology analysis

The total number of OGs found was about 11,500. Nearly all of these could be scaffolded (*i*.*e*. ordered in the correct genome context), based on comparison of the assembled contigs with the three circular reference genomes. This number of OGs represents a rough estimate of the current pangenome of the evolutionary clade consisting of *B*. *licheniformis* and *B*. *paralicheniformis*. This estimate is likely to be falsely high, due to the inclusion of OGs representing fragments of proteins (encoded by pseudogenes) and over-prediction of genes by RAST (generally small ORFs).

We found 2915 genes representing the core genome of this evolutionary clade. These genes were present, in single copies, in every *B*. *licheniformis* and *B*. *paralicheniformis* genome, as well as in the CHCC20375 genome.

### Phylogeny based on predicted core proteins

Phylogenetic trees were made based on the amino acid variations in all 2915 predicted core proteins. A core genome phylogenetic tree was made including the genomes of *B*. *subtilis* 168, and the newly sequenced *B*. *cereus* DSM 7459, *Bacillus sonorensis* DSM 12369 strains which were used as outgroups (S2 Fig). The tree shows that the strains identified as *B*. *licheniformis* or *B*. *paralicheniformis*, as well as CHCC20375 are more phylogenetically related to each other and the relevant type strains than to the other *Bacillus* species, confirming that these strains truly are *B*. *licheniformis*, *B*. *paralicheniformis* or, in the case of CHCC20375, a closely related species.

**Fig 1:**
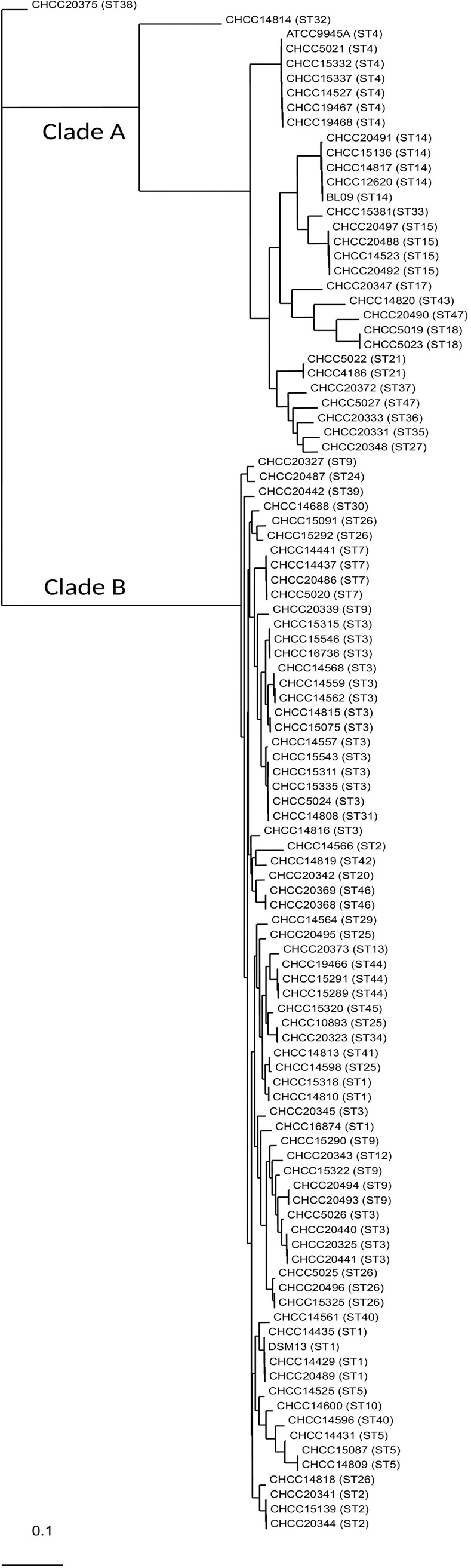
Whole genome phylogenetic tree. Number in parentheses; Multi Locus Sequence Type. Clade A corresponds to *B*. *paralicheniformis* and clade B to *B*. *licheniformis*.

The predicted core proteins in the 73 *B*. *licheniformis* and 30 *B*. *paralicheniformis* strains as well as CHCC20375 were analysed separately. We found 33,796 single amino acid polymorphisms (SAPs) in the 2915 conserved core proteins. These SAPs were used to generate a phylogenetic tree which included the 104 strains, but not other *Bacillus* species (Fig 1). The *B*. *paralicheniformis* and *B*. *licheniformis* genomes separate into two distinct clades designated A and B, respectively.

These two distinct clades correspond to the two phylogenetic groups recently proposed by Dunlap et al. [19], *i*.*e*. group 1 containing *B*. *licheniformis* strains including the *B*. *licheniformis* reference strain DSM13 (clade B in our study), and group 2 containing *B*. *paralicheniformis* strains including the *B*. *paralicheniformis* reference strains ATCC9945A and BL-09 (clade A in our study). The main gene clusters and functions previously proposed to be unique for either group 1 or group 2 strains [19], were indeed found to be largely unique for our clade B or clade A, respectively (S3 Table); we therefore conclude, that clade A is *B*. *paralicheniformis* and clade B is *B*. *licheniformis*.

The MLSTs deduced from the genome sequences followed the clade sub-branches for *B*. *paralicheniformis*, but for *B*. *licheniformis* the sequence types were more dispersed (Fig 1). The most common types found in the MLST database, ST1, ST3 and ST9, were also the most common ones found in the present study; some clustering was seen but for all three types, strains were found in at least two different sub-branches. Thus, the current MLST scheme [27] is not ideal for revealing phylogenetic relationships among *B*. *licheniformis* strains.

### Identification, phylogenetic analysis and phenotypic effect of putative antimicrobial resistance genes

The text search of the RAST annotated function using the terms “chloramphenicol” and “aminoglycoside” identified two and three genes, respectively. A manual assessment of these genes and adjacent genes using nucleotide and amino acid BLAST revealed three genes to have some sequence similarity to genes known to confer resistance to the respective antibiotics. These putative resistance genes where found in all *B*. *licheniformis* and *B*. *paralicheniformis* strains. One gene encodes a putative chloramphenicol acetyltransferase (CAT; EC 2.3.1.28), which may cause reduced susceptibility to chloramphenicol. Two genes, adjacent to each other, encode a putative aminoglycoside 6-adenylyl transferase (AadK; EC 2.7.7) and a putative aminoglycoside 3'-phosphotransferase (APH; EC 2.7.1.B26), which may cause reduced susceptibility to streptomycin and other aminoglycosides.

We previously reported the presence of an N6-methyltransferase gene, *ermD*, conferring resistance to erythromycin [38], in the chromosome of several strains included in the present study [20]. Screening of the remainder of the strains revealed that, in this collection, all 30 *B*. *paralicheniformis* strains, 20 of 73 *B*. *licheniformis* strains and the *B*. *paralicheniformis/licheniformis* strain CHCC20375 harbor *ermD*.

The sequences were compared for identity to known resistance genes in Genbank and for correlations to the MIC values of the strains (S1 Table). Moreover, *ermD* and the three putative antimicrobial resistance mechanisms were analyzed further for their diversity, location and organization in the genomes of the 104 strains.

#### Putative chloramphenicol resistance proteins (CAT)

Chloramphenicol is a bacteriostatic antibiotic whose activity is based on a reversible binding to the peptidyltransferase centre in the 50S ribosomal subunit of 70S ribosomes [39].

The molecular basis of bacterial resistance to chloramphenicol has been reviewed by Schwarz et al. [40]. The most frequently encountered mechanism of bacterial resistance to chloramphenicol is enzymatic inactivation by acetylation of the drug via different types of chloramphenicol acetyltransferases (CATs). Classical CATs (so-called Type A [40]) have been detected in a wide variety of bacteria [41, 42].

**Fig 2:**
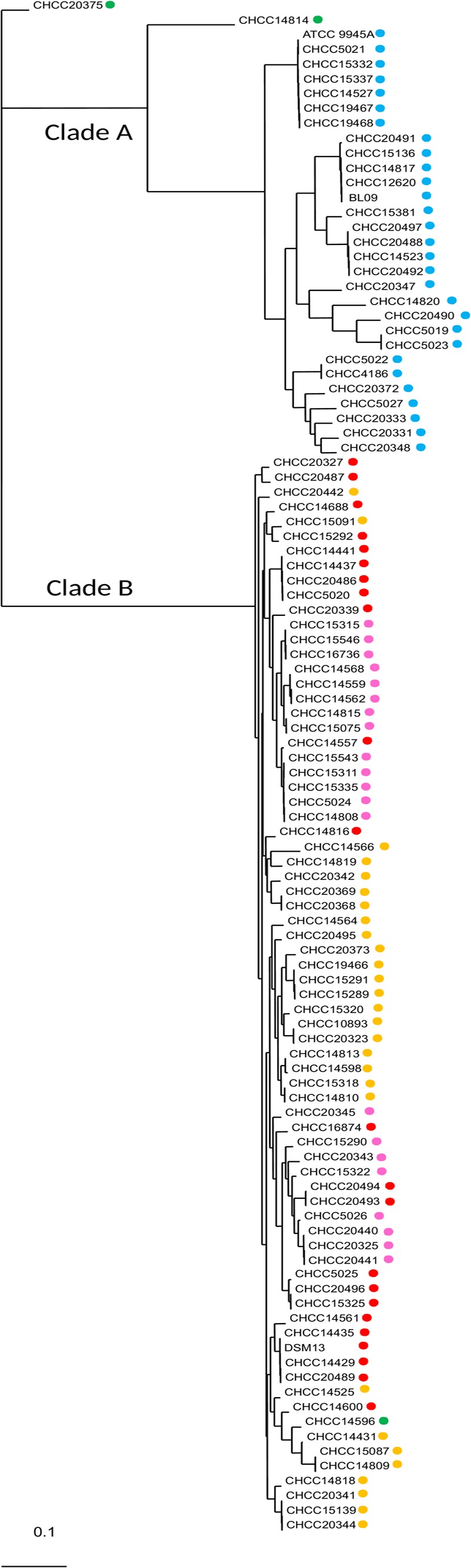
Distribution of CAT protein variants in the phylogenetic tree. Number in parentheses; Multi Locus Sequence Type. Clade A corresponds to *B*. *paralicheniformis* and clade B to *B*. *licheniformis*. The colored dots refer to the CAT types shown in Table 1: Variant 1 (blue dots); Variant 2 (pink dots); Variant 3 (amber dots); Variant 4 (red dots); Outliers (green dots).

We found a putative *cat* gene in all 104 genomes. The *cat* gene is predicted to encode a chloramphenicol O-acetyl transferase (EC 2.3.1.28; OG_3041) of 200–216 amino acids. A multiple sequence alignment of these CAT proteins is shown in S3 Fig. A manual analysis suggests there are four main sequence variants (Table 2); these are plotted on the whole genome phylogenetic tree in Fig 2.

**Table 2:** Variants of chloramphenicol acetyltransferase (CAT) proteins and the corresponding MIC.

CAT type*	No. of strains	0 SAPs	1 SAP	2 SAPs	3 SAPs	Species	MIC
Variant 1	29	16		10	3	*B*. *paralicheniformis*	8(15), 16(12),32(2)
Variant 2	21	21				*B*. *licheniformis*	4(20), 8(1)
Variant 3	28	18	10			*B*. *licheniformis*	8(1), 16(22), 32(5)
Variant 4	23	14	6	3		*B*. *licheniformis*	4(1), 8(5),16(16), 32(1)

*Two outlier strains (CHCC14814 and CHCC20375) and *B*. *licheniformis* CHCC14596 have more variation and did not fit into any of the four CAT types.

SAP (single amino acid polymorphism); MIC (minimal inhibitory concentration); number in parentheses refer to the number of strains.

The MIC values were found to be at or above the cut-off value recommended by EFSA (8 mg/L)[43] for 83 of the 104 strains (S1 Table). These strains all harbored a full-length version of the *cat* gene (except for one strain with MIC 8 mg/L). The 21 strains which had a truncated version of the gene, designated variant 2, were susceptible to chloramphenicol (except for one strain with MIC 8 mg/L); it is therefore likely that the *cat* gene is responsible for this phenotype in most strains.

The sequence variants 1–4 follow the whole-genome core tree (Fig 2), suggesting that they are changing due to normal Darwinian evolution together with all other genes. Variant 1 was found only in *B*. *paralicheniformis*, while the other 3 variants were found only in *B*. *licheniformis*, generally following the subclades (with some exceptions). Variant 1 sequences differed by >15 SAPs from the other 3 variants; these 3 latter variants differed by at most nine SAPs from each other, and variants 2 and 3 differed only by one SAP and the C-terminal truncation. The *B*. *paralicheniformis*/*licheniformis* strain (CHCC20375) and the more distantly related *B*. *paralicheniformis* strain (CHCC14814) and one additional *B*. *licheniformis* strain (CHCC14596) (outliers: green dots) had greater variation in their amino acid sequences.

A comparison of the *B*. *licheniformis* and *B*. *paralicheniformis* CAT proteins with Type A representatives of the 16 previously defined CAT groups found in other species [40] showed that they were distinct (S4 Fig). Moreover, a BLAST analysis showed that the *B*. *licheniformis* and *B*. *paralicheniformis* CAT proteins (and genes) were >90% identical; the closest related CAT in other species was from *B*. *subtilis* (71% amino acid identity, 74% nucleotide identity). The current classification distinguishes CAT groups on the basis of sequence identity of less than 80% at the amino acid level [40] so, the *B*. *licheniformis* and *B*. *paralicheniformis cat* genes represent a distinct group.

The *cat* genes in the 104 strains were found to be in the same genomic location in a highly conserved region of the chromosome, (S5 Fig; for details see S4 Table**)**. The flanking genes were always the same and encode a bifunctional DNA-binding transcriptional regulator/O6-methylguanine-DNA methyltransferase AdaA (OG_3042) and a methylated-DNA-protein-cysteine methyltransferase AdaB (EC 2.1.1.63; OG_3043). The only genomic context variation found in this region was an additional four genes encoding a potassium-transporting ATPase (EC3.6.3.12; TC 3.A.3.7.1). These four genes were found in all strains of *B*. *paralicheniformis* (except CHCC14814, the phylogenetically borderline *B*. *paralicheniformis* strain) and were absent in all *B*. *licheniformis and the* CHCC20375 which was closely related to both species, suggesting that this transporter was acquired or lost at the time the two clades split.

There were no mobile genetic elements (IS elements, transposons, phages) in this region. The G+C content of the *cat* gene is 43–44%, which is close to the average 46% of the entire chromosome, which further supports this putative gene being intrinsic and not acquired by horizontal gene transfer.

#### Putative streptomycin/aminoglycoside resistance proteins (AadK and Aph)

Streptomycin belongs to the family of aminoglycoside antibiotics [44]. There are several mechanisms that contribute to the development of aminoglycoside resistance. These include the deactivation of aminoglycosides by enzymes which can cause acetylation via aminoglycoside acetyltransferase (AAC), adenylation via aminoglycoside nucleotidyltransferase (ANT) or phosphorylation via aminoglycoside phosphotransferase (APH) [45–47]. Enzymatic modification of the amino or hydroxyl groups of aminoglycosides causes them to bind poorly to ribosomal RNA allowing bacteria to survive [48].

Two adjacent genes on the chromosome of all 104 *B*. *licheniformis* and *B*. *paralicheniformis* strains encode a putative aminoglycoside 6-adenylyl transferase (AadK; EC 2.7.7.-; OG_2048) of 289–296 amino acids, and a putative aminoglycoside 3'-phosphotransferase (APH; EC 2.7.1.B26; OG_2049) of 315 amino acids. The MIC value for streptomycin was found to be at or above the EFSA cut-off value for *Bacillus* (8 mg/L) [43] for nearly all strains (S1 Table), consistent with these genes resulting in reduced susceptibility to streptomycin.

Multiple sequence alignments of all AadK proteins and all APH proteins are shown in S6 and S7 Figs, respectively. For both proteins, there was very little variation in the amino acid sequences. The AadK proteins only show two sequence variants: variant 1 was present only in *B*. *paralicheniformis*; variant 2 was present only in *B*. *licheniformis* (Table 3). Among the strains with variant 2, 22 had an insertion of seven amino acids. The range of MICs of streptomycin was similar among the variant 2 strains; strains with the insert had MICs in the range 4–64 mg/L while strains without the insert had a range of 4–32 mg/L. The proteins from CHCC20375 and CHCC14814 showed more variation and could not be grouped into any of the sequence type variants. These data do not support a role for *aadK* and *aph* in streptomycin resistance in *B*. *licheniformis* and *B*. *paralicheniformis* as there is no correlation between sequence type and the MIC to streptomycin. However, the presence of these genes may influence streptomycin resistance as *B*. *licheniformis* and *B*. *paralicheniformis* strains in general are less susceptible to streptomycin than other *Bacillus* species [20].

**Table 3:** Sequence type variants of AadK proteins and corresponding MIC.

AadK type*	No. of strains	0 SAPs	1 SAP	2 SAPs	insert	MIC	Phylogeny clade
Variant 1	29	21	7	1		4(5),8(9),16(11),32(4)	*B*. *paralicheniformis*
Variant 2	73	51			22	4(3), 8(18), 16(38), 32(13),64(1)	*B*. *licheniformis*

*B. *paralicheniformis*/*licheniformis* strain (CHCC20375) and the more distantly related *B*. *paralicheniformis* strain (CHCC14814) have more variation and did not fit into either of the two AadK types.

SAP (single amino acid polymorphism); MIC (minimal inhibitory concentration); number in parentheses refers to number of strains.

All *B*. *licheniformis* and *B*. *paralicheniformis* AadK proteins have at least 93% identity in amino acid sequence, whereas the next closest orthologs were from the recently described *Bacillus glycinifermentans* [49] (78% amino acid identity, 76% nucleotide identity) and *B*. *sonorensis* (78% amino acid identity). Similarly, all the *B*. *licheniformis* and *B*. *paralicheniformis* APH proteins had at least 88% identity in amino acid sequence, whereas the next closest orthologs were from *B*. *glycinifermentans* (61% amino acid identity, 66% nucleotide identity).

The *aadK* and *aph* genes were found adjacent to each other and present in the genomes of all *B*. *licheniformis* and *B*. *paralicheniformis* strains and always at the same position in the chromosomes (S8 Fig). However, there was some variation in the surrounding genes. The directly downstream gene was always a pyrimidine nucleotidase (YjjG family, HAD superfamily; OG_2050), which is also present in *B*. *subtilis* 168 (BSU07330). Further downstream there were an additional five genes encoding a GABA permease (OG_3930), and (in the opposite orientation) a citrate transporter with a 2-component regulator (see S4 Table for details). These additional genes were present in all *B*. *licheniformis* strains, while in *B*. *paralicheniformis* only strain CHCC20331 had this extra set of five genes.

Upstream there was also some variation in genome context. All *B*. *paralicheniformis* strains had an upstream gene encoding a putative erythromycin esterase (Pfam05139; OG_4637); in *E*. *coli* these enzymes confer resistance to the antibiotic erythromycin [50, 51]. However, the amino acid sequence identity was only 20–21% to the protein in *E*. *coli*. The *B*. *subtilis* ortholog, BSU02310 (hydrolase YbfO, 446 AA), had only 31% amino acid identity. Furthermore, seven strains of *B*. *paralicheniformis* had extra genes for lantibiotic biosynthesis and/or resistance (3 variants of genes) further upstream: strains CHCC20372, CHCC20331, CHCC20333, CHCC5027, CHCC20347, CHCC14820, and CHCC14814 (not shown in S8 Fig). These strains clustered in 2 subclades, so these lantibiotic biosynthesis genes were probably acquired more recently by horizontal gene transfer.

No mobile genetic elements (IS elements, transposons, phages) were found in this region. The G+C content of the *aadK* and *aph* genes was 49% and 47% respectively, which is close to the average 46% of the entire chromosome (S8 Fig).

#### Erythromycin/macrolide resistance protein ErmD

Erythromycin belongs to the macrolide class of antibiotics, which inhibit protein synthesis by stimulating dissociation of the peptidyl-tRNA molecule from the ribosome during elongation. This results in chain termination and a reversible stoppage of protein synthesis [38]. The resistance mechanisms described includes rRNA methylases and efflux systems. A large number of rRNA methylase genes (*erm*) have been described; all *erm* enzymes methylate the same adenine residue, resulting in a macrolide-lincosamide-streptogramin B resistance (MLS_B_) phenotype. Nomenclature for macrolide and *erm* genes is defined by Roberts et al. [38], which divide the genes into classes based on the percentage of amino acid sequence similarity.

**Fig 3:**
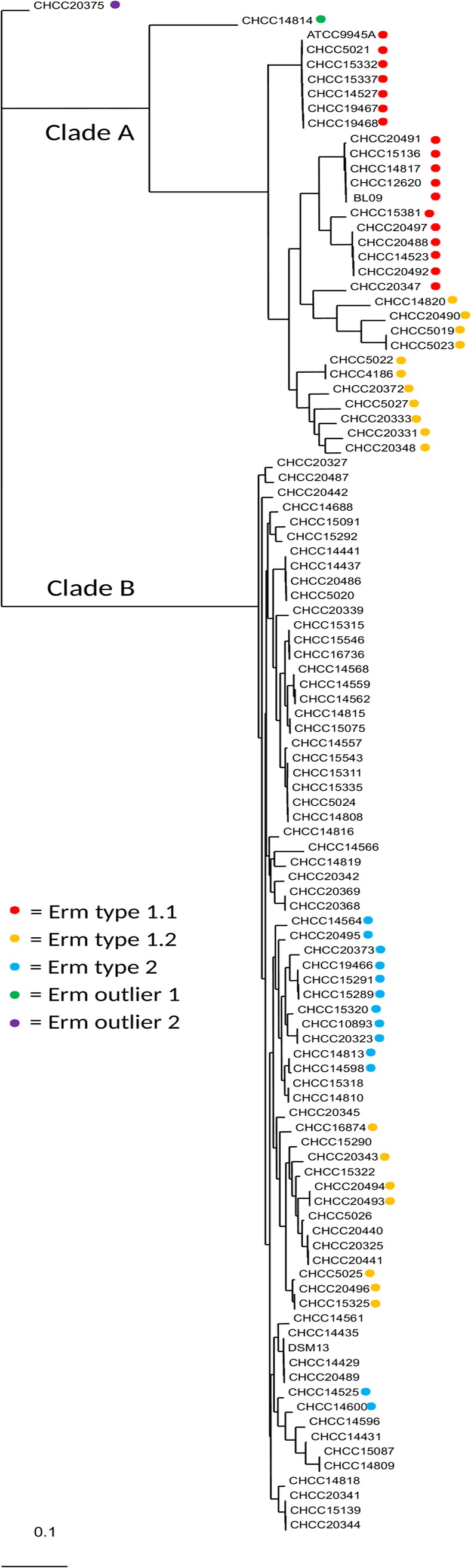
Distribution of ErmD protein variants in the phylogenetic tree. Number in parentheses; Multi Locus Sequence Type. Clade A corresponds to *B*. *paralicheniformis* and clade B to *B*. *licheniformis*. The colored dots refer to the ErmD variants shown in Fig 4: Erm type 1.1 (red dot); Erm type 1.2 (amber dot); Erm type 2 (blue dot); Erm outlier 1 CHCC14814 (green dot); Erm outlier 2 CHCC20375 (purple dot).

**Fig 4:**
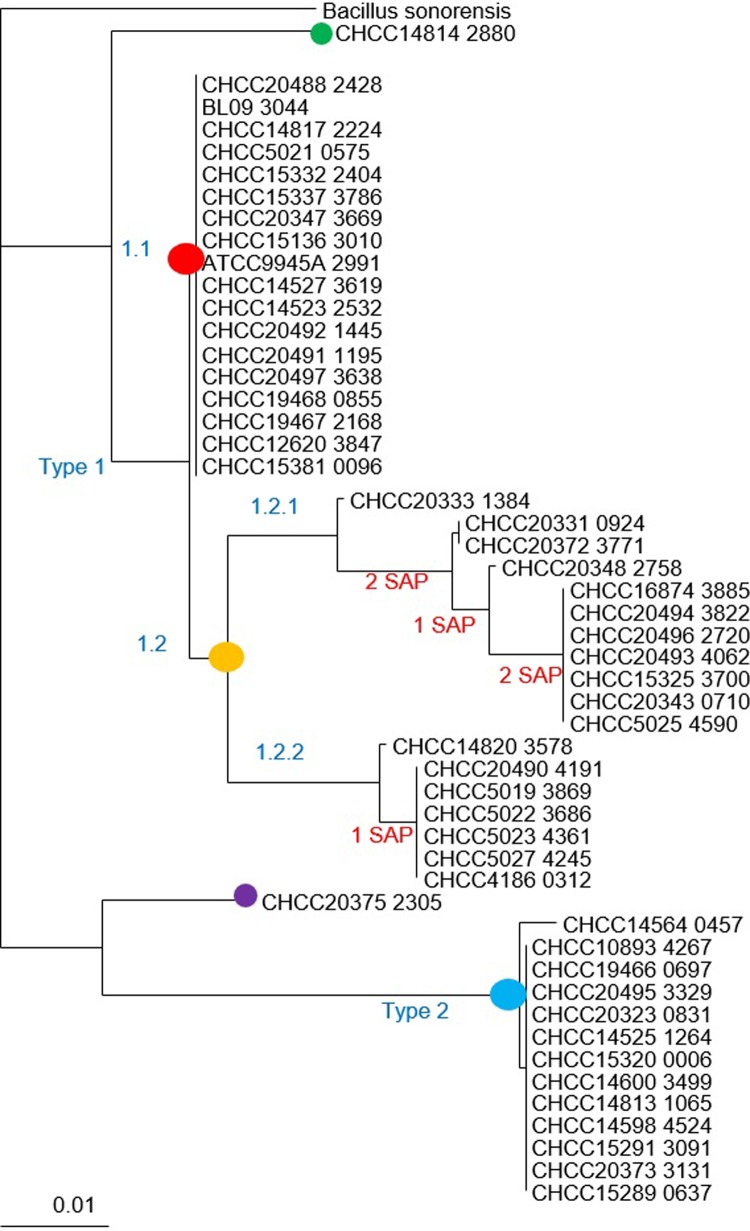
Phylogenetic tree of ErmD proteins. The tree was built using approximately-maximum-likelihood algorithms (Fasttree, see methods) and rooted on the longest branch length. To assess branch length support, Shimodaira-Hasegawa tests were performed in 1,000 resamplings. Each strain code is followed by the gene locus tag number. Three previously published sequences in the ErmD class are included, i.e. L08389 [52], M29832 [53] and M77505 [22]. Sequences of strains to the right of a vertical line are 100% identical. The colored dots designate the ErmD variants: Erm type 1.1 (red dot); Erm type 1.2 (amber dot); Erm type 2 (blue dot); Erm outlier 1 CHCC14814 (green dot); Erm outlier 2 CHCC20375 (purple dot).

We found an *ermD* gene, encoding rRNA adenine-N6-dimethylase (EC 2.1.1.182; OG_4245) of 286–287 amino acids, to be present in all 30 *B*. *paralicheniformis* strains, in 20 of 73 *B*. *licheniformis* strains and in the *B*. *paralicheniformis/licheniformis* strain (CHCC20375). The presence/absence of *ermD* genes was plotted on the whole genome core tree (Fig 3). The presence of the *ermD* gene correlated nearly perfectly with the observed erythromycin resistance of these strains; the only exception was strain CHCC14564 which had the gene but was not resistant to erythromycin (S1 Table). This strain has a unique SAP (T_121_ -> I_121_). A multiple sequence alignment of all ErmD proteins is shown in S9 Fig, and a phylogenetic tree depicting the evolutionary relationship of these ErmD protein sequences is shown in Fig 4. Four ErmD protein variants were identified.

Variant 1.1 (red dot) was present in 18 strains of *B*. *paralicheniformis*. Strain CHCC14814 (green dot), which was phylogenetically more borderline in the *B*. *paralicheniformis* clade, had an ErmD sequence that differs by 8 SAPs from variant 1.1 sequences. Variant 1.2 (amber dot) separates into two branches, which differ by up to six SAPs. Variant 1.2.2 occurs only in *B*. *paralicheniformis*, while variant 1.2.1 occurs in both species. Variant 2 (blue dot) was found only in *B*. *licheniformis*, and 12 out of 13 sequences were identical. Outlier strain CHCC20375 (purple dot) had an ErmD sequence that differed by 15 SAPs from variant 2 sequences. All variant 1 sequences differ by 15–17 SAPs from variant 2 sequences. The gene with closest identity to *ermD* on the amino acid level was annotated by RAST as an rRNA methylase in the closely related species *B*. *sonorensis* (strain CHCC20335). The gene product branched separately from the other *ermD* genes and was more distantly related; strain CHCC20335 is susceptible to erythromycin (MIC 0.5 mg/L). Adimpong et al., 2012 [21] also found *B*. *sonerensis* strains to be susceptible to erythromycin. Nearly all of the type 2 sequences were found in one sub-branch of *B*. *licheniformis*, and most type 1.2.1 sequences were found in another sub-branch of *B*. *licheniformis* (Fig 3).

The *ermD* genes were found in the same position in all the *B*. *licheniformis* and *B*. *paralicheniformis* chromosomes, but there was considerable variation in the gene context surrounding the *ermD* gene (example in S10 Fig; details in S4 Table**).** All strains that had an *ermD* gene also had a flanking gene encoding a tautomerase (OG_4246), in the opposite orientation downstream of *ermD*. Two flanking genes encoding a methyltransferase (OG_3469) and a DNA alkylation repair protein (OG_3021) were present in the same location of all genomes except in CHCC14566. A general difference between *B*. *licheniformis* and *B*. *paralicheniformis* genomes was that all *B*. *licheniformis* have two additional genes immediately upstream to *ermD* of which one encodes an acetylesterase (OG_3973; S9 Fig). Other specific differences were found in this region in smaller sets of strains. Seven strains had additional genes, encoding a zinc transporter and a gluconate permease, flanking the *ermD* gene of type 1.2.1 in a sub-branch of *B*. *licheniformis* (yellow dot in Fig 4). Two strains, CHCC20333 and CHCC20348, had 20 additional genes downstream of *ermD* (type 1.2.1); of these, five were identified by HGTector as potentially transferable, but a manual analysis revealed no genes related to transferable elements (for details see S4 Table). There were no mobile genetic elements (IS elements, transposons, phages) in this region in any of the 104 strains; so, although some variation was found, especially downstream of *ermD*, this does not appear to be caused by the integration of mobile genetic elements.

Upstream of the *ermD* gene is a long leader region encoding two short leader peptides, which may be involved in *ermD* regulation and control of methylase expression at the translational level [54, 55]. An alignment was made of upstream regions of representatives of the four sequence variants (S11 Fig). It appears that all upstream regions were highly similar and encode the same two leader peptides suggesting *ermD* to be regulated in both species as described previously for other *erm* genes [54, 55]. Moreover, a previous study showed all four *ermD* variant types to be inducible more than seven-fold by the presence of low concentrations of erythromycin [20]. The *ermD* gene has a lower GC content than the rest of the chromosome (38.1–38.7 GC% in *ermD* and approximately 46% in the genome), which could be due to the presence of the attenuation-mediated transcriptional regulation and a normal low level of expression. GC content is not always uniform in bacterial genomes; the GC content tends to be higher in highly expressed genes [56].

Genes in the *ermD* class were previously reported to share >97% amino acid sequence identity; these include *erm* genes from *B*. *licheniformis* and *B*. *paralicheniformis* [22, 38, 53, 57] as well as from a strain isolated from soil in Korea and listed as *B*. *anthracis* [52]. This *B*. *anthracis* strain was identified by phenotypic methods and the 16S rDNA sequence of the strain is not publicly available to our knowledge. We believe the methods used were not sufficient for the correct species identification of the strain and suggest it to be either *B*. *licheniformis* or *B*. *paralicheniformis*.

The presence of *ermD* on a plasmid was previously reported [21]. In that study, the authors investigated eight strains of *B*. *licheniformis* with *ermD* and suggested, based on a PCR analysis of what they believed to be plasmid DNA, that *ermD* is present on a plasmid in some strains. The *ermD* gene was not directly linked to genes necessarily present on the plasmid (e.g. a replicon) and it is therefore likely the PCR product amplified was due to the presence of chromosomal DNA in their samples. We reanalyzed the same eight strains and found they contain *ermD* variants 1.2.1 or 2. The *ermD* gene was present in the chromosome in all eight strains, at the same position and context as in other strains with the same variants; thus there is no evidence for a plasmid-borne *ermD* in these strains (NCBI Biosample no. SAMN10470865-72).

Based on the findings in the present study and published results describing the *ermD* class of genes, it is most likely that *ermD* is present on the chromosome in evolutionary clades of *B*. *licheniformis*, *B*. *paralicheniformis* and closely related *Bacillus* species, but does not occur in other bacteria nor is it associated with any mobile genetic elements. The absence of *ermD* in many *B*. *licheniformis* isolates is proposed to be caused by independent deletion events in these lineages rather than site specific acquisitions in the lineages that bear *ermD*.

In 2002, the EU Scientific Committee on Animal Health issued an opinion which considered the use of a *B*. *licheniformis* strain possessing the *ermD* gene (NCTC 13123; AlCareTM) as a feed additive unsafe because of the risk of dissemination of genes that confer resistance to the MLS group of antibiotics via the food chain [58]. Even though our results do not support the reasoning underlying this opinion, *B*. *licheniformis* and *B*. *paralicheniformis* with resistance to macrolides should still be screened for the presence of resistance genes as these strains could potentially contain other classes of *erm* genes present on mobile genetic elements in addition to the *ermD* gene harbored on the chromosome [57].

An earlier study suggested that erythromycin resistance in *B*. *licheniformis* strains correlates (almost perfectly) with the capacity to produce the antibiotic bacitracin, a non-ribosomally synthesized branched cyclic dodecapeptide [59]. Bacitracin is synthesized by a large multienzyme complex composed of the three bacitracin synthetases BA1, BA2 and BA3, encoded by genes *bacA-bacC* [60]. We analyzed the 101 *B*. *licheniformis and B*. *paralicheniformis* strains and the three type strains for the presence of these bacitracin biosynthesis genes. The complete 10-gene bacitracin cluster, encoding enzymes, transporter and two-component regulatory system, is found in all *B*. *paralicheniformis* strains and also in the outlier strain CHCC20375, but in none of the *B*. *licheniformis* strains (S3 Table). Since many *B*. *licheniformis* strains also have an erythromycin resistance gene *ermD*, we find that this resistance trait does not correlate with bacitracin production in this species.

All genomes were analyzed with the tool HGtector [37] which uses statistical criteria to search for genes which are likely to have been acquired by horizontal gene transfer. Most genome were found to have between 5 and 50 genes which fulfill the criteria (S5 Table). Most of the identified genes encode proteins which are typically associated with mobile elements, e.g. phage proteins, RM systems, integrases/recombinases, helicases, etc (S5 Table). HGtector did not identify any of the putative *cat*, *aph*, *aadK* and *ermD* genes in *B*. *licheniformis* and *B*. *paralicheniformis* as potentially acquired by horizontal gene transfer.

EUCAST defines a microorganism as ‘wild-type’ (or innocuous) for a species based on the absence of acquired resistance to a drug in question; a microorganism is categorized as ‘wild-type’ by applying the appropriate cut-off value in a defined phenotypic test system [61]. The phenotype and genetic background for resistance to chloramphenicol and streptomycin does not support the MIC epidemiological cutoff (ECOFF) values for interpretation of chloramphenicol (8 mg/L), or streptomycin (8mg/L) susceptibility defined at the genus level by EFSA. ECOFFs are in the middle of the population distribution for both *B*. *licheniformis* and *B*. *paralicheniformis* (S1 Table). This supports revisiting the interpretation criteria and defining ECOFF values at the species level, as suggested in the previous study on MIC distribution of five *Bacillus* species [20]. Moreover, *B*. *paralicheniformis* should be considered intrinsically resistant to erythromycin so testing for erythromycin resistance in this species is not relevant.

## Conclusions

Environmental microorganisms generally live in complex ecosystems, and to survive they have developed numerous defense systems. Biosynthetic pathways for natural antibiotics are ancient, and numerous mechanisms for antibiotic resistance and tolerance have evolved over the past millennia [2, 5, 62, 63, 64].

The comparison of the complete genome sequences of more than 100 *B*. *licheniformis* and *B*. *paralicheniformis* strains, based on whole-genome phylogenetic tree analysis as previously described [19], showed that these species have separated through Darwinian evolution into two phylogenetically distinct clades. Only one strain was identified outside these clades, which presumably are very ancient.

Our analysis of the putative *cat*, *aph*, *aadK* and the *ermD* genes in *B*. *licheniformis* and *B*. *paralicheniformis* strongly suggests that these genes, present in the chromosome of the two species, are intrinsic and can be considered part of the ancient resistome, as the variation in the genes follows the phylogenetic relationships of the strains. Moreover, the genes are only very distantly related to the *cat*, *aph*, *aad* and *erm* genes present on mobile genetic elements or in other bacteria.

We conclude that the putative *cat*, *aph*, *aadK* and *ermD* may cause resistance or reduced susceptibility to specific antibiotics in the commercially important bacterial species *B*. *licheniformis and B*. *paralicheniformis*, but that these genes are most likely not horizontally transferable to other bacteria and consequently, will not add to the pool of resistance genes that pose a threat to human or animal health. Whole genome sequencing and phylogenetic analysis combined with amino acid sequence analysis and phenotypic susceptibility testing are useful approaches for determining intrinsic resistance and studying evolution of resistance determinants.

## Supporting information

S1 Fig Minimum spanning tree of MLSTs. Generated with the “MLST for categorical data” template in the advanced cluster analysis module of BioNumerics 6.6 (Applied Maths, Biomerieux). Each node represents a sequence type with the type number next to the node. The size of the node is defined by the number of strains. Strains included in this study are coloured blue, the other strains (white) are taken from pubmlst.org/blicheniformis. Thick solid lines: sequence types differ in one allele; medium solid lines: sequence types differ in two alleles; thin solid lines: sequence types differ in three alleles; dashed lines: sequence types differ in four alleles. Sequence types that differ in more than four alleles are not connected. Partitions (grey area) are built from sequence types that differ in two alleles and less. The cluster on the left is *B*. *licheniformis*, that on the right is *B*. *paralicheniformis*.
(TIF)

S2 Fig Whole genome phylogenetic tree with 3 outgroup strains. Whole genome phylogenetic tree reconstructed from the amino acid differences in the proteome of the core genomes of all 104 *B*. *licheniformis*, and *B*. *paralicheniformis* isolates and the outgroup strains *Bacillus subtilis* 168, *Bacillus cereus* CHCC20329 and *Bacillus sonorensis* CHCC20335. The tree was made based on approximate maximum likelihood and constructed using FastTree [35]. Clade A corresponds to *B*. *paralicheniformis* and clade B to *B*. *licheniformis*. Number in parentheses: Multi locus sequence types.
(PPTX)

S3 Fig Multiple sequence alignment of all CAT proteins. Sequence alignments were made with Clustal Omega using default settings. The left column indicates the locus tag of each *cat* gene per strain. Identical amino acids are indicated by an asterisk below each column. Residue numbers are indicated at the end of each row.
(DOCX)

S4 Fig Sequence comparison of the *B*. *licheniformis* CAT proteins with representatives of the previously defined 16 groups [39]. Cat proteins were aligned using Muscle [33, 34] and the phylogenetic tree (approximate maximum likelihood) was constructed using FastTree [35].
(PPTX)

S5 Fig Chromosomal region surrounding *cat* genes. The chromosomal region surrounding the *cat* gene (red border) in strains *B*. *paralicheniformis* 9945A and *B*. *licheniformis* DSM13 is shown. The figure was made with MGcV [31]. In the top section genes are color-coded according to functional category, and in the bottom section according to G+C% (numbers above selected genes indicate the G+C%). See [31] for color legends. Variable regions are indicated between dashed blue lines.
(PPTX)

S6 Fig Multiple sequence alignment of all AadK proteins. Sequence alignments were made with Clustal 2.1 using default settings. The left column indicates the locus tag of each *aadK* gene per strain. Identical amino acids are indicated by an asterisk below each column. Residue numbers are indicated at the end of each row.
(DOCX)

S7 Fig Multiple sequence alignment of all APH proteins. Sequence alignments were made with Clustal Omega using default settings. The left column indicates the locus tag of each *aph* gene per strain. Identical amino acids are indicated by an asterisk below each column. Residue numbers are indicated at the end of each row.
(DOCX)

S8 Fig Chromosomal region surrounding *aadK and aph* genes. The chromosomal region surrounding the *aadK* (red border) and *aph* genes in strains *B*. *paralicheniformis* 9945A and *B*. *licheniformis* DSM13 is shown. The figure was made with MGcV [31]. In the top section genes are color-coded according to functional category, and in the bottom section according to G+C% (numbers above selected genes indicate the G+C%). See [31] for color legends. Variable regions are indicated between dashed blue lines.
(PPTX)

S9 Fig Multiple sequence alignment of all ErmD proteins. Sequence alignments were made with Clustal Omega using default settings. The left column indicates the locus tag of each *ermD* gene per strain. Identical amino acids are indicated by an asterisk below each column. Residue numbers are indicated at the end of each row.
(DOCX)

S10 Fig Chromosomal region surrounding *ermD* genes. The chromosomal region surrounding the *ermD* gene in strains *B*. *paralicheniformis* 9945A and *B*. *licheniformis* DSM13 is shown. The figure was made with MGcV [31]. In the top section genes are color-coded according to functional category, and in the bottom section according to GC% (numbers above selected genes indicate the GC%). See [31] for color legends. Variable regions are indicated between dashed blue lines.
(PPTX)

S11 Fig Nucleotide alignment of representative *ermD* variants including the upstream regions. Sequence alignments were made with Clustal 2.1 using default settings. Two examples of each *ermD* sequence variant are shown, including about 400 upstream nucleotides (upstream numbering is shown at the left). Identical nucleotides are indicated by an asterisk below each column. The start codon of the *ermD* gene is shown in green. The two putative encoded leader peptides LP1 and LP2 are shown above the nucleotide sequences.
(DOCX)

S1 Table List of bacterial strains. Minimal inhibitory concentrations (MICs) are given in mg/L, ANI are given as ANIb values (AMIm gave a similar result). Footnotes: a, the year given is either the documented year of isolation or the earliest documented year of availability (e.g. year of deposition) indicated by <; b, listed as ST1 in the MLST database, but our analysis with sequence Genbank no. CP005965 gave ST4; c, strains listed in DSMZ catalogue as *Bacillus licheniformis* and *Bacillus* sp., respectively, *indicates novel Multilocus sequence types (STs) found in this study.
(XLSX)

S2 Table Genome sequencing statistics. The sequencing statistics are shown for the 101 sequenced *B*. *paralicheniformis/licheniformis* strains, *B*. *cereus strain* DSM 7459 and *B*. *sonorensis* strain DSM 12369.
(XLSX)

S3 Table Selected unique gene clusters in clades of *B*. *licheniformis* and *B*. *paralicheniformis*. Clade A strains have unique gene clusters for lantipeptide biosynthesis, urea utilization, bacitracin biosynthesis, respiratory nitrate reductase, and non-ribosomal peptide biosynthesis. Clade B strains have unique gene clusters for lichenicidin biosynthesis and a prophage. For each gene, information is given for contig number, locus tag, start and stop nucleotide on the contig, and size of encoded protein in amino acids. Ortholog group (OG) numbers are given in column A. Color codes: light blue = pseudogene; yellow = not clear which gene fragment in assembly belongs in the cluster. Column DD shows the manually curated annotation.
(XLSX)

S4 Table Genomic regions surrounding antibiotic resistance genes. Separate worksheets show the order of genes surrounding the antibiotic resistance genes *cat*, *aadK* and *aph*, and *ermD*. For each gene information is given for contig number, locus tag, start and stop nucleotide on the contig, and size of encoded protein in amino acids. Ortholog group (OG) numbers are given in column A. Color codes: light blue = pseudogene; green = OG with antibiotic resistance gene. Column DD shows the manually curated annotation.
(XLSX)

S5 Table Detection of putative horizontally acquired genes. For each gene, information is given for contig number, locus tag, start and stop nucleotide on the contig, and size of encoded protein in amino acids. Ortholog group (OG) numbers are given in column A. Color code orange indicates putative HGT genes as predicted by HGTector [37]. Column B shows the manually curated annotation. Row 3 shows the total number of putative HGT genes per strain.
(XLSX)
