# Supplementary figures and images for: Putative antibiotic resistance genes present in extant Bacillus licheniformis and Bacillus paralicheniformis strains are probably intrinsic and part of the ancient resistome
Source: PLoS One. 2019 Jan 15;14(1):e0210363. doi: 10.1371/journal.pone.0210363 (PMC6333372; doi:10.1371/journal.pone.0210363)

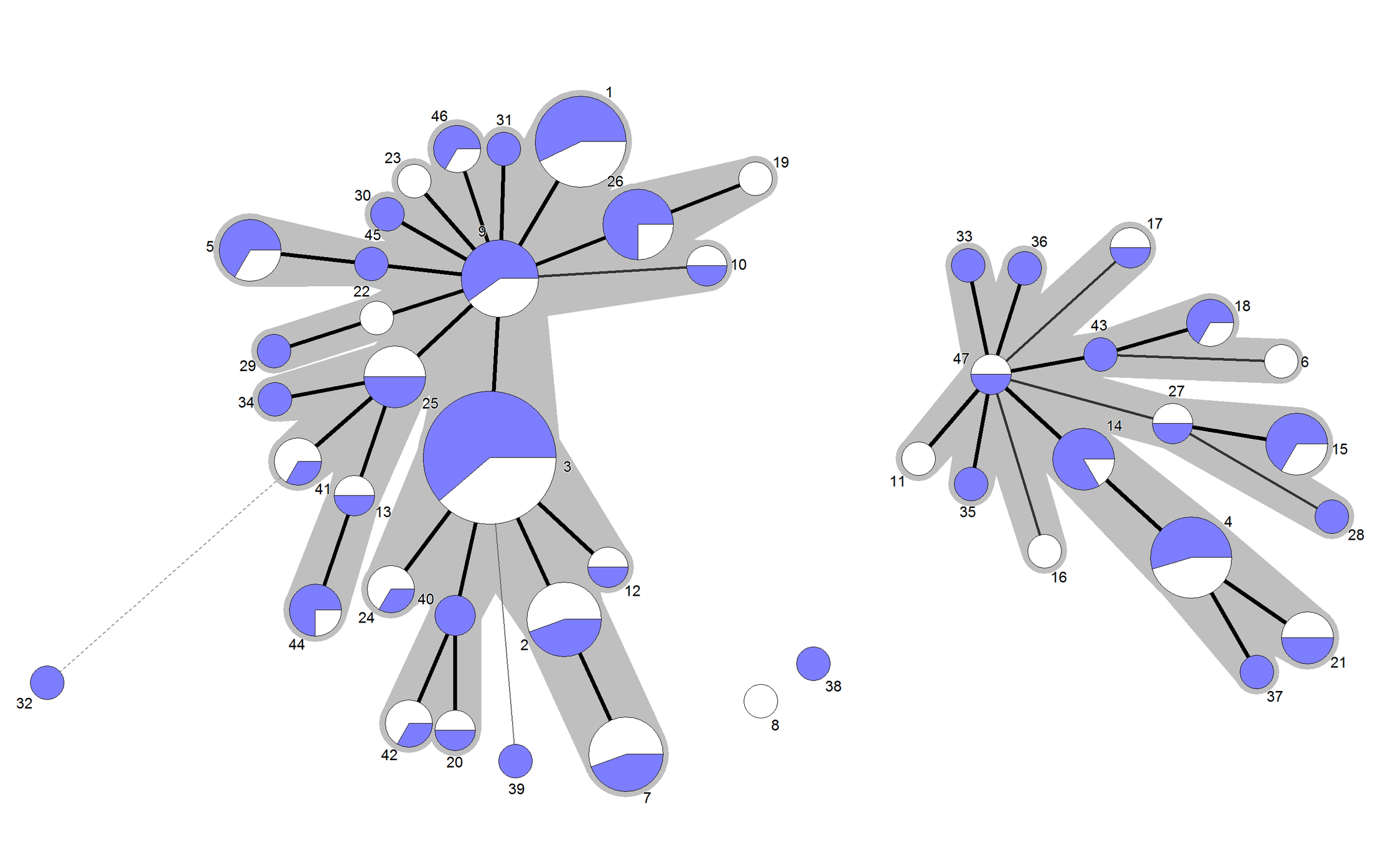

Supplement: S1 Fig — Generated with the “MLST for categorical data” template in the advanced cluster analysis module of BioNumerics 6.6 (Applied Maths, Biomerieux). Each node represents a sequence type with the type number next to the node. The size of the node is defined by the number of strains. Strains included in this study are coloured blue, the other strains (white) are taken from pubmlst.org/blicheniformis. Thick solid lines: sequence types differ in one allele; medium solid lines: sequence types differ in two alleles; thin solid lines: sequence types differ in three alleles; dashed lines: sequence types differ in four alleles. Sequence types that differ in more than four alleles are not connected. Partitions (grey area) are built from sequence types that differ in two alleles and less. The cluster on the left is B. licheniformis, that on the right is B. paralicheniformis. (TIF) [file pone.0210363.s001.tif]

## Slide 1
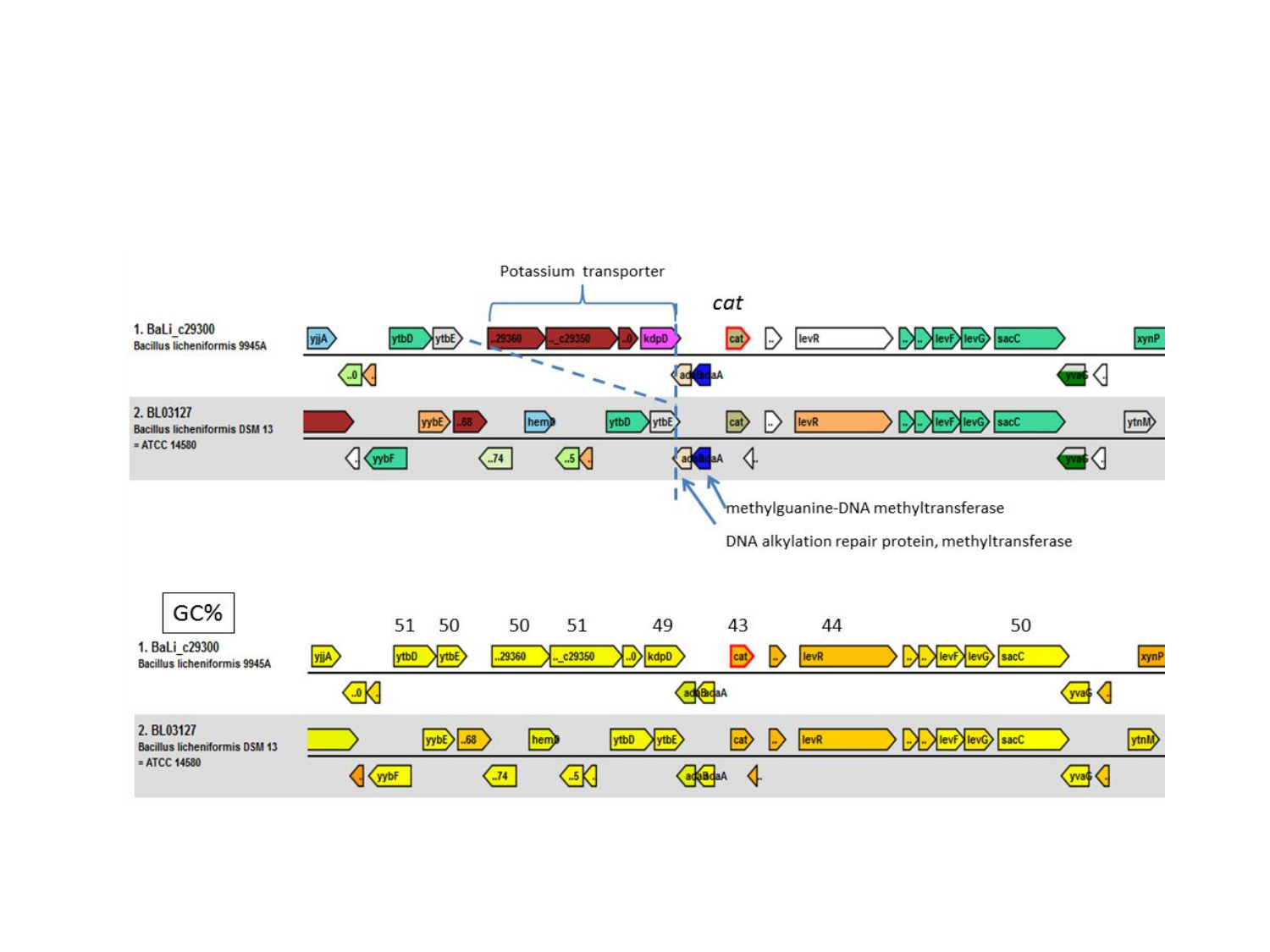

#

Supplement: S5 Fig — The chromosomal region surrounding the cat gene (red border) in strains B. paralicheniformis 9945A and B. licheniformis DSM13 is shown. The figure was made with MGcV [31]. In the top section genes are color-coded according to functional category, and in the bottom section according to G+C% (numbers above selected genes indicate the G+C%). See [31] for color legends. Variable regions are indicated between dashed blue lines. (PPTX) [file pone.0210363.s005.pptx]

## Slide 1
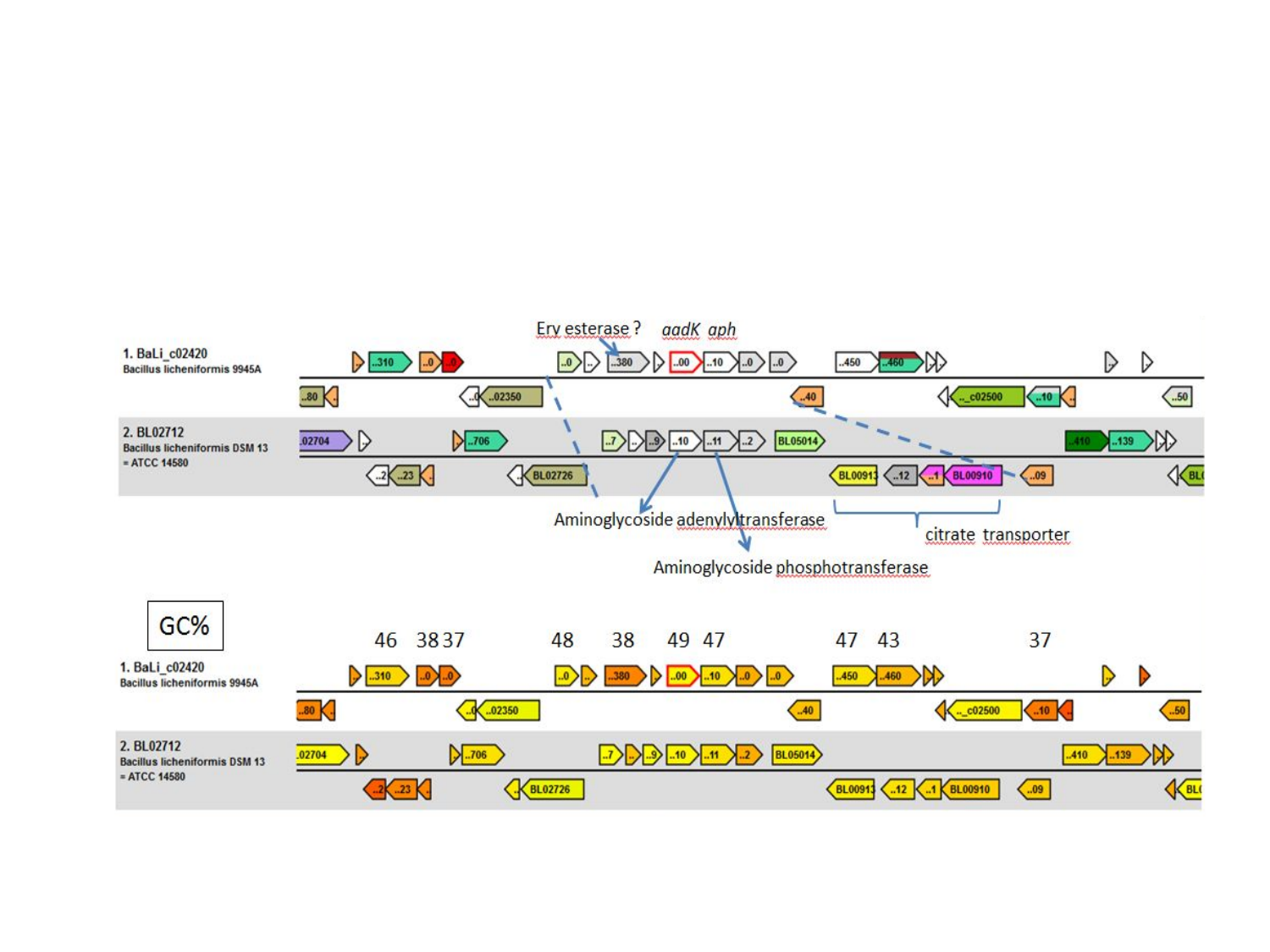

Supplement: S8 Fig — The chromosomal region surrounding the aadK (red border) and aph genes in strains B. paralicheniformis 9945A and B. licheniformis DSM13 is shown. The figure was made with MGcV [31]. In the top section genes are color-coded according to functional category, and in the bottom section according to G+C% (numbers above selected genes indicate the G+C%). See [31] for color legends. Variable regions are indicated between dashed blue lines. (PPTX) [file pone.0210363.s008.pptx]

## Slide 1
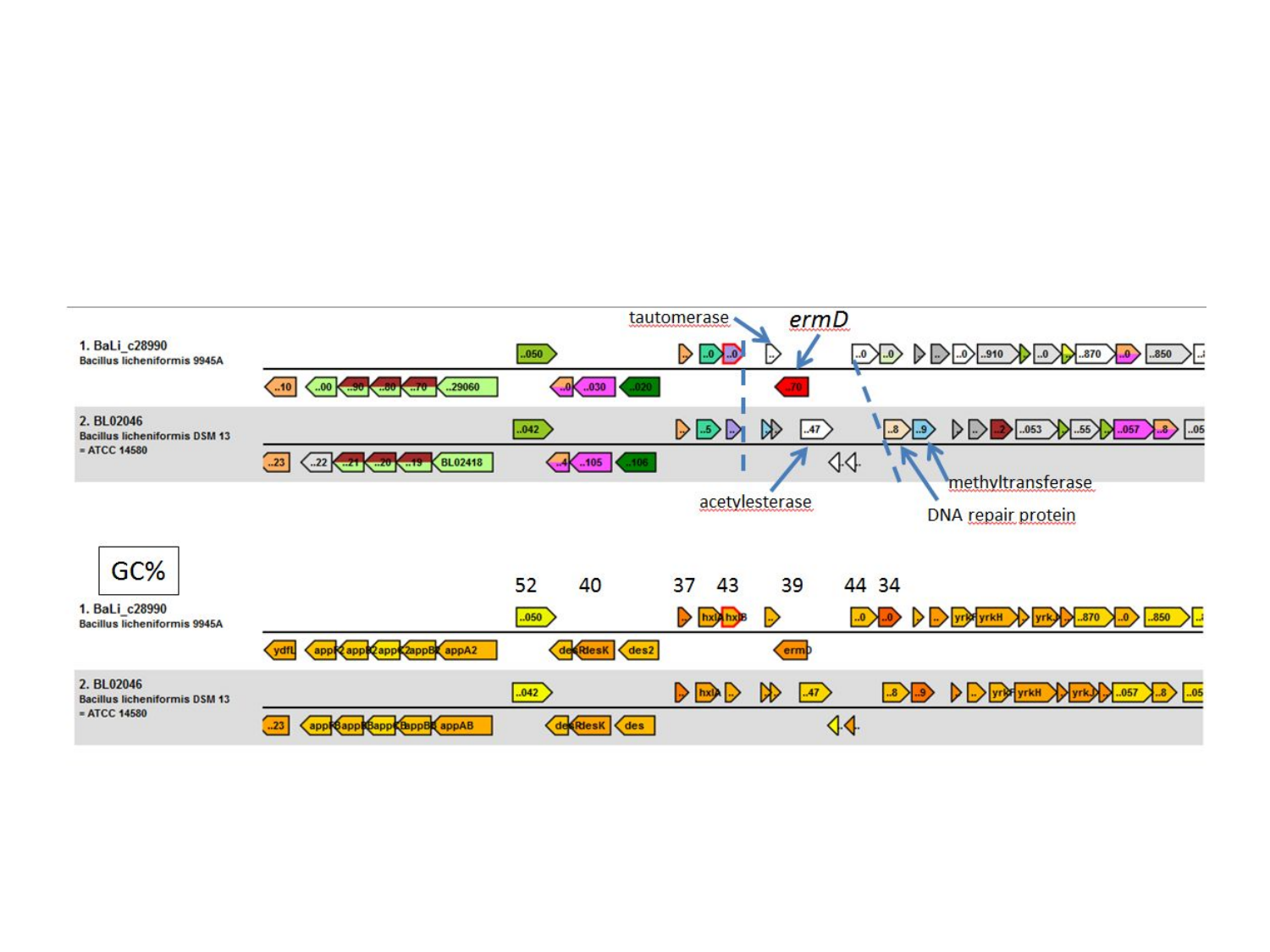

Supplement: S10 Fig — The chromosomal region surrounding the ermD gene in strains B. paralicheniformis 9945A and B. licheniformis DSM13 is shown. The figure was made with MGcV [31]. In the top section genes are color-coded according to functional category, and in the bottom section according to GC% (numbers above selected genes indicate the GC%). See [31] for color legends. Variable regions are indicated between dashed blue lines. (PPTX) [file pone.0210363.s010.pptx]
