# Supplementary material for: Putative antibiotic resistance genes present in extant Bacillus licheniformis and Bacillus paralicheniformis strains are probably intrinsic and part of the ancient resistome
Source: PLoS One. 2019 Jan 15;14(1):e0210363. doi: 10.1371/journal.pone.0210363 (PMC6333372; doi:10.1371/journal.pone.0210363)
Supplement: S2 Fig — Whole genome phylogenetic tree reconstructed from the amino acid differences in the proteome of the core genomes of all 104 B. licheniformis, and B. paralicheniformis isolates and the outgroup strains Bacillus subtilis 168, Bacillus cereus CHCC20329 and Bacillus sonorensis CHCC20335. The tree was made based on approximate maximum likelihood and constructed using FastTree [35]. Clade A corresponds to B. paralicheniformis and clade B to B. licheniformis. Number in parentheses: Multi locus sequence types. (PPTX) [file pone.0210363.s002.pptx]

## Slide 1
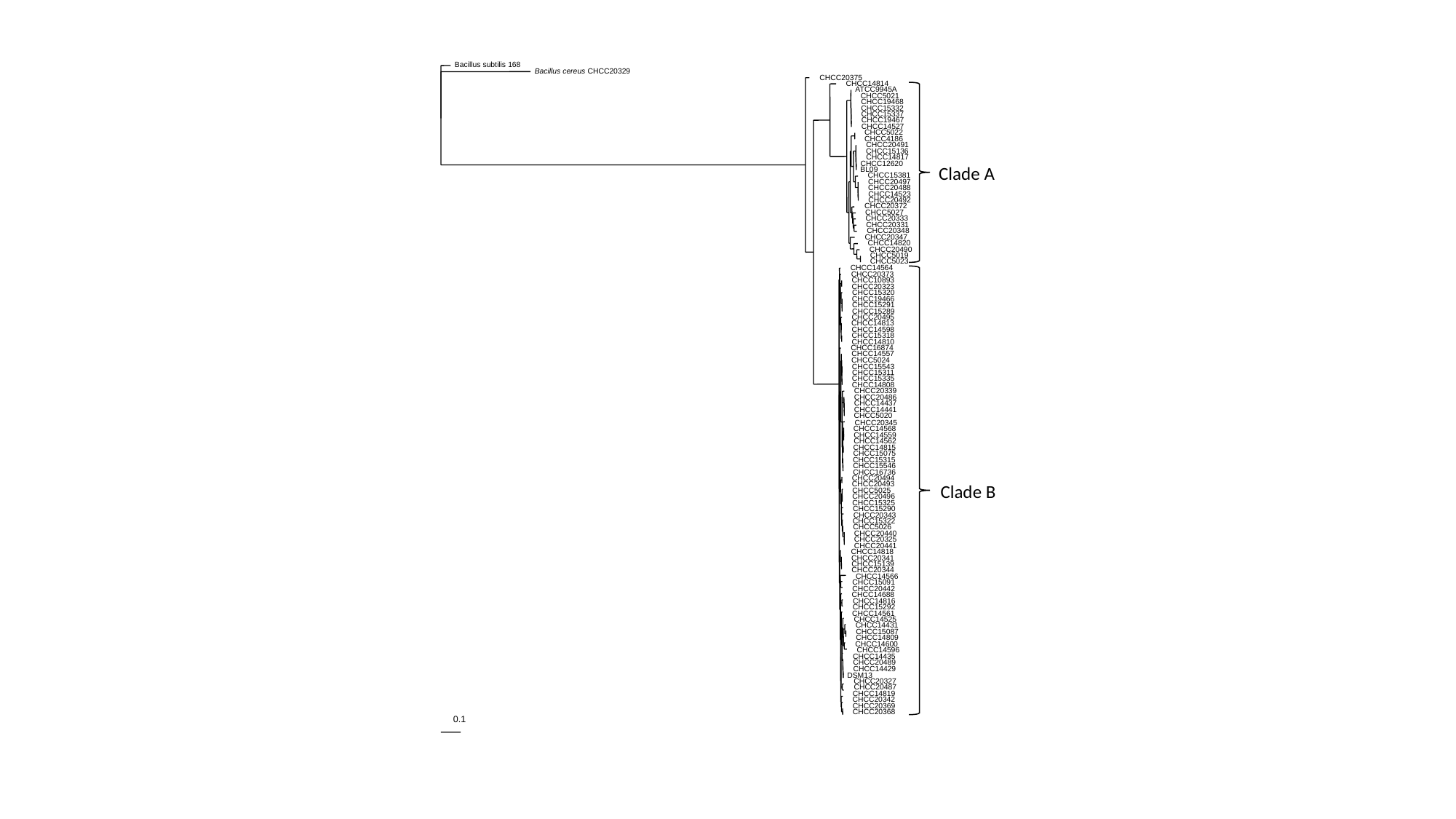

Bacillus subtilis 168
Bacillus cereus CHCC20329
CHCC20375
CHCC14814
ATCC9945A
CHCC5021
CHCC19468
CHCC15332
CHCC15337
CHCC19467
CHCC14527
CHCC5022
CHCC4186
CHCC20491
CHCC15136
CHCC14817
Clade A
CHCC12620
BL09
CHCC15381
CHCC20497
CHCC20488
CHCC14523
CHCC20492
CHCC20372
CHCC5027
CHCC20333
CHCC20331
CHCC20348
CHCC20347
CHCC14820
CHCC20490
CHCC5019
CHCC5023
CHCC14564
CHCC20373
CHCC10893
CHCC20323
CHCC15320
CHCC19466
CHCC15291
CHCC15289
CHCC20495
CHCC14813
CHCC14598
CHCC15318
CHCC14810
CHCC16874
CHCC14557
CHCC5024
CHCC15543
CHCC15311
CHCC15335
CHCC14808
CHCC20339
CHCC20486
CHCC14437
CHCC14441
CHCC5020
CHCC20345
CHCC14568
CHCC14559
CHCC14562
CHCC14815
CHCC15075
CHCC15315
CHCC15546
CHCC16736
CHCC20494
Clade B
CHCC20493
CHCC5025
CHCC20496
CHCC15325
CHCC15290
CHCC20343
CHCC15322
CHCC5026
CHCC20440
CHCC20325
CHCC20441
CHCC14818
CHCC20341
CHCC15139
CHCC20344
CHCC14566
CHCC15091
CHCC20442
CHCC14688
CHCC14816
CHCC15292
CHCC14561
CHCC14525
CHCC14431
CHCC15087
CHCC14809
CHCC14600
CHCC14596
CHCC14435
CHCC20489
CHCC14429
DSM13
CHCC20327
CHCC20487
CHCC14819
CHCC20342
CHCC20369
CHCC20368
0.1
