# Supplementary material for: Putative antibiotic resistance genes present in extant Bacillus licheniformis and Bacillus paralicheniformis strains are probably intrinsic and part of the ancient resistome
Source: PLoS One. 2019 Jan 15;14(1):e0210363. doi: 10.1371/journal.pone.0210363 (PMC6333372; doi:10.1371/journal.pone.0210363)
Supplement: S3 Fig — Sequence alignments were made with Clustal Omega using default settings. The left column indicates the locus tag of each cat gene per strain. Identical amino acids are indicated by an asterisk below each column. Residue numbers are indicated at the end of each row. (DOCX) [file pone.0210363.s003.docx]

CHCC15139_3882 MNFQTIELDTWYRKSYFDHYMKDAKCSFSITANVKVTNLLALLKKKKIKLYPAFIYIVSR 60

CHCC20344_2494 MNFQTIELDTWYRKSYFDHYMKDAKCSFSITANVKVTNLLALLKKKKIKLYPAFIYIVSR 60

CHCC20341_2443 MNFQTIELDTWYRKSYFDHYMKDAKCSFSITANVKVTNLLALLKKKKIKLYPAFIYIVSR 60

CHCC14818_3402 MNFQTIELDTWYRKSYFDHYMKDAKCSFSITANVKVTNLLALLKKKKIKLYPAFIYIVSR 60

CHCC14819_0760 MNFQTIELDTWYRKSYFDHYMKDAKCSFSITANVKVTNLLALLKKKKIKLYPAFIYIVSR 60

CHCC15291_3055 MNFQTIELDTWYRKSYFDHYMKDAKCSFSITANVNVTNLLALLKKKKIKLYPAFIYIVSR 60

CHCC14566_4422 MNFQTIELDTWYRKSYFDHYMKDAKCSFSITANVNVTNLLALLKKKKIKLYPAFIYIVSR 60

CHCC20342_0667 MNFQTIELDTWYRKSYFDHYMKDAKCSFSITANVNVTNLLALLKKKKIKLYPAFIYIVSR 60

CHCC20369_4383 MNFQTIELDTWYRKSYFDHYMKDAKCSFSITANVNVTNLLALLKKKKIKLYPAFIYIVSR 60

CHCC20368_3469 MNFQTIELDTWYRKSYFDHYMKDAKCSFSITANVNVTNLLALLKKKKIKLYPAFIYIVSR 60

CHCC14598_4486 MNFQTIELDTWYRKSYFDHYMKDAKCSFSITANVNVTNLLALLKKKKIKLYPAFIYIVSR 60

CHCC15289_3447 MNFQTIELDTWYRKSYFDHYMKDAKCSFSITANVNVTNLLALLKKKKIKLYPAFIYIVSR 60

CHCC20495_3293 MNFQTIELDTWYRKSYFDHYMKDAKCSFSITANVNVTNLLALLKKKKIKLYPAFIYIVSR 60

CHCC14810_3357 MNFQTIELDTWYRKSYFDHYMKDAKCSFSITANVNVTNLLALLKKKKIKLYPAFIYIVSR 60

CHCC20373_3167 MNFQTIELDTWYRKSYFDHYMKDAKCSFSITANVNVTNLLALLKKKKIKLYPAFIYIVSR 60

CHCC14564_0420 MNFQTIELDTWYRKSYFDHYMKDAKCSFSITANVNVTNLLALLKKKKIKLYPAFIYIVSR 60

CHCC14813_2931 MNFQTIELDTWYRKSYFDHYMKDAKCSFSITANVNVTNLLALLKKKKIKLYPAFIYIVSR 60

CHCC20442_0193 MNFQTIELDTWYRKSYFDHYMKDAKCSFSITANVNVTNLLALLKKKKIKLYPAFIYIVSR 60

CHCC14809_0373 MNFQTIELDTWYRKSYFDHYMKDAKCSFSITANVNVTNLLALLKKKKIKLYPAFIYIVSR 60

CHCC15320_1343 MNFQTIELDTWYRKSYFDHYMKDAKCSFSITANVNVTNLLALLKKKKIKLYPAFIYIVSR 60

CHCC14525_1227 MNFQTIELDTWYRKSYFDHYMKDAKCSFSITANVNVTNLLALLKKKKIKLYPAFIYIVSR 60

CHCC15318_0524 MNFQTIELDTWYRKSYFDHYMKDAKCSFSITANVNVTNLLALLKKKKIKLYPAFIYIVSR 60

CHCC20323_0869 MNFQTIELDTWYRKSYFDHYMKDAKCSFSITANVNVTNLLALLKKKKIKLYPAFIYIVSR 60

CHCC15087_4076 MNFQTIELDTWYRKSYFDHYMKDAKCSFSITANVNVTNLLALLKKKKIKLYPAFIYIVSR 60

CHCC14431_1770 MNFQTIELDTWYRKSYFDHYMKDAKCSFSITANVNVTNLLALLKKKKIKLYPAFIYIVSR 60

CHCC15091_0059 MNFQTIELDTWYRKSYFDHYMKDAKCSFSITANVNVTNLLALLKKKKIKLYPAFIYIVSR 60

CHCC19466_0660 MNFQTIELDTWYRKSYFDHYMKDAKCSFSITANVNVTNLLALLKKKKIKLYPAFIYIVSR 60

CHCC10893_4304 MNFQTIELDTWYRKSYFDHYMKDAKCSFSITANVNVTNLLALLKKKKIKLYPAFIYIVSR 60

CHCC15546_2749 MNFQTIELDTWYRKSYFDHYMKEAKCSFSITANVNVTNLLALLKKKKIKLYPAFIYIVSR 60

CHCC15543_2412 MNFQTIELDTWYRKSYFDHYMKEAKCSFSITANVNVTNLLALLKKKKIKLYPAFIYIVSR 60

CHCC16736_1335 MNFQTIELDTWYRKSYFDHYMKEAKCSFSITANVNVTNLLALLKKKKIKLYPAFIYIVSR 60

CHCC14559_1158 MNFQTIELDTWYRKSYFDHYMKEAKCSFSITANVNVTNLLALLKKKKIKLYPAFIYIVSR 60

CHCC20325_0259 MNFQTIELDTWYRKSYFDHYMKEAKCSFSITANVNVTNLLALLKKKKIKLYPAFIYIVSR 60

CHCC15315_0117 MNFQTIELDTWYRKSYFDHYMKEAKCSFSITANVNVTNLLALLKKKKIKLYPAFIYIVSR 60

CHCC15311_4297 MNFQTIELDTWYRKSYFDHYMKEAKCSFSITANVNVTNLLALLKKKKIKLYPAFIYIVSR 60

CHCC14568_3889 MNFQTIELDTWYRKSYFDHYMKEAKCSFSITANVNVTNLLALLKKKKIKLYPAFIYIVSR 60

CHCC15322_2635 MNFQTIELDTWYRKSYFDHYMKEAKCSFSITANVNVTNLLALLKKKKIKLYPAFIYIVSR 60

CHCC20345_2951 MNFQTIELDTWYRKSYFDHYMKEAKCSFSITANVNVTNLLALLKKKKIKLYPAFIYIVSR 60

CHCC20343_0747 MNFQTIELDTWYRKSYFDHYMKEAKCSFSITANVNVTNLLALLKKKKIKLYPAFIYIVSR 60

CHCC15335_1117 MNFQTIELDTWYRKSYFDHYMKEAKCSFSITANVNVTNLLALLKKKKIKLYPAFIYIVSR 60

CHCC14808_0727 MNFQTIELDTWYRKSYFDHYMKEAKCSFSITANVNVTNLLALLKKKKIKLYPAFIYIVSR 60

CHCC5026_2084 MNFQTIELDTWYRKSYFDHYMKEAKCSFSITANVNVTNLLALLKKKKIKLYPAFIYIVSR 60

CHCC5024_1205 MNFQTIELDTWYRKSYFDHYMKEAKCSFSITANVNVTNLLALLKKKKIKLYPAFIYIVSR 60

CHCC20440_0720 MNFQTIELDTWYRKSYFDHYMKEAKCSFSITANVNVTNLLALLKKKKIKLYPAFIYIVSR 60

CHCC20441_4480 MNFQTIELDTWYRKSYFDHYMKEAKCSFSITANVNVTNLLALLKKKKIKLYPAFIYIVSR 60

CHCC14815_4043 MNFQTIELDTWYRKSYFDHYMKEAKCSFSITANVNVTNLLALLKKKKIKLYPAFIYIVSR 60

CHCC15290_0718 MNFQTIELDTWYRKSYFDHYMKEAKCSFSITANVNVTNLLALLKKKKIKLYPAFIYIVSR 60

CHCC14562_3973 MNFQTIELDTWYRKSYFDHYMKEAKCSFSITANVNVTNLLALLKKKKIKLYPAFIYIVSR 60

CHCC15075_2813 MNFQTIELDTWYRKSYFDHYMKEAKCSFSITANVNVTNLLALLKKKKIKLYPAFIYIVSR 60

CHCC15381_0137 MNFQTIDLDTWYRKSYFDHYMKEAKCSFSITTNVNVTNLLAVLKKKKIKLYPVFVYIVSR 60

CHCC20372_3730 MNFQTIDLDTWYRKSYFDHYMKEAKCSFSITTNVNVTNLLAVLKKKKIKLYPVFVYIVSR 60

CHCC19467_2131 MNFQTIDLDTWYRKSYFDHYMKEAKCSFSITTNVNVTNLLAVLKKKKIKLYPVFIYIVSR 60

CHCC19468_0817 MNFQTIDLDTWYRKSYFDHYMKEAKCSFSITTNVNVTNLLAVLKKKKIKLYPVFIYIVSR 60

CHCC20497_3599 MNFQTIDLDTWYRKSYFDHYMKEAKCSFSITTNVNVTNLLAVLKKKKIKLYPVFIYIVSR 60

CHCC20492_1406 MNFQTIDLDTWYRKSYFDHYMKEAKCSFSITTNVNVTNLLAVLKKKKIKLYPVFIYIVSR 60

CHCC14523_2493 MNFQTIDLDTWYRKSYFDHYMKEAKCSFSITTNVNVTNLLAVLKKKKIKLYPVFIYIVSR 60

CHCC14527_1718 MNFQTIDLDTWYRKSYFDHYMKEAKCSFSITTNVNVTNLLAVLKKKKIKLYPVFIYIVSR 60

CHCC20333_1346 MNFQTIDLDTWYRKSYFDHYMKEAKCSFSITTNVNVTNLLAVLKKKKIKLYPVFIYIVSR 60

ATCC9945A_3028 MNFQTIDLDTWYRKSYFDHYMKEAKCSFSITTNVNVTNLLAVLKKKKIKLYPVFIYIVSR 60

CHCC20347_3632 MNFQTIDLDTWYRKSYFDHYMKEAKCSFSITTNVNVTNLLAVLKKKKIKLYPVFIYIVSR 60

CHCC15337_3824 MNFQTIDLDTWYRKSYFDHYMKEAKCSFSITTNVNVTNLLAVLKKKKIKLYPVFIYIVSR 60

CHCC15332_2367 MNFQTIDLDTWYRKSYFDHYMKEAKCSFSITTNVNVTNLLAVLKKKKIKLYPVFIYIVSR 60

CHCC5022_4011 MNFQTIDLDTWYRKSYFDHYMKEAKCSFSITTNVNVTNLLAVLKKKKIKLYPVFIYIVSR 60

CHCC5021_0537 MNFQTIDLDTWYRKSYFDHYMKEAKCSFSITTNVNVTNLLAVLKKKKIKLYPVFIYIVSR 60

CHCC5027_4207 MNFQTIDLDTWYRKSYFDHYMKEAKCSFSITTNVNVTNLLAVLKKKKIKLYPVFIYIVSR 60

CHCC14820_3541 MNFQTIDLDTWYRKSYFDHYMKEAKCSFSITTNVNVTNLLAVLKKKKIKLYPVFIYIVSR 60

CHCC4186_3571 MNFQTIDLDTWYRKSYFDHYMKEAKCSFSITTNVNVTNLLAVLKKKKIKLYPVFIYIVSR 60

CHCC20488_2389 MNFQTIDLDTWYRKSYFDHYMKEAKCSFSITTNVNVTNLLAVLKKKKIKLYPVFIYIVSR 60

CHCC20331_0965 MNFQTIDLDTWYRKSYFDHYMKEAKCSFSITANVNVTNLFAVLKKKKIKLYPVFIYIVSR 60

CHCC20348_2795 MNFQTIDLDTWYRKSYFDHYMKEAKCSFSITANVNVTNLFAVLKKKKIKLYPVFIYIVSR 60

CHCC12620_12620 MNFQTIDLDTWYRKSYFDHYMKEAKCSFSITANVNVTNLLAVLKKKKIKLYPVFIYIVSR 60

CHCC20491_1156 MNFQTIDLDTWYRKSYFDHYMKEAKCSFSITANVNVTNLLAVLKKKKIKLYPVFIYIVSR 60

CHCC15136_3048 MNFQTIDLDTWYRKSYFDHYMKEAKCSFSITANVNVTNLLAVLKKKKIKLYPVFIYIVSR 60

CHCC14817_2262 MNFQTIDLDTWYRKSYFDHYMKEAKCSFSITANVNVTNLLAVLKKKKIKLYPVFIYIVSR 60

ATCCBL09_3082 MNFQTIDLDTWYRKSYFDHYMKEAKCSFSITANVNVTNLLAVLKKKKIKLYPVFIYIVSR 60

CHCC20490_4151 MNFQTIDLDTWYRKSYFDHYMKEAKCSFSITANVNVTNLLAVLKKKKIKLYPVFIYIVSR 60

CHCC5019_3909 MNFQTIDLDTWYRKSYFDHYMKEAKCSFSITANVNVTNLLAVLKKKKIKLYPVFIYIVSR 60

CHCC5023_4401 MNFQTIDLDTWYRKSYFDHYMKEAKCSFSITANVNVTNLLAVLKKKKIKLYPVFIYIVSR 60

CHCC14814_4408 MNFQTIKLDTWYRKPYFDHYMKEAKCSFSITANVNVTNLLAMLKKKKLKLYPAFIYIVSK 60

CHCC20375_2721 MNFQIIELDTWYRKSYFDHYMKEAKCSFSITENVNVTNLLAVLKKKKIKLYPAFIYIVSR 60

CHCC20494_3859 MNFQTIELDTWYRKSYFDHYMKEAKCSFSITANVNVTNLLAVLKKKKLKLYPAFIYIVSR 60

CHCC20493_4099 MNFQTIELDTWYRKSYFDHYMKEAKCSFSITANVNVTNLLAVLKKKKLKLYPAFIYIVSR 60

CHCC14557_2557 MNFQTIELDTWYRKSYFDHYMKEAKCSFSITANVNVKNLLAVLKKKKLKLYPAFIYIVSR 60

CHCC16874_3846 MNFQTIELDTWYRKSYFDHYMKEAKCSFSITANVNVTNLLAVLKKKKLKLYPAFIYIVSR 60

CHCC20496_2682 MNFQTIELDTWYRKSYFDHYMKEAKCSFSITANVNVTNLLAVLKKKKLKLYPAFIYIVSR 60

CHCC20339_2019 MNFQTIELDTWYRKSYFDHYMKEAKCSFSITANVNVTNLLAVLKKKKLKLYPAFIYIVSR 60

CHCC15325_3739 MNFQTIELDTWYRKSYFDHYMKEAKCSFSITANVNVTNLLAVLKKKKLKLYPAFIYIVSR 60

CHCC5025_4628 MNFQTIELDTWYRKSYFDHYMKEAKCSFSITANVNVTNLLAVLKKKKLKLYPAFIYIVSR 60

CHCC14429_1025 MNFQTIELDTWYRKSYFDHYMKEAKCSFSITANVNVTNLLAVLKKKKLKLYPAFIYIVSR 60

CHCC14435_1076 MNFQTIELDTWYRKSYFDHYMKEAKCSFSITANVNVTNLLAVLKKKKLKLYPAFIYIVSR 60

CHCC14437_3617 MNFQTIELDTWYRKSYFDHYMKEAKCSFSITANVNVTNLLAVLKKKKLKLYPAFIYIVSR 60

CHCC20327_4458 MNFQTIELDTWYRKSYFDHYMKEAKCSFSITANVNVTNLLAVLKKKKLKLYPAFIYIVSR 60

CHCC20489_3325 MNFQTIELDTWYRKSYFDHYMKEAKCSFSITANVNVTNLLAVLKKKKLKLYPAFIYIVSR 60

CHCC20486_3539 MNFQTIELDTWYRKSYFDHYMKEAKCSFSITANVNVTNLLAVLKKKKLKLYPAFIYIVSR 60

CHCC20487_0369 MNFQTIELDTWYRKSYFDHYMKEAKCSFSITANVNVTNLLAVLKKKKLKLYPAFIYIVSR 60

CHCC14441_2582 MNFQTIELDTWYRKSYFDHYMKEAKCSFSITANVNVTNLLAVLKKKKLKLYPAFIYIVSR 60

CHCC14688_4106 MNFQTIELDTWYRKSYFDHYMKEAKCSFSITANVNVTNLLAVLKKKKLKLYPAFIYIVSR 60

CHCC5020_0388 MNFQTIELDTWYRKSYFDHYMKEAKCSFSITANVNVTNLLAVLKKKKLKLYPAFIYIVSR 60

CHCC14816_2696 MNFQTIELDTWYRKSYFDHYMKEAKCSFSITANVNVTNLLAVLKKKKLKLYPAFIYIVSR 60

CHCC15292_1484 MNFQTIELDTWYRKSYFDHYMKEAKCSFSITANVNVTNLLAVLKKKKLKLYPAFIYIVSR 60

CHCC14561_0446 MNFQTIELDTWYRKSYFDHYMKEAKCSFSITANVNVTNLLAVLKKKKLKLYPAFIYIVSR 60

DSM13_2943 MNFQTIELDTWYRKSYFDHYMKEAKCSFSITANVNVTNLLAVLKKKKLKLYPAFIYIVSR 60

CHCC14600_2465 MNFQTIELDTWYRKSYFDHYMKEAKCSFSITANVNVTNLLAVLKKKKLKLYPAFIYIVSR 60

CHCC14596_4179 MNFQTIELDTWYRKSYFDHYMKEAKCSFSITANVNVTNLLAVLKKKKLKLYPAFIYIVSR 60

**** *.*******.*******:******** **:*.**:*:*****:****.*:****:

CHCC15139_3882 VIHSRPEFRTTFDDKGRLGYWEQMHPCYAIFHQDDQTFSALWTEYSDDFSQFYHQYLLDA 120

CHCC20344_2494 VIHSRPEFRTTFDDKGRLGYWEQMHPCYAIFHQDDQTFSALWTEYSDDFSQFYHQYLLDA 120

CHCC20341_2443 VIHSRPEFRTTFDDKGRLGYWEQMHPCYAIFHQDDQTFSALWTEYSDDFSQFYHQYLLDA 120

CHCC14818_3402 VIHSRPEFRTTFDDKGRLGYWEQMHPCYAIFHQDDQTFSALWTEYSDDFSQFYHQYLLDA 120

CHCC14819_0760 VIHSRPEFRTTFDDKGRLGYWEQMHPCYAIFHQDDQTFSALWTEYSDDFSQFYHQYLLDA 120

CHCC15291_3055 VIHSRPEFRTTFDDKGRLGYWEQMHPCYAIFHQDDQTFSALWTEYSDDFSQFYHQYLLDA 120

CHCC14566_4422 VIHSRPEFRTTFDDKGRLGYWEQMHPCYAIFHQDDQTFSALWTEYSDDFSQFYHQYLLDA 120

CHCC20342_0667 VIHSRPEFRTTFDDKGRLGYWEQMHPCYAIFHQDDQTFSALWTEYSDDFSQFYHQYLLDA 120

CHCC20369_4383 VIHSRPEFRTTFDDKGRLGYWEQMHPCYAIFHQDDQTFSALWTEYSDDFSQFYHQYLLDA 120

CHCC20368_3469 VIHSRPEFRTTFDDKGRLGYWEQMHPCYAIFHQDDQTFSALWTEYSDDFSQFYHQYLLDA 120

CHCC14598_4486 VIHSRPEFRTTFDDKGRLGYWEQMHPCYAIFHQDDQTFSALWTEYSDDFSQFYHQYLLDA 120

CHCC15289_3447 VIHSRPEFRTTFDDKGRLGYWEQMHPCYAIFHQDDQTFSALWTEYSDDFSQFYHQYLLDA 120

CHCC20495_3293 VIHSRPEFRTTFDDKGRLGYWEQMHPCYAIFHQDDQTYSALWTEYSDDFSQFYHQYLLDA 120

CHCC14810_3357 VIHSRPEFRTTFDDKGRLGYWEQMHPCYAIFHQDDQTFSALWTEYSDDFSQFYHQYLLDA 120

CHCC20373_3167 VIHSRPEFRTTFDDKGRLGYWEQMHPCYAIFHQDDQTFSALWTEYSDDFSQFYHQYLLDA 120

CHCC14564_0420 VIHSRPEFRTTFDDKGRLGYWEQMHPCYAIFHQDDQTFSALWTEYSDDFSQFYHQYLLDA 120

CHCC14813_2931 VIHSRPEFRTTFDDKGRLGYWEQMHPCYAIFHQDDQTFSALWTEYSDDFSQFYHQYLLDA 120

CHCC20442_0193 VIHSRPEFRTTFDDKGRLGYWEQMHPCYAIFHQDDQTFSALWTEYSDDFSQFYHQYLLDA 120

CHCC14809_0373 VIHSRPEFRTTFDDKGRLGYWEQMHPCYAIFHQDDQTFSALWTEYSDDFSQFYHQYLLDA 120

CHCC15320_1343 VIHSRPEFRTTFDDKGRLGYWEQMHPCYAIFHQDDQTFSALWTEYSDDFSQFYHQYLLDA 120

CHCC14525_1227 VIHSRPEFRTTFDDKGRLGYWEQMHPCYAIFHQDDQTFSALWTEYSDDFSQFYHQYLLDA 120

CHCC15318_0524 VIHSRPEFRTTFDDKGRLGYWEQMHPCYAIFHQDDQTFSALWTEYSDDFSQFYHQYLLDA 120

CHCC20323_0869 VIHSRPEFRTTFDDKGRLGYWEQMHPCYAIFHQDDQTFSALWTEYSDDFSQFYHQYLLDA 120

CHCC15087_4076 VIHSRPEFRTTFDDKGRLGYWEQMHPCYAIFHQDDQTFSALWTEYSDDFSQFYHQYLLDA 120

CHCC14431_1770 VIHSRPEFRTTFDDKGRLGYWEQMHPCYAIFHQDDQTFSALWTEYSDDFSQFYHQYLLDA 120

CHCC15091_0059 VIHSRPEFRTTFDDKGRLGYWEQMHPCYAIFHQDDQTFSALWTEYSDDFSQFYHQYLLDA 120

CHCC19466_0660 VIHSRPEFRTTFDDKGRLGYWEQMHPCYAIFHQDDQTFSALWTEYSDDFSQFYHQYLLDA 120

CHCC10893_4304 VIHSRPEFRTTFDDKGRLGYWEQMHPCYAIFHQDDQTFSALWTEYSDDFSQFYHQYLLDA 120

CHCC15546_2749 VIHSRPEFRTTFDDKGRLGYWEQMHPCYAIFHQDDQTFSALWTEYSDDFSQFYHQYLLDA 120

CHCC15543_2412 VIHSRPEFRTTFDDKGRLGYWEQMHPCYAIFHQDDQTFSALWTEYSDDFSQFYHQYLLDA 120

CHCC16736_1335 VIHSRPEFRTTFDDKGRLGYWEQMHPCYAIFHQDDQTFSALWTEYSDDFSQFYHQYLLDA 120

CHCC14559_1158 VIHSRPEFRTTFDDKGRLGYWEQMHPCYAIFHQDDQTFSALWTEYSDDFSQFYHQYLLDA 120

CHCC20325_0259 VIHSRPEFRTTFDDKGRLGYWEQMHPCYAIFHQDDQTFSALWTEYSDDFSQFYHQYLLDA 120

CHCC15315_0117 VIHSRPEFRTTFDDKGRLGYWEQMHPCYAIFHQDDQTFSALWTEYSDDFSQFYHQYLLDA 120

CHCC15311_4297 VIHSRPEFRTTFDDKGRLGYWEQMHPCYAIFHQDDQTFSALWTEYSDDFSQFYHQYLLDA 120

CHCC14568_3889 VIHSRPEFRTTFDDKGRLGYWEQMHPCYAIFHQDDQTFSALWTEYSDDFSQFYHQYLLDA 120

CHCC15322_2635 VIHSRPEFRTTFDDKGRLGYWEQMHPCYAIFHQDDQTFSALWTEYSDDFSQFYHQYLLDA 120

CHCC20345_2951 VIHSRPEFRTTFDDKGRLGYWEQMHPCYAIFHQDDQTFSALWTEYSDDFSQFYHQYLLDA 120

CHCC20343_0747 VIHSRPEFRTTFDDKGRLGYWEQMHPCYAIFHQDDQTFSALWTEYSDDFSQFYHQYLLDA 120

CHCC15335_1117 VIHSRPEFRTTFDDKGRLGYWEQMHPCYAIFHQDDQTFSALWTEYSDDFSQFYHQYLLDA 120

CHCC14808_0727 VIHSRPEFRTTFDDKGRLGYWEQMHPCYAIFHQDDQTFSALWTEYSDDFSQFYHQYLLDA 120

CHCC5026_2084 VIHSRPEFRTTFDDKGRLGYWEQMHPCYAIFHQDDQTFSALWTEYSDDFSQFYHQYLLDA 120

CHCC5024_1205 VIHSRPEFRTTFDDKGRLGYWEQMHPCYAIFHQDDQTFSALWTEYSDDFSQFYHQYLLDA 120

CHCC20440_0720 VIHSRPEFRTTFDDKGRLGYWEQMHPCYAIFHQDDQTFSALWTEYSDDFSQFYHQYLLDA 120

CHCC20441_4480 VIHSRPEFRTTFDDKGRLGYWEQMHPCYAIFHQDDQTFSALWTEYSDDFSQFYHQYLLDA 120

CHCC14815_4043 VIHSRPEFRTTFDDKGRLGYWEQMHPCYAIFHQDDQTFSALWTEYSDDFSQFYHQYLLDA 120

CHCC15290_0718 VIHSRPEFRTTFDDKGRLGYWEQMHPCYAIFHQDDQTFSALWTEYSDDFSQFYHQYLLDA 120

CHCC14562_3973 VIHSRPEFRTTFDDKGRLGYWEQMHPCYAIFHQDDQTFSALWTEYSDDFSQFYHQYLLDA 120

CHCC15075_2813 VIHSRPEFRTTFDDKGRLGYWEQMHPCYAIFHQDDQTFSALWTEYSDDFSQFYHQYLLDA 120

CHCC15381_0137 AIHSRPEFRTTFNDKGQLGYWEQMHPCYTIFHQDDQTFSALWTEYSDDFSRFYRQYLQDA 120

CHCC20372_3730 AIHSRPEFRTTFNDKGQLGYWEQMHPCYTIFHQDDQTFSALWTEYSDDFSRFYRQYLQDA 120

CHCC19467_2131 AIHSRPEFRTTFNDKGQLGYWEQMHPCYTIFHQDDQTFSALWTEYSNDFSRFYRQYLQDA 120

CHCC19468_0817 AIHSRPEFRTTFNDKGQLGYWEQMHPCYTIFHQDDQTFSALWTEYSNDFSRFYRQYLQDA 120

CHCC20497_3599 AIHSRPEFRTTFNDKGQLGYWEQMHPCYTIFHQDDQTFSALWTEYSNDFSRFYRQYLQDA 120

CHCC20492_1406 AIHSRPEFRTTFNDKGQLGYWEQMHPCYTIFHQDDQTFSALWTEYSNDFSRFYRQYLQDA 120

CHCC14523_2493 AIHSRPEFRTTFNDKGQLGYWEQMHPCYTIFHQDDQTFSALWTEYSNDFSRFYRQYLQDA 120

CHCC14527_1718 AIHSRPEFRTTFNDKGQLGYWEQMHPCYTIFHQDDQTFSALWTEYSNDFSRFYRQYLQDA 120

CHCC20333_1346 AIHSRPEFRTTFNDKGQLGYWEQMHPCYTIFHQDDQTFSALWTEYSNDFSRFYRQYLQDA 120

ATCC9945A_3028 AIHSRPEFRTTFNDKGQLGYWEQMHPCYTIFHQDDQTFSALWTEYSNDFSRFYRQYLQDA 120

CHCC20347_3632 AIHSRPEFRTTFNDKGQLGYWEQMHPCYTIFHQDDQTFSALWTEYSNDFSRFYRQYLQDA 120

CHCC15337_3824 AIHSRPEFRTTFNDKGQLGYWEQMHPCYTIFHQDDQTFSALWTEYSNDFSRFYRQYLQDA 120

CHCC15332_2367 AIHSRPEFRTTFNDKGQLGYWEQMHPCYTIFHQDDQTFSALWTEYSNDFSRFYRQYLQDA 120

CHCC5022_4011 AIHSRPEFRTTFNDKGQLGYWEQMHPCYTIFHQDDQTFSALWTEYSNDFSRFYRQYLQDA 120

CHCC5021_0537 AIHSRPEFRTTFNDKGQLGYWEQMHPCYTIFHQDDQTFSALWTEYSNDFSRFYRQYLQDA 120

CHCC5027_4207 AIHSRPEFRTTFNDKGQLGYWEQMHPCYTIFHQDDQTFSALWTEYSNDFSRFYRQYLQDA 120

CHCC14820_3541 AIHSRPEFRTTFNDKGQLGYWEQMHPCYTIFHQDDQTFSALWTEYSNDFSRFYRQYLQDA 120

CHCC4186_3571 AIHSRPEFRTTFNDKGQLGYWEQMHPCYTIFHQDDQTFSALWTEYSNDFSRFYRQYLQDA 120

CHCC20488_2389 AIHSRPEFRTTFNDKGQLGYWEQMHPCYTIFHQDDQTFSALWTEYSNDFSRFYRQYLQDA 120

CHCC20331_0965 AIHSRPEFRTTFNDKGQLGYWEQMHPCYTIFHQDDQTFSALWTEYSDDFSRFYRQYLQDA 120

CHCC20348_2795 AIHSRPEFRTTFNDKGQLGYWEQMHPCYTIFHQDDQTFSALWTEYSDDFSRFYRQYLQDA 120

CHCC12620_12620 AIHSRPEFRTTFNDKGQLGYWEQMHPCYTIFHQDDQTFSALWTEYSDDFSRFYRQYLQDA 120

CHCC20491_1156 AIHSRPEFRTTFNDKGQLGYWEQMHPCYTIFHQDDQTFSALWTEYSDDFSRFYRQYLQDA 120

CHCC15136_3048 AIHSRPEFRTTFNDKGQLGYWEQMHPCYTIFHQDDQTFSALWTEYSDDFSRFYRQYLQDA 120

CHCC14817_2262 AIHSRPEFRTTFNDKGQLGYWEQMHPCYTIFHQDDQTFSALWTEYSDDFSRFYRQYLQDA 120

BL09_3082 AIHSRPEFRTTFNDKGQLGYWEQMHPCYTIFHQDDQTFSALWTEYSDDFSRFYRQYLQDA 120

CHCC20490_4151 GIHSRPEFRTTFNDKGQLGYWEQMHPCYTIFHQDDQTFSALWTEYSDDFSRFYRQYLQDA 120

CHCC5019_3909 GIHSRPEFRTTFNDKGQLGYWEQMHPCYTIFHQDDQTFSALWTEYSDDFSRFYRQYLQDA 120

CHCC5023_4401 GIHSRPEFRTTFNDKGQLGYWEQMHPCYTIFHQDDQTFSALWTEYSDDFSRFYRQYLQDA 120

CHCC14814_4408 AIHSRPEFRTTFNGQGQLGYWEQMHPCYTIFHQDDHTFSALWTEYSDDFSRFYRQYLQDA 120

CHCC20375_2721 VIHSRPEFRTTFDDKGQLGYWEQMHPCYTIFHQDDQTFSALWTEYSDDFSQFYRQYLLDA 120

CHCC20494_3859 VIHSRPEFRTTFDDKGRLGYWEQMHPCYAIFHQDDQTFSALWTEYLDDFSQFYHQYLLDA 120

CHCC20493_4099 VIHSRPEFRTTFDDKGRLGYWEQMHPCYAIFHQDDQTFSALWTEYLDDFSQFYHQYLLDA 120

CHCC14557_2557 VIHSRPEFRTTFDDKGRLGYWEQMHPCYAIFHQDDQTFSALWTEYSDDFSQFYHQYLLDA 120

CHCC16874_3846 VIHSRPEFRTTFDDKGRLGYWEQMHPCYAIFHQDDQTFSALWTEYSDDFSQFYHQYLLDA 120

CHCC20496_2682 VIHSRPEFRTTFDDKGRLGYWEQMHPCYAIFHQDDQTFSALWTEYSDDFSQFYHQYLLDA 120

CHCC20339_2019 VIHSRPEFRTTFDDKGRLGYWEQMHPCYAIFHQDDQTFSALWTEYSDDFSQFYHQYLLDA 120

CHCC15325_3739 VIHSRPEFRTTFDDKGRLGYWEQMHPCYAIFHQDDQTFSALWTEYSDDFSQFYHQYLLDA 120

CHCC5025_4628 VIHSRPEFRTTFDDKGRLGYWEQMHPCYAIFHQDDQTFSALWTEYSDDFSQFYHQYLLDA 120

CHCC14429_1025 VIHSRPEFRTTFDDKGQLGYWEQMHPCYAIFHQDDQTFSALWTEYSDDFSQFYHQYLLDA 120

CHCC14435_1076 VIHSRPEFRTTFDDKGQLGYWEQMHPCYAIFHQDDQTFSALWTEYSDDFSQFYHQYLLDA 120

CHCC14437_3617 VIHSRPEFRTTFDDKGQLGYWEQMHPCYAIFHQDDQTFSALWTEYSDDFSQFYHQYLLDA 120

CHCC20327_4458 VIHSRPEFRTTFDDKGQLGYWEQMHPCYAIFHQDDQTFSALWTEYSDDFSQFYHQYLLDA 120

CHCC20489_3325 VIHSRPEFRTTFDDKGQLGYWEQMHPCYAIFHQDDQTFSALWTEYSDDFSQFYHQYLLDA 120

CHCC20486_3539 VIHSRPEFRTTFDDKGQLGYWEQMHPCYAIFHQDDQTFSALWTEYSDDFSQFYHQYLLDA 120

CHCC20487_0369 VIHSRPEFRTTFDDKGQLGYWEQMHPCYAIFHQDDQTFSALWTEYSDDFSQFYHQYLLDA 120

CHCC14441_2582 VIHSRPEFRTTFDDKGQLGYWEQMHPCYAIFHQDDQTFSALWTEYSDDFSQFYHQYLLDA 120

CHCC14688_4106 VIHSRPEFRTTFDDKGQLGYWEQMHPCYAIFHQDDQTFSALWTEYSDDFSQFYHQYLLDA 120

CHCC5020_0388 VIHSRPEFRTTFDDKGQLGYWEQMHPCYAIFHQDDQTFSALWTEYSDDFSQFYHQYLLDA 120

CHCC14816_2696 VIHSRPEFRTTFDDKGQLGYWEQMHPCYAIFHQDDQTFSALWTEYSDDFSQFYHQYLLDA 120

CHCC15292_1484 VIHSRPEFRTTFDDKGQLGYWEQMHPCYAIFHQDDQTFSALWTEYSDDFSQFYHQYLLDA 120

CHCC14561_0446 VIHSRPEFRTTFDDKGQLGYWEQMHPCYAIFHQDDQTFSALWTEYSDDFSQFYHQYLLDA 120

DSM13_2943 VIHSRPEFRTTFDDKGQLGYWEQMHPCYAIFHQDDQTFSALWTEYSDDFSQFYHQYLLDA 120

CHCC14600_2465 VIHSRPEFRTTFDDKGQLGYWEQMHPCYAIFHQDDQTFSALWTEYSDDFSQFYHQYLLDA 120

CHCC14596_4179 VIHSRPEFRTTFDDKGQLGYWEQMHPCYTIFHQDDQTFSALWTEYSDDFSQFYHQYLLDA 120

***********:.:*:***********:******:*:******* :***:**:*** **

CHCC15139_3882 ERFGDKRGLWAKPDIPPNTFSVSSIPWVSFTNFNLNLDNSEHLLPIITNGKYFSEGRETF 180

CHCC20344_2494 ERFGDKRGLWAKPDIPPNTFSVSSIPWVSFTNFNLNLDNSEHLLPIITNGKYFSEGRETF 180

CHCC20341_2443 ERFGDKRGLWAKPDIPPNTFSVSSIPWVSFTNFNLNLDNSEHLLPIITNGKYFSEGRETF 180

CHCC14818_3402 ERFGDKRGLWAKPDIPPNTFSVSSIPWVSFTNFNLNLDNSEHLLPIITNGKYFSEGRETF 180

CHCC14819_0760 ERFGDKRGLWAKPDIPPNTFSVSSIPWVSFTNFNLNLDNSEHLLPIITNGKYFSEGRETF 180

CHCC15291_3055 ERFGDKRGLWAKPDIPPNTFSVSSIPWVSFTNFNLNLDNSEHLLPIITNGKYFSEGRETF 180

CHCC14566_4422 ERFGDKRGLWAKQDIPPNTFSVSSIPWVSFTNFNLNLDNSEHLLPIITNGKYFSEGRETF 180

CHCC20342_0667 ERFGDKRGLWAKQDIPPNTFSVSSIPWVSFTNFNLNLDNSEHLLPIITNGKYFSEGRETF 180

CHCC20369_4383 ERFGDKRGLWAKQDIPPNTFSVSSIPWVSFTNFNLNLDNSEHLLPIITNGKYFSEGRETF 180

CHCC20368_3469 ERFGDKRGLWAKQDIPPNTFSVSSIPWVSFTNFNLNLDNSEHLLPIITNGKYFSEGRETF 180

CHCC14598_4486 ERFGDKRGLWAKPDIPPNTFSVSSIPWVSFTNFNLNLDNSEHLLPIITNGKYFSEGRETF 180

CHCC15289_3447 ERFGDKRGLWAKPDIPPNTFSVSSIPWVSFTNFNLNLDNSEHLLPIITNGKYFSEGRETF 180

CHCC20495_3293 ERFGDKRGLWAKPDIPPNTFSVSSIPWVSFTNFNLNLDNSEHLLPIITNGKYFSEGRETF 180

CHCC14810_3357 ERFGDKRGLWAKPDIPPNTFSVSSIPWVSFTNFNLNLDNSEHLLPIITNGKYFSEGRETF 180

CHCC20373_3167 ERFGDKRGLWAKPDIPPNTFSVSSIPWVSFTNFNLNLDNSEHLLPIITNGKYFSEGRETF 180

CHCC14564_0420 ERFGDKRGLWAKPDIPPNTFSVSSIPWVSFTNFNLNLDNSEHLLPIITNGKYFSEGRETF 180

CHCC14813_2931 ERFGDKRGLWAKPDIPPNTFSVSSIPWVSFTNFNLNLDNSEHLLPIITNGKYFSEGRETF 180

CHCC20442_0193 ERFGDKRGLWAKPDIPPNTFSVSSIPWVSFTNFNLNLDNSEHLLPIITNGKYFSEGRETF 180

CHCC14809_0373 ERFGDKRGLWAKPDIPPNTFSVSSIPWVSFTNFNLNLDNSEHLLPIITNGKYFSEGRETF 180

CHCC15320_1343 ERFGDKRGLWAKPDIPPNTFSVSSIPWVSFTNFNLNLDNSEHLLPIITNGKYFSEGRETF 180

CHCC14525_1227 ERFGDKRGLWAKPDIPPNTFSVSSIPWVSFTNFNLNLDNSEHLLPIITNGKYFSEGRETF 180

CHCC15318_0524 ERFGDKRGLWAKPDIPPNTFSVSSIPWVSFTNFNLNLDNSEHLLPIITNGKYFSEGRETF 180

CHCC20323_0869 ERFGDKRGLWAKPDIPPNTFSVSSIPWVSFTNFNLNLDNSEHLLPIITNGKYFSEGRETF 180

CHCC15087_4076 ERFGDKRGLWAKPDIPPNTFSVSSIPWVSFTNFNLNLDNSEHLLPIITNGKYFSEGRETF 180

CHCC14431_1770 ERFGDKRGLWAKPDIPPNTFSVSSIPWVSFTNFNLNLDNSEHLLPIITNGKYFSEGRETF 180

CHCC15091_0059 ERFGDKRGLWAKPDIPPNTFSVSSIPWVSFTNFNLNLDNSEHLLPIITNGKYFSEGRETF 180

CHCC19466_0660 ERFGDKRGLWAKPDIPPNTFSVSSIPWVSFTNFNLNLDNSEHLLPIITNGKYFSEGRETF 180

CHCC10893_4304 ERFGDKRGLWAKPDIPPNTFSVSSIPWVSFTNFNLNLDNSEHLLPIITNGKYFSEGRETF 180

CHCC15546_2749 ERFGDKRGLWAKPDIPPNTFSVSSIPWVSFTNFNLNLDNSEHLLPIITNGKYFSEGRETF 180

CHCC15543_2412 ERFGDKRGLWAKPDIPPNTFSVSSIPWVSFTNFNLNLDNSEHLLPIITNGKYFSEGRETF 180

CHCC16736_1335 ERFGDKRGLWAKPDIPPNTFSVSSIPWVSFTNFNLNLDNSEHLLPIITNGKYFSEGRETF 180

CHCC14559_1158 ERFGDKRGLWAKPDIPPNTFSVSSIPWVSFTNFNLNLDNSEHLLPIITNGKYFSEGRETF 180

CHCC20325_0259 ERFGDKRGLWAKPDIPPNTFSVSSIPWVSFTNFNLNLDNSEHLLPIITNGKYFSEGRETF 180

CHCC15315_0117 ERFGDKRGLWAKPDIPPNTFSVSSIPWVSFTNFNLNLDNSEHLLPIITNGKYFSEGRETF 180

CHCC15311_4297 ERFGDKRGLWAKPDIPPNTFSVSSIPWVSFTNFNLNLDNSEHLLPIITNGKYFSEGRETF 180

CHCC14568_3889 ERFGDKRGLWAKPDIPPNTFSVSSIPWVSFTNFNLNLDNSEHLLPIITNGKYFSEGRETF 180

CHCC15322_2635 ERFGDKRGLWAKPDIPPNTFSVSSIPWVSFTNFNLNLDNSEHLLPIITNGKYFSEGRETF 180

CHCC20345_2951 ERFGDKRGLWAKPDIPPNTFSVSSIPWVSFTNFNLNLDNSEHLLPIITNGKYFSEGRETF 180

CHCC20343_0747 ERFGDKRGLWAKPDIPPNTFSVSSIPWVSFTNFNLNLDNSEHLLPIITNGKYFSEGRETF 180

CHCC15335_1117 ERFGDKRGLWAKPDIPPNTFSVSSIPWVSFTNFNLNLDNSEHLLPIITNGKYFSEGRETF 180

CHCC14808_0727 ERFGDKRGLWAKPDIPPNTFSVSSIPWVSFTNFNLNLDNSEHLLPIITNGKYFSEGRETF 180

CHCC5026_2084 ERFGDKRGLWAKPDIPPNTFSVSSIPWVSFTNFNLNLDNSEHLLPIITNGKYFSEGRETF 180

CHCC5024_1205 ERFGDKRGLWAKPDIPPNTFSVSSIPWVSFTNFNLNLDNSEHLLPIITNGKYFSEGRETF 180

CHCC20440_0720 ERFGDKRGLWAKPDIPPNTFSVSSIPWVSFTNFNLNLDNSEHLLPIITNGKYFSEGRETF 180

CHCC20441_4480 ERFGDKRGLWAKPDIPPNTFSVSSIPWVSFTNFNLNLDNSEHLLPIITNGKYFSEGRETF 180

CHCC14815_4043 ERFGDKRGLWAKPDIPPNTFSVSSIPWVSFTNFNLNLDNSEHLLPIITNGKYFSEGRETF 180

CHCC15290_0718 ERFGDKRGLWAKPDIPPNTFSVSSIPWVSFTNFNLNLDNSEHLLPIITNGKYFSEGRETF 180

CHCC14562_3973 ERFGDKRGLWAKPDIPPNTFSVSSIPWVSFTNFNLNLDNSEHLLPIITNGKYFSEGRETF 180

CHCC15075_2813 ERFGDKRGLWAKPDIPPNTFSVSSIPWVSFTNFNLNLDNSEHLLPIITNGKYFSEGRETF 180

CHCC15381_0137 ERFGDKKGLWAKPDIPPNAFSVSSIPWVRFTNFNLNLDNSEHLLPIITNGKYFSEGGETF 180

CHCC20372_3730 ERFGDKKGLWAKPDIPPNAFSVSSIPWVRFTNFNLNLDNSEHLLPIITNGKYFSEGGETF 180

CHCC19467_2131 ERFGDKKGLWAKPDIPPNAFSVSSIPWVRFTNFNLNLDNSEHLLPIITNGKYFSEGGETF 180

CHCC19468_0817 ERFGDKKGLWAKPDIPPNAFSVSSIPWVRFTNFNLNLDNSEHLLPIITNGKYFSEGGETF 180

CHCC20497_3599 ERFGDKKGLWAKPDIPPNAFSVSSIPWVRFTNFNLNLDNSEHLLPIITNGKYFSEGGETF 180

CHCC20492_1406 ERFGDKKGLWAKPDIPPNAFSVSSIPWVRFTNFNLNLDNSEHLLPIITNGKYFSEGGETF 180

CHCC14523_2493 ERFGDKKGLWAKPDIPPNAFSVSSIPWVRFTNFNLNLDNSEHLLPIITNGKYFSEGGETF 180

CHCC14527_1718 ERFGDKKGLWAKPDIPPNAFSVSSIPWVRFTNFNLNLDNSEHLLPIITNGKYFSEGGETF 180

CHCC20333_1346 ERFGDKKGLWAKPDIPPNAFSVSSIPWVRFTNFNLNLDNSEHLLPIITNGKYFSEGGETF 180

ATCC9945A_3028 ERFGDKKGLWAKPDIPPNAFSVSSIPWVRFTNFNLNLDNSEHLLPIITNGKYFSEGGETF 180

CHCC20347_3632 ERFGDKKGLWAKPDIPPNAFSVSSIPWVRFTNFNLNLDNSEHLLPIITNGKYFSEGGETF 180

CHCC15337_3824 ERFGDKKGLWAKPDIPPNAFSVSSIPWVRFTNFNLNLDNSEHLLPIITNGKYFSEGGETF 180

CHCC15332_2367 ERFGDKKGLWAKPDIPPNAFSVSSIPWVRFTNFNLNLDNSEHLLPIITNGKYFSEGGETF 180

CHCC5022_4011 ERFGDKKGLWAKPDIPPNAFSVSSIPWVRFTNFNLNLDNSEHLLPIITNGKYFSEGGETF 180

CHCC5021_0537 ERFGDKKGLWAKPDIPPNAFSVSSIPWVRFTNFNLNLDNSEHLLPIITNGKYFSEGGETF 180

CHCC5027_4207 ERFGDKKGLWAKPDIPPNAFSVSSIPWVRFTNFNLNLDNSEHLLPIITNGKYFSEGGETF 180

CHCC14820_3541 ERFGDKKGLWAKPDIPPNAFSVSSIPWVRFTNFNLNLDNSEHLLPIITNGKYFSEGGETF 180

CHCC4186_3571 ERFGDKKGLWAKPDIPPNAFSVSSIPWVRFTNFNLNLDNSEHLLPIITNGKYFSEGGETF 180

CHCC20488_2389 ERFGDKKGLWAKPDIPPNAFSVSSIPWVRFTNFNLNLDNSEHLLPIITNGKYFSEGGETF 180

CHCC20331_0965 ERFGDKKGLWAKPDIPPNAFSVSSIPWVRFTNFNLNLDNSEHLLPIITNGKYFSEGGETF 180

CHCC20348_2795 ERFGDKKGLWAKPDIPPNAFSVSSIPWVRFTNFNLNLDNSEHLLPIITNGKYFSEGGETF 180

CHCC12620_12620 ERFGDKKGLWAKPDIPPNAFSVSSIPWVRFTNFNLNLDNSEHLLPIITNGKYFSEGGETF 180

CHCC20491_1156 ERFGDKKGLWAKPDIPPNAFSVSSIPWVRFTNFNLNLDNSEHLLPIITNGKYFSEGGETF 180

CHCC15136_3048 ERFGDKKGLWAKPDIPPNAFSVSSIPWVRFTNFNLNLDNSEHLLPIITNGKYFSEGGETF 180

CHCC14817_2262 ERFGDKKGLWAKPDIPPNAFSVSSIPWVRFTNFNLNLDNSEHLLPIITNGKYFSEGGETF 180

BL09_3082 ERFGDKKGLWAKPDIPPNAFSVSSIPWVRFTNFNLNLDNSEHLLPIITNGKYFSEGGETF 180

CHCC20490_4151 ERFGDKKGLWAKPDIPPNAFSVSSIPWVRFTNFNLNLDNSEHLLPIITNGKYFSEGGETF 180

CHCC5019_3909 ERFGDKKGLWAKPDIPPNAFSVSSIPWVRFTNFNLNLDNSEHLLPIITNGKYFSEGGETF 180

CHCC5023_4401 ERFGDKKGLWAKPDIPPNAFSVSSIPWVRFTNFNLNLDNSEHLLPIITNGKYFSEGGETF 180

CHCC14814_4408 ERFGDKKGLWAKPDIPPNTFSVSSIPWVRFTNFNLNLDNSEHLLPMITNGKYFSEGSETF 180

CHCC20375_2721 ERFGDKRGLWGKPDIPPNTFSVSSIPWVSFTNFNLNLDNSEHLLPIITNGKYFSEGKETF 180

CHCC20494_3859 ERFGDKRGLWAKPDIPPNTFSVSSIPWVRFSNFNLNLDNSEHLLPIITNGKYFSEGRETF 180

CHCC20493_4099 ERFGDKRGLWAKPDIPPNTFSVSSIPWVRFSNFNLNLDNSEHLLPIITNGKYFSEGRETF 180

CHCC14557_2557 ERFGDKRGLWAKPDIPPNTFSVSSIPWVRFSNFNLNLDNSEHLLPIITNGKYFSEGRETF 180

CHCC16874_3846 ERFGDKRGLWAKPDIPPNTFSVSSIPWVRFSNFNLNLDNSEHLLPIITNGKYFSEGRETF 180

CHCC20496_2682 ERFGDKRGLWAKPDIPPNTFSVSSIPWVRFSNFNLNLDNSEHLLPIITNGKYFSEGRETF 180

CHCC20339_2019 ERFGDKRGLWAKPDIPPNTFSVSSIPWVRFSNFNLNLDNSEHLLPIITNGKYFSEGRETF 180

CHCC15325_3739 ERFGDKRGLWAKPDIPPNTFSVSSIPWVRFSNFNLNLDNSEHLLPIITNGKYFSEGRETF 180

CHCC5025_4628 ERFGDKRGLWAKPDIPPNTFSVSSIPWVRFSNFNLNLDNSEHLLPIITNGKYFSEGRETF 180

CHCC14429_1025 ERFGDKRGLWAKPDIPPNTFSVSSIPWVRFSNFNLNLDNSEHLLPIITNGKYFSEGRETF 180

CHCC14435_1076 ERFGDKRGLWAKPDIPPNTFSVSSIPWVRFSNFNLNLDNSEHLLPIITNGKYFSEGRETF 180

CHCC14437_3617 ERFGDKRGLWAKPDIPPNTFSVSSIPWVRFSNFNLNLDNSEHLLPIITNGKYFSEGRETF 180

CHCC20327_4458 ERFGDKRGLWAKPDIPPNTFSVSSIPWVRFSNFNLNLDNSEHLLPIITNGKYFSEGRETF 180

CHCC20489_3325 ERFGDKRGLWAKPDIPPNTFSVSSIPWVRFSNFNLNLDNSEHLLPIITNGKYFSEGRETF 180

CHCC20486_3539 ERFGDKRGLWAKPDIPPNTFSVSSIPWVRFSNFNLNLDNSEHLLPIITNGKYFSEGRETF 180

CHCC20487_0369 ERFGDKRGLWAKPDIPPNTFSVSSIPWVRFSNFNLNLDNSEHLLPIITNGKYFSEGRETF 180

CHCC14441_2582 ERFGDKRGLWAKPDIPPNTFSVSSIPWVRFSNFNLNLDNSEHLLPIITNGKYFSEGRETF 180

CHCC14688_4106 ERFGDKRGLWAKPDIPPNTFSVSSIPWVRFSNFNLNLDNSEHLLPIITNGKYFSEGRETF 180

CHCC5020_0388 ERFGDKRGLWAKPDIPPNTFSVSSIPWVRFSNFNLNLDNSEHLLPIITNGKYFSEGRETF 180

CHCC14816_2696 ERFGDKRGLWAKPDIPPNTFSVSSIPWVRFSNFNLNLDNSEHLLPIITNGKYFSEGRETF 180

CHCC15292_1484 ERFGDKRGLWAKPDIPPNTFSVSSIPWVRFSNFNLNLDNSEHLLPIITNGKYFSEGRETF 180

CHCC14561_0446 ERFGDKRGLWAKPDIPPNTFSVSSIPWVRFSNFNLNLDNSEHLLPIITNGKYFSEGRETF 180

DSM13_2943 ERFGDKRGLWAKPDIPPNTFSVSSIPWVRFSNFNLNLDNSEHLLPIITNGKYFSEGRETF 180

CHCC14600_2465 ERFGDKRGLWAKPDIPPNTFSVSSIPWVRFSNFNLNLDNSEHLLPIITNGKYFSEGRETF 180

CHCC14596_4179 ERFGDKRGLWAKPDIPPNTFSVSSIPWVSFTNFNLNLDNSEHLLPIITNGKYFSEGRETF 180

******:***.* *****:********* ::**************:********** ***

CHCC15139_3882 LPVSLQVHHAVCDGYHAGAFMNELARLAADCKEWLV 216

CHCC20344_2494 LPVSLQVHHAVCDGYHAGAFMNELARLAADCKEWLV 216

CHCC20341_2443 LPVSLQVHHAVCDGYHAGAFMNELARLAADCKEWLV 216

CHCC14818_3402 LPVSLQVHHAVCDGYHAGAFMNELARLAADCKEWLV 216

CHCC14819_0760 LPVSLQVHHAVCDGYHAGAFMNELARLAADCKEWLV 216

CHCC15291_3055 LPVSLQVHHAVCDGYHAGAFMNELARLAADCKEWLV 216

CHCC14566_4422 LPVSLQVHHAVCDGYHAGAFMNELARLAADCKEWLV 216

CHCC20342_0667 LPVSLQVHHAVCDGYHAGAFMNELARLAADCKEWLV 216

CHCC20369_4383 LPVSLQVHHAVCDGYHAGAFMNELARLAADCKEWLV 216

CHCC20368_3469 LPVSLQVHHAVCDGYHAGAFMNELARLAADCKEWLV 216

CHCC14598_4486 LPVSLQVHHAVCDGYHAGAFMNELARLAADCKEWLV 216

CHCC15289_3447 LPVSLQVHHAVCDGYHAGAFMNELARLAADCKEWLV 216

CHCC20495_3293 LPVSLQVHHAVCDGYHAGAFMNELARLAADCKEWLV 216

CHCC14810_3357 LPVSLQVHHAVCDGYHAGAFMNELARLAADCKEWLV 216

CHCC20373_3167 LPVSLQVHHAVCDGYHAGAFMNELARLAADCKEWLV 216

CHCC14564_0420 LPVSLQVHHAVCDGYHAGAFMNELARLAADCKEWLV 216

CHCC14813_2931 LPVSLQVHHAVCDGYHAGAFMNELARLAADCKEWLV 216

CHCC20442_0193 LPVSLQVHHAVCDGYHAGAFMNELARLAADCKEWLV 216

CHCC14809_0373 LPVSLQVHHAVCDGYHAGAFMNELARLAADCKEWLV 216

CHCC15320_1343 LPVSLQVHHAVCDGYHAGAFMNELARLAADCKEWLV 216

CHCC14525_1227 LPVSLQVHHAVCDGYHAGAFMNELARLAADCKEWLV 216

CHCC15318_0524 LPVSLQVHHAVCDGYHAGAFMNELARLAADCKEWLV 216

CHCC20323_0869 LPVSLQVHHAVCDGYHAGAFMNELARLAADCKEWLV 216

CHCC15087_4076 LPVSLQVHHAVCDGYHAGAFMNELARLAADCKEWLV 216

CHCC14431_1770 LPVSLQVHHAVCDGYHAGAFMNELARLAADCKEWLV 216

CHCC15091_0059 LPVSLQVHHAVCDGYHAGAFMNELARLAADCKEWLV 216

CHCC19466_0660 LPVSLQVHHAVCDGYHAGAFMNELARLAADCKEWLV 216

CHCC10893_4304 LPVSLQVHHAVCDGYHAGAFMNELARLAADCKEWLV 216

CHCC15546_2749 LPVPCKYTMPCVT-----AIMPAPS----------- 200

CHCC15543_2412 LPVPCKYTMPCVT-----AIMPAPS----------- 200

CHCC16736_1335 LPVPCKYTMPCVT-----AIMPAPS----------- 200

CHCC14559_1158 LPVPCKYTMPCVT-----AIMPAPS----------- 200

CHCC20325_0259 LPVPCKYTMPCVT-----AIMPAPS----------- 200

CHCC15315_0117 LPVPCKYTMPCVT-----AIMPAPS----------- 200

CHCC15311_4297 LPVPCKYTMPCVT-----AIMPAPS----------- 200

CHCC14568_3889 LPVPCKYTMPCVT-----AIMPAPS----------- 200

CHCC15322_2635 LPVPCKYTMPCVT-----AIMPAPS----------- 200

CHCC20345_2951 LPVPCKYTMPCVT-----AIMPAPS----------- 200

CHCC20343_0747 LPVPCKYTMPCVT-----AIMPAPS----------- 200

CHCC15335_1117 LPVPCKYTMPCVT-----AIMPAPS----------- 200

CHCC14808_0727 LPVPCKYTMPCVT-----AIMPAPS----------- 200

CHCC5026_2084 LPVPCKYTMPCVT-----AIMPAPS----------- 200

CHCC5024_1205 LPVPCKYTMPCVT-----AIMPAPS----------- 200

CHCC20440_0720 LPVPCKYTMPCVT-----AIMPAPS----------- 200

CHCC20441_4480 LPVPCKYTMPCVT-----AIMPAPS----------- 200

CHCC14815_4043 LPVPCKYTMPCVT-----AIMPAPS----------- 200

CHCC15290_0718 LPVPCKYTMPCVT-----AIMPAPS----------- 200

CHCC14562_3973 LPVPCKYTMPCVT-----AIMPAPS----------- 200

CHCC15075_2813 LPVPCKYTMPCVT-----AIMPAPS----------- 200

CHCC15381_0137 LPVSLQVHHAVCDGYHAGAFMNELERLAADCEEWLM 216

CHCC20372_3730 LPVSLQVHHAVCDGYHAGAFMNELERLAADCEEWLM 216

CHCC19467_2131 LPVSLQVHHAVCDGYHAGAFMNELERLAADCEEWLM 216

CHCC19468_0817 LPVSLQVHHAVCDGYHAGAFMNELERLAADCEEWLM 216

CHCC20497_3599 LPVSLQVHHAVCDGYHAGAFMNELERLAADCEEWLM 216

CHCC20492_1406 LPVSLQVHHAVCDGYHAGAFMNELERLAADCEEWLM 216

CHCC14523_2493 LPVSLQVHHAVCDGYHAGAFMNELERLAADCEEWLM 216

CHCC14527_1718 LPVSLQVHHAVCDGYHAGAFMNELERLAADCEEWLM 216

CHCC20333_1346 LPVSLQVHHAVCDGYHAGAFMNELERLAADCEEWLM 216

ATCC9945A_3028 LPVSLQVHHAVCDGYHAGAFMNELERLAADCEEWLM 216

CHCC20347_3632 LPVSLQVHHAVCDGYHAGAFMNELERLAADCEEWLM 216

CHCC15337_3824 LPVSLQVHHAVCDGYHAGAFMNELERLAADCEEWLM 216

CHCC15332_2367 LPVSLQVHHAVCDGYHAGAFMNELERLAADCEEWLM 216

CHCC5022_4011 LPVSLQVHHAVCDGYHAGAFMNELERLAADCEEWLM 216

CHCC5021_0537 LPVSLQVHHAVCDGYHAGAFMNELERLAADCEEWLM 216

CHCC5027_4207 LPVSLQVHHAVCDGYHAGAFMNELERLAADCEEWLM 216

CHCC14820_3541 LPVSLQVHHAVCDGYHAGAFMNELERLAADCEEWLM 216

CHCC4186_3571 LPVSLQVHHAVCDGYHAGAFMNELERLAADCEEWLM 216

CHCC20488_2389 LPVSLQVHHAVCDGYHAGAFMNELERLAADCEEWLM 216

CHCC20331_0965 LPVSLQVHHAVCDGYHAGAFMNELERLAADCEEWLM 216

CHCC20348_2795 LPVSLQVHHAVCDGYHAGAFMNELERLAADCEEWLM 216

CHCC12620_12620 LPVSLQVHHAVCDGYHAGAFMNELERLAADCEEWLM 216

CHCC20491_1156 LPVSLQVHHAVCDGYHAGAFMNELERLAADCEEWLM 216

CHCC15136_3048 LPVSLQVHHAVCDGYHAGAFMNELERLAADCEEWLM 216

CHCC14817_2262 LPVSLQVHHAVCDGYHAGAFMNELERLAADCEEWLM 216

BL09_3082 LPVSLQVHHAVCDGYHAGAFMNELERLAADCEEWLM 216

CHCC20490_4151 LPVSLQVHHAVCDGYHAGAFMNELERLAADCEEWLM 216

CHCC5019_3909 LPVSLQVHHAVCDGYHAGAFMNELERLAADCEEWLM 216

CHCC5023_4401 LPVSLQVHHAVCDGYHAGAFMNELERLAADCEEWLM 216

CHCC14814_4408 LPVSLQVHHAVCDGYHAGAFMNELERLAADCEEWLV 216

CHCC20375_2721 LPVSLQVHHAVCDGYHAGAFMNELERLAADCEEWLV 216

CHCC20494_3859 LPVSLQVHHAVCDGYHAGAFINELERLAADCEEWLV 216

CHCC20493_4099 LPVSLQVHHAVCDGYHAGAFINELERLAADCEEWLV 216

CHCC14557_2557 LPVSLQVHHAVCDGYHAGAFINELERLAADCEEWLV 216

CHCC16874_3846 LPVSLQVHHAVCDGYHAGAFINELERLAADCEEWLV 216

CHCC20496_2682 LPVSLQVHHAVCDGYHAGAFINELERLAADCEEWLV 216

CHCC20339_2019 LPVSLQVHHAVCDGYHAGAFINELERLAADCEEWLV 216

CHCC15325_3739 LPVSLQVHHAVCDGYHAGAFINELERLAADCEEWLV 216

CHCC5025_4628 LPVSLQVHHAVCDGYHAGAFINELERLAADCEEWLV 216

CHCC14429_1025 LPVSLQVHHAVCDGYHAGAFINELERLAADCEEWLV 216

CHCC14435_1076 LPVSLQVHHAVCDGYHAGAFINELERLAADCEEWLV 216

CHCC14437_3617 LPVSLQVHHAVCDGYHAGAFINELERLAADCEEWLV 216

CHCC20327_4458 LPVSLQVHHAVCDGYHAGAFINELERLAADCEEWLV 216

CHCC20489_3325 LPVSLQVHHAVCDGYHAGAFINELERLAADCEEWLV 216

CHCC20486_3539 LPVSLQVHHAVCDGYHAGAFINELERLAADCEEWLV 216

CHCC20487_0369 LPVSLQVHHAVCDGYHAGAFINELERLAADCEEWLV 216

CHCC14441_2582 LPVSLQVHHAVCDGYHAGAFINELERLAADCEEWLV 216

CHCC14688_4106 LPVSLQVHHAVCDGYHAGAFINELERLAADCEEWLV 216

CHCC5020_0388 LPVSLQVHHAVCDGYHAGAFINELERLAADCEEWLV 216

CHCC14816_2696 LPVSLQVHHAVCDGYHAGAFINELERLAADCEEWLV 216

CHCC15292_1484 LPVSLQVHHAVCDGYHAGAFINELERLAADCEEWLV 216

CHCC14561_0446 LPVSLQVHHAVCDGYHAGAFINELERLAADCEEWLV 216

DSM13_2943 LPVSLQVHHAVCDGYHAGAFINELERLAADCEEWLV 216

CHCC14600_2465 LPVSLQVHHAVCDGYHAGAFMNELERLAADCEEWLV 216

CHCC14596_4179 LPVSLQVHHAVCDGYHAGAFMNELARLAADCKEWLV 216

***. : . *::
