# Supplementary material for: Putative antibiotic resistance genes present in extant Bacillus licheniformis and Bacillus paralicheniformis strains are probably intrinsic and part of the ancient resistome
Source: PLoS One. 2019 Jan 15;14(1):e0210363. doi: 10.1371/journal.pone.0210363 (PMC6333372; doi:10.1371/journal.pone.0210363)
Supplement: S4 Fig — Cat proteins were aligned using Muscle [33, 34] and the phylogenetic tree (approximate maximum likelihood) was constructed using FastTree [35]. (PPTX) [file pone.0210363.s004.pptx]

## Slide 1
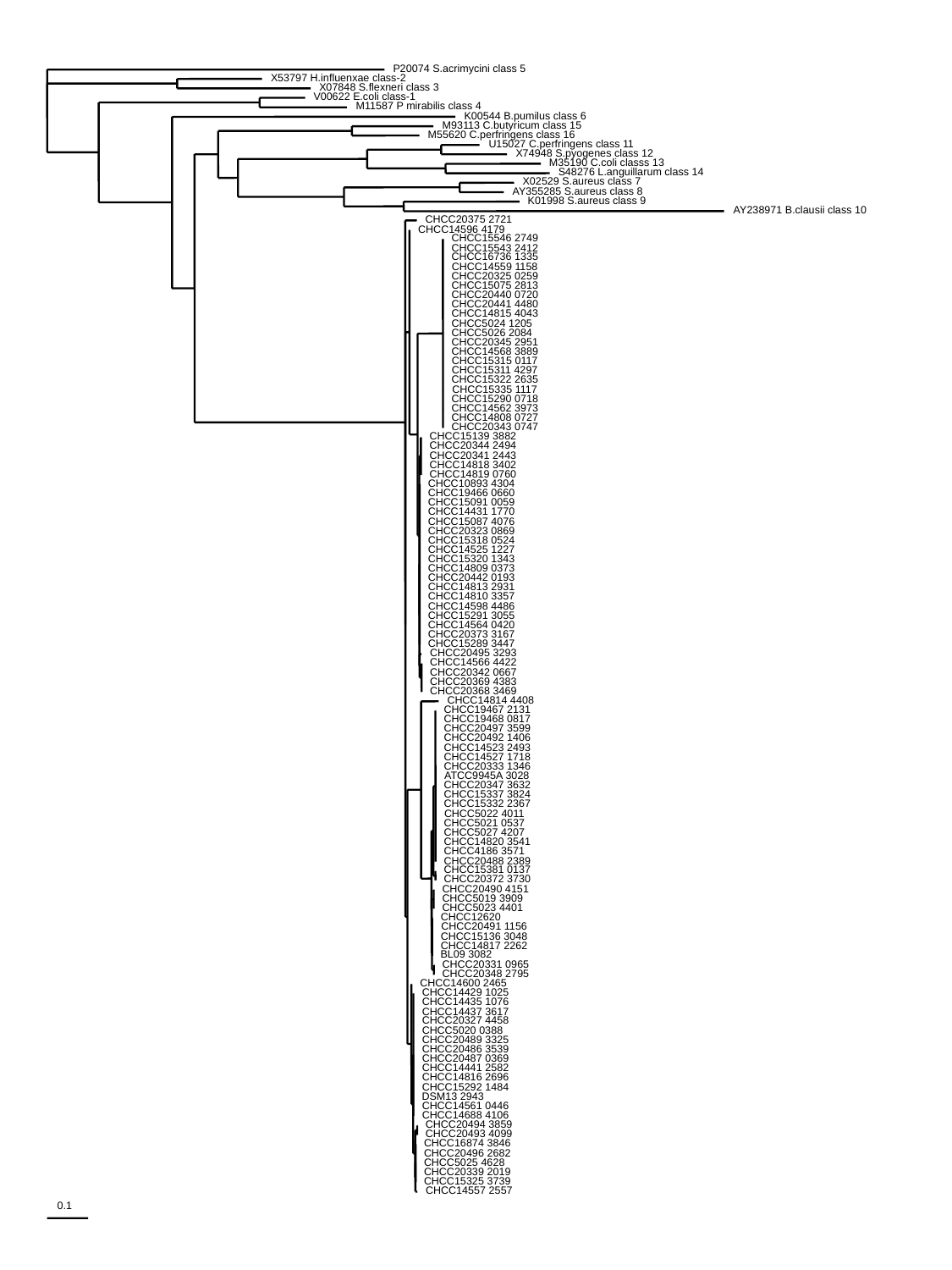

P20074 S.acrimycini class 5
X53797 H.influenxae class-2
X07848 S.flexneri class 3
V00622 E.coli class-1
M11587 P mirabilis class 4
K00544 B.pumilus class 6
M93113 C.butyricum class 15
M55620 C.perfringens class 16
U15027 C.perfringens class 11
X74948 S.pyogenes class 12
M35190 C.coli classs 13
S48276 L.anguillarum class 14
X02529 S.aureus class 7
AY355285 S.aureus class 8
K01998 S.aureus class 9
AY238971 B.clausii class 10
CHCC20375 2721
CHCC14596 4179
CHCC15546 2749
CHCC15543 2412
CHCC16736 1335
CHCC14559 1158
CHCC20325 0259
CHCC15075 2813
CHCC20440 0720
CHCC20441 4480
CHCC14815 4043
CHCC5024 1205
CHCC5026 2084
CHCC20345 2951
CHCC14568 3889
CHCC15315 0117
CHCC15311 4297
CHCC15322 2635
CHCC15335 1117
CHCC15290 0718
CHCC14562 3973
CHCC14808 0727
CHCC20343 0747
CHCC15139 3882
CHCC20344 2494
CHCC20341 2443
CHCC14818 3402
CHCC14819 0760
CHCC10893 4304
CHCC19466 0660
CHCC15091 0059
CHCC14431 1770
CHCC15087 4076
CHCC20323 0869
CHCC15318 0524
CHCC14525 1227
CHCC15320 1343
CHCC14809 0373
CHCC20442 0193
CHCC14813 2931
CHCC14810 3357
CHCC14598 4486
CHCC15291 3055
CHCC14564 0420
CHCC20373 3167
CHCC15289 3447
CHCC20495 3293
CHCC14566 4422
CHCC20342 0667
CHCC20369 4383
CHCC20368 3469
CHCC14814 4408
CHCC19467 2131
CHCC19468 0817
CHCC20497 3599
CHCC20492 1406
CHCC14523 2493
CHCC14527 1718
CHCC20333 1346
ATCC9945A 3028
CHCC20347 3632
CHCC15337 3824
CHCC15332 2367
0.1
CHCC5022 4011
CHCC5021 0537
CHCC5027 4207
CHCC14820 3541
CHCC4186 3571
CHCC20488 2389
CHCC15381 0137
CHCC20372 3730
CHCC20490 4151
CHCC5019 3909
CHCC5023 4401
CHCC12620
CHCC20491 1156
CHCC15136 3048
CHCC14817 2262
BL09 3082
CHCC20331 0965
CHCC20348 2795
CHCC14600 2465
CHCC14429 1025
CHCC14435 1076
CHCC14437 3617
CHCC20327 4458
CHCC5020 0388
CHCC20489 3325
CHCC20486 3539
CHCC20487 0369
CHCC14441 2582
CHCC14816 2696
CHCC15292 1484
DSM13 2943
CHCC14561 0446
CHCC14688 4106
CHCC20494 3859
CHCC20493 4099
CHCC16874 3846
CHCC20496 2682
CHCC5025 4628
CHCC20339 2019
CHCC15325 3739
CHCC14557 2557
