# Supplementary material for: Putative antibiotic resistance genes present in extant Bacillus licheniformis and Bacillus paralicheniformis strains are probably intrinsic and part of the ancient resistome
Source: PLoS One. 2019 Jan 15;14(1):e0210363. doi: 10.1371/journal.pone.0210363 (PMC6333372; doi:10.1371/journal.pone.0210363)
Supplement: S6 Fig — Sequence alignments were made with Clustal 2.1 using default settings. The left column indicates the locus tag of each aadK gene per strain. Identical amino acids are indicated by an asterisk below each column. Residue numbers are indicated at the end of each row. (DOCX) [file pone.0210363.s006.docx]

CHCC15381_2836 MRTEQEIIDLVLKVAREDDRVRAVGMNGSRTNP-------NVPKDPFRDYDMVYVVTDMQ 53

CHCC12620_0739 MRTEQEIIDLVLKVAREDDRVRAVGMNGSRTNP-------NVPKDPFRDYDMVYVVTDMQ 53

CHCC19467_4580 MRTEQEIIDLVLKVAREDDRVRAVGMNGSRTNP-------NVPKDPFRDYDMVYVVTDMQ 53

CHCC19468_2703 MRTEQEIIDLVLKVAREDDRVRAVGMNGSRTNP-------NVPKDPFRDYDMVYVVTDMQ 53

CHCC20497_4637 MRTEQEIIDLVLKVAREDDRVRAVGMNGSRTNP-------NVPKDPFRDYDMVYVVTDMQ 53

CHCC20491_0559 MRTEQEIIDLVLKVAREDDRVRAVGMNGSRTNP-------NVPKDPFRDYDMVYVVTDMQ 53

CHCC20492_2745 MRTEQEIIDLVLKVAREDDRVRAVGMNGSRTNP-------NVPKDPFRDYDMVYVVTDMQ 53

CHCC14523_3914 MRTEQEIIDLVLKVAREDDRVRAVGMNGSRTNP-------NVPKDPFRDYDMVYVVTDMQ 53

CHCC14527_2372 MRTEQEIIDLVLKVAREDDRVRAVGMNGSRTNP-------NVPKDPFRDYDMVYVVTDMQ 53

ATCC9945A_0220 MRTEQEIIDLVLKVAREDDRVRAVGMNGSRTNP-------NVPKDPFRDYDMVYVVTDMQ 53

CHCC15136_3271 MRTEQEIIDLVLKVAREDDRVRAVGMNGSRTNP-------NVPKDPFRDYDMVYVVTDMQ 53

CHCC5019_4456 MRTEQEIIDLVLKVAREDDRVRAVGMNGSRTNP-------NVPKDPFRDYDMVYVVTDMQ 53

CHCC15337_4610 MRTEQEIIDLVLKVAREDDRVRAVGMNGSRTNP-------NVPKDPFRDYDMVYVVTDMQ 53

CHCC15332_3737 MRTEQEIIDLVLKVAREDDRVRAVGMNGSRTNP-------NVPKDPFRDYDMVYVVTDMQ 53

CHCC5022_3897 MRTEQEIIDLVLKVAREDDRVRAVGMNGSRTNP-------NVPKDPFRDYDMVYVVTDMQ 53

CHCC5023_1979 MRTEQEIIDLVLKVAREDDRVRAVGMNGSRTNP-------NVPKDPFRDYDMVYVVTDMQ 53

CHCC5021_2115 MRTEQEIIDLVLKVAREDDRVRAVGMNGSRTNP-------NVPKDPFRDYDMVYVVTDMQ 53

CHCC14817_1577 MRTEQEIIDLVLKVAREDDRVRAVGMNGSRTNP-------NVPKDPFRDYDMVYVVTDMQ 53

CHCC4186_2037 MRTEQEIIDLVLKVAREDDRVRAVGMNGSRTNP-------NVPKDPFRDYDMVYVVTDMQ 53

CHCC20488_0914 MRTEQEIIDLVLKVAREDDRVRAVGMNGSRTNP-------NVPKDPFRDYDMVYVVTDMQ 53

BL09_0219 MRTEQEIIDLVLKVAREDDRVRAVGMNGSRTNP-------NVPKDPFRDYDMVYVVTDMQ 53

CHCC20331_1437 MRTEQEIIDLVLKVAREDDRVRAVGMNGSRTNP-------NVPKDPFRDYDMVYVVTDMQ 53

CHCC20347_3231 MRTEQEIIDLVLKVAREDDRVRAVGMNGSRTNP-------NVPKDPFRDYDMVYVVTDMQ 53

CHCC20333_3258 MRTEQEIIDLVLKVAREDDRVRAVGMNGSRTNP-------NVPKDPFRDYDMVYVVTDMQ 53

CHCC20490_1916 MRTEQEIIDLVLKVAREDDRVRAVGMNGSRTNP-------NVPKDPFRDYDMVYVVTDMQ 53

CHCC20348_4391 MRTEQEIIDLVLKVAREDDRVRAVGMNGSRTNP-------NVPKDPFRDYDMVYVVTDMQ 53

CHCC5027_3368 MRTEQEIIDLVLKVAREDDRVRAVGMNGSRTNP-------NVPKDPFRDYDMVYVVTDMQ 53

CHCC14820_1469 MRTEQEIIDLVLKVAREDDRVRAVGMNGSRTNP-------NVPKDPFRDYDMVYVVTDMQ 53

CHCC20372_2718 MRTEQEIIDLVLKVAREDDRVRAVGMNGSRTNP-------NVPKDPFRDYDMVYVVTDMQ 53

CHCC20373_0250 MRTEQEIIDLVLKVAREDSRVRAVGMNGSRTNP-------NVPKDPFRDYDIVYVVTDMQ 53

CHCC15289_3953 MRTEQEIIDLVLKVAREDSRVRAVGMNGSRTNP-------NVPKDPFRDYDIVYVVTDMQ 53

CHCC14564_2767 MRTEQEIIDLVLKVAREDSRVRAVGMNGSRTNP-------NVPKDPFRDYDIVYVVTDMQ 53

CHCC15290_2999 MRTEQEIIDLVLKVAREDSRVRAVGMNGSRTNP-------NVPKDPFRDYDIVYVVTDMQ 53

CHCC20368_2019 MRTEQEIIDLVLKVAREDSRVRAVGMNGSRTNP-------NVPKDPFRDYDIVYVVTDMQ 53

CHCC15291_0394 MRTEQEIIDLVLKVAREDSRVRAVGMNGSRTNP-------NVPKDPFRDYDIVYVVTDMQ 53

CHCC20369_2215 MRTEQEIIDLVLKVAREDSRVRAVGMNGSRTNP-------NVPKDPFRDYDIVYVVTDMQ 53

CHCC14598_1058 MRTEQEIIDLVLKVAREDSRVRAVGMNGSRTNP-------NVPKDPFRDYDIVYVVTDMQ 53

CHCC14596_0534 MRTEQEIIDLVLKVAREDSRVRAVGMNGSRTNP-------NVPKDPFRDYDIVYVVTDMQ 53

CHCC14810_4312 MRTEQEIIDLVLKVAREDSRVRAVGMNGSRTNP-------NVPKDPFRDYDIVYVVTDMQ 53

CHCC14819_0399 MRTEQEIIDLVLKVAREDSRVRAVGMNGSRTNP-------NVPKDPFRDYDIVYVVTDMQ 53

CHCC14813_2856 MRTEQEIIDLVLKVAREDSRVRAVGMNGSRTNP-------NVPKDPFRDYDIVYVVTDMQ 53

CHCC20441_1080 MRTEQEIIDLVLKVAREDSRVRAVGMNGSRTNP-------NVPKDPFRDYDIVYVVTDMQ 53

CHCC20440_1830 MRTEQEIIDLVLKVAREDSRVRAVGMNGSRTNP-------NVPKDPFRDYDIVYVVTDMQ 53

CHCC20442_1308 MRTEQEIIDLVLKVAREDSRVRAVGMNGSRTNP-------NVPKDPFRDYDIVYVVTDMQ 53

CHCC5024_4269 MRTEQEIIDLVLKVAREDSRVRAVGMNGSRTNP-------NVPKDPFRDYDIVYVVTDMQ 53

CHCC5026_1706 MRTEQEIIDLVLKVAREDSRVRAVGMNGSRTNP-------NVPKDPFRDYDIVYVVTDMQ 53

CHCC5020_0926 MRTEQEIIDLVLKVAREDSRVRAVGMNGSRTNP-------NVPKDPFRDYDIVYVVTDMQ 53

CHCC14600_3227 MRTEQEIIDLVLKVAREDSRVRAVGMNGSRTNP-------NVPKDPFRDYDIVYVVTDMQ 53

CHCC14808_3671 MRTEQEIIDLVLKVAREDSRVRAVGMNGSRTNP-------NVPKDPFRDYDIVYVVTDMQ 53

CHCC14809_3060 MRTEQEIIDLVLKVAREDSRVRAVGMNGSRTNP-------NVPKDPFRDYDIVYVVTDMQ 53

CHCC15335_1764 MRTEQEIIDLVLKVAREDSRVRAVGMNGSRTNP-------NVPKDPFRDYDIVYVVTDMQ 53

CHCC20342_1142 MRTEQEIIDLVLKVAREDSRVRAVGMNGSRTNP-------NVPKDPFRDYDIVYVVTDMQ 53

CHCC20343_0033 MRTEQEIIDLVLKVAREDSRVRAVGMNGSRTNP-------NVPKDPFRDYDIVYVVTDMQ 53

CHCC20341_2338 MRTEQEIIDLVLKVAREDSRVRAVGMNGSRTNP-------NVPKDPFRDYDIVYVVTDMQ 53

CHCC20344_4475 MRTEQEIIDLVLKVAREDSRVRAVGMNGSRTNP-------NVPKDPFRDYDIVYVVTDMQ 53

CHCC15139_1359 MRTEQEIIDLVLKVAREDSRVRAVGMNGSRTNP-------NVPKDPFRDYDIVYVVTDMQ 53

CHCC15320_2309 MRTEQEIIDLVLKVAREDSRVRAVGMNGSRTNP-------NVPKDPFRDYDIVYVVTDMQ 53

CHCC15322_3643 MRTEQEIIDLVLKVAREDSRVRAVGMNGSRTNP-------NVPKDPFRDYDIVYVVTDMQ 53

CHCC14525_3596 MRTEQEIIDLVLKVAREDSRVRAVGMNGSRTNP-------NVPKDPFRDYDIVYVVTDMQ 53

CHCC14441_1578 MRTEQEIIDLVLKVAREDSRVRAVGMNGSRTNP-------NVPKDPFRDYDIVYVVTDMQ 53

CHCC15311_0626 MRTEQEIIDLVLKVAREDSRVRAVGMNGSRTNP-------NVPKDPFRDYDIVYVVTDMQ 53

CHCC15315_4193 MRTEQEIIDLVLKVAREDSRVRAVGMNGSRTNP-------NVPKDPFRDYDIVYVVTDMQ 53

CHCC20487_3824 MRTEQEIIDLVLKVAREDSRVRAVGMNGSRTNP-------NVPKDPFRDYDIVYVVTDMQ 53

CHCC20486_2040 MRTEQEIIDLVLKVAREDSRVRAVGMNGSRTNP-------NVPKDPFRDYDIVYVVTDMQ 53

CHCC15318_3693 MRTEQEIIDLVLKVAREDSRVRAVGMNGSRTNP-------NVPKDPFRDYDIVYVVTDMQ 53

CHCC20323_0096 MRTEQEIIDLVLKVAREDSRVRAVGMNGSRTNP-------NVPKDPFRDYDIVYVVTDMQ 53

CHCC20325_3729 MRTEQEIIDLVLKVAREDSRVRAVGMNGSRTNP-------NVPKDPFRDYDIVYVVTDMQ 53

CHCC15087_0205 MRTEQEIIDLVLKVAREDSRVRAVGMNGSRTNP-------NVPKDPFRDYDIVYVVTDMQ 53

CHCC14437_0388 MRTEQEIIDLVLKVAREDSRVRAVGMNGSRTNP-------NVPKDPFRDYDIVYVVTDMQ 53

CHCC14431_3647 MRTEQEIIDLVLKVAREDSRVRAVGMNGSRTNP-------NVPKDPFRDYDIVYVVTDMQ 53

CHCC14566_2259 MRTEQEIIDLVLKVAREDSRVRAVGMNGSRTNP-------NVPKDPFRDYDIVYVVTDMQ 53

CHCC20493_0357 MRTEQEIIDLVLKVAREDSRVRAVGMNGSRTNP-------NVPKDPFRDYDIVYVVTDMQ 53

CHCC20494_1491 MRTEQEIIDLVLKVAREDSRVRAVGMNGSRTNP-------NVPKDPFRDYDIVYVVTDMQ 53

CHCC20495_0876 MRTEQEIIDLVLKVAREDSRVRAVGMNGSRTNP-------NVPKDPFRDYDIVYVVTDMQ 53

CHCC16736_1642 MRTEQEIIDLVLKVAREDSRVRAVGMNGSRTNP-------NVPKDPFRDYDIVYVVTDMQ 53

CHCC19466_3945 MRTEQEIIDLVLKVAREDSRVRAVGMNGSRTNP-------NVPKDPFRDYDIVYVVTDMQ 53

CHCC15543_3600 MRTEQEIIDLVLKVAREDSRVRAVGMNGSRTNP-------NVPKDPFRDYDIVYVVTDMQ 53

CHCC15546_0033 MRTEQEIIDLVLKVAREDSRVRAVGMNGSRTNP-------NVPKDPFRDYDIVYVVTDMQ 53

CHCC10893_2818 MRTEQEIIDLVLKVAREDSRVRAVGMNGSRTNP-------NVPKDPFRDYDIVYVVTDMQ 53

CHCC16874_4694 MRTEQEIIDLVLKVAREDSRVRAVGMNGSRTNPNVPRTNPNVPKDPFRDYDIVYVVTDMQ 60

CHCC15091_2903 MRTEQEIIDLVLKVAREDSRVRAVGMNGSRTNPNVPRTNPNVPKDPFRDYDIVYVVTDMQ 60

CHCC14429_3013 MRTEQEIIDLVLKVAREDSRVRAVGMNGSRTNPNVPRTNPNVPKDPFRDYDIVYVVTDMQ 60

CHCC20496_3908 MRTEQEIIDLVLKVAREDSRVRAVGMNGSRTNPNVPRTNPNVPKDPFRDYDIVYVVTDMQ 60

CHCC14559_1463 MRTEQEIIDLVLKVAREDSRVRAVGMNGSRTNPNVPRTNPNVPKDPFRDYDIVYVVTDMQ 60

CHCC14557_3890 MRTEQEIIDLVLKVAREDSRVRAVGMNGSRTNPNVPRTNPNVPKDPFRDYDIVYVVTDMQ 60

CHCC14435_2912 MRTEQEIIDLVLKVAREDSRVRAVGMNGSRTNPNVPRTNPNVPKDPFRDYDIVYVVTDMQ 60

CHCC20489_0347 MRTEQEIIDLVLKVAREDSRVRAVGMNGSRTNPNVPRTNPNVPKDPFRDYDIVYVVTDMQ 60

CHCC14568_0477 MRTEQEIIDLVLKVAREDSRVRAVGMNGSRTNPNVPRTNPNVPKDPFRDYDIVYVVTDMQ 60

CHCC20339_0023 MRTEQEIIDLVLKVAREDSRVRAVGMNGSRTNPNVPRTNPNVPKDPFRDYDIVYVVTDMQ 60

CHCC15325_0415 MRTEQEIIDLVLKVAREDSRVRAVGMNGSRTNPNVPRTNPNVPKDPFRDYDIVYVVTDMQ 60

CHCC20345_4264 MRTEQEIIDLVLKVAREDSRVRAVGMNGSRTNPNVPRTNPNVPKDPFRDYDIVYVVTDMQ 60

CHCC14688_3358 MRTEQEIIDLVLKVAREDSRVRAVGMNGSRTNPNVPRTNPNVPKDPFRDYDIVYVVTDMQ 60

CHCC5025_0548 MRTEQEIIDLVLKVAREDSRVRAVGMNGSRTNPNVPRTNPNVPKDPFRDYDIVYVVTDMQ 60

CHCC14816_0249 MRTEQEIIDLVLKVAREDSRVRAVGMNGSRTNPNVPRTNPNVPKDPFRDYDIVYVVTDMQ 60

CHCC14815_0971 MRTEQEIIDLVLKVAREDSRVRAVGMNGSRTNPNVPRTNPNVPKDPFRDYDIVYVVTDMQ 60

CHCC14818_3872 MRTEQEIIDLVLKVAREDSRVRAVGMNGSRTNPNVPRTNPNVPKDPFRDYDIVYVVTDMQ 60

CHCC15292_0697 MRTEQEIIDLVLKVAREDSRVRAVGMNGSRTNPNVPRTNPNVPKDPFRDYDIVYVVTDMQ 60

CHCC14562_0762 MRTEQEIIDLVLKVAREDSRVRAVGMNGSRTNPNVPRTNPNVPKDPFRDYDIVYVVTDMQ 60

CHCC14561_2620 MRTEQEIIDLVLKVAREDSRVRAVGMNGSRTNPNVPRTNPNVPKDPFRDYDIVYVVTDMQ 60

DSM13_0192 MRTEQEIIDLVLKVAREDSRVRAVGMNGSRTNPNVPRTNPNVPKDPFRDYDIVYVVTDMQ 60

CHCC15075_1356 MRTEQEIIDLVLKVAREDSRVRAVGMNGSRTNPNVPRTNPNVPKDPFRDYDIVYVVTDMQ 60

CHCC20327_1735 MRTEQEIIDLVLKVAREDSRVRAVGMNGSRTNP-------NVPKDPFRDYDIVYVVTDMQ 53

CHCC14814_3092 MRTEQEIIDLVLKVAREDSRVRAVGMNGSRTNP-------NVPKDPFRDYDMVYVVTDMQ 53

CHCC20375_3122 MRTEQEIIDLVLKIAREDSRVRAVGMNGSRTNP-------NVPKDPFRDYDMVYVVTDMQ 53

*************:****.************** ***********:*******:

CHCC15381_2836 SFLDEPGWVDVFGERIIMQTPEAMELFPNELGNRFSYLMLFTDGSRIDLILVPLEEKLEY 113

CHCC12620_0739 SFLDEPGWVDVFGERIIMQTPEAMELFPNELGNRFSYLMLFTDGSRIDLILVPLEEKLEY 113

CHCC19467_4580 SFLDEPGWVDVFGERIIMQTPEAMELFPNELGNRFSYLMLFTDGSRIDLILVPLEEKLEY 113

CHCC19468_2703 SFLDEPGWVDVFGERIIMQTPEAMELFPNELGNRFSYLMLFTDGSRIDLILVPLEEKLEY 113

CHCC20497_4637 SFLDEPGWVDVFGERIIMQTPEAMELFPNELGNRFSYLMLFTDGSRIDLILVPLEEKLEY 113

CHCC20491_0559 SFLDEPGWVDVFGERIIMQTPEAMELFPNELGNRFSYLMLFTDGSRIDLILVPLEEKLEY 113

CHCC20492_2745 SFLDEPGWVDVFGERIIMQTPEAMELFPNELGNRFSYLMLFTDGSRIDLILVPLEEKLEY 113

CHCC14523_3914 SFLDEPGWVDVFGERIIMQTPEAMELFPNELGNRFSYLMLFTDGSRIDLILVPLEEKLEY 113

CHCC14527_2372 SFLDEPGWVDVFGERIIMQTPEAMELFPNELGNRFSYLMLFTDGSRIDLILVPLEEKLEY 113

ATCC9945A_0220 SFLDEPGWVDVFGERIIMQTPEAMELFPNELGNRFSYLMLFTDGSRIDLILVPLEEKLEY 113

CHCC15136_3271 SFLDEPGWVDVFGERIIMQTPEAMELFPNELGNRFSYLMLFTDGSRIDLILVPLEEKLEY 113

CHCC5019_4456 SFLDEPGWVDVFGERIIMQTPEAMELFPNELGNRFSYLMLFTDGSRIDLILVPLEEKLEY 113

CHCC15337_4610 SFLDEPGWVDVFGERIIMQTPEAMELFPNELGNRFSYLMLFTDGSRIDLILVPLEEKLEY 113

CHCC15332_3737 SFLDEPGWVDVFGERIIMQTPEAMELFPNELGNRFSYLMLFTDGSRIDLILVPLEEKLEY 113

CHCC5022_3897 SFLDEPGWVDVFGERIIMQTPEAMELFPNELGNRFSYLMLFTDGSRIDLILVPLEEKLEY 113

CHCC5023_1979 SFLDEPGWVDVFGERIIMQTPEAMELFPNELGNRFSYLMLFTDGSRIDLILVPLEEKLEY 113

CHCC5021_2115 SFLDEPGWVDVFGERIIMQTPEAMELFPNELGNRFSYLMLFTDGSRIDLILVPLEEKLEY 113

CHCC14817_1577 SFLDEPGWVDVFGERIIMQTPEAMELFPNELGNRFSYLMLFTDGSRIDLILVPLEEKLEY 113

CHCC4186_2037 SFLDEPGWVDVFGERIIMQTPEAMELFPNELGNRFSYLMLFTDGSRIDLILVPLEEKLEY 113

CHCC20488_0914 SFLDEPGWVDVFGERIIMQTPEAMELFPNELGNRFSYLMLFTDGSRIDLILVPLEEKLEY 113

BL09_0219 SFLDEPGWVDVFGERIIMQTPEAMELFPNELGNRFSYLMLFTDGSRIDLILVPLEEKLEY 113

CHCC20331_1437 SFLDEPGWVDVFGERIIMQTPEAMELFPNELGNRFSYLMLFTDGSRIDLILVPLEEKLEY 113

CHCC20347_3231 SFLDEPGWVDVFGERIIMQTPEAMELFPNELGNRFSYLMLFTDGSRIDLILVPLEEKLEY 113

CHCC20333_3258 SFLDEPGWVDVFGERIIMQTPEAMELFPNELGNRFSYLMLFTDGSRIDLILVPLEEKLEY 113

CHCC20490_1916 SFLDEPGWVDVFGERIIMQTPEAMELFPNELGNRFSYLMLFTDGSRIDLILVPLEEKLEY 113

CHCC20348_4391 SFLDEPGWVDVFGERIIMQTPEAMELFPNELGNRFSYLMLFTDGSRIDLILVPLEEKLEY 113

CHCC5027_3368 SFLDEPGWVDVFGERIIMQTPEAMELFPNELGNRFSYLMLFTDGSRIDLILVPLEEKLEY 113

CHCC14820_1469 SFLDEPGWVDVFGERIIMQTPEAMELFPNELGNRFSYLMLFTDGSRIDLILVPLEEKLEY 113

CHCC20372_2718 SFLDEPGWVDVFGERIIMQTPEAMELFPNELGNRFSYLMLFTDGSRIDLILVPLEEKLEY 113

CHCC20373_0250 SFLDEPGWVDVFGERIIMQTPEAMELFPNELGNRFSYLMLFTDGSRIDLILVPLEEKLEY 113

CHCC15289_3953 SFLDEPGWVDVFGERIIMQTPEAMELFPNELGNRFSYLMLFTDGSRIDLILVPLEEKLEY 113

CHCC14564_2767 SFLDEPGWVDVFGERIIMQTPEAMELFPNELGNRFSYLMLFTDGSRIDLILVPLEEKLEY 113

CHCC15290_2999 SFLDEPGWVDVFGERIIMQTPEAMELFPNELGNRFSYLMLFTDGSRIDLILVPLEEKLEY 113

CHCC20368_2019 SFLDEPGWVDVFGERIIMQTPEAMELFPNELGNRFSYLMLFTDGSRIDLILVPLEEKLEY 113

CHCC15291_0394 SFLDEPGWVDVFGERIIMQTPEAMELFPNELGNRFSYLMLFTDGSRIDLILVPLEEKLEY 113

CHCC20369_2215 SFLDEPGWVDVFGERIIMQTPEAMELFPNELGNRFSYLMLFTDGSRIDLILVPLEEKLEY 113

CHCC14598_1058 SFLDEPGWVDVFGERIIMQTPEAMELFPNELGNRFSYLMLFTDGSRIDLILVPLEEKLEY 113

CHCC14596_0534 SFLDEPGWVDVFGERIIMQTPEAMELFPNELGNRFSYLMLFTDGSRIDLILVPLEEKLEY 113

CHCC14810_4312 SFLDEPGWVDVFGERIIMQTPEAMELFPNELGNRFSYLMLFTDGSRIDLILVPLEEKLEY 113

CHCC14819_0399 SFLDEPGWVDVFGERIIMQTPEAMELFPNELGNRFSYLMLFTDGSRIDLILVPLEEKLEY 113

CHCC14813_2856 SFLDEPGWVDVFGERIIMQTPEAMELFPNELGNRFSYLMLFTDGSRIDLILVPLEEKLEY 113

CHCC20441_1080 SFLDEPGWVDVFGERIIMQTPEAMELFPNELGNRFSYLMLFTDGSRIDLILVPLEEKLEY 113

CHCC20440_1830 SFLDEPGWVDVFGERIIMQTPEAMELFPNELGNRFSYLMLFTDGSRIDLILVPLEEKLEY 113

CHCC20442_1308 SFLDEPGWVDVFGERIIMQTPEAMELFPNELGNRFSYLMLFTDGSRIDLILVPLEEKLEY 113

CHCC5024_4269 SFLDEPGWVDVFGERIIMQTPEAMELFPNELGNRFSYLMLFTDGSRIDLILVPLEEKLEY 113

CHCC5026_1706 SFLDEPGWVDVFGERIIMQTPEAMELFPNELGNRFSYLMLFTDGSRIDLILVPLEEKLEY 113

CHCC5020_0926 SFLDEPGWVDVFGERIIMQTPEAMELFPNELGNRFSYLMLFTDGSRIDLILVPLEEKLEY 113

CHCC14600_3227 SFLDEPGWVDVFGERIIMQTPEAMELFPNELGNRFSYLMLFTDGSRIDLILVPLEEKLEY 113

CHCC14808_3671 SFLDEPGWVDVFGERIIMQTPEAMELFPNELGNRFSYLMLFTDGSRIDLILVPLEEKLEY 113

CHCC14809_3060 SFLDEPGWVDVFGERIIMQTPEAMELFPNELGNRFSYLMLFTDGSRIDLILVPLEEKLEY 113

CHCC15335_1764 SFLDEPGWVDVFGERIIMQTPEAMELFPNELGNRFSYLMLFTDGSRIDLILVPLEEKLEY 113

CHCC20342_1142 SFLDEPGWVDVFGERIIMQTPEAMELFPNELGNRFSYLMLFTDGSRIDLILVPLEEKLEY 113

CHCC20343_0033 SFLDEPGWVDVFGERIIMQTPEAMELFPNELGNRFSYLMLFTDGSRIDLILVPLEEKLEY 113

CHCC20341_2338 SFLDEPGWVDVFGERIIMQTPEAMELFPNELGNRFSYLMLFTDGSRIDLILVPLEEKLEY 113

CHCC20344_4475 SFLDEPGWVDVFGERIIMQTPEAMELFPNELGNRFSYLMLFTDGSRIDLILVPLEEKLEY 113

CHCC15139_1359 SFLDEPGWVDVFGERIIMQTPEAMELFPNELGNRFSYLMLFTDGSRIDLILVPLEEKLEY 113

CHCC15320_2309 SFLDEPGWVDVFGERIIMQTPEAMELFPNELGNRFSYLMLFTDGSRIDLILVPLEEKLEY 113

CHCC15322_3643 SFLDEPGWVDVFGERIIMQTPEAMELFPNELGNRFSYLMLFTDGSRIDLILVPLEEKLEY 113

CHCC14525_3596 SFLDEPGWVDVFGERIIMQTPEAMELFPNELGNRFSYLMLFTDGSRIDLILVPLEEKLEY 113

CHCC14441_1578 SFLDEPGWVDVFGERIIMQTPEAMELFPNELGNRFSYLMLFTDGSRIDLILVPLEEKLEY 113

CHCC15311_0626 SFLDEPGWVDVFGERIIMQTPEAMELFPNELGNRFSYLMLFTDGSRIDLILVPLEEKLEY 113

CHCC15315_4193 SFLDEPGWVDVFGERIIMQTPEAMELFPNELGNRFSYLMLFTDGSRIDLILVPLEEKLEY 113

CHCC20487_3824 SFLDEPGWVDVFGERIIMQTPEAMELFPNELGNRFSYLMLFTDGSRIDLILVPLEEKLEY 113

CHCC20486_2040 SFLDEPGWVDVFGERIIMQTPEAMELFPNELGNRFSYLMLFTDGSRIDLILVPLEEKLEY 113

CHCC15318_3693 SFLDEPGWVDVFGERIIMQTPEAMELFPNELGNRFSYLMLFTDGSRIDLILVPLEEKLEY 113

CHCC20323_0096 SFLDEPGWVDVFGERIIMQTPEAMELFPNELGNRFSYLMLFTDGSRIDLILVPLEEKLEY 113

CHCC20325_3729 SFLDEPGWVDVFGERIIMQTPEAMELFPNELGNRFSYLMLFTDGSRIDLILVPLEEKLEY 113

CHCC15087_0205 SFLDEPGWVDVFGERIIMQTPEAMELFPNELGNRFSYLMLFTDGSRIDLILVPLEEKLEY 113

CHCC14437_0388 SFLDEPGWVDVFGERIIMQTPEAMELFPNELGNRFSYLMLFTDGSRIDLILVPLEEKLEY 113

CHCC14431_3647 SFLDEPGWVDVFGERIIMQTPEAMELFPNELGNRFSYLMLFTDGSRIDLILVPLEEKLEY 113

CHCC14566_2259 SFLDEPGWVDVFGERIIMQTPEAMELFPNELGNRFSYLMLFTDGSRIDLILVPLEEKLEY 113

CHCC20493_0357 SFLDEPGWVDVFGERIIMQTPEAMELFPNELGNRFSYLMLFTDGSRIDLILVPLEEKLEY 113

CHCC20494_1491 SFLDEPGWVDVFGERIIMQTPEAMELFPNELGNRFSYLMLFTDGSRIDLILVPLEEKLEY 113

CHCC20495_0876 SFLDEPGWVDVFGERIIMQTPEAMELFPNELGNRFSYLMLFTDGSRIDLILVPLEEKLEY 113

CHCC16736_1642 SFLDEPGWVDVFGERIIMQTPEAMELFPNELGNRFSYLMLFTDGSRIDLILVPLEEKLEY 113

CHCC19466_3945 SFLDEPGWVDVFGERIIMQTPEAMELFPNELGNRFSYLMLFTDGSRIDLILVPLEEKLEY 113

CHCC15543_3600 SFLDEPGWVDVFGERIIMQTPEAMELFPNELGNRFSYLMLFTDGSRIDLILVPLEEKLEY 113

CHCC15546_0033 SFLDEPGWVDVFGERIIMQTPEAMELFPNELGNRFSYLMLFTDGSRIDLILVPLEEKLEY 113

CHCC10893_2818 SFLDEPGWVDVFGERIIMQTPEAMELFPNELGNRFSYLMLFTDGSRIDLILVPLEEKLEY 113

CHCC16874_4694 SFLDEPGWVDVFGERIIMQTPEAMELFPNELGNRFSYLMLFTDGSRIDLILVPLEEKLEY 120

CHCC15091_2903 SFLDEPGWVDVFGERIIMQTPEAMELFPNELGNRFSYLMLFTDGSRIDLILVPLEEKLEY 120

CHCC14429_3013 SFLDEPGWVDVFGERIIMQTPEAMELFPNELGNRFSYLMLFTDGSRIDLILVPLEEKLEY 120

CHCC20496_3908 SFLDEPGWVDVFGERIIMQTPEAMELFPNELGNRFSYLMLFTDGSRIDLILVPLEEKLEY 120

CHCC14559_1463 SFLDEPGWVDVFGERIIMQTPEAMELFPNELGNRFSYLMLFTDGSRIDLILVPLEEKLEY 120

CHCC14557_3890 SFLDEPGWVDVFGERIIMQTPEAMELFPNELGNRFSYLMLFTDGSRIDLILVPLEEKLEY 120

CHCC14435_2912 SFLDEPGWVDVFGERIIMQTPEAMELFPNELGNRFSYLMLFTDGSRIDLILVPLEEKLEY 120

CHCC20489_0347 SFLDEPGWVDVFGERIIMQTPEAMELFPNELGNRFSYLMLFTDGSRIDLILVPLEEKLEY 120

CHCC14568_0477 SFLDEPGWVDVFGERIIMQTPEAMELFPNELGNRFSYLMLFTDGSRIDLILVPLEEKLEY 120

CHCC20339_0023 SFLDEPGWVDVFGERIIMQTPEAMELFPNELGNRFSYLMLFTDGSRIDLILVPLEEKLEY 120

CHCC15325_0415 SFLDEPGWVDVFGERIIMQTPEAMELFPNELGNRFSYLMLFTDGSRIDLILVPLEEKLEY 120

CHCC20345_4264 SFLDEPGWVDVFGERIIMQTPEAMELFPNELGNRFSYLMLFTDGSRIDLILVPLEEKLEY 120

CHCC14688_3358 SFLDEPGWVDVFGERIIMQTPEAMELFPNELGNRFSYLMLFTDGSRIDLILVPLEEKLEY 120

CHCC5025_0548 SFLDEPGWVDVFGERIIMQTPEAMELFPNELGNRFSYLMLFTDGSRIDLILVPLEEKLEY 120

CHCC14816_0249 SFLDEPGWVDVFGERIIMQTPEAMELFPNELGNRFSYLMLFTDGSRIDLILVPLEEKLEY 120

CHCC14815_0971 SFLDEPGWVDVFGERIIMQTPEAMELFPNELGNRFSYLMLFTDGSRIDLILVPLEEKLEY 120

CHCC14818_3872 SFLDEPGWVDVFGERIIMQTPEAMELFPNELGNRFSYLMLFTDGSRIDLILVPLEEKLEY 120

CHCC15292_0697 SFLDEPGWVDVFGERIIMQTPEAMELFPNELGNRFSYLMLFTDGSRIDLILVPLEEKLEY 120

CHCC14562_0762 SFLDEPGWVDVFGERIIMQTPEAMELFPNELGNRFSYLMLFTDGSRIDLILVPLEEKLEY 120

CHCC14561_2620 SFLDEPGWVDVFGERIIMQTPEAMELFPNELGNRFSYLMLFTDGSRIDLILVPLEEKLEY 120

DSM13_0192 SFLDEPGWVDVFGERIIMQTPEAMELFPNELGNRFSYLMLFTDGSRIDLILVPLEEKLEY 120

CHCC15075_1356 SFLDEPGWVDVFGERIIMQTPEAMELFPNELGNRFSYLMLFTDGSRIDLILVPLEEKLEY 120

CHCC20327_1735 SFLDEPGWVDVFGERIIMQTPKAMELFPNELGNRFSYLMLFTDGSRIDLILVPLEEKLEY 113

CHCC14814_3092 SFLDEPGWVDVFGERIIMQTPEAMELFPNELGNRFSYLMLFKDGSRIDLILVPLEEKLEY 113

CHCC20375_3122 SFLDEPGWVDVFGERIIMQTPEAMELFPNELGNRFSYLMLFTDGSRIDLILVPLEEKLEY 113

*********************:*******************.******************

CHCC15381_2836 CREDGLTVILLDKDQDLPVIPPPTDREYWVQKPSPQCFADCCNEFWWTSTYVAKGLWRQE 173

CHCC12620_0739 CREDGLTVILLDKDQDLPVIPPPTDREYWVQKPSPQCFADCCNEFWWTSTYVAKGLWRQE 173

CHCC19467_4580 CREDGLTVILLDKDQDLPVIPPPTDREYWVQKPSPQCFADCCNEFWWTSTYVAKGLWRQE 173

CHCC19468_2703 CREDGLTVILLDKDQDLPVIPPPTDREYWVQKPSPQCFADCCNEFWWTSTYVAKGLWRQE 173

CHCC20497_4637 CREDGLTVILLDKDQDLPVIPPPTDREYWVQKPSPQCFADCCNEFWWTSTYVAKGLWRQE 173

CHCC20491_0559 CREDGLTVILLDKDQDLPVIPPPTDREYWVQKPSPQCFADCCNEFWWTSTYVAKGLWRQE 173

CHCC20492_2745 CREDGLTVILLDKDQDLPVIPPPTDREYWVQKPSPQCFADCCNEFWWTSTYVAKGLWRQE 173

CHCC14523_3914 CREDGLTVILLDKDQDLPVIPPPTDREYWVQKPSPQCFADCCNEFWWTSTYVAKGLWRQE 173

CHCC14527_2372 CREDGLTVILLDKDQDLPVIPPPTDREYWVQKPSPQCFADCCNEFWWTSTYVAKGLWRQE 173

ATCC9945A_0220 CREDGLTVILLDKDQDLPVIPPPTDREYWVQKPSPQCFADCCNEFWWTSTYVAKGLWRQE 173

CHCC15136_3271 CREDGLTVILLDKDQDLPVIPPPTDREYWVQKPSPQCFADCCNEFWWTSTYVAKGLWRQE 173

CHCC5019_4456 CREDGLTVILLDKDQDLPVIPPPTDREYWVQKPSPQCFADCCNEFWWTSTYVAKGLWRQE 173

CHCC15337_4610 CREDGLTVILLDKDQDLPVIPPPTDREYWVQKPSPQCFADCCNEFWWTSTYVAKGLWRQE 173

CHCC15332_3737 CREDGLTVILLDKDQDLPVIPPPTDREYWVQKPSPQCFADCCNEFWWTSTYVAKGLWRQE 173

CHCC5022_3897 CREDGLTVILLDKDQDLPVIPPPTDREYWVQKPSPQCFADCCNEFWWTSTYVAKGLWRQE 173

CHCC5023_1979 CREDGLTVILLDKDQDLPVIPPPTDREYWVQKPSPQCFADCCNEFWWTSTYVAKGLWRQE 173

CHCC5021_2115 CREDGLTVILLDKDQDLPVIPPPTDREYWVQKPSPQCFADCCNEFWWTSTYVAKGLWRQE 173

CHCC14817_1577 CREDGLTVILLDKDQDLPVIPPPTDREYWVQKPSPQCFADCCNEFWWTSTYVAKGLWRQE 173

CHCC4186_2037 CREDGLTVILLDKDQDLPVIPPPTDREYWVQKPSPQCFADCCNEFWWTSTYVAKGLWRQE 173

CHCC20488_0914 CREDGLTVILLDKDQDLPVIPPPTDREYWVQKPSPQCFADCCNEFWWTSTYVAKGLWRQE 173

BL09_0219 CREDGLTVILLDKDQDLPVIPPPTDREYWVQKPSPQCFADCCNEFWWTSTYVAKGLWRQE 173

CHCC20331_1437 CREDGLTVILLDKDQDLPVIPPPTDREYWVQKPSPQCFADCCNEFWWTSTYVAKGLWRQE 173

CHCC20347_3231 CREDGLTVILLDKDQDLPVIPPPTDREYWVQKPSPQCFADCCNEFWWTSTYVAKGLWRQE 173

CHCC20333_3258 CREDGLTVILLDKDQDLPVIPPPTDREYWVQKPSPQCFADCCNEFWWTSTYVAKGLWRQE 173

CHCC20490_1916 CREDGLTVILLDKDQDLPVIPPPTDREYWVQKPSPQCFADCCNEFWWTSTYVAKGLWRQE 173

CHCC20348_4391 CREDGLTVILLDKDQDLPVIPPPTDREYWVQKPSPQCFADCCNEFWWTSTYVAKGLWRQE 173

CHCC5027_3368 CREDGLTVILLDKDQDLPVIPPPTDREYWVQKPSPQCFADCCNEFWWTSTYVAKGLWRQE 173

CHCC14820_1469 CREDGLTVILLDKDQDLPVIPPPTDREYWVQKPSPQCFADCCNEFWWTSTYVAKGLWRQE 173

CHCC20372_2718 CREDGLTVILLDKDQDLPVIPPPTDREYWVQKPSPQCFADCCNEFWWTSTYVAKGLWRQE 173

CHCC20373_0250 SREDGLTVILLDKDQDLPVIPPPTDREYWVQKPSPQCFADCCNEFWWTSTYVAKGLWRQE 173

CHCC15289_3953 SREDGLTVILLDKDQDLPVIPPPTDREYWVQKPSPQCFADCCNEFWWTSTYVAKGLWRQE 173

CHCC14564_2767 SREDGLTVILLDKDQDLPVIPPPTDREYWVQKPSPQCFADCCNEFWWTSTYVAKGLWRQE 173

CHCC15290_2999 SREDGLTVILLDKDQDLPVIPPPTDREYWVQKPSPQCFADCCNEFWWTSTYVAKGLWRQE 173

CHCC20368_2019 SREDGLTVILLDKDQDLPVIPPPTDREYWVQKPSPQCFADCCNEFWWTSTYVAKGLWRQE 173

CHCC15291_0394 SREDGLTVILLDKDQDLPVIPPPTDREYWVQKPSPQCFADCCNEFWWTSTYVAKGLWRQE 173

CHCC20369_2215 SREDGLTVILLDKDQDLPVIPPPTDREYWVQKPSPQCFADCCNEFWWTSTYVAKGLWRQE 173

CHCC14598_1058 SREDGLTVILLDKDQDLPVIPPPTDREYWVQKPSPQCFADCCNEFWWTSTYVAKGLWRQE 173

CHCC14596_0534 SREDGLTVILLDKDQDLPVIPPPTDREYWVQKPSPQCFADCCNEFWWTSTYVAKGLWRQE 173

CHCC14810_4312 SREDGLTVILLDKDQDLPVIPPPTDREYWVQKPSPQCFADCCNEFWWTSTYVAKGLWRQE 173

CHCC14819_0399 SREDGLTVILLDKDQDLPVIPPPTDREYWVQKPSPQCFADCCNEFWWTSTYVAKGLWRQE 173

CHCC14813_2856 SREDGLTVILLDKDQDLPVIPPPTDREYWVQKPSPQCFADCCNEFWWTSTYVAKGLWRQE 173

CHCC20441_1080 SREDGLTVILLDKDQDLPVIPPPTDREYWVQKPSPQCFADCCNEFWWTSTYVAKGLWRQE 173

CHCC20440_1830 SREDGLTVILLDKDQDLPVIPPPTDREYWVQKPSPQCFADCCNEFWWTSTYVAKGLWRQE 173

CHCC20442_1308 SREDGLTVILLDKDQDLPVIPPPTDREYWVQKPSPQCFADCCNEFWWTSTYVAKGLWRQE 173

CHCC5024_4269 SREDGLTVILLDKDQDLPVIPPPTDREYWVQKPSPQCFADCCNEFWWTSTYVAKGLWRQE 173

CHCC5026_1706 SREDGLTVILLDKDQDLPVIPPPTDREYWVQKPSPQCFADCCNEFWWTSTYVAKGLWRQE 173

CHCC5020_0926 SREDGLTVILLDKDQDLPVIPPPTDREYWVQKPSPQCFADCCNEFWWTSTYVAKGLWRQE 173

CHCC14600_3227 SREDGLTVILLDKDQDLPVIPPPTDREYWVQKPSPQCFADCCNEFWWTSTYVAKGLWRQE 173

CHCC14808_3671 SREDGLTVILLDKDQDLPVIPPPTDREYWVQKPSPQCFADCCNEFWWTSTYVAKGLWRQE 173

CHCC14809_3060 SREDGLTVILLDKDQDLPVIPPPTDREYWVQKPSPQCFADCCNEFWWTSTYVAKGLWRQE 173

CHCC15335_1764 SREDGLTVILLDKDQDLPVIPPPTDREYWVQKPSPQCFADCCNEFWWTSTYVAKGLWRQE 173

CHCC20342_1142 SREDGLTVILLDKDQDLPVIPPPTDREYWVQKPSPQCFADCCNEFWWTSTYVAKGLWRQE 173

CHCC20343_0033 SREDGLTVILLDKDQDLPVIPPPTDREYWVQKPSPQCFADCCNEFWWTSTYVAKGLWRQE 173

CHCC20341_2338 SREDGLTVILLDKDQDLPVIPPPTDREYWVQKPSPQCFADCCNEFWWTSTYVAKGLWRQE 173

CHCC20344_4475 SREDGLTVILLDKDQDLPVIPPPTDREYWVQKPSPQCFADCCNEFWWTSTYVAKGLWRQE 173

CHCC15139_1359 SREDGLTVILLDKDQDLPVIPPPTDREYWVQKPSPQCFADCCNEFWWTSTYVAKGLWRQE 173

CHCC15320_2309 SREDGLTVILLDKDQDLPVIPPPTDREYWVQKPSPQCFADCCNEFWWTSTYVAKGLWRQE 173

CHCC15322_3643 SREDGLTVILLDKDQDLPVIPPPTDREYWVQKPSPQCFADCCNEFWWTSTYVAKGLWRQE 173

CHCC14525_3596 SREDGLTVILLDKDQDLPVIPPPTDREYWVQKPSPQCFADCCNEFWWTSTYVAKGLWRQE 173

CHCC14441_1578 SREDGLTVILLDKDQDLPVIPPPTDREYWVQKPSPQCFADCCNEFWWTSTYVAKGLWRQE 173

CHCC15311_0626 SREDGLTVILLDKDQDLPVIPPPTDREYWVQKPSPQCFADCCNEFWWTSTYVAKGLWRQE 173

CHCC15315_4193 SREDGLTVILLDKDQDLPVIPPPTDREYWVQKPSPQCFADCCNEFWWTSTYVAKGLWRQE 173

CHCC20487_3824 SREDGLTVILLDKDQDLPVIPPPTDREYWVQKPSPQCFADCCNEFWWTSTYVAKGLWRQE 173

CHCC20486_2040 SREDGLTVILLDKDQDLPVIPPPTDREYWVQKPSPQCFADCCNEFWWTSTYVAKGLWRQE 173

CHCC15318_3693 SREDGLTVILLDKDQDLPVIPPPTDREYWVQKPSPQCFADCCNEFWWTSTYVAKGLWRQE 173

CHCC20323_0096 SREDGLTVILLDKDQDLPVIPPPTDREYWVQKPSPQCFADCCNEFWWTSTYVAKGLWRQE 173

CHCC20325_3729 SREDGLTVILLDKDQDLPVIPPPTDREYWVQKPSPQCFADCCNEFWWTSTYVAKGLWRQE 173

CHCC15087_0205 SREDGLTVILLDKDQDLPVIPPPTDREYWVQKPSPQCFADCCNEFWWTSTYVAKGLWRQE 173

CHCC14437_0388 SREDGLTVILLDKDQDLPVIPPPTDREYWVQKPSPQCFADCCNEFWWTSTYVAKGLWRQE 173

CHCC14431_3647 SREDGLTVILLDKDQDLPVIPPPTDREYWVQKPSPQCFADCCNEFWWTSTYVAKGLWRQE 173

CHCC14566_2259 SREDGLTVILLDKDQDLPVIPPPTDREYWVQKPSPQCFADCCNEFWWTSTYVAKGLWRQE 173

CHCC20493_0357 SREDGLTVILLDKDQDLPVIPPPTDREYWVQKPSPQCFADCCNEFWWTSTYVAKGLWRQE 173

CHCC20494_1491 SREDGLTVILLDKDQDLPVIPPPTDREYWVQKPSPQCFADCCNEFWWTSTYVAKGLWRQE 173

CHCC20495_0876 SREDGLTVILLDKDQDLPVIPPPTDREYWVQKPSPQCFADCCNEFWWTSTYVAKGLWRQE 173

CHCC16736_1642 SREDGLTVILLDKDQDLPVIPPPTDREYWVQKPSPQCFADCCNEFWWTSTYVAKGLWRQE 173

CHCC19466_3945 SREDGLTVILLDKDQDLPVIPPPTDREYWVQKPSPQCFADCCNEFWWTSTYVAKGLWRQE 173

CHCC15543_3600 SREDGLTVILLDKDQDLPVIPPPTDREYWVQKPSPQCFADCCNEFWWTSTYVAKGLWRQE 173

CHCC15546_0033 SREDGLTVILLDKDQDLPVIPPPTDREYWVQKPSPQCFADCCNEFWWTSTYVAKGLWRQE 173

CHCC10893_2818 SREDGLTVILLDKDQDLPVIPPPTDREYWVQKPSPQCFADCCNEFWWTSTYVAKGLWRQE 173

CHCC16874_4694 SREDGLTVILLDKDQDLPVIPPPTDREYWVQKPSPQCFADCCNEFWWTSTYVAKGLWRQE 180

CHCC15091_2903 SREDGLTVILLDKDQDLPVIPPPTDREYWVQKPSPQCFADCCNEFWWTSTYVAKGLWRQE 180

CHCC14429_3013 SREDGLTVILLDKDQDLPVIPPPTDREYWVQKPSPQCFADCCNEFWWTSTYVAKGLWRQE 180

CHCC20496_3908 SREDGLTVILLDKDQDLPVIPPPTDREYWVQKPSPQCFADCCNEFWWTSTYVAKGLWRQE 180

CHCC14559_1463 SREDGLTVILLDKDQDLPVIPPPTDREYWVQKPSPQCFADCCNEFWWTSTYVAKGLWRQE 180

CHCC14557_3890 SREDGLTVILLDKDQDLPVIPPPTDREYWVQKPSPQCFADCCNEFWWTSTYVAKGLWRQE 180

CHCC14435_2912 SREDGLTVILLDKDQDLPVIPPPTDREYWVQKPSPQCFADCCNEFWWTSTYVAKGLWRQE 180

CHCC20489_0347 SREDGLTVILLDKDQDLPVIPPPTDREYWVQKPSPQCFADCCNEFWWTSTYVAKGLWRQE 180

CHCC14568_0477 SREDGLTVILLDKDQDLPVIPPPTDREYWVQKPSPQCFADCCNEFWWTSTYVAKGLWRQE 180

CHCC20339_0023 SREDGLTVILLDKDQDLPVIPPPTDREYWVQKPSPQCFADCCNEFWWTSTYVAKGLWRQE 180

CHCC15325_0415 SREDGLTVILLDKDQDLPVIPPPTDREYWVQKPSPQCFADCCNEFWWTSTYVAKGLWRQE 180

CHCC20345_4264 SREDGLTVILLDKDQDLPVIPPPTDREYWVQKPSPQCFADCCNEFWWTSTYVAKGLWRQE 180

CHCC14688_3358 SREDGLTVILLDKDQDLPVIPPPTDREYWVQKPSPQCFADCCNEFWWTSTYVAKGLWRQE 180

CHCC5025_0548 SREDGLTVILLDKDQDLPVIPPPTDREYWVQKPSPQCFADCCNEFWWTSTYVAKGLWRQE 180

CHCC14816_0249 SREDGLTVILLDKDQDLPVIPPPTDREYWVQKPSPQCFADCCNEFWWTSTYVAKGLWRQE 180

CHCC14815_0971 SREDGLTVILLDKDQDLPVIPPPTDREYWVQKPSPQCFADCCNEFWWTSTYVAKGLWRQE 180

CHCC14818_3872 SREDGLTVILLDKDQDLPVIPPPTDREYWVQKPSPQCFADCCNEFWWTSTYVAKGLWRQE 180

CHCC15292_0697 SREDGLTVILLDKDQDLPVIPPPTDREYWVQKPSPQCFADCCNEFWWTSTYVAKGLWRQE 180

CHCC14562_0762 SREDGLTVILLDKDQDLPVIPPPTDREYWVQKPSPQCFADCCNEFWWTSTYVAKGLWRQE 180

CHCC14561_2620 SREDGLTVILLDKDQDLPVIPPPTDREYWVQKPSPQCFADCCNEFWWTSTYVAKGLWRQE 180

DSM13_0192 SREDGLTVILLDKDQDLPVIPPPTDREYWVQKPSPQCFADCCNEFWWTSTYVAKGLWRQE 180

CHCC15075_1356 SREDGLTVILLDKDQDLPVIPPPTDREYWVQKPSPQCFADCCNEFWWTSTYVAKGLWRQE 180

CHCC20327_1735 SREDGLTVILLDKDQDLPVIPPPTDREYWVQKPSPQCFSDCCNEFWWTSTYVAKGLWRQE 173

CHCC14814_3092 CREDGLTVILLDKDQDLPAIPPPTDREYWVQKPSPQCFADCCNEFWWTSTYVAKGLWRQE 173

CHCC20375_3122 CREDSLTVILLDKDQDLPAIPPPTDREYWVQKPSTQCFADCCNEFWWTSTYVAKGLWRQE 173

.***.*************.***************.***:*********************

CHCC15381_2836 ILYALDHLNLVRAMLLKMLEWKVGFETGFSLSIGKNAKFLDRYVDRNTWERLLETYPPAG 233

CHCC12620_0739 ILYALDHLNLVRAMLLKMLEWKVGFETGFSLSIGKNAKFLDRYVDRNTWERLLETYPPAG 233

CHCC19467_4580 ILYALDHLNLVRAMLLKMLEWKVGFETGFSLSIGKNAKFLDRYVDRNTWERLLETYPPAG 233

CHCC19468_2703 ILYALDHLNLVRAMLLKMLEWKVGFETGFSLSIGKNAKFLDRYVDRNTWERLLETYPPAG 233

CHCC20497_4637 ILYALDHLNLVRAMLLKMLEWKVGFETGFSLSIGKNAKFLDRYVDRNTWERLLETYPPAG 233

CHCC20491_0559 ILYALDHLNLVRAMLLKMLEWKVGFETGFSLSIGKNAKFLDRYVDRNTWERLLETYPPAG 233

CHCC20492_2745 ILYALDHLNLVRAMLLKMLEWKVGFETGFSLSIGKNAKFLDRYVDRNTWERLLETYPPAG 233

CHCC14523_3914 ILYALDHLNLVRAMLLKMLEWKVGFETGFSLSIGKNAKFLDRYVDRNTWERLLETYPPAG 233

CHCC14527_2372 ILYALDHLNLVRAMLLKMLEWKVGFETGFSLSIGKNAKFLDRYVDRNTWERLLETYPPAG 233

ATCC9945A_0220 ILYALDHLNLVRAMLLKMLEWKVGFETGFSLSIGKNAKFLDRYVDRNTWERLLETYPPAG 233

CHCC15136_3271 ILYALDHLNLVRAMLLKMLEWKVGFETGFSLSIGKNAKFLDRYVDRNTWERLLETYPPAG 233

CHCC5019_4456 ILYALDHLNLVRAMLLKMLEWKVGFETGFSLSIGKNAKFLDRYVDRNTWERLLETYPPAG 233

CHCC15337_4610 ILYALDHLNLVRAMLLKMLEWKVGFETGFSLSIGKNAKFLDRYVDRNTWERLLETYPPAG 233

CHCC15332_3737 ILYALDHLNLVRAMLLKMLEWKVGFETGFSLSIGKNAKFLDRYVDRNTWERLLETYPPAG 233

CHCC5022_3897 ILYALDHLNLVRAMLLKMLEWKVGFETGFSLSIGKNAKFLDRYVDRNTWERLLETYPPAG 233

CHCC5023_1979 ILYALDHLNLVRAMLLKMLEWKVGFETGFSLSIGKNAKFLDRYVDRNTWERLLETYPPAG 233

CHCC5021_2115 ILYALDHLNLVRAMLLKMLEWKVGFETGFSLSIGKNAKFLDRYVDRNTWERLLETYPPAG 233

CHCC14817_1577 ILYALDHLNLVRAMLLKMLEWKVGFETGFSLSIGKNAKFLDRYVDRNTWERLLETYPPAG 233

CHCC4186_2037 ILYALDHLNLVRAMLLKMLEWKVGFETGFSLSIGKNAKFLDRYVDRNTWERLLETYPPAG 233

CHCC20488_0914 ILYALDHLNLVRAMLLKMLEWKVGFETGFSLSIGKNAKFLDRYVDRNTWERLLETYPPAG 233

BL09_0219 ILYALDHLNLVRAMLLKMLEWKVGFETGFSLSIGKNAKFLDRYVDRNTWERLLETYPPAG 233

CHCC20331_1437 ILYALDHLNLIRAMLLKMLEWKVGFETGFSLSIGKNAKFLDRYVDRNTWERLLETYPPAG 233

CHCC20347_3231 ILYALDHLNLIRAMLLKMLEWKVGFETGFSLSIGKNAKFLDRYVDRNTWERLLETYPPAD 233

CHCC20333_3258 ILYALDHLNLVRAMMLKMLEWKVGFETGFSLSIGKNAKFLDRYVDRNTWERLLETYPPAD 233

CHCC20490_1916 ILYALDHLNLVRAMLLKMLEWKVGFETGFSLSIGKNAKFLDRYVDRNTWERLLETYPPAD 233

CHCC20348_4391 ILYALDHLNLVRAMLLKMLEWKVGFETGFSLSIGKNAKFLDRYVDRNTWERLLETYPPAD 233

CHCC5027_3368 ILYALDHLNLVRAMLLKMLEWKVGFETGFSLSIGKNAKFLDRYVDRNTWERLLETYPPAD 233

CHCC14820_1469 ILYALDHLNLVRAMLLKMLEWKVGFETGFSLSIGKNAKFLDRYVDRNTWERLLETYPPAD 233

CHCC20372_2718 ILYALDHLNLVRAMLLKMLEWKVGFETGFSLSIGKNAKFLDRYVDRNTWERLLETYPPAD 233

CHCC20373_0250 ILYALDHLNLVRAMLLKMLEWKVGFETGFSLSIGKNAKFLDRYVDQNTWERLLETYPPAD 233

CHCC15289_3953 ILYALDHLNLVRAMLLKMLEWKVGFETGFSLSIGKNAKFLDRYVDQNTWERLLETYPPAD 233

CHCC14564_2767 ILYALDHLNLVRAMLLKMLEWKVGFETGFSLSIGKNAKFLDRYVDQNTWERLLETYPPAD 233

CHCC15290_2999 ILYALDHLNLVRAMLLKMLEWKVGFETGFSLSIGKNAKFLDRYVDQNTWERLLETYPPAD 233

CHCC20368_2019 ILYALDHLNLVRAMLLKMLEWKVGFETGFSLSIGKNAKFLDRYVDQNTWERLLETYPPAD 233

CHCC15291_0394 ILYALDHLNLVRAMLLKMLEWKVGFETGFSLSIGKNAKFLDRYVDQNTWERLLETYPPAD 233

CHCC20369_2215 ILYALDHLNLVRAMLLKMLEWKVGFETGFSLSIGKNAKFLDRYVDQNTWERLLETYPPAD 233

CHCC14598_1058 ILYALDHLNLVRAMLLKMLEWKVGFETGFSLSIGKNAKFLDRYVDQNTWERLLETYPPAD 233

CHCC14596_0534 ILYALDHLNLVRAMLLKMLEWKVGFETGFSLSIGKNAKFLDRYVDQNTWERLLETYPPAD 233

CHCC14810_4312 ILYALDHLNLVRAMLLKMLEWKVGFETGFSLSIGKNAKFLDRYVDQNTWERLLETYPPAD 233

CHCC14819_0399 ILYALDHLNLVRAMLLKMLEWKVGFETGFSLSIGKNAKFLDRYVDQNTWERLLETYPPAD 233

CHCC14813_2856 ILYALDHLNLVRAMLLKMLEWKVGFETGFSLSIGKNAKFLDRYVDQNTWERLLETYPPAD 233

CHCC20441_1080 ILYALDHLNLVRAMLLKMLEWKVGFETGFSLSIGKNAKFLDRYVDQNTWERLLETYPPAD 233

CHCC20440_1830 ILYALDHLNLVRAMLLKMLEWKVGFETGFSLSIGKNAKFLDRYVDQNTWERLLETYPPAD 233

CHCC20442_1308 ILYALDHLNLVRAMLLKMLEWKVGFETGFSLSIGKNAKFLDRYVDQNTWERLLETYPPAD 233

CHCC5024_4269 ILYALDHLNLVRAMLLKMLEWKVGFETGFSLSIGKNAKFLDRYVDQNTWERLLETYPPAD 233

CHCC5026_1706 ILYALDHLNLVRAMLLKMLEWKVGFETGFSLSIGKNAKFLDRYVDQNTWERLLETYPPAD 233

CHCC5020_0926 ILYALDHLNLVRAMLLKMLEWKVGFETGFSLSIGKNAKFLDRYVDQNTWERLLETYPPAD 233

CHCC14600_3227 ILYALDHLNLVRAMLLKMLEWKVGFETGFSLSIGKNAKFLDRYVDQNTWERLLETYPPAD 233

CHCC14808_3671 ILYALDHLNLVRAMLLKMLEWKVGFETGFSLSIGKNAKFLDRYVDQNTWERLLETYPPAD 233

CHCC14809_3060 ILYALDHLNLVRAMLLKMLEWKVGFETGFSLSIGKNAKFLDRYVDQNTWERLLETYPPAD 233

CHCC15335_1764 ILYALDHLNLVRAMLLKMLEWKVGFETGFSLSIGKNAKFLDRYVDQNTWERLLETYPPAD 233

CHCC20342_1142 ILYALDHLNLVRAMLLKMLEWKVGFETGFSLSIGKNAKFLDRYVDQNTWERLLETYPPAD 233

CHCC20343_0033 ILYALDHLNLVRAMLLKMLEWKVGFETGFSLSIGKNAKFLDRYVDQNTWERLLETYPPAD 233

CHCC20341_2338 ILYALDHLNLVRAMLLKMLEWKVGFETGFSLSIGKNAKFLDRYVDQNTWERLLETYPPAD 233

CHCC20344_4475 ILYALDHLNLVRAMLLKMLEWKVGFETGFSLSIGKNAKFLDRYVDQNTWERLLETYPPAD 233

CHCC15139_1359 ILYALDHLNLVRAMLLKMLEWKVGFETGFSLSIGKNAKFLDRYVDQNTWERLLETYPPAD 233

CHCC15320_2309 ILYALDHLNLVRAMLLKMLEWKVGFETGFSLSIGKNAKFLDRYVDQNTWERLLETYPPAD 233

CHCC15322_3643 ILYALDHLNLVRAMLLKMLEWKVGFETGFSLSIGKNAKFLDRYVDQNTWERLLETYPPAD 233

CHCC14525_3596 ILYALDHLNLVRAMLLKMLEWKVGFETGFSLSIGKNAKFLDRYVDQNTWERLLETYPPAD 233

CHCC14441_1578 ILYALDHLNLVRAMLLKMLEWKVGFETGFSLSIGKNAKFLDRYVDQNTWERLLETYPPAD 233

CHCC15311_0626 ILYALDHLNLVRAMLLKMLEWKVGFETGFSLSIGKNAKFLDRYVDQNTWERLLETYPPAD 233

CHCC15315_4193 ILYALDHLNLVRAMLLKMLEWKVGFETGFSLSIGKNAKFLDRYVDQNTWERLLETYPPAD 233

CHCC20487_3824 ILYALDHLNLVRAMLLKMLEWKVGFETGFSLSIGKNAKFLDRYVDQNTWERLLETYPPAD 233

CHCC20486_2040 ILYALDHLNLVRAMLLKMLEWKVGFETGFSLSIGKNAKFLDRYVDQNTWERLLETYPPAD 233

CHCC15318_3693 ILYALDHLNLVRAMLLKMLEWKVGFETGFSLSIGKNAKFLDRYVDQNTWERLLETYPPAD 233

CHCC20323_0096 ILYALDHLNLVRAMLLKMLEWKVGFETGFSLSIGKNAKFLDRYVDQNTWERLLETYPPAD 233

CHCC20325_3729 ILYALDHLNLVRAMLLKMLEWKVGFETGFSLSIGKNAKFLDRYVDQNTWERLLETYPPAD 233

CHCC15087_0205 ILYALDHLNLVRAMLLKMLEWKVGFETGFSLSIGKNAKFLDRYVDQNTWERLLETYPPAD 233

CHCC14437_0388 ILYALDHLNLVRAMLLKMLEWKVGFETGFSLSIGKNAKFLDRYVDQNTWERLLETYPPAD 233

CHCC14431_3647 ILYALDHLNLVRAMLLKMLEWKVGFETGFSLSIGKNAKFLDRYVDQNTWERLLETYPPAD 233

CHCC14566_2259 ILYALDHLNLVRAMLLKMLEWKVGFETGFSLSIGKNAKFLDRYVDQNTWERLLETYPPAD 233

CHCC20493_0357 ILYALDHLNLVRAMLLKMLEWKVGFETGFSLSIGKNAKFLDRYVDQNTWERLLETYPPAD 233

CHCC20494_1491 ILYALDHLNLVRAMLLKMLEWKVGFETGFSLSIGKNAKFLDRYVDQNTWERLLETYPPAD 233

CHCC20495_0876 ILYALDHLNLVRAMLLKMLEWKVGFETGFSLSIGKNAKFLDRYVDQNTWERLLETYPPAD 233

CHCC16736_1642 ILYALDHLNLVRAMLLKMLEWKVGFETGFSLSIGKNAKFLDRYVDQNTWERLLETYPPAD 233

CHCC19466_3945 ILYALDHLNLVRAMLLKMLEWKVGFETGFSLSIGKNAKFLDRYVDQNTWERLLETYPPAD 233

CHCC15543_3600 ILYALDHLNLVRAMLLKMLEWKVGFETGFSLSIGKNAKFLDRYVDQNTWERLLETYPPAD 233

CHCC15546_0033 ILYALDHLNLVRAMLLKMLEWKVGFETGFSLSIGKNAKFLDRYVDQNTWERLLETYPPAD 233

CHCC10893_2818 ILYALDHLNLVRAMLLKMLEWKVGFETGFSLSIGKNAKFLDRYVDQNTWERLLETYPPAD 233

CHCC16874_4694 ILYALDHLNLVRAMLLKMLEWKVGFETGFSLSIGKNAKFLDRYVDQNTWERLLETYPPAD 240

CHCC15091_2903 ILYALDHLNLVRAMLLKMLEWKVGFETGFSLSIGKNAKFLDRYVDQNTWERLLETYPPAD 240

CHCC14429_3013 ILYALDHLNLVRAMLLKMLEWKVGFETGFSLSIGKNAKFLDRYVDQNTWERLLETYPPAD 240

CHCC20496_3908 ILYALDHLNLVRAMLLKMLEWKVGFETGFSLSIGKNAKFLDRYVDQNTWERLLETYPPAD 240

CHCC14559_1463 ILYALDHLNLVRAMLLKMLEWKVGFETGFSLSIGKNAKFLDRYVDQNTWERLLETYPPAD 240

CHCC14557_3890 ILYALDHLNLVRAMLLKMLEWKVGFETGFSLSIGKNAKFLDRYVDQNTWERLLETYPPAD 240

CHCC14435_2912 ILYALDHLNLVRAMLLKMLEWKVGFETGFSLSIGKNAKFLDRYVDQNTWERLLETYPPAD 240

CHCC20489_0347 ILYALDHLNLVRAMLLKMLEWKVGFETGFSLSIGKNAKFLDRYVDQNTWERLLETYPPAD 240

CHCC14568_0477 ILYALDHLNLVRAMLLKMLEWKVGFETGFSLSIGKNAKFLDRYVDQNTWERLLETYPPAD 240

CHCC20339_0023 ILYALDHLNLVRAMLLKMLEWKVGFETGFSLSIGKNAKFLDRYVDQNTWERLLETYPPAD 240

CHCC15325_0415 ILYALDHLNLVRAMLLKMLEWKVGFETGFSLSIGKNAKFLDRYVDQNTWERLLETYPPAD 240

CHCC20345_4264 ILYALDHLNLVRAMLLKMLEWKVGFETGFSLSIGKNAKFLDRYVDQNTWERLLETYPPAD 240

CHCC14688_3358 ILYALDHLNLVRAMLLKMLEWKVGFETGFSLSIGKNAKFLDRYVDQNTWERLLETYPPAD 240

CHCC5025_0548 ILYALDHLNLVRAMLLKMLEWKVGFETGFSLSIGKNAKFLDRYVDQNTWERLLETYPPAD 240

CHCC14816_0249 ILYALDHLNLVRAMLLKMLEWKVGFETGFSLSIGKNAKFLDRYVDQNTWERLLETYPPAD 240

CHCC14815_0971 ILYALDHLNLVRAMLLKMLEWKVGFETGFSLSIGKNAKFLDRYVDQNTWERLLETYPPAD 240

CHCC14818_3872 ILYALDHLNLVRAMLLKMLEWKVGFETGFSLSIGKNAKFLDRYVDQNTWERLLETYPPAD 240

CHCC15292_0697 ILYALDHLNLVRAMLLKMLEWKVGFETGFSLSIGKNAKFLDRYVDQNTWERLLETYPPAD 240

CHCC14562_0762 ILYALDHLNLVRAMLLKMLEWKVGFETGFSLSIGKNAKFLDRYVDQNTWERLLETYPPAD 240

CHCC14561_2620 ILYALDHLNLVRAMLLKMLEWKVGFETGFSLSIGKNAKFLDRYVDQNTWERLLETYPPAD 240

DSM13_0192 ILYALDHLNLVRAMLLKMLEWKVGFETGFSLSIGKNAKFLDRYVDQNTWERLLETYPPAD 240

CHCC15075_1356 ILYALDHLNLVRAMLLKMLEWKVGFETGFSLSIGKNAKFLDRYVDQNTWERLLETYPPAD 240

CHCC20327_1735 ILYALDHLNLVRAMLLKMLEWKVGFETGFSLSIGKNAKFLDRYVDQNTWERLLETYPPAD 233

CHCC14814_3092 ILYALDHLNLVRAMLLKMLEWKVGFETGFSLSIGKNAKFLDRYVDRNTWERLLETYPPAD 233

CHCC20375_3122 ILYALDHLNLVRAMLLKMLEWKVGFETGFSLSIGKNAKFLDRYVDRNTWERLLETYPPAD 233

**********:***:******************************:*************.

CHCC15381_2836 YERVWNSLFKMWDLFEETAAAVAEHLAEQYLFAEAEKVKQYVRRVQQLKPDAKVID 289

CHCC12620_0739 YERVWNSLFKMWDLFEETAAAVAEHLAEQYLFAEAEKVKQYVRRVQQLKPDAKVID 289

CHCC19467_4580 YERVWNSLFKMWDLFEETAAAVAEHLAEQYLFAEAEKVKQYVRRVQQLKPDAKVID 289

CHCC19468_2703 YERVWNSLFKMWDLFEETAAAVAEHLAEQYLFAEAEKVKQYVRRVQQLKPDAKVID 289

CHCC20497_4637 YERVWNSLFKMWDLFEETAAAVAEHLAEQYLFAEAEKVKQYVRRVQQLKPDAKVID 289

CHCC20491_0559 YERVWNSLFKMWDLFEETAAAVAEHLAEQYLFAEAEKVKQYVRRVQQLKPDAKVID 289

CHCC20492_2745 YERVWNSLFKMWDLFEETAAAVAEHLAEQYLFAEAEKVKQYVRRVQQLKPDAKVID 289

CHCC14523_3914 YERVWNSLFKMWDLFEETAAAVAEHLAEQYLFAEAEKVKQYVRRVQQLKPDAKVID 289

CHCC14527_2372 YERVWNSLFKMWDLFEETAAAVAEHLAEQYLFAEAEKVKQYVRRVQQLKPDAKVID 289

ATCC9945A_0220 YERVWNSLFKMWDLFEETAAAVAEHLAEQYLFAEAEKVKQYVRRVQQLKPDAKVID 289

CHCC15136_3271 YERVWNSLFKMWDLFEETAAAVAEHLAEQYLFAEAEKVKQYVRRVQQLKPDAKVID 289

CHCC5019_4456 YERVWNSLFKMWDLFEETAAAVAEHLAEQYLFAEAEKVKQYVRRVQQLKPDAKVID 289

CHCC15337_4610 YERVWNSLFKMWDLFEETAAAVAEHLAEQYLFAEAEKVKQYVRRVQQLKPDAKVID 289

CHCC15332_3737 YERVWNSLFKMWDLFEETAAAVAEHLAEQYLFAEAEKVKQYVRRVQQLKPDAKVID 289

CHCC5022_3897 YERVWNSLFKMWDLFEETAAAVAEHLAEQYLFAEAEKVKQYVRRVQQLKPDAKVID 289

CHCC5023_1979 YERVWNSLFKMWDLFEETAAAVAEHLAEQYLFAEAEKVKQYVRRVQQLKPDAKVID 289

CHCC5021_2115 YERVWNSLFKMWDLFEETAAAVAEHLAEQYLFAEAEKVKQYVRRVQQLKPDAKVID 289

CHCC14817_1577 YERVWNSLFKMWDLFEETAAAVAEHLAEQYLFAEAEKVKQYVRRVQQLKPDAKVID 289

CHCC4186_2037 YERVWNSLFKMWDLFEETAAAVAEHLAEQYLFAEAEKVKQYVRRVQQLKPDAKVID 289

CHCC20488_0914 YERVWNSLFKMWDLFEETAAAVAEHLAEQYLFAEAEKVKQYVRRVQQLKPDAKVID 289

BL09_0219 YERVWNSLFKMWDLFEETAAAVAEHLAEQYLFAEAEKVKQYVRRVQQLKPDAKVID 289

CHCC20331_1437 YERVWNSLFKMWDLFEETAAAVAEHLAEQYLFAEAEKVKQYVRRVQQLKPDAKVID 289

CHCC20347_3231 YERVWNSLFKMWDLFEETAAAVAEHLAEQYLFAEAEKVKQYVRRVQQLKPDAKVID 289

CHCC20333_3258 YERVWNSLFKMWDLFEETAAAVAEHLAEQYLFAEAEKVKQYVRRVQQLKPDAKVID 289

CHCC20490_1916 YERVWNSLFKMWDLFEETAAAVAEHLAEQYLFAEAEKVKQYVRRVQQLKPDAKVID 289

CHCC20348_4391 YERVWNSLFKMWDLFEETAAAVAEHLAEQYLFAEAEKVKQYVRRVQQLKPDAKVID 289

CHCC5027_3368 YERVWNSLFKMWDLFEETAAAVAEHLAEQYLFAEAEKVKQYVRRVQQLKPDAKVID 289

CHCC14820_1469 YERVWNSLFKMWDLFEETAAAVAEHLAEQYLFAEAEKVKQYVRRVQQLKPDAKVID 289

CHCC20372_2718 YERVWNSLFKMWDLFEETAAAVAEHLAEQYLFAEAEKVKQYVRRVQQLKPDAKVID 289

CHCC20373_0250 YKRVWNSLFKMWELFEETAAAVAEHLNEQYLFAEAEKVRQYLRRVQQLKPDAKEID 289

CHCC15289_3953 YKRVWNSLFKMWELFEETAAAVAEHLNEQYLFAEAEKVRQYLRRVQQLKPDAKEID 289

CHCC14564_2767 YKRVWNSLFKMWELFEETAAAVAEHLNEQYLFAEAEKVRQYLRRVQQLKPDAKEID 289

CHCC15290_2999 YKRVWNSLFKMWELFEETAAAVAEHLNEQYLFAEAEKVRQYLRRVQQLKPDAKEID 289

CHCC20368_2019 YKRVWNSLFKMWELFEETAAAVAEHLNEQYLFAEAEKVRQYLRRVQQLKPDAKEID 289

CHCC15291_0394 YKRVWNSLFKMWELFEETAAAVAEHLNEQYLFAEAEKVRQYLRRVQQLKPDAKEID 289

CHCC20369_2215 YKRVWNSLFKMWELFEETAAAVAEHLNEQYLFAEAEKVRQYLRRVQQLKPDAKEID 289

CHCC14598_1058 YKRVWNSLFKMWELFEETAAAVAEHLNEQYLFAEAEKVRQYLRRVQQLKPDAKEID 289

CHCC14596_0534 YKRVWNSLFKMWELFEETAAAVAEHLNEQYLFAEAEKVRQYLRRVQQLKPDAKEID 289

CHCC14810_4312 YKRVWNSLFKMWELFEETAAAVAEHLNEQYLFAEAEKVRQYLRRVQQLKPDAKEID 289

CHCC14819_0399 YKRVWNSLFKMWELFEETAAAVAEHLNEQYLFAEAEKVRQYLRRVQQLKPDAKEID 289

CHCC14813_2856 YKRVWNSLFKMWELFEETAAAVAEHLNEQYLFAEAEKVRQYLRRVQQLKPDAKEID 289

CHCC20441_1080 YKRVWNSLFKMWELFEETAAAVAEHLNEQYLFAEAEKVRQYLRRVQQLKPDAKEID 289

CHCC20440_1830 YKRVWNSLFKMWELFEETAAAVAEHLNEQYLFAEAEKVRQYLRRVQQLKPDAKEID 289

CHCC20442_1308 YKRVWNSLFKMWELFEETAAAVAEHLNEQYLFAEAEKVRQYLRRVQQLKPDAKEID 289

CHCC5024_4269 YKRVWNSLFKMWELFEETAAAVAEHLNEQYLFAEAEKVRQYLRRVQQLKPDAKEID 289

CHCC5026_1706 YKRVWNSLFKMWELFEETAAAVAEHLNEQYLFAEAEKVRQYLRRVQQLKPDAKEID 289

CHCC5020_0926 YKRVWNSLFKMWELFEETAAAVAEHLNEQYLFAEAEKVRQYLRRVQQLKPDAKEID 289

CHCC14600_3227 YKRVWNSLFKMWELFEETAAAVAEHLNEQYLFAEAEKVRQYLRRVQQLKPDAKEID 289

CHCC14808_3671 YKRVWNSLFKMWELFEETAAAVAEHLNEQYLFAEAEKVRQYLRRVQQLKPDAKEID 289

CHCC14809_3060 YKRVWNSLFKMWELFEETAAAVAEHLNEQYLFAEAEKVRQYLRRVQQLKPDAKEID 289

CHCC15335_1764 YKRVWNSLFKMWELFEETAAAVAEHLNEQYLFAEAEKVRQYLRRVQQLKPDAKEID 289

CHCC20342_1142 YKRVWNSLFKMWELFEETAAAVAEHLNEQYLFAEAEKVRQYLRRVQQLKPDAKEID 289

CHCC20343_0033 YKRVWNSLFKMWELFEETAAAVAEHLNEQYLFAEAEKVRQYLRRVQQLKPDAKEID 289

CHCC20341_2338 YKRVWNSLFKMWELFEETAAAVAEHLNEQYLFAEAEKVRQYLRRVQQLKPDAKEID 289

CHCC20344_4475 YKRVWNSLFKMWELFEETAAAVAEHLNEQYLFAEAEKVRQYLRRVQQLKPDAKEID 289

CHCC15139_1359 YKRVWNSLFKMWELFEETAAAVAEHLNEQYLFAEAEKVRQYLRRVQQLKPDAKEID 289

CHCC15320_2309 YKRVWNSLFKMWELFEETAAAVAEHLNEQYLFAEAEKVRQYLRRVQQLKPDAKEID 289

CHCC15322_3643 YKRVWNSLFKMWELFEETAAAVAEHLNEQYLFAEAEKVRQYLRRVQQLKPDAKEID 289

CHCC14525_3596 YKRVWNSLFKMWELFEETAAAVAEHLNEQYLFAEAEKVRQYLRRVQQLKPDAKEID 289

CHCC14441_1578 YKRVWNSLFKMWELFEETAAAVAEHLNEQYLFAEAEKVRQYLRRVQQLKPDAKEID 289

CHCC15311_0626 YKRVWNSLFKMWELFEETAAAVAEHLNEQYLFAEAEKVRQYLRRVQQLKPDAKEID 289

CHCC15315_4193 YKRVWNSLFKMWELFEETAAAVAEHLNEQYLFAEAEKVRQYLRRVQQLKPDAKEID 289

CHCC20487_3824 YKRVWNSLFKMWELFEETAAAVAEHLNEQYLFAEAEKVRQYLRRVQQLKPDAKEID 289

CHCC20486_2040 YKRVWNSLFKMWELFEETAAAVAEHLNEQYLFAEAEKVRQYLRRVQQLKPDAKEID 289

CHCC15318_3693 YKRVWNSLFKMWELFEETAAAVAEHLNEQYLFAEAEKVRQYLRRVQQLKPDAKEID 289

CHCC20323_0096 YKRVWNSLFKMWELFEETAAAVAEHLNEQYLFAEAEKVRQYLRRVQQLKPDAKEID 289

CHCC20325_3729 YKRVWNSLFKMWELFEETAAAVAEHLNEQYLFAEAEKVRQYLRRVQQLKPDAKEID 289

CHCC15087_0205 YKRVWNSLFKMWELFEETAAAVAEHLNEQYLFAEAEKVRQYLRRVQQLKPDAKEID 289

CHCC14437_0388 YKRVWNSLFKMWELFEETAAAVAEHLNEQYLFAEAEKVRQYLRRVQQLKPDAKEID 289

CHCC14431_3647 YKRVWNSLFKMWELFEETAAAVAEHLNEQYLFAEAEKVRQYLRRVQQLKPDAKEID 289

CHCC14566_2259 YKRVWNSLFKMWELFEETAAAVAEHLNEQYLFAEAEKVRQYLRRVQQLKPDAKEID 289

CHCC20493_0357 YKRVWNSLFKMWELFEETAAAVAEHLNEQYLFAEAEKVRQYLRRVQQLKPDAKEID 289

CHCC20494_1491 YKRVWNSLFKMWELFEETAAAVAEHLNEQYLFAEAEKVRQYLRRVQQLKPDAKEID 289

CHCC20495_0876 YKRVWNSLFKMWELFEETAAAVAEHLNEQYLFAEAEKVRQYLRRVQQLKPDAKEID 289

CHCC16736_1642 YKRVWNSLFKMWELFEETAAAVAEHLNEQYLFAEAEKVRQYLRRVQQLKPDAKEID 289

CHCC19466_3945 YKRVWNSLFKMWELFEETAAAVAEHLNEQYLFAEAEKVRQYLRRVQQLKPDAKEID 289

CHCC15543_3600 YKRVWNSLFKMWELFEETAAAVAEHLNEQYLFAEAEKVRQYLRRVQQLKPDAKEID 289

CHCC15546_0033 YKRVWNSLFKMWELFEETAAAVAEHLNEQYLFAEAEKVRQYLRRVQQLKPDAKEID 289

CHCC10893_2818 YKRVWNSLFKMWELFEETAAAVAEHLNEQYLFAEAEKVRQYLRRVQQLKPDAKEID 289

CHCC16874_4694 YKRVWNSLFKMWELFEETAAAVAEHLNEQYLFAEAEKVRQYLRRVQQLKPDAKEID 296

CHCC15091_2903 YKRVWNSLFKMWELFEET-AAVAEHLNEQYLFAEAEKVRQYLRRVQQLKPDAKEID 295

CHCC14429_3013 YKRVWNSLFKMWELFEETAAAVAEHLNEQYLFAEAEKVRQYLRRVQQLKPDAKEID 296

CHCC20496_3908 YKRVWNSLFKMWELFEETAAAVAEHLNEQYLFAEAEKVRQYLRRVQQLKPDAKEID 296

CHCC14559_1463 YKRVWNSLFKMWELFEETAAAVAEHLNEQYLFAEAEKVRQYLRRVQQLKPDAKEID 296

CHCC14557_3890 YKRVWNSLFKMWELFEETAAAVAEHLNEQYLFAEAEKVRQYLRRVQQLKPDAKEID 296

CHCC14435_2912 YKRVWNSLFKMWELFEETAAAVAEHLNEQYLFAEAEKVRQYLRRVQQLKPDAKEID 296

CHCC20489_0347 YKRVWNSLFKMWELFEETAAAVAEHLNEQYLFAEAEKVRQYLRRVQQLKPDAKEID 296

CHCC14568_0477 YKRVWNSLFKMWELFEETAAAVAEHLNEQYLFAEAEKVRQYLRRVQQLKPDAKEID 296

CHCC20339_0023 YKRVWNSLFKMWELFEETAAAVAEHLNEQYLFAEAEKVRQYLRRVQQLKPDAKEID 296

CHCC15325_0415 YKRVWNSLFKMWELFEETAAAVAEHLNEQYLFAEAEKVRQYLRRVQQLKPDAKEID 296

CHCC20345_4264 YKRVWNSLFKMWELFEETAAAVAEHLNEQYLFAEAEKVRQYLRRVQQLKPDAKEID 296

CHCC14688_3358 YKRVWNSLFKMWELFEETAAAVAEHLNEQYLFAEAEKVRQYLRRVQQLKPDAKEID 296

CHCC5025_0548 YKRVWNSLFKMWELFEETAAAVAEHLNEQYLFAEAEKVRQYLRRVQQLKPDAKEID 296

CHCC14816_0249 YKRVWNSLFKMWELFEETAAAVAEHLNEQYLFAEAEKVRQYLRRVQQLKPDAKEID 296

CHCC14815_0971 YKRVWNSLFKMWELFEETAAAVAEHLNEQYLFAEAEKVRQYLRRVQQLKPDAKEID 296

CHCC14818_3872 YKRVWNSLFKMWELFEETAAAVAEHLNEQYLFAEAEKVRQYLRRVQQLKPDAKEID 296

CHCC15292_0697 YKRVWNSLFKMWELFEETAAAVAEHLNEQYLFAEAEKVRQYLRRVQQLKPDAKEID 296

CHCC14562_0762 YKRVWNSLFKMWELFEETAAAVAEHLNEQYLFAEAEKVRQYLRRVQQLKPDAKEID 296

CHCC14561_2620 YKRVWNSLFKMWELFEETAAAVAEHLNEQYLFAEAEKVRQYLRRVQQLKPDAKEID 296

DSM13_0192 YKRVWNSLFKMWELFEETAAAVAEHLNEQYLFAEAEKVRQYLRRVQQLKPDAKEID 296

CHCC15075_1356 YKRVWNSLFKMWELFEETAAAVAEHLNEQYLFAEAEKVRQYLRRVQQLKPDAKEID 296

CHCC20327_1735 YKRVWNSLFKMWELFEET-AAVAEHLNEQYLFAEAEKVRQYLRRVQQLKPDAKEID 288

CHCC14814_3092 HERVWHSLFKMGDLFEETAAAVAEHLDEQYLFREAEKVKQYVRRVQQLKPAAKEID 289

CHCC20375_3122 HERVWNSLFKMGDLFEETVAAVAEHLNEQYLFAEAEKVKQYLRRVQQLKPAAKEID 289

:***:***** :***** ******* ***** *****:**:******** ** **
