# Supplementary material for: Putative antibiotic resistance genes present in extant Bacillus licheniformis and Bacillus paralicheniformis strains are probably intrinsic and part of the ancient resistome
Source: PLoS One. 2019 Jan 15;14(1):e0210363. doi: 10.1371/journal.pone.0210363 (PMC6333372; doi:10.1371/journal.pone.0210363)
Supplement: S7 Fig — Sequence alignments were made with Clustal Omega using default settings. The left column indicates the locus tag of each aph gene per strain. Identical amino acids are indicated by an asterisk below each column. Residue numbers are indicated at the end of each row. (DOCX) [file pone.0210363.s007.docx]

Figure S7: Multiple sequence alignment of APH proteins

CLUSTAL 2.1 multiple sequence alignment

CHCC10893_2819 MFSIKKMLQNHYGIDSVNISPEQGGWTALAYQVTDGEAAFFLKVYDKNRASTAKWAALID 60

CHCC19466_3946 MFSIKKMLQNHYGIDSVNISPEQGGWTALAYQVTDGEAAFFLKVYDKNRASTAKWAALID 60

CHCC20494_1490 MFSIKKMLQNHYGIDSVNISPEQGGWTALAYQVTDGEAAFFLKVYDKNRASTAKWAALID 60

CHCC20493_0356 MFSIKKMLQNHYGIDSVNISPEQGGWTALAYQVTDGEAAFFLKVYDKNRASTAKWAALID 60

CHCC14437_0389 MFSIKKMLQNHYGIDSVNISPEQGGWTALAYQVTDGEAAFFLKVYDKNRASTAKWAALID 60

CHCC20323_0097 MFSIKKMLQNHYGIDSVNISPEQGGWTALAYQVTDGEAAFFLKVYDKNRASTAKWAALID 60

CHCC15318_3692 MFSIKKMLQNHYGIDSVNISPEQGGWTALAYQVTDGEAAFFLKVYDKNRASTAKWAALID 60

CHCC20486_2039 MFSIKKMLQNHYGIDSVNISPEQGGWTALAYQVTDGEAAFFLKVYDKNRASTAKWAALID 60

CHCC14441_1577 MFSIKKMLQNHYGIDSVNISPEQGGWTALAYQVTDGEAAFFLKVYDKNRASTAKWAALID 60

CHCC14525_3597 MFSIKKMLQNHYGIDSVNISPEQGGWTALAYQVTDGEAAFFLKVYDKNRASTAKWAALID 60

CHCC15322_3644 MFSIKKMLQNHYGIDSVNISPEQGGWTALAYQVTDGEAAFFLKVYDKNRASTAKWAALID 60

CHCC15139_1360 MFSIKKMLQNHYGIDSVNISPEQGGWTALAYQVTDGEAAFFLKVYDKNRASTAKWAALID 60

CHCC20344_4474 MFSIKKMLQNHYGIDSVNISPEQGGWTALAYQVTDGEAAFFLKVYDKNRASTAKWAALID 60

CHCC20341_2337 MFSIKKMLQNHYGIDSVNISPEQGGWTALAYQVTDGEAAFFLKVYDKNRASTAKWAALID 60

CHCC20343_0034 MFSIKKMLQNHYGIDSVNISPEQGGWTALAYQVTDGEAAFFLKVYDKNRASTAKWAALID 60

CHCC20342_1143 MFSIKKMLQNHYGIDSVNISPEQGGWTALAYQVTDGEAAFFLKVYDKNRASTAKWAALID 60

CHCC14600_3226 MFSIKKMLQNHYGIDSVNISPEQGGWTALAYQVTDGEAAFFLKVYDKNRASTAKWAALID 60

CHCC5020_0927 MFSIKKMLQNHYGIDSVNISPEQGGWTALAYQVTDGEAAFFLKVYDKNRASTAKWAALID 60

CHCC5026_1707 MFSIKKMLQNHYGIDSVNISPEQGGWTALAYQVTDGEAAFFLKVYDKNRASTAKWAALID 60

CHCC14813_2855 MFSIKKMLQNHYGIDSVNISPEQGGWTALAYQVTDGEAAFFLKVYDKNRASTAKWAALID 60

CHCC14810_4313 MFSIKKMLQNHYGIDSVNISPEQGGWTALAYQVTDGEAAFFLKVYDKNRASTAKWAALID 60

CHCC14598_1059 MFSIKKMLQNHYGIDSVNISPEQGGWTALAYQVTDGEAAFFLKVYDKNRASTAKWAALID 60

CHCC15291_0393 MFSIKKMLQNHYGIDSVNISPEQGGWTALAYQVTDGEAAFFLKVYDKNRASTAKWAALID 60

CHCC15290_3000 MFSIKKMLQNHYGIDSVNISPEQGGWTALAYQVTDGEAAFFLKVYDKNRASTAKWAALID 60

CHCC14564_2766 MFSIKKMLQNHYGIDSVNISPEQGGWTALAYQVTDGEAAFFLKVYDKNRASTAKWAALID 60

CHCC20373_0249 MFSIKKMLQNHYGIDSVNISPEQGGWTALAYQVTDGEAAFFLKVYDKNRASTAKWAALID 60

CHCC15289_3954 MFSIKKMLQNHYGIDSVNISPEQGGWTALAYQVTDGEAAFFLKVYDKNRASTAKWAALID 60

CHCC20325_3728 MFSIKKMLQNHYGIDSVNISPEQGGWTALAYQVTDGEAAFFLKVYDKNRASTAKWAALID 60

CHCC20440_1829 MFSIKKMLQNHYGIDSVNISPEQGGWTALAYQVTDGEAAFFLKVYDKNRASTAKWAALID 60

CHCC20441_1081 MFSIKKMLQNHYGIDSVNISPEQGGWTALAYQVTDGEAAFFLKVYDKNRASTAKWAALID 60

CHCC15546_0034 MFSIKKMLQNHYGIDSVNISPEQGGWTALAYQVTDGEAAFFLKVYDKNRASTAKWAALID 60

CHCC15543_3601 MFSIKKMLQNHYGIDSVNISPEQGGWTALAYQVTDGEAAFFLKVYDKNRASTAKWAALID 60

CHCC16874_4693 MFSIKKMLQNHYGIDSVNISPEQGGWTALAYQVTDGEAAFFLKVYDKNRASTAKWAALID 60

CHCC16736_1643 MFSIKKMLQNHYGIDSVNISPEQGGWTALAYQVTDGEAAFFLKVYDKNRASTAKWAALID 60

CHCC20495_0877 MFSIKKMLQNHYGIDSVNISPEQGGWTALAYQVTDGEAAFFLKVYDKNRASTAKWAALID 60

CHCC14557_3891 MFSIKKMLQNHYGIDSVNISPEQGGWTALAYQVTDGEAAFFLKVYDKNRASTAKWAALID 60

CHCC15315_4194 MFSIKKMLQNHYGIDSVNISPEQGGWTALAYQVTDGEAAFFLKVYDKNRASTAKWAALID 60

CHCC15311_0625 MFSIKKMLQNHYGIDSVNISPEQGGWTALAYQVTDGEAAFFLKVYDKNRASTAKWAALID 60

CHCC20345_4265 MFSIKKMLQNHYGIDSVNISPEQGGWTALAYQVTDGEAAFFLKVYDKNRASTAKWAALID 60

CHCC15335_1763 MFSIKKMLQNHYGIDSVNISPEQGGWTALAYQVTDGEAAFFLKVYDKNRASTAKWAALID 60

CHCC14808_3670 MFSIKKMLQNHYGIDSVNISPEQGGWTALAYQVTDGEAAFFLKVYDKNRASTAKWAALID 60

CHCC5024_4270 MFSIKKMLQNHYGIDSVNISPEQGGWTALAYQVTDGEAAFFLKVYDKNRASTAKWAALID 60

CHCC14819_0398 MFSIKKMLQNHYGIDSVNISPEQGGWTALAYQVTDGEAAFFLKVYDKNRASTAKWAALID 60

CHCC14429_3014 MFSIKKMLQNHYGIDSVNISPEQGGWTALAYQVTDGEAAFFLKVYDKNRASTAKWAALID 60

CHCC15091_2902 MFSIKKMLQNHYGIDSVNISPEQGGWTALAYQVTDGEAAFFLKVYDKNRASTAKWAALID 60

CHCC20496_3909 MFSIKKMLQNHYGIDSVNISPEQGGWTALAYQVTDGEAAFFLKVYDKNRASTAKWAALID 60

CHCC14559_1464 MFSIKKMLQNHYGIDSVNISPEQGGWTALAYQVTDGEAAFFLKVYDKNRASTAKWAALID 60

CHCC14566_2258 MFSIKKMLQNHYGIDSVNISPEQGGWTALAYQVTDGEAAFFLKVYDKNRASTAKWAALID 60

CHCC14435_2913 MFSIKKMLQNHYGIDSVNISPEQGGWTALAYQVTDGEAAFFLKVYDKNRASTAKWAALID 60

CHCC20489_0346 MFSIKKMLQNHYGIDSVNISPEQGGWTALAYQVTDGEAAFFLKVYDKNRASTAKWAALID 60

CHCC20487_3825 MFSIKKMLQNHYGIDSVNISPEQGGWTALAYQVTDGEAAFFLKVYDKNRASTAKWAALID 60

CHCC14568_0476 MFSIKKMLQNHYGIDSVNISPEQGGWTALAYQVTDGEAAFFLKVYDKNRASTAKWAALID 60

CHCC20339_0022 MFSIKKMLQNHYGIDSVNISPEQGGWTALAYQVTDGEAAFFLKVYDKNRASTAKWAALID 60

CHCC15325_0414 MFSIKKMLQNHYGIDSVNISPEQGGWTALAYQVTDGEAAFFLKVYDKNRASTAKWAALID 60

CHCC14688_3357 MFSIKKMLQNHYGIDSVNISPEQGGWTALAYQVTDGEAAFFLKVYDKNRASTAKWAALID 60

CHCC20442_1307 MFSIKKMLQNHYGIDSVNISPEQGGWTALAYQVTDGEAAFFLKVYDKNRASTAKWAALID 60

CHCC14815_0972 MFSIKKMLQNHYGIDSVNISPEQGGWTALAYQVTDGEAAFFLKVYDKNRASTAKWAALID 60

CHCC20368_2018 MFSIKKMLQNHYGIDSVNISPEQGGWTALAYQVTDGEAAFFLKVYDKNRASTAKWAALID 60

CHCC14562_0763 MFSIKKMLQNHYGIDSVNISPEQGGWTALAYQVTDGEAAFFLKVYDKNRASTAKWAALID 60

CHCC15320_2308 MFSIKKMLQNHYGIDSVNISPEQGGWTALAYQVTDGEAAFFLKVYDKNRASTAKWAALID 60

CHCC20369_2216 MFSIKKMLQNHYGIDSVNISPEQGGWTALAYQVTDGEAAFFLKVYDKNRASTAKWAALID 60

CHCC14431_3646 MFSIKKMLQNHYGIDSVNISPEQGGWTALAYQVTDGEAAFFLKVYDKNRASTAKWAALID 60

CHCC15087_0206 MFSIKKMLQNHYGIDSVNISPEQGGWTALAYQVTDGEAAFFLKVYDKNRASTAKWAALID 60

CHCC14809_3059 MFSIKKMLQNHYGIDSVNISPEQGGWTALAYQVTDGEAAFFLKVYDKNRASTAKWAALID 60

CHCC14596_0535 MFSIKKMLQNHYGIDSVNISPEQGGWTALAYQVTDGEAAFFLKVYDKNRASTAKWAALID 60

CHCC15075_1355 MFSIKKMLQNHYGIDSVNISPEQGGWTALAYQVTDGEAAFFLKVYDKNRASTAKWAALID 60

DSM13_0193 MFSIKKMLQNHYGIDSVNISPEQGGWTALAYQVTDGEAAFFLKVYDKNRASTAKWAALID 60

CHCC14561_2621 MFSIKKMLQNHYGIDSVNISPEQGGWTALAYQVTDGEAAFFLKVYDKNRASTAKWAALID 60

CHCC15292_0696 MFSIKKMLQNHYGIDSVNISPEQGGWTALAYQVTDGEAAFFLKVYDKNRASTAKWAALID 60

CHCC14818_3873 MFSIKKMLQNHYGIDSVNISPEQGGWTALAYQVTDGEAAFFLKVYDKNRASTAKWAALID 60

CHCC14816_0250 MFSIKKMLQNHYGIDSVNISPEQGGWTALAYQVTDGEAAFFLKVYDKNRASTAKWAALID 60

CHCC5025_0547 MFSIKKMLQNHYGIDSVNISPEQGGWTALAYQVTDGEAAFFLKVYDKNRASTAKWAALID 60

CHCC20327_1736 MFSIKKMLQDHYGIDSVNISPEQGGWTALAYQVTDGEAAFFLKVYDKNRASTAKWAALID 60

CHCC14814_3093 MFSLKKILKDHYGIDSVNISPEQGGWTALAYQVTDGEAAFFLKVYDKNRASTAKWTALID 60

CHCC15381_2837 MFSLKKILKGHYGIDSVNISPEQGGWTALAYQVTDGKAAFFLKVYDKNRASTAKWTALID 60

CHCC19467_4581 MFSLKKILKGHYGIDSVNISPEQGGWTALAYQVTDGKAAFFLKVYDKNRASTAKWTALID 60

CHCC19468_2702 MFSLKKILKGHYGIDSVNISPEQGGWTALAYQVTDGKAAFFLKVYDKNRASTAKWTALID 60

CHCC20497_4636 MFSLKKILKGHYGIDSVNISPEQGGWTALAYQVTDGKAAFFLKVYDKNRASTAKWTALID 60

CHCC20492_2744 MFSLKKILKGHYGIDSVNISPEQGGWTALAYQVTDGKAAFFLKVYDKNRASTAKWTALID 60

CHCC14523_3915 MFSLKKILKGHYGIDSVNISPEQGGWTALAYQVTDGKAAFFLKVYDKNRASTAKWTALID 60

CHCC14527_2373 MFSLKKILKGHYGIDSVNISPEQGGWTALAYQVTDGKAAFFLKVYDKNRASTAKWTALID 60

ATCC9945A_0221 MFSLKKILKGHYGIDSVNISPEQGGWTALAYQVTDGKAAFFLKVYDKNRASTAKWTALID 60

CHCC5019_4455 MFSLKKILKGHYGIDSVNISPEQGGWTALAYQVTDGKAAFFLKVYDKNRASTAKWTALID 60

CHCC15337_4611 MFSLKKILKGHYGIDSVNISPEQGGWTALAYQVTDGKAAFFLKVYDKNRASTAKWTALID 60

CHCC15332_3736 MFSLKKILKGHYGIDSVNISPEQGGWTALAYQVTDGKAAFFLKVYDKNRASTAKWTALID 60

CHCC5023_1980 MFSLKKILKGHYGIDSVNISPEQGGWTALAYQVTDGKAAFFLKVYDKNRASTAKWTALID 60

CHCC5021_2116 MFSLKKILKGHYGIDSVNISPEQGGWTALAYQVTDGKAAFFLKVYDKNRASTAKWTALID 60

CHCC20488_0915 MFSLKKILKGHYGIDSVNISPEQGGWTALAYQVTDGKAAFFLKVYDKNRASTAKWTALID 60

CHCC20331_1438 MFSLKKILKGHYGIDSVNISPEQGGWTALAYQVTDGKAAFFLKVYDKNRASTAKWTALID 60

CHCC20490_1917 MFSLKKILKEHYGIDSVNISPEQGGWTALAYQVTDGKAAFFLKVYDKNRASTAKWTALID 60

CHCC20347_3230 MFSLKKILKEHYGIDSVNISPEQGGWTALAYQVTDGKAAFFLKVYDKNRASTAKWTALID 60

CHCC14820_1470 MFSLKKILKEHYGIDSVNISPEQGGWTALAYQVTDGKAAFFLKVYDKNRASTAKWTALID 60

CHCC20372_2719 MFSLKKILKEHYGIDSVNISPEQGGWTALAYQVTDGKAAFFLKVYDKNRASTAKWTALID 60

CHCC20348_4390 MFSLKKILKEHYGIDSVNISPEQGGWTALAYQVTDGKAAFFLKVYDKNRASTAKWTALID 60

CHCC5027_3367 MFSLKKILKEHYGIDSVNISPEQGGWTALAYQVTDGKAAFFLKVYDKNRASTAKWTALID 60

CHCC12620_0738 MFSLKKILKGHYGIDSVNISPEQGGWTALAYQVTDGKAAFFLKVYDKNRASTAKWTALID 60

CHCC20491_0560 MFSLKKILKGHYGIDSVNISPEQGGWTALAYQVTDGKAAFFLKVYDKNRASTAKWTALID 60

CHCC15136_3272 MFSLKKILKGHYGIDSVNISPEQGGWTALAYQVTDGKAAFFLKVYDKNRASTAKWTALID 60

CHCC5022_3898 MFSLKKILKGHYGIDSVNISPEQGGWTALAYQVTDGKAAFFLKVYDKNRASTAKWTALID 60

CHCC14817_1576 MFSLKKILKGHYGIDSVNISPEQGGWTALAYQVTDGKAAFFLKVYDKNRASTAKWTALID 60

CHCC4186_2036 MFSLKKILKGHYGIDSVNISPEQGGWTALAYQVTDGKAAFFLKVYDKNRASTAKWTALID 60

BL09_0220 MFSLKKILKGHYGIDSVNISPEQGGWTALAYQVTDGKAAFFLKVYDKNRASTAKWTALID 60

CHCC20333_3257 MFSLKKILKEHYGIDSVNISPEQGGWTALAYQVTDGKAAFFLKVYDKNRASTAKWTALID 60

CHCC20375_3121 MFSLKKILKDHYGIDSVNISPEQGGWTALAYQVTDGEAAFFLKVYDKNRASTSKWTALID 60

***:**:*: **************************:***************:**:****

CHCC10893_2819 NYIPVLGWLGDHTALNGRLPVPLLTTAGEYKCEDDDAVYMLYEYIAGETIGDQPLGKGCV 120

CHCC19466_3946 NYIPVLGWLGDHTALNGRLPVPLLTTAGEYKCEDDDAVYMLYEYIAGETIGDQPLGKGCV 120

CHCC20494_1490 NYIPVLGWLGDHTALNGRLPVPLLTTAGEYKCEDDDAVYMLYEYIAGETIGDQPLGKGCV 120

CHCC20493_0356 NYIPVLGWLGDHTALNGRLPVPLLTTAGEYKCEDDDAVYMLYEYIAGETIGDQPLGKGCV 120

CHCC14437_0389 NYIPVLGWLGDHTALNGRLPVPLLTTAGEYKCEDDDAVYMLYEYIAGETIGDQPLGKGCV 120

CHCC20323_0097 NYIPVLGWLGDHTALNGRLPVPLLTTAGEYKCEDDDAVYMLYEYIAGETIGDQPLGKGCV 120

CHCC15318_3692 NYIPVLGWLGDHTALNGRLPVPLLTTAGEYKCEDDDAVYMLYEYIAGETIGDQPLGKGCV 120

CHCC20486_2039 NYIPVLGWLGDHTALNGRLPVPLLTTAGEYKCEDDDAVYMLYEYIAGETIGDQPLGKGCV 120

CHCC14441_1577 NYIPVLGWLGDHTALNGRLPVPLLTTAGEYKCEDDDAVYMLYEYIAGETIGDQPLGKGCV 120

CHCC14525_3597 NYIPVLGWLGDHTALNGRLPVPLLTTAGEYKCEDDDAVYMLYEYIAGETIGDQPLGKGCV 120

CHCC15322_3644 NYIPVLGWLGDHTALNGRLPVPLLTTAGEYKCEDDDAVYMLYEYIAGETIGDQPLGKGCV 120

CHCC15139_1360 NYIPVLGWLGDHTALNGRLPVPLLTTAGEYKCEDDDAVYMLYEYIAGETIGDQPLGKGCV 120

CHCC20344_4474 NYIPVLGWLGDHTALNGRLPVPLLTTAGEYKCEDDDAVYMLYEYIAGETIGDQPLGKGCV 120

CHCC20341_2337 NYIPVLGWLGDHTALNGRLPVPLLTTAGEYKCEDDDAVYMLYEYIAGETIGDQPLGKGCV 120

CHCC20343_0034 NYIPVLGWLGDHTALNGRLPVPLLTTAGEYKCEDDDAVYMLYEYIAGETIGDQPLGKGCV 120

CHCC20342_1143 NYIPVLGWLGDHTALNGRLPVPLLTTAGEYKCEDDDAVYMLYEYIAGETIGDQPLGKGCV 120

CHCC14600_3226 NYIPVLGWLGDHTALNGRLPVPLLTTAGEYKCEDDDAVYMLYEYIAGETIGDQPLGKGCV 120

CHCC5020_0927 NYIPVLGWLGDHTALNGRLPVPLLTTAGEYKCEDDDAVYMLYEYIAGETIGDQPLGKGCV 120

CHCC5026_1707 NYIPVLGWLGDHTALNGRLPVPLLTTAGEYKCEDDDAVYMLYEYIAGETIGDQPLGKGCV 120

CHCC14813_2855 NYIPVLGWLGDHTALNGRLPVPLLTTAGEYKCEDDDAVYMLYEYIAGETIGDQPLGKGCV 120

CHCC14810_4313 NYIPVLGWLGDHTALNGRLPVPLLTTAGEYKCEDDDAVYMLYEYIAGETIGDQPLGKGCV 120

CHCC14598_1059 NYIPVLGWLGDHTALNGRLPVPLLTTAGEYKCEDDDAVYMLYEYIAGETIGDQPLGKGCV 120

CHCC15291_0393 NYIPVLGWLGDHTALNGRLPVPLLTTAGEYKCEDDDAVYMLYEYIAGETIGDQPLGKGCV 120

CHCC15290_3000 NYIPVLGWLGDHTALNGRLPVPLLTTAGEYKCEDDDAVYMLYEYIAGETIGDQPLGKGCV 120

CHCC14564_2766 NYIPVLGWLGDHTALNGRLPVPLLTTAGEYKCEDDDAVYMLYEYIAGETIGDQPLGKGCV 120

CHCC20373_0249 NYIPVLGWLGDHTALNGRLPVPLLTTAGEYKCEDDDAVYMLYEYIAGETIGDQPLGKGCV 120

CHCC15289_3954 NYIPVLGWLGDHTALNGRLPVPLLTTAGEYKCEDDDAVYMLYEYIAGETIGDQPLGKGCV 120

CHCC20325_3728 NYIPVLGWLGDHTALNGRLPVPLLTTAGEYKCEDDDAVYMLYEYIAGETIGDQPLGKGCV 120

CHCC20440_1829 NYIPVLGWLGDHTALNGRLPVPLLTTAGEYKCEDDDAVYMLYEYIAGETIGDQPLGKGCV 120

CHCC20441_1081 NYIPVLGWLGDHTALNGRLPVPLLTTAGEYKCEDDDAVYMLYEYIAGETIGDQPLGKGCV 120

CHCC15546_0034 DYIPVLGWLGDHTALNGRLPVPLLTTAGEYKCEDDDAVYMLYEYIAGETIGDQPLGKGCV 120

CHCC15543_3601 DYIPVLGWLGDHTALNGRLPVPLLTTAGEYKCEDDDAVYMLYEYIAGETIGDQPLGKGCV 120

CHCC16874_4693 DYIPVLGWLGDHTALNGRLPVPLLTTAGEYKCEDDDAVYMLYEYIAGETIGDQPLGKGCV 120

CHCC16736_1643 DYIPVLGWLGDHTALNGRLPVPLLTTAGEYKCEDDDAVYMLYEYIAGETIGDQPLGKGCV 120

CHCC20495_0877 DYIPVLGWLGDHTALNGRLPVPLLTTAGEYKCEDDDAVYMLYEYIAGETIGDQPLGKGCV 120

CHCC14557_3891 DYIPVLGWLGDHTALNGRLPVPLLTTAGEYKCEDDDAVYMLYEYIAGETIGDQPLGKGCV 120

CHCC15315_4194 DYIPVLGWLGDHTALNGRLPVPLLTTAGEYKCEDDDAVYMLYEYIAGETIGDQPLGKGCV 120

CHCC15311_0625 DYIPVLGWLGDHTALNGRLPVPLLTTAGEYKCEDDDAVYMLYEYIAGETIGDQPLGKGCV 120

CHCC20345_4265 DYIPVLGWLGDHTALNGRLPVPLLTTAGEYKCEDDDAVYMLYEYIAGETIGDQPLGKGCV 120

CHCC15335_1763 DYIPVLGWLGDHTALNGRLPVPLLTTAGEYKCEDDDAVYMLYEYIAGETIGDQPLGKGCV 120

CHCC14808_3670 DYIPVLGWLGDHTALNGRLPVPLLTTAGEYKCEDDDAVYMLYEYIAGETIGDQPLGKGCV 120

CHCC5024_4270 DYIPVLGWLGDHTALNGRLPVPLLTTAGEYKCEDDDAVYMLYEYIAGETIGDQPLGKGCV 120

CHCC14819_0398 DYIPVLGWLGDHTALNGRLPVPLLTTAGEYKCEDDDAVYMLYEYIAGETIGDQPLGKGCV 120

CHCC14429_3014 DYIPVLGWLGDHTALNGRLPVPLLTTAGEYKCEDDDAVYMLYEYIAGETIGDQPLGKGCV 120

CHCC15091_2902 DYIPVLGWLGDHTALNGRLPVPLLTTAGEYKCEDDDAVYMLYEYIAGETIGDQPLGKGCV 120

CHCC20496_3909 DYIPVLGWLGDHTALNGRLPVPLLTTAGEYKCEDDDAVYMLYEYIAGETIGDQPLGKGCV 120

CHCC14559_1464 DYIPVLGWLGDHTALNGRLPVPLLTTAGEYKCEDDDAVYMLYEYIAGETIGDQPLGKGCV 120

CHCC14566_2258 DYIPVLGWLGDHTALNGRLPVPLLTTAGEYKCEDDDAVYMLYEYIAGETIGDQPLGKGCV 120

CHCC14435_2913 DYIPVLGWLGDHTALNGRLPVPLLTTAGEYKCEDDDAVYMLYEYIAGETIGDQPLGKGCV 120

CHCC20489_0346 DYIPVLGWLGDHTALNGRLPVPLLTTAGEYKCEDDDAVYMLYEYIAGETIGDQPLGKGCV 120

CHCC20487_3825 DYIPVLGWLGDHTALNGRLPVPLLTTAGEYKCEDDDAVYMLYEYIAGETIGDQPLGKGCV 120

CHCC14568_0476 DYIPVLGWLGDHTALNGRLPVPLLTTAGEYKCEDDDAVYMLYEYIAGETIGDQPLGKGCV 120

CHCC20339_0022 DYIPVLGWLGDHTALNGRLPVPLLTTAGEYKCEDDDAVYMLYEYIAGETIGDQPLGKGCV 120

CHCC15325_0414 DYIPVLGWLGDHTALNGRLPVPLLTTAGEYKCEDDDAVYMLYEYIAGETIGDQPLGKGCV 120

CHCC14688_3357 DYIPVLGWLGDHTALNGRLPVPLLTTAGEYKCEDDDAVYMLYEYIAGETIGDQPLGKGCV 120

CHCC20442_1307 DYIPVLGWLGDHTALNGRLPVPLLTTAGEYKCEDDDAVYMLYEYIAGETIGDQPLGKGCV 120

CHCC14815_0972 DYIPVLGWLGDHTALNGRLPVPLLTTAGEYKCEDDDAVYMLYEYIAGETIGDQPLGKGCV 120

CHCC20368_2018 DYIPVLGWLGDHTALNGRLPVPLLTTAGEYKCEDDDAVYMLYEYIAGETIGDQPLGKGCV 120

CHCC14562_0763 DYIPVLGWLGDHTALNGRLPVPLLTTAGEYKCEDDDAVYMLYEYIAGETIGDQPLGKGCV 120

CHCC15320_2308 DYIPVLGWLGDHTALNGRLPVPLLTTAGEYKCEDDDAVYMLYEYIAGETIGDQPLGKGCV 120

CHCC20369_2216 DYIPVLGWLGDHTALNGRLPVPLLTTAGEYKCEDDDAVYMLYEYIAGETIGDQPLGKGCV 120

CHCC14431_3646 DYIPVLGWLGDHTALNGRLPVPLLTTAGEYKCEDDDAVYMLYEYIAGETIGDQPLGKGCV 120

CHCC15087_0206 DYIPVLGWLGDHTALNGRLPVPLLTTAGEYKCEDDDAVYMLYEYIAGETIGDQPLGKGCV 120

CHCC14809_3059 DYIPVLGWLGDHTALNGRLPVPLLTTAGEYKCEDDDAVYMLYEYIAGETIGDQPLGKGCV 120

CHCC14596_0535 DYIPVLGWLGDHTALNGRLPVPLLTTAGEYKCEDDDAVYMLYEYIAGETIGDQPLGKGCV 120

CHCC15075_1355 DYIPVLGWLGDHTALNGRLPVPLLTTAGEYKCEDDDAVYMLYEYIAGETIGDQPLGKGCV 120

DSM13_0193 DYIPVLGWLGDHTALNGRLPVPLLTTAGEYKCEDDDAVYMLYEYIAGETIGDQPLGKGCV 120

CHCC14561_2621 DYIPVLGWLGDHTALNGRLPVPLLTTAGEYKCEDDDAVYMLYEYIAGETIGDQPLGKGCV 120

CHCC15292_0696 DYIPVLGWLGDHTALNGRLPVPLLTTAGEYKCEDDDAVYMLYEYIAGETIGDQPLGKGCV 120

CHCC14818_3873 DYIPVLGWLGDHTALNGRLPVPLLTTAGEYKCEDDDAVYMLYEYIAGETIGDQPLGKGCV 120

CHCC14816_0250 DYIPVLGWLGDHTALNGRLPVPLLTTAGEYKCEDDDAVYMLYEYIAGETIGDQPLGKGCV 120

CHCC5025_0547 DYIPVLGWLGDHTALNGRLPVPLLTTAGEYKCEDDDAVYMLYEYIAGETIGDQPLGKGCV 120

CHCC20327_1736 DYIPVLGWLGDHTALNGRLPVPLLTTAGEYKCEDDDAVYMLYEYIAGETIGDQPLGKGCV 120

CHCC14814_3093 DYIPVLKWLGDHTALNGKLPVPLLTTTGEYKCEDDDAVYVLYEYIAGETIGGRPLDKGCA 120

CHCC15381_2837 DYMPVLRWLSDRTALSGKLPVPLLTTNGEYKCEDDDAVYVLYEYIAGETIGGRPLGKGCE 120

CHCC19467_4581 DYMPVLRWLSDRTALSGKLPVPLLTTNGEYKCEDDDAVYVLYEYIAGETIGGRPLGKGCE 120

CHCC19468_2702 DYMPVLRWLSDRTALSGKLPVPLLTTNGEYKCEDDDAVYVLYEYIAGETIGGRPLGKGCE 120

CHCC20497_4636 DYMPVLRWLSDRTALSGKLPVPLLTTNGEYKCEDDDAVYVLYEYIAGETIGGRPLGKGCE 120

CHCC20492_2744 DYMPVLRWLSDRTALSGKLPVPLLTTNGEYKCEDDDAVYVLYEYIAGETIGGRPLGKGCE 120

CHCC14523_3915 DYMPVLRWLSDRTALSGKLPVPLLTTNGEYKCEDDDAVYVLYEYIAGETIGGRPLGKGCE 120

CHCC14527_2373 DYMPVLRWLSDRTALSGKLPVPLLTTNGEYKCEDDDAVYVLYEYIAGETIGGRPLGKGCE 120

ATCC9945A_0221 DYMPVLRWLSDRTALSGKLPVPLLTTNGEYKCEDDDAVYVLYEYIAGETIGGRPLGKGCE 120

CHCC5019_4455 DYMPVLRWLSDRTALSGKLPVPLLTTNGEYKCEDDDAVYVLYEYIAGETIGGRPLGKGCE 120

CHCC15337_4611 DYMPVLRWLSDRTALSGKLPVPLLTTNGEYKCEDDDAVYVLYEYIAGETIGGRPLGKGCE 120

CHCC15332_3736 DYMPVLRWLSDRTALSGKLPVPLLTTNGEYKCEDDDAVYVLYEYIAGETIGGRPLGKGCE 120

CHCC5023_1980 DYMPVLRWLSDRTALSGKLPVPLLTTNGEYKCEDDDAVYVLYEYIAGETIGGRPLGKGCE 120

CHCC5021_2116 DYMPVLRWLSDRTALSGKLPVPLLTTNGEYKCEDDDAVYVLYEYIAGETIGGRPLGKGCE 120

CHCC20488_0915 DYMPVLRWLSDRTALSGKLPVPLLTTNGEYKCEDDDAVYVLYEYIAGETIGGRPLGKGCE 120

CHCC20331_1438 DYMPVLRWLSDRTALSGKLPVPLLTKNGEYKCEDDDAVYVLYEYIAGETIGGRPLGKGCA 120

CHCC20490_1917 DYMPVLRWLSDRTALSGKLPVPLLTTNGEYKCEDDDAVYVLYEYIAGETIGGRPLGKGCA 120

CHCC20347_3230 DYMPVLRWLSDRTALSGKLPVPLLTTNGEYKCEDDDAVYVLYEYIAGETIGGRPLGKGCA 120

CHCC14820_1470 DYMPVLRWLSDRTALSGKLPVPLLTTNGEYKCEDDDAVYVLYEYIAGETIGGRPLGKGCA 120

CHCC20372_2719 DYMPVLRWLSDRTALSGKLPVPLLTTNGEYKCEDDDAVYVLYEYIAGETIGGRPLGKGCA 120

CHCC20348_4390 DYMPVLRWLSDRTALSGKLPVPLLTTNGEYKCEDDDAVYVLYEYIAGETIGGRPLGKGCE 120

CHCC5027_3367 DYMPVLRWLSDRTALSGKLPVPLLTTNGEYKCEDDDAVYVLYEYIAGETIGGRPLGKGCE 120

CHCC12620_0738 DYMPVLRWLSDRTALSGKLPVPLLTTNGEYKCEDDDAVYVLYEYIAGETIGGRPLGKGCE 120

CHCC20491_0560 DYMPVLRWLSDRTALSGKLPVPLLTTNGEYKCEDDDAVYVLYEYIAGETIGGRPLGKGCE 120

CHCC15136_3272 DYMPVLRWLSDRTALSGKLPVPLLTTNGEYKCEDDDAVYVLYEYIAGETIGGRPLGKGCE 120

CHCC5022_3898 DYMPVLRWLSDRTALSGKLPVPLLTTNGEYKCEDDDAVYVLYEYIAGETIGGRPLGKGCE 120

CHCC14817_1576 DYMPVLRWLSDRTALSGKLPVPLLTTNGEYKCEDDDAVYVLYEYIAGETIGGRPLGKGCE 120

CHCC4186_2036 DYMPVLRWLSDRTALSGKLPVPLLTTNGEYKCEDDDAVYVLYEYIAGETIGGRPLGKGCE 120

BL09_0220 DYMPVLRWLSDRTALSGKLPVPLLTTNGEYKCEDDDAVYVLYEYIAGETIGGRPLGKGCE 120

CHCC20333_3257 DYMPVLRWLSDRTALSGKLPVPLLTTNGEYKCEDDDAVYVLYEYIAGETIGGRPLGKGCE 120

CHCC20375_3121 DYMPVLRWLRDHTALNGKLPVPLLTTTGEYKCEDEDAVYVLYEYIAGETIGDQPLGKGSV 120

:*:*** ** *:***.*:*******. *******:****:***********.:**.**.

CHCC10893_2819 EKLAGIIAELHRYDETIPVKTEGIKEKYDISFLDEMTQWLDHLPCELATLIQPYSGAIRD 180

CHCC19466_3946 EKLAGIIAELHRYDETIPVKTEGIKEKYDISFLDEMTQWLDHLPCELATLIQPYSGAIRD 180

CHCC20494_1490 EKLAGIIAELHRYDETIPVKTEGIKEKYDISFLDEMTQWLDHLPCELATLIQPYSGAIRD 180

CHCC20493_0356 EKLAGIIAELHRYDETIPVKTEGIKEKYDISFLDEMTQWLDHLPCELATLIQPYSGAIRD 180

CHCC14437_0389 EKLAGIIAELHRYDETIPVKTEGIKEKYDISFLDEMTQWLDHLPCELATLIQPYSGAIRD 180

CHCC20323_0097 EKLAGIIAELHRYDETIPVKTEGIKEKYDISFLDEMTQWLDHLPCELATLIQPYSGAIRD 180

CHCC15318_3692 EKLAGIIAELHRYDETIPVKTEGIKEKYDISFLDEMTQWLDHLPCELATLIQPYSGAIRD 180

CHCC20486_2039 EKLAGIIAELHRYDETIPVKTEGIKEKYDISFLDEMTQWLDHLPCELATLIQPYSGAIRD 180

CHCC14441_1577 EKLAGIIAELHRYDETIPVKTEGIKEKYDISFLDEMTQWLDHLPCELATLIQPYSGAIRD 180

CHCC14525_3597 EKLAGIIAELHRYDETIPVKTEGIKEKYDISFLDEMTQWLDHLPCELATLIQPYSGAIRD 180

CHCC15322_3644 EKLAGIIAELHRYDETIPVKTEGIKEKYDISFLDEMTQWLDHLPCELATLIQPYSGAIRD 180

CHCC15139_1360 EKLAGIIAELHRYDETIPVKTEGIKEKYDISFLDEMTQWLDHLPCELATLIQPYSGAIRD 180

CHCC20344_4474 EKLAGIIAELHRYDETIPVKTEGIKEKYDISFLDEMTQWLDHLPCELATLIQPYSGAIRD 180

CHCC20341_2337 EKLAGIIAELHRYDETIPVKTEGIKEKYDISFLDEMTQWLDHLPCELATLIQPYSGAIRD 180

CHCC20343_0034 EKLAGIIAELHRYDETIPVKTEGIKEKYDISFLDEMTQWLDHLPCELATLIQPYSGAIRD 180

CHCC20342_1143 EKLAGIIAELHRYDETIPVKTEGIKEKYDISFLDEMTQWLDHLPCELATLIQPYSGAIRD 180

CHCC14600_3226 EKLAGIIAELHRYDETIPVKTEGIKEKYDISFLDEMTQWLDHLPCELATLIQPYSGAIRD 180

CHCC5020_0927 EKLAGIIAELHRYDETIPVKTEGIKEKYDISFLDEMTQWLDHLPCELATLIQPYSGAIRD 180

CHCC5026_1707 EKLAGIIAELHRYDETIPVKTEGIKEKYDISFLDEMTQWLDHLPCELATLIQPYSGAIRD 180

CHCC14813_2855 EKLAGIIAELHRYDETIPVKTEGIKEKYDISFLDEMTQWLDHLPCELATLIQPYSGAIRD 180

CHCC14810_4313 EKLAGIIAELHRYDETIPVKTEGIKEKYDISFLDEMTQWLDHLPCELATLIQPYSGAIRD 180

CHCC14598_1059 EKLAGIIAELHRYDETIPVKTEGIKEKYDISFLDEMTQWLDHLPCELATLIQPYSGAIRD 180

CHCC15291_0393 EKLAGIIAELHRYDETIPVKTEGIKEKYDISFLDEMTQWLDHLPCELATLIQPYSGAIRD 180

CHCC15290_3000 EKLAGIIAELHRYDETIPVKTEGIKEKYDISFLDEMTQWLDHLPCELATLIQPYSGAIRD 180

CHCC14564_2766 EKLAGIIAELHRYDETIPVKTEGIKEKYDISFLDEMTQWLDHLPCELATLIQPYSGAIRD 180

CHCC20373_0249 EKLAGIIAELHRYDETIPVKTEGIKEKYDISFLDEMTQWLDHLPCELATLIQPYSGAIRD 180

CHCC15289_3954 EKLAGIIAELHRYDETIPVKTEGIKEKYDISFLDEMTQWLDHLPCELATLIQPYSGAIRD 180

CHCC20325_3728 EKLAGIIAELHRYDETIPVKTEGIKEKYDISFLDEMTQWLDHLPCELATLIQPYSGAIRD 180

CHCC20440_1829 EKLAGIIAELHRYDETIPVKTEGIKEKYDISFLDEMTQWLDHLPCELATLIQPYSGAIRD 180

CHCC20441_1081 EKLAGIIAELHRYDETIPVKTEGIKEKYDISFLDEMTQWLDHLPCELATLIQPYSGAIRD 180

CHCC15546_0034 EKLAGIIAELHRYDETIPVKTEGIKEKYDISFLDEMTQWLDHLPCELATLIQPYSGAIRD 180

CHCC15543_3601 EKLAGIIAELHRYDETIPVKTEGIKEKYDISFLDEMTQWLDHLPCELATLIQPYSGAIRD 180

CHCC16874_4693 EKLAGIIAELHRYDETIPVKTEGIKEKYDISFLDEMTQWLDHLPCELATLIQPYSGAIRD 180

CHCC16736_1643 EKLAGIIAELHRYDETIPVKTEGIKEKYDISFLDEMTQWLDHLPCELATLIQPYSGAIRD 180

CHCC20495_0877 EKLAGIIAELHRYDETIPVKTEGIKEKYDISFLDEMTQWLDHLPCELATLIQPYSGAIRD 180

CHCC14557_3891 EKLAGIIAELHRYDETIPVKTEGIKEKYDISFLDEMTQWLDHLPCELATLIQPYSGAIRD 180

CHCC15315_4194 EKLAGIIAELHRYDETIPVKTEGIKEKYDISFLDEMTQWLDHLPCELATLIQPYSGAIRD 180

CHCC15311_0625 EKLAGIIAELHRYDETIPVKTEGIKEKYDISFLDEMTQWLDHLPCELATLIQPYSGAIRD 180

CHCC20345_4265 EKLAGIIAELHRYDETIPVKTEGIKEKYDISFLDEMTQWLDHLPCELATLIQPYSGAIRD 180

CHCC15335_1763 EKLAGIIAELHRYDETIPVKTEGIKEKYDISFLDEMTQWLDHLPCELATLIQPYSGAIRD 180

CHCC14808_3670 EKLAGIIAELHRYDETIPVKTEGIKEKYDISFLDEMTQWLDHLPCELATLIQPYSGAIRD 180

CHCC5024_4270 EKLAGIIAELHRYDETIPVKTEGIKEKYDISFLDEMTQWLDHLPCELATLIQPYSGAIRD 180

CHCC14819_0398 EKLAGIIAELHRYDETIPVKTEGIKEKYDISFLDEMTQWLDHLPCELATLIQPYSGAIRD 180

CHCC14429_3014 EKLAGIIAELHRYDETIPVKTEGIKEKYDISFLDEMTQWLDHLPCELATLIQPYSGAIRD 180

CHCC15091_2902 EKLAGIIAELHRYDETIPVKTEGIKEKYDISFLDEMTQWLDHLPCELATLIQPYSGAIRD 180

CHCC20496_3909 EKLAGIIAELHRYDETIPVKTEGIKEKYDISFLDEMTQWLDHLPCELATLIQPYSGAIRD 180

CHCC14559_1464 EKLAGIIAELHRYDETIPVKTEGIKEKYDISFLDEMTQWLDHLPCELATLIQPYSGAIRD 180

CHCC14566_2258 EKLAGIIAELHRYDETIPVKTEGIKEKYDISFLDEMTQWLDHLPCELATLIQPYSGAIRD 180

CHCC14435_2913 EKLAGIIAELHRYDETIPVKTEGIKEKYDISFLDEMTQWLDHLPCELATLIQPYSGAIRD 180

CHCC20489_0346 EKLAGIIAELHRYDETIPVKTEGIKEKYDISFLDEMTQWLDHLPCELATLIQPYSGAIRD 180

CHCC20487_3825 EKLAGIIAELHRYDETIPVKTEGIKEKYDISFLDEMTQWLDHLPCELATLIQPYSGAIRD 180

CHCC14568_0476 EKLAGIIAELHRYDETIPVKTEGIKEKYDISFLDEMTQWLDHLPCELATLIQPYSGAIRD 180

CHCC20339_0022 EKLAGIIAELHRYDETIPVKTEGIKEKYDISFLDEMTQWLDHLPCELATLIQPYSGAIRD 180

CHCC15325_0414 EKLAGIIAELHRYDETIPVKTEGIKEKYDISFLDEMTQWLDHLPCELATLIQPYSGAIRD 180

CHCC14688_3357 EKLAGIIAELHRYDETIPVKTEGIKEKYDISFLDEMTQWLDHLPCELATLIQPYSGAIRD 180

CHCC20442_1307 EKLAGIIAELHRYDETIPVKTEGIKEKYDISFLDEMTQWLDHLPCELATLIQPYSGAIRD 180

CHCC14815_0972 EKLAGIIAELHRYDETIPVKTEGIKEKYDISFLDEMTQWLDHLPCELATLIQPYSGAIRD 180

CHCC20368_2018 EKLAGIIAELHRYDETIPVKTEGIKEKYDISFLDEMTQWLDHLPCELATLIQPYSGAIRD 180

CHCC14562_0763 EKLAGIIAELHRYDETIPVKTEGIKEKYDISFLDEMTQWLDHLPCELATLIQPYSGAIRD 180

CHCC15320_2308 EKLAGIIAELHRYDETIPVKTEGIKEKYDISFLDEMTQWLDHLPCELATLIQPYSGAIRD 180

CHCC20369_2216 EKLAGIIAELHRYDETIPVKTEGIKEKYDISFLDEMTQWLDHLPCELATLIQPYSGAIRD 180

CHCC14431_3646 EKLAGIIAELHRYDETIPVKTEGIKEKYDISFLDEMTQWLDHLPCELATLVQPYSGAIRD 180

CHCC15087_0206 EKLAGIIAELHRYDETIPVKTEGIKEKYDISFLDEMTQWLDHLPCELATLVQPYSGAIRD 180

CHCC14809_3059 EKLAGIIAELHRYDETIPVKTEGIKEKYDISFLDEMTQWLDHLPCELATLVQPYSGAIRD 180

CHCC14596_0535 EKLAGIIAELHRYDETIPVKTEGIKEKYDISFLDEMTQWLDHLPCELATLVQPYSGAIRD 180

CHCC15075_1355 EKLAGIIAELHRYDETIPVKTEGIKEKYDISFLDEMTQWLDHLPCELATLIQPYSGAIRD 180

DSM13_0193 EKLAGIIAELHRYDETIPVKTEGIKEKYDISFLDEMTQWLDHLPCELATLIQPYSGAIRD 180

CHCC14561_2621 EKLAGIIAELHRYDETIPVKTEGIKEKYDISFLDEMTQWLDHLPCELATLIQPYSGAIRD 180

CHCC15292_0696 EKLAGIIAELHRYDETIPVKTEGIKEKYDISFLDEMTQWLDHLPCELATLIQPYSGAIRD 180

CHCC14818_3873 EKLAGIIAELHRYDETIPVKTEGIKEKYDISFLDEMTQWLDHLPCELATLIQPYSGAIRD 180

CHCC14816_0250 EKLAGIIAELHRYDETIPVKTEGIKEKYDISFLDEMTQWLDHLPCELATLIQPYSGAIRD 180

CHCC5025_0547 EKLAGIIAELHRYDETIPVKTEGIKEKYDISFLDEMTQWLDHLPCELATLIQPYSGAIRD 180

CHCC20327_1736 EKLAGIIAELHRYDETIPVKTEGIKEKYDISFLDEMTQWLDHLPCELATLIQPYSGAIRD 180

CHCC14814_3093 EKLASIIAELHRYDETIPLKTEGIKENYDISFLGKMKQWLDHLPCELATLIQPYSGAIRE 180

CHCC15381_2837 EKLASIIAELHRYDETIPVKTERIKENYDISFLGEMKQWLDHLPCELAPLIQPYIGSIRE 180

CHCC19467_4581 EKLASIIAELHRYDETIPVKTERIKENYDISFLGEMKQWLDHLPCELAPLIQPYIGSIRE 180

CHCC19468_2702 EKLASIIAELHRYDETIPVKTERIKENYDISFLGEMKQWLDHLPCELAPLIQPYIGSIRE 180

CHCC20497_4636 EKLASIIAELHRYDETIPVKTERIKENYDISFLGEMKQWLDHLPCELAPLIQPYIGSIRE 180

CHCC20492_2744 EKLASIIAELHRYDETIPVKTERIKENYDISFLGEMKQWLDHLPCELAPLIQPYIGSIRE 180

CHCC14523_3915 EKLASIIAELHRYDETIPVKTERIKENYDISFLGEMKQWLDHLPCELAPLIQPYIGSIRE 180

CHCC14527_2373 EKLASIIAELHRYDETIPVKTERIKENYDISFLGEMKQWLDHLPCELAPLIQPYIGSIRE 180

ATCC9945A_0221 EKLASIIAELHRYDETIPVKTERIKENYDISFLGEMKQWLDHLPCELAPLIQPYIGSIRE 180

CHCC5019_4455 EKLASIIAELHRYDETIPVKTERIKENYDISFLGEMKQWLDHLPCELAPLIQPYIGSIRE 180

CHCC15337_4611 EKLASIIAELHRYDETIPVKTERIKENYDISFLGEMKQWLDHLPCELAPLIQPYIGSIRE 180

CHCC15332_3736 EKLASIIAELHRYDETIPVKTERIKENYDISFLGEMKQWLDHLPCELAPLIQPYIGSIRE 180

CHCC5023_1980 EKLASIIAELHRYDETIPVKTERIKENYDISFLGEMKQWLDHLPCELAPLIQPYIGSIRE 180

CHCC5021_2116 EKLASIIAELHRYDETIPVKTERIKENYDISFLGEMKQWLDHLPCELAPLIQPYIGSIRE 180

CHCC20488_0915 EKLASIIAELHRYDETIPVKTERIKENYDISFLGEMKQWLDHLPCELAPLIQPYIGSIRE 180

CHCC20331_1438 KKLASIIAELHRYDETIPVKTERIKENYDISFIGEMKQWLDHLPCELAPLIQPYIGSIRE 180

CHCC20490_1917 KKLASIIAELHRYDETIPVKTERIKENYDISFLGEMKQWLDHLPCELAPLIQPYIGSIRE 180

CHCC20347_3230 KKLASIIAELHRYDETIPVKTERIKENYDISFLGEMKQWLDHLPCELAPLIQPYIGSIRE 180

CHCC14820_1470 KKLASIIAELHRYDETIPVKTERIKENYDISFLGEMKQWLDHLPCELAPLIQPYIGSIRE 180

CHCC20372_2719 KKLASIIAELHRYDETIPVKTERIKENYDISFLGEMKQWLDHLPCELAPLIQPYIGSIRE 180

CHCC20348_4390 EKLASIIAELHRYDETIPVKTERIKENYDISFLGEMKQWLDHLPCELAPLIQPYIGSIRE 180

CHCC5027_3367 EKLASIIAELHRYDETIPVKTERIKENYDISFLGEMKQWLDHLPCELAPLIQPYIGSIRE 180

CHCC12620_0738 EKLASIIAELHRYDETIPVKTERIKENYDISFLGEMKQWLDHLPCELAPLIQPYIGSIRE 180

CHCC20491_0560 EKLASIIAELHRYDETIPVKTERIKENYDISFLGEMKQWLDHLPCELAPLIQPYIGSIRE 180

CHCC15136_3272 EKLASIIAELHRYDETIPVKTERIKENYDISFLGEMKQWLDHLPCELAPLIQPYIGSIRE 180

CHCC5022_3898 EKLASIIAELHRYDETIPVKTERIKENYDISFLGEMKQWLDHLPCELAPLIQPYIGSIRE 180

CHCC14817_1576 EKLASIIAELHRYDETIPVKTERIKENYDISFLGEMKQWLDHLPCELAPLIQPYIGSIRE 180

CHCC4186_2036 EKLASIIAELHRYDETIPVKTERIKENYDISFLGEMKQWLDHLPCELAPLIQPYIGSIRE 180

BL09_0220 EKLASIIAELHRYDETIPVKTERIKENYDISFLGEMKQWLDHLPCELAPLIQPYIGSIRE 180

CHCC20333_3257 EKLASIIAELHRYDETIPVKTERIKENYDISFLGEMKQWLDHLPCELAPLIQPYIGSIRE 180

CHCC20375_3121 EKLASIIADLHRYDETIPVETEGIKENYDISFLGEMKQWLDHLPCELASLIQPYIGSIRE 180

:***.***:*********::** ***:*****:.:*.***********.*:*** *:**:

CHCC10893_2819 MMESALSLADDLKCSGRRLALCHTDVHGWN-LMKTGGELILIDWEGLKLAPVEADLMFFA 239

CHCC19466_3946 MMESALSLADDLKCSGRRLALCHTDVHGWN-LMKTGGELILIDWEGLKLAPVEADLMFFA 239

CHCC20494_1490 MMESALSLADDLKCSGRRLALCHTDVHGWN-LMKTGGELILIDWEGLKLAPVEADLMFFA 239

CHCC20493_0356 MMESALSLADDLKCSGRRLALCHTDVHGWN-LMKTGGELILIDWEGLKLAPVEADLMFFA 239

CHCC14437_0389 MMESALSLADDLKCSGRRLALCHTDVHGWN-LMKTGGELILIDWEGLKLAPVEADLMFFA 239

CHCC20323_0097 MMESALSLADDLKCSGRRLALCHTDVHGWN-LMKTGGELILIDWEGLKLAPVEADLMFFA 239

CHCC15318_3692 MMESALSLADDLKCSGRRLALCHTDVHGWN-LMKTGGELILIDWEGLKLAPVEADLMFFA 239

CHCC20486_2039 MMESALSLADDLKCSGRRLALCHTDVHGWN-LMKTGGELILIDWEGLKLAPVEADLMFFA 239

CHCC14441_1577 MMESALSLADDLKCSGRRLALCHTDVHGWN-LMKTGGELILIDWEGLKLAPVEADLMFFA 239

CHCC14525_3597 MMESALSLADDLKCSGRRLALCHTDVHGWN-LMKTGGELILIDWEGLKLAPVEADLMFFA 239

CHCC15322_3644 MMESALSLADDLKCSGRRLALCHTDVHGWN-LMKTGGELILIDWEGLKLAPVEADLMFFA 239

CHCC15139_1360 MMESALSLADDLKCSGRRLALCHTDVHGWN-LMKTGGELILIDWEGLKLAPVEADLMFFA 239

CHCC20344_4474 MMESALSLADDLKCSGRRLALCHTDVHGWN-LMKTGGELILIDWEGLKLAPVEADLMFFA 239

CHCC20341_2337 MMESALSLADDLKCSGRRLALCHTDVHGWN-LMKTGGELILIDWEGLKLAPVEADLMFFA 239

CHCC20343_0034 MMESALSLADDLKCSGRRLALCHTDVHGWN-LMKTGGELILIDWEGLKLAPVEADLMFFA 239

CHCC20342_1143 MMESALSLADDLKCSGRRLALCHTDVHGWN-LMKTGGELILIDWEGLKLAPVEADLMFFA 239

CHCC14600_3226 MMESALSLADDLKCSGRRLALCHTDVHGWN-LMKTGGELILIDWEGLKLAPVEADLMFFA 239

CHCC5020_0927 MMESALSLADDLKCSGRRLALCHTDVHGWN-LMKTGGELILIDWEGLKLAPVEADLMFFA 239

CHCC5026_1707 MMESALSLADDLKCSGRRLALCHTDVHGWN-LMKTGGELILIDWEGLKLAPVEADLMFFA 239

CHCC14813_2855 MMESALSLADDLKCSGRRLALCHTDVHGWN-LMKTGGELILIDWEGLKLAPVEADLMFFA 239

CHCC14810_4313 MMESALSLADDLKCSGRRLALCHTDVHGWN-LMKTGGELILIDWEGLKLAPVEADLMFFA 239

CHCC14598_1059 MMESALSLADDLKCSGRRLALCHTDVHGWN-LMKTGGELILIDWEGLKLAPVEADLMFFA 239

CHCC15291_0393 MMESALSLADDLKCSGRRLALCHTDVHGWN-LMKTGGELILIDWEGLKLAPVEADLMFFA 239

CHCC15290_3000 MMESALSLADDLKCSGRRLALCHTDVHGWN-LMKTGGELILIDWEGLKLAPVEADLMFFA 239

CHCC14564_2766 MMESALSLADDLKCSGRRLALCHTDVHGWN-LMKTGGELILIDWEGLKLAPVEADLMFFA 239

CHCC20373_0249 MMESALSLADDLKCSGRRLALCHTDVHGWN-LMKTGGELILIDWEGLKLAPVEADLMFFA 239

CHCC15289_3954 MMESALSLADDLKCSGRRLALCHTDVHGWN-LMKTGGELILIDWEGLKLAPVEADLMFFA 239

CHCC20325_3728 MMESALSLADDLKCSGRRLALCHTDVHGWN-LMKTGGELILIDWEGLKLAPVEADLMFFA 239

CHCC20440_1829 MMESALSLADDLKCSGRRLALCHTDVHGWN-LMKTGGELILIDWEGLKLAPVEADLMFFA 239

CHCC20441_1081 MMESALSLADDLKCSGRRLALCHTDVHGWN-LMKTGGELILIDWEGLKLAPVEADLMFFA 239

CHCC15546_0034 MMESALSLADDLKCSGRRLALCHTDVHGWN-LMKTGGELILIDWEGLKLAPVEADLMFFA 239

CHCC15543_3601 MMESALSLADDLKCSGRRLALCHTDVHGWN-LMKTGGELILIDWEGLKLAPVEADLMFFA 239

CHCC16874_4693 MMESALSLADDLKCSGRRLALCHTDVHGWN-LMKTGGELILIDWEGLKLAPVEADLMFFA 239

CHCC16736_1643 MMESALSLADDLKCSGRRLALCHTDVHGWN-LMKTGGELILIDWEGLKLAPVEADLMFFA 239

CHCC20495_0877 MMESALSLADDLKCSGRRLALCHTDVHGWN-LMKTGGELILIDWEGLKLAPVEADLMFFA 239

CHCC14557_3891 MMESALSLADDLKCSGRRLALCHTDVHGWN-LMKTGGELILIDWEGLKLAPVEADLMFFA 239

CHCC15315_4194 MMESALSLADDLKCSGRRLALCHTDVHGWN-LMKTGGELILIDWEGLKLAPVEADLMFFA 239

CHCC15311_0625 MMESALSLADDLKCSGRRLALCHTDVHGWN-LMKTGGELILIDWEGLKLAPVEADLMFFA 239

CHCC20345_4265 MMESALSLADDLKCSGRRLALCHTDVHGWN-LMKTGGELILIDWEGLKLAPVEADLMFFA 239

CHCC15335_1763 MMESALSLADDLKCSGRRLALCHTDVHGWN-LMKTGGELILIDWEGLKLAPVEADLMFFA 239

CHCC14808_3670 MMESALSLADDLKCSGRRLALCHTDVHGWN-LMKTGGELILIDWEGLKLAPVEADLMFFA 239

CHCC5024_4270 MMESALSLADDLKCSGRRLALCHTDVHGWN-LMKTGGELILIDWEGLKLAPVEADLMFFA 239

CHCC14819_0398 MMESALSLADDLKCSGRRLALCHTDVHGWN-LMKTGGELILIDWEGLKLAPVEADLMFFA 239

CHCC14429_3014 MMESALSLADDLKCSGRRLALCHTDVHGWN-LMKTGGELILIDWEGLKLAPVEADLMFFA 239

CHCC15091_2902 MMESALSLADDLKCSGRRLALCHTDVHGWN-LMKTGGELILIDWEGLKLAPVEADLMFFA 239

CHCC20496_3909 MMESALSLADDLKCSGRRLALCHTDVHGWN-LMKTGGELILIDWEGLKLAPVEADLMFFA 239

CHCC14559_1464 MMESALSLADDLKCSGRRLALCHTDVHGWN-LMKTGGELILIDWEGLKLAPVEADLMFFA 239

CHCC14566_2258 MMESALSLADDLKCSGRRLALCHTDVHGWN-LMKTGGELILIDWEGLKLAPVEADLMFFA 239

CHCC14435_2913 MMESALSLADDLKCSGRRLALCHTDVHGWN-LMKTGGELILIDWEGLKLAPVEADLMFFA 239

CHCC20489_0346 MMESALSLADDLKCSGRRLALCHTDVHGWN-LMKTGGELILIDWEGLKLAPVEADLMFFA 239

CHCC20487_3825 MMESALSLADDLKCSGRRLALCHTDVHGWN-LMKTGGELILIDWEGLKLAPVEADLMFFA 239

CHCC14568_0476 MMESALSLADDLKCSGRRLALCHTDVHGWN-LMKTGGELILIDWEGLKLAPVEADLMFFA 239

CHCC20339_0022 MMESALSLADDLKCSGRRLALCHTDVHGWN-LMKTGGELILIDWEGLKLAPVEADLMFFA 239

CHCC15325_0414 MMESALSLADDLKCSGRRLALCHTDVHGWN-LMKTGGELILIDWEGLKLAPVEADLMFFA 239

CHCC14688_3357 MMESALSLADDLKCSGRRLALCHTDVHGWN-LMKTGGELILIDWEGLKLAPVEADLMFFA 239

CHCC20442_1307 MMESALSLADDLKCSGRRLALCHTDVHGWN-LMKTGGELILIDWEGLKLAPVEADLMFFA 239

CHCC14815_0972 MMESALSLADDLKCSGRRLALCHTDVHGWN-LMKTGGELILIDWEGLKLAPVEADLMFFA 239

CHCC20368_2018 MMESALSLADDLKCSGRRLALCHTDVHGWN-LMKTGGELILIDWEGLKLAPVEADLMFFA 239

CHCC14562_0763 MMESALSLADDLKCSGRRLALCHTDVHGWN-LMKTGGELILIDWEGLKLAPVEADLMFFA 239

CHCC15320_2308 MMESALSLADDLKCSGRRLALCHTDVHGWN-LMKTGGELILIDWEGLKLAPVEADLMFFA 239

CHCC20369_2216 MMESALSLADDLKCSGRRLALCHTDVHGWN-LMKTGGELILIDWEGLKLAPVEADLMFFA 239

CHCC14431_3646 MMESALSLADDLKCSGRRLALCHTDVHGWN-LMKTGGELILIDWEGLKLAPVEADLMFFA 239

CHCC15087_0206 MMESALSLADDLKCSGRRLALCHTDVHGWN-LMKTGGELILIDWEGLKLAPVEADLMFFA 239

CHCC14809_3059 MMESALSLADDLKCSGRRLALCHTDVHGWN-LMKTGGELILIDWEGLKLAPVEADLMFFA 239

CHCC14596_0535 MMESALSLADDLKCSGRRLALCHTDVHGWN-LMKTGGELILIDWEGLKLAPVEADLMFFA 239

CHCC15075_1355 MMESALSLADDLKCSGRRLALCHTDVHGWN-LMKTGGELILIDWEGLKLAPVEADLMFFA 239

DSM13_0193 MMESALSLADDLKCSGRRLALCHTDVHGWN-LMKTGGELILIDWEGLKLAPVEADLMFFA 239

CHCC14561_2621 MMESALSLADDLKCSGRRLALCHTDVHGWN-LMKTGGELILIDWEGLKLAPVEADLMFFA 239

CHCC15292_0696 MMESALSLADDLKCSGRRLALCHTDVHGWN-LMKTGGELILIDWEGLKLAPVEADLMFFA 239

CHCC14818_3873 MMESALSLADDLKCSGRRLALCHTDVHGWN-LMKTGGELILIDWEGLKLAPVEADLMFFA 239

CHCC14816_0250 MMESALSLADDLKCSGRRLALCHTDVHGWN-LMKTGGELILIDWEGLKLAPVEADLMFFA 239

CHCC5025_0547 MMESALSLADDLKCSGRRLALCHTDVHGWN-LMKTGGELILIDWEGLKLAPVEADLMFFA 239

CHCC20327_1736 MMESALSLADDLKCSGRRLALCHTDVHGWN-LMKTGGELILIDWEGLKLAPVEADLMFFA 239

CHCC14814_3093 MMESALSLADDLKCSGRRLALCHTDVHGWNLMMKTGGELILIDWEGLKLAPVEADLMFFA 240

CHCC15381_2837 MMEAALSLADDLKCSDRRLALCHTDIHGWN-MMKTGGELILIDWEGLKLAPVEADLIFFA 239

CHCC19467_4581 MMEAALSLADDLKCSDRRLALCHTDIHGWN-MMKTGGELILIDWEGLKLAPVEADLIFFA 239

CHCC19468_2702 MMEAALSLADDLKCSDRRLALCHTDIHGWN-MMKTGGELILIDWEGLKLAPVEADLIFFA 239

CHCC20497_4636 MMEAALSLADDLKCSDRRLALCHTDIHGWN-MMKTGGELILIDWEGLKLAPVEADLIFFA 239

CHCC20492_2744 MMEAALSLADDLKCSDRRLALCHTDIHGWN-MMKTGGELILIDWEGLKLAPVEADLIFFA 239

CHCC14523_3915 MMEAALSLADDLKCSDRRLALCHTDIHGWN-MMKTGGELILIDWEGLKLAPVEADLIFFA 239

CHCC14527_2373 MMEAALSLADDLKCSDRRLALCHTDIHGWN-MMKTGGELILIDWEGLKLAPVEADLIFFA 239

ATCC9945A_0221 MMEAALSLADDLKCSDRRLALCHTDIHGWN-MMKTGGELILIDWEGLKLAPVEADLIFFA 239

CHCC5019_4455 MMEAALSLADDLKCSDRRLALCHTDIHGWN-MMKTGGELILIDWEGLKLAPVEADLIFFA 239

CHCC15337_4611 MMEAALSLADDLKCSDRRLALCHTDIHGWN-MMKTGGELILIDWEGLKLAPVEADLIFFA 239

CHCC15332_3736 MMEAALSLADDLKCSDRRLALCHTDIHGWN-MMKTGGELILIDWEGLKLAPVEADLIFFA 239

CHCC5023_1980 MMEAALSLADDLKCSDRRLALCHTDIHGWN-MMKTGGELILIDWEGLKLAPVEADLIFFA 239

CHCC5021_2116 MMEAALSLADDLKCSDRRLALCHTDIHGWN-MMKTGGELILIDWEGLKLAPVEADLIFFA 239

CHCC20488_0915 MMEAALSLADDLKCSDRRLALCHTDIHGWN-MMKTGGELILIDWEGLKLAPVEADLIFFA 239

CHCC20331_1438 MMEAALSLADDLKCSDRRLALCHTDIHGWN-MMKTGGELILIDWEGLKLAPVEADLIFFA 239

CHCC20490_1917 MMEAALSLADDLKCSDRRLALCHTDIHGWN-MMKTGGELILIDWEGLKLAPVEADLMFFA 239

CHCC20347_3230 MMEAALSLADDLKCSDRRLALCHTDIHGWN-MMKTGGELILIDWEGLKLAPVEADLMFFA 239

CHCC14820_1470 MMEAALSLADDLKCSDRRLALCHTDIHGWN-MMKTGGELILIDWEGLKLAPVEADLMFFA 239

CHCC20372_2719 MMEAALSLADDLKCSDRRLALCHTDIHGWN-MMKTGGELILIDWEGLKLAPVEADLMFFA 239

CHCC20348_4390 MMEAALSLADDLKCSDRRLALCHTDIHGWN-MMKTGGELILIDWEGLKLAPVEADLMFFA 239

CHCC5027_3367 MMEAALSLADDLKCSDRRLALCHTDIHGWN-MMKTGGELILIDWEGLKLAPVEADLMFFA 239

CHCC12620_0738 MMEAALSLADDLKCSDRRLALCHTDIHGWN-MMKTGGELILIDWEGLKLAPVEADLMFFA 239

CHCC20491_0560 MMEAALSLADDLKCSDRRLALCHTDIHGWN-MMKTGGELILIDWEGLKLAPVEADLMFFA 239

CHCC15136_3272 MMEAALSLADDLKCSDRRLALCHTDIHGWN-MMKTGGELILIDWEGLKLAPVEADLMFFA 239

CHCC5022_3898 MMEAALSLADDLKCSDRRLALCHTDIHGWN-MMKTGGELILIDWEGLKLAPVEADLMFFA 239

CHCC14817_1576 MMEAALSLADDLKCSDRRLALCHTDIHGWN-MMKTGGELILIDWEGLKLAPVEADLMFFA 239

CHCC4186_2036 MMEAALSLADDLKCSDRRLALCHTDIHGWN-MMKTGGELILIDWEGLKLAPVEADLMFFA 239

BL09_0220 MMEAALSLADDLKCSDRRLALCHTDIHGWN-MMKTGGELILIDWEGLKLAPVEADLMFFA 239

CHCC20333_3257 MMEAALSLADDLKCSDRRLALCHTDIHGWN-MMKTGGELILIDWEGLKLAPVEADLMFFA 239

CHCC20375_3121 MMEAALSLADDLKCSGRRMALCHTDVHGWN-LMKTGGELILIDWEGLKLAPVEADLMFFA 239

***:***********.**:******:**** :************************:***

CHCC10893_2819 NQPYAQEFLRVYCETHKGFEIDQNALRFYQIRRRLEDIWEFTEQLAFDIQAEKEKAETMS 299

CHCC19466_3946 NQPYAQEFLRVYCETHKGFEIDQNALRFYQIRRRLEDIWEFTEQLAFDIQAEKEKAETMS 299

CHCC20494_1490 NQPYAQEFLRVYCETHKGFEIDQNALRFYQIRRRLEDIWEFTEQLAFDIQAEKEKAETMS 299

CHCC20493_0356 NQPYAQEFLRVYCETHKGFEIDQNALRFYQIRRRLEDIWEFTEQLAFDIQAEKEKAETMS 299

CHCC14437_0389 NQPYAQEFLRVYCETHKGFEIDQNALRFYQIRRRLEDIWEFTEQLAFDIQAEKEKAETMS 299

CHCC20323_0097 NQPYAQEFLRVYCETHKGFEIDQNALRFYQIRRRLEDIWEFTEQLAFDIQAEKEKAETMS 299

CHCC15318_3692 NQPYAQEFLRVYCETHKGFEIDQNALRFYQIRRRLEDIWEFTEQLAFDIQAEKEKAETMS 299

CHCC20486_2039 NQPYAQEFLRVYCETHKGFEIDQNALRFYQIRRRLEDIWEFTEQLAFDIQAEKEKAETMS 299

CHCC14441_1577 NQPYAQEFLRVYCETHKGFEIDQNALRFYQIRRRLEDIWEFTEQLAFDIQAEKEKAETMS 299

CHCC14525_3597 NQPYAQEFLRVYCETHKGFEIDQNALRFYQIRRRLEDIWEFTEQLAFDIQAEKEKAETMS 299

CHCC15322_3644 NQPYAQEFLRVYCETHKGFEIDQNALRFYQIRRRLEDIWEFTEQLAFDIQAEKEKAETMS 299

CHCC15139_1360 NQPYAQEFLRVYCETHKGFEIDQNALRFYQIRRRLEDIWEFTEQLAFDIQAEKEKAETMS 299

CHCC20344_4474 NQPYAQEFLRVYCETHKGFEIDQNALRFYQIRRRLEDIWEFTEQLAFDIQAEKEKAETMS 299

CHCC20341_2337 NQPYAQEFLRVYCETHKGFEIDQNALRFYQIRRRLEDIWEFTEQLAFDIQAEKEKAETMS 299

CHCC20343_0034 NQPYAQEFLRVYCETHKGFEIDQNALRFYQIRRRLEDIWEFTEQLAFDIQAEKEKAETMS 299

CHCC20342_1143 NQPYAQEFLRVYCETHKGFEIDQNALRFYQIRRRLEDIWEFTEQLAFDIQAEKEKAETMS 299

CHCC14600_3226 NQPYAQEFLRVYCETHKGFEIDQNALRFYQIRRRLEDIWEFTEQLAFDIQAEKEKAETMS 299

CHCC5020_0927 NQPYAQEFLRVYCETHKGFEIDQNALRFYQIRRRLEDIWEFTEQLAFDIQAEKEKAETMS 299

CHCC5026_1707 NQPYAQEFLRVYCETHKGFEIDQNALRFYQIRRRLEDIWEFTEQLAFDIQAEKEKAETMS 299

CHCC14813_2855 NQPYAQEFLRVYCETHKGFEIDQNALRFYQIRRRLEDIWEFTEQLAFDIQAEKEKAETMS 299

CHCC14810_4313 NQPYAQEFLRVYCETHKGFEIDQNALRFYQIRRRLEDIWEFTEQLAFDIQAEKEKAETMS 299

CHCC14598_1059 NQPYAQEFLRVYCETHKGFEIDQNALRFYQIRRRLEDIWEFTEQLAFDIQAEKEKAETMS 299

CHCC15291_0393 NQPYAQEFLRVYCETHKGFEIDQNALRFYQIRRRLEDIWEFTEQLAFDIQAEKEKAETMS 299

CHCC15290_3000 NQPYAQEFLRVYCETHKGFEIDQNALRFYQIRRRLEDIWEFTEQLAFDIQAEKEKAETMS 299

CHCC14564_2766 NQPYAQEFLRVYCETHKGFEIDQNALRFYQIRRRLEDIWEFTEQLAFDIQAEKEKAETMS 299

CHCC20373_0249 NQPYAQEFLRVYCETHKGFEIDQNALRFYQIRRRLEDIWEFTEQLAFDIQAEKEKAETMS 299

CHCC15289_3954 NQPYAQEFLRVYCETHKGFEIDQNALRFYQIRRRLEDIWEFTEQLAFDIQAEKEKAETMS 299

CHCC20325_3728 NQPYAQEFLRVYCETHKGFEIDQNALRFYQIRRRLEDIWEFTEQLAFDIQA-----ETMS 294

CHCC20440_1829 NQPYAQEFLRVYCETHKGFEIDQNALRFYQIRRRLEDIWEFTEQLAFDIQA-----ETMS 294

CHCC20441_1081 NQPYAQEFLRVYCETHKGFEIDQNALRFYQIRRRLEDIWEFTEQLAFDIQA-----ETMS 294

CHCC15546_0034 NQPYAQEFLRVYCETHKGFEIDQNALRFYQIRRRLEDIWEFTEQLAFDIQAEKEKAETMS 299

CHCC15543_3601 NQPYAQEFLRVYCETHKGFEIDQNALRFYQIRRRLEDIWEFTEQLAFDIQAEKEKAETMS 299

CHCC16874_4693 NQPYAQEFLRVYCETHKGFEIDQNALRFYQIRRRLEDIWEFTEQLAFDIQAEKEKAETMS 299

CHCC16736_1643 NQPYAQEFLRVYCETHKGFEIDQNALRFYQIRRRLEDIWEFTEQLAFDIQAEKEKAETMS 299

CHCC20495_0877 NQPYAQEFLRVYCETHKGFEIDQNALRFYQIRRRLEDIWEFTEQLAFDIQAEKEKAETMS 299

CHCC14557_3891 NQPYAQEFLRVYCETHKGFEIDQNALRFYQIRRRLEDIWEFTEQLAFDIQAEKEKAETMS 299

CHCC15315_4194 NQPYAQEFLRVYCETHKGFEIDQNALRFYQIRRRLEDIWEFTEQLAFDIQAEKEKAETMS 299

CHCC15311_0625 NQPYAQEFLRVYCETHKGFEIDQNALRFYQIRRRLEDIWEFTEQLAFDIQAEKEKAETMS 299

CHCC20345_4265 NQPYAQEFLRVYCETHKGFEIDQNALRFYQIRRRLEDIWEFTEQLAFDIQAEKEKAETMS 299

CHCC15335_1763 NQPYAQEFLRVYCETHKGFEIDQNALRFYQIRRRLEDIWEFTEQLAFDIQAEKEKAETMS 299

CHCC14808_3670 NQPYAQEFLRVYCETHKGFEIDQNALRFYQIRRRLEDIWEFTEQLAFDIQAEKEKAETMS 299

CHCC5024_4270 NQPYAQEFLRVYCETHKGFEIDQNALRFYQIRRRLEDIWEFTEQLAFDIQAEKEKAETMS 299

CHCC14819_0398 NQPYAQEFLRVYCETHKGFEIDQNALRFYQIRRRLEDIWEFTEQLAFDIQAEKEKAETMS 299

CHCC14429_3014 NQPYAQEFLRVYCETHKGFEIDQNALRFYQIRRRLEDIWEFTEQLAFDIQTEKEKAETMS 299

CHCC15091_2902 NQPYAQEFLRVYCETHKGFEIDQNALRFYQIRRRLEDIWEFTEQLAFDIQTEKEKAETMS 299

CHCC20496_3909 NQPYAQEFLRVYCETHKGFEIDQNALRFYQIRRRLEDIWEFTEQLAFDIQTEKEKAETMS 299

CHCC14559_1464 NQPYAQEFLRVYCETHKGFEIDQNALRFYQIRRRLEDIWEFTEQLAFDIQTEKEKAETMS 299

CHCC14566_2258 NQPYAQEFLRVYCETHKGFEIDQNALRFYQIRRRLEDIWEFTEQLAFDIQTEKEKAETMS 299

CHCC14435_2913 NQPYAQEFLRVYCETHKGFEIDQNALRFYQIRRRLEDIWEFTEQLAFDIQTEKEKAETMS 299

CHCC20489_0346 NQPYAQEFLRVYCETHKGFEIDQNALRFYQIRRRLEDIWEFTEQLAFDIQTEKEKAETMS 299

CHCC20487_3825 NQPYAQEFLRVYCETHKGFEIDQNALRFYQIRRRLEDIWEFTEQLAFDIQTEKEKAETMS 299

CHCC14568_0476 NQPYAQEFLRVYCETHKGFEIDQNALRFYQIRRRLEDIWEFTEQLAFDIQTEKEKAETMS 299

CHCC20339_0022 NQPYAQEFLRVYCETHKGFEIDQNALRFYQIRRRLEDIWEFTEQLAFDIQTEKEKAETMS 299

CHCC15325_0414 NQPYAQEFLRVYCETHKGFEIDQNALRFYQIRRRLEDIWEFTEQLAFDIQTEKEKAETMS 299

CHCC14688_3357 NQPYAQEFLRVYCETHKGFEIDQNALRFYQIRRRLEDIWEFTEQLAFDIQTEKEKAETMS 299

CHCC20442_1307 NQPYAQEFLRVYCETHKGFEIDQNALRFYQIRRRLEDIWEFTEQLAFDIQTEKEKAETMS 299

CHCC14815_0972 NQPYAQEFLRVYCETHKGFEIDQNALRFYQIRRRLEDIWEFTEQLAFDIQTEKEKAETMS 299

CHCC20368_2018 NQPYAQEFLRVYCETHKGFEIDQNALRFYQIRRRLEDIWEFTEQLAFDIQTEKEKAETMS 299

CHCC14562_0763 NQPYAQEFLRVYCETHKGFEIDQNALRFYQIRRRLEDIWEFTEQLAFDIQTEKEKAETMS 299

CHCC15320_2308 NQPYAQEFLRVYCETHKGFKIDQNALRFYQIRRRLEDIWEFTEQLAFDIQTEKEKAETMS 299

CHCC20369_2216 NQPYAQEFLRVYCETHKGFEIDQNALRFYQIRRRLEDIWEFTEQLAFDIQTEKEKAETMS 299

CHCC14431_3646 NQPYAQEFLRVYCETHKGFEIDQNALRFYQIRRRLEDIWEFTEQLAFDIQTEKEKAETMS 299

CHCC15087_0206 NQPYAQEFLRVYCETHKGFEIDQNALRFYQIRRRLEDIWEFTEQLAFDIQTEKEKAETMS 299

CHCC14809_3059 NQPYAQEFLRVYCETHKGFEIDQNALRFYQIRRRLEDIWEFTEQLAFDIQTEKEKAETMS 299

CHCC14596_0535 NQPYAQEFLRVYCETHKGFEIDQNALRFYQIRRRLEDIWEFTEQLAFDIQTEKEKAETMS 299

CHCC15075_1355 NQPYAQEFLRVYCETHKGFEIDQNALRFYQIRRRLEDIWEFTEQLAFDIQTEKEKAETMS 299

DSM13_0193 NQPYAQEFLRVYCETHKGFEIDQNALRFYQIRRRLEDIWEFTEQLAFDIQTEKEKAETMS 299

CHCC14561_2621 NQPYAQEFLRVYCETHKGFEIDQNALRFYQIRRRLEDIWEFTEQLAFDIQTEKEKAETMS 299

CHCC15292_0696 NQPYAQEFLRVYCETHKGFEIDQNALRFYQIRRRLEDIWEFTEQLAFDIQTEKEKAETMS 299

CHCC14818_3873 NQPYAQEFLRVYCETHKGFEIDQNALRFYQIRRRLEDIWEFTEQLAFDIQTEKEKAETMS 299

CHCC14816_0250 NQPYAQEFLRVYCETHKGFEIDQNALRFYQIRRRLEDIWEFTEQLAFDIQTEKEKAETMS 299

CHCC5025_0547 NQPYAQEFLRVYCETHKGFEIDQNALRFYQIRRRLEDIWEFTEQLAFDIQTEKEKAETMS 299

CHCC20327_1736 NQPYAQEFLRVYCETHKGFEIDQNALRFYQIRRRLEDIWEFTEQLAFDIQTEKEKAETMS 299

CHCC14814_3093 NQPYVQEFLRVYREIHKGFEIDQNALRFYQIRRRLEDIWEFTEQLAFDIQTEKEKAETMS 300

CHCC15381_2837 NQPYVQEFLRVYRETHKGFEIDQNALRFYQIRRRLEDIWEFTEQLAFDIQTENEKAETMS 299

CHCC19467_4581 NQPYVQEFLRVYRETHKGFEIDQNALRFYQIRRRLEDIWEFTEQLAFDIQTENEKAETMS 299

CHCC19468_2702 NQPYVQEFLRVYRETHKGFEIDQNALRFYQIRRRLEDIWEFTEQLAFDIQTENEKAETMS 299

CHCC20497_4636 NQPYVQEFLRVYRETHKGFEIDQNALRFYQIRRRLEDIWEFTEQLAFDIQTENEKAETMS 299

CHCC20492_2744 NQPYVQEFLRVYRETHKGFEIDQNALRFYQIRRRLEDIWEFTEQLAFDIQTENEKAETMS 299

CHCC14523_3915 NQPYVQEFLRVYRETHKGFEIDQNALRFYQIRRRLEDIWEFTEQLAFDIQTENEKAETMS 299

CHCC14527_2373 NQPYVQEFLRVYRETHKGFEIDQNALRFYQIRRRLEDIWEFTEQLAFDIQTENEKAETMS 299

ATCC9945A_0221 NQPYVQEFLRVYRETHKGFEIDQNALRFYQIRRRLEDIWEFTEQLAFDIQTENEKAETMS 299

CHCC5019_4455 NQPYVQEFLRVYRETHKGFEIDQNALRFYQIRRRLEDIWEFTEQLAFDIQTENEKAETMS 299

CHCC15337_4611 NQPYVQEFLRVYRETHKGFEIDQNALRFYQIRRRLEDIWEFTEQLAFDIQTENEKAETMS 299

CHCC15332_3736 NQPYVQEFLRVYRETHKGFEIDQNALRFYQIRRRLEDIWEFTEQLAFDIQTENEKAETMS 299

CHCC5023_1980 NQPYVQEFLRVYRETHKGFEIDQNALRFYQIRRRLEDIWEFTEQLAFDIQTENEKAETMS 299

CHCC5021_2116 NQPYVQEFLRVYRETHKGFEIDQNALRFYQIRRRLEDIWEFTEQLAFDIQTENEKAETMS 299

CHCC20488_0915 NQPYVQEFLRVYRETHKGFEIDQNALRFYQIRRRLEDIWEFTEQLAFDIQTENEKAETMS 299

CHCC20331_1438 NQPYVQEFLRVYRETHKGFEIDQNALRFYQIRRRLEDIWEFTEQLAFDIQTENEKAETMS 299

CHCC20490_1917 NQPYVQEFLRVYPETHKGFEIDQNALRFYQIRRRLEDIWEFTEQLAFDIQTENEKAETMS 299

CHCC20347_3230 NQPYVQEFLRVYPETHKGFEIDQNALRFYQIRRRLEDIWEFTEQLAFDIQTENEKAETMS 299

CHCC14820_1470 NQPYVQEFLRVYRETHKGFEIDQNALRFYQIRRRLEDIWEFTEQLAFDIQTENEKAETMS 299

CHCC20372_2719 NQPYVQEFLRVYRETHKGFEIDQNALRFYQIRRRLEDIWEFTEQLAFDIQTENEKAETMS 299

CHCC20348_4390 NQPYVQEFLRVYRETHKGFEIDQNALRFYQIRRRLEDIWEFTEQLAFDIQTENEKAETMS 299

CHCC5027_3367 NQPYVQEFLRVYRETHKGFEIDQNALRFYQIRRRLEDIWEFTEQLAFDIQTENEKAETMS 299

CHCC12620_0738 NQPYVQEFLRVYRETHKGFEIDQNALRFYQIRRRLEDIWEFTEQLAFDIQTEKEKAETLS 299

CHCC20491_0560 NQPYVQEFLRVYRETHKGFEIDQNALRFYQIRRRLEDIWEFTEQLAFDIQTEKEKAETLS 299

CHCC15136_3272 NQPYVQEFLRVYRETHKGFEIDQNALRFYQIRRRLEDIWEFTEQLAFDIQTEKEKAETLS 299

CHCC5022_3898 NQPYVQEFLRVYRETHKGFEIDQNALRFYQIRRRLEDIWEFTEQLAFDIQTEKEKAETLS 299

CHCC14817_1576 NQPYVQEFLRVYRETHKGFEIDQNALRFYQIRRRLEDIWEFTEQLAFDIQTEKEKAETLS 299

CHCC4186_2036 NQPYVQEFLRVYRETHKGFEIDQNALRFYQIRRRLEDIWEFTEQLAFDIQTEKEKAETLS 299

BL09_0220 NQPYVQEFLRVYRETHKGFEIDQNALRFYQIRRRLEDIWEFTEQLAFDIQTEKEKAETLS 299

CHCC20333_3257 NQPYVQEFLRVYRETHKGFEIDQNALRFYQIRRRLEDIWEFTEQLAFDIQTEKEKAETLS 299

CHCC20375_3121 NQPYAQEFLRVYCETHKGFEIDQNALRFYQIRRRLEDIWEFTEQLAFDIQTETEKAETIS 299

****.******* * ****:******************************: **:*

CHCC10893_2819 LLKNELEAIKDSKTGY 315

CHCC19466_3946 LLKNELEAIKDSKTGY 315

CHCC20494_1490 LLKNELEAIKDSKTGY 315

CHCC20493_0356 LLKNELEAIKDSKTGY 315

CHCC14437_0389 LLKNELEAIKDSKTGY 315

CHCC20323_0097 LLKNELEAIKDSKTGY 315

CHCC15318_3692 LLKNELEAIKDSKTGY 315

CHCC20486_2039 LLKNELEAIKDSKTGY 315

CHCC14441_1577 LLKNELEAIKDSKTGY 315

CHCC14525_3597 LLKNELEAIKDSKTGY 315

CHCC15322_3644 LLKNELEAIKDSKTGY 315

CHCC15139_1360 LLKNELEAIKDSKTGY 315

CHCC20344_4474 LLKNELEAIKDSKTGY 315

CHCC20341_2337 LLKNELEAIKDSKTGY 315

CHCC20343_0034 LLKNELEAIKDSKTGY 315

CHCC20342_1143 LLKNELEAIKDSKTGY 315

CHCC14600_3226 LLKNELEAIKDSKTGY 315

CHCC5020_0927 LLKNELEAIKDSKTGY 315

CHCC5026_1707 LLKNELEAIKDSKTGY 315

CHCC14813_2855 LLKNELEAIKDSKTGY 315

CHCC14810_4313 LLKNELEAIKDSKTGY 315

CHCC14598_1059 LLKNELEAIKDSKTGY 315

CHCC15291_0393 LLKNELEAIKDSKTGY 315

CHCC15290_3000 LLKNELEAIKDSKTGY 315

CHCC14564_2766 LLKNELEAIKDSKTGY 315

CHCC20373_0249 LLKNELEAIKDSKTGY 315

CHCC15289_3954 LLKNELEAIKDSKTGY 315

CHCC20325_3728 LLKNELEAIKDSKTGY 310

CHCC20440_1829 LLKNELEAIKDSKTGY 310

CHCC20441_1081 LLKNELEAIKDSKTGY 310

CHCC15546_0034 LLKNELEAIKDSKTGY 315

CHCC15543_3601 LLKNELEAIKDSKTGY 315

CHCC16874_4693 LLKNELEAIKDSKTGY 315

CHCC16736_1643 LLKNELEAIKDSKTGY 315

CHCC20495_0877 LLKNELEAIKDSKTGY 315

CHCC14557_3891 LLKNELEAIKDSKTGY 315

CHCC15315_4194 LLKNELEAIKDSKTGY 315

CHCC15311_0625 LLKNELEAIKDSKTGY 315

CHCC20345_4265 LLKNELEAIKDSKTGY 315

CHCC15335_1763 LLKNELEAIKDSKTGY 315

CHCC14808_3670 LLKNELEAIKDSKTGY 315

CHCC5024_4270 LLKNELEAIKDSKTGY 315

CHCC14819_0398 LLKNELEAIKDSKTGY 315

CHCC14429_3014 LLKNELEAIKDSKTGY 315

CHCC15091_2902 LLKNELEAIKDSKTGY 315

CHCC20496_3909 LLKNELEAIKDSKTGY 315

CHCC14559_1464 LLKNELEAIKDSKTGY 315

CHCC14566_2258 LLKNELEAIKDSKTGY 315

CHCC14435_2913 LLKNELEAIKDSKTGY 315

CHCC20489_0346 LLKNELEAIKDSKTGY 315

CHCC20487_3825 LLKNELEAIKDSKTGY 315

CHCC14568_0476 LLKNELEAIKDSKTGY 315

CHCC20339_0022 LLKNELEAIKDSKTGY 315

CHCC15325_0414 LLKNELEAIKDSKTGY 315

CHCC14688_3357 LLKNELEAIKDSKTGY 315

CHCC20442_1307 LLKNELEAIKDSKTGY 315

CHCC14815_0972 LLKNELEAIKDSKTGY 315

CHCC20368_2018 LLKNELEAIKDSKTGY 315

CHCC14562_0763 LLKNELEAIKDSKTGY 315

CHCC15320_2308 LLKNELEAIKDSKTGY 315

CHCC20369_2216 FLKNELEAIKDSKTGY 315

CHCC14431_3646 LLKNELEAIKDSKTGY 315

CHCC15087_0206 LLKNELEAIKDSKTGY 315

CHCC14809_3059 LLKNELEAIKDSKTGY 315

CHCC14596_0535 LLKNELEAIKDSKTGY 315

CHCC15075_1355 LLKNELEAIKDSKTGY 315

DSM13_0193 LLKNELEAIKDSKTGY 315

CHCC14561_2621 LLKNELEAIKDSKTGY 315

CHCC15292_0696 LLKNELEAIKDSKTGY 315

CHCC14818_3873 LLKNELEAIKDSKTGY 315

CHCC14816_0250 LLKNELEAIKDSKTGY 315

CHCC5025_0547 LLKNELEAIKDSKTGY 315

CHCC20327_1736 LLKNELEAIKDSKTGY 315

CHCC14814_3093 LLKNELEAIEDSKINY 316

CHCC15381_2837 LLKNELEAIEDSKIDC 315

CHCC19467_4581 LLKNELEAIEDSKIDC 315

CHCC19468_2702 LLKNELEAIEDSKIDC 315

CHCC20497_4636 LLKNELEAIEDSKIDC 315

CHCC20492_2744 LLKNELEAIEDSKIDC 315

CHCC14523_3915 LLKNELEAIEDSKIDC 315

CHCC14527_2373 LLKNELEAIEDSKIDC 315

ATCC9945A_0221 LLKNELEAIEDSKIDC 315

CHCC5019_4455 LLKNELEAIEDSKIDC 315

CHCC15337_4611 LLKNELEAIEDSKIDC 315

CHCC15332_3736 LLKNELEAIEDSKIDC 315

CHCC5023_1980 LLKNELEAIEDSKIDC 315

CHCC5021_2116 LLKNELEAIEDSKIDC 315

CHCC20488_0915 LLKNELEAIEDSKIDC 315

CHCC20331_1438 LLKNELEAIEDSKIDC 315

CHCC20490_1917 LLKNELEAIEDSKIDC 315

CHCC20347_3230 LLKNELEAIEDSKIDC 315

CHCC14820_1470 LLKNELEAIEDSKIDC 315

CHCC20372_2719 LLKNELEAIEDSKIDC 315

CHCC20348_4390 LLKNELEAIEDSKIDC 315

CHCC5027_3367 LLKNELEAIEDSKIDC 315

CHCC12620_0738 LLKNELEAIEDSKIDC 315

CHCC20491_0560 LLKNELEAIEDSKIDC 315

CHCC15136_3272 LLKNELEAIEDSKIDC 315

CHCC5022_3898 LLKNELEAIEDSKIDC 315

CHCC14817_1576 LLKNELEAIEDSKIDC 315

CHCC4186_2036 LLKNELEAIEDSKIDC 315

BL09_0220 LLKNELEAIEDSKIDC 315

CHCC20333_3257 LLKNELEAIEDSKIDC 315

CHCC20375_3121 LLKNELEAIEDSKIDY 315

:********.*** .
