# Supplementary material for: Putative antibiotic resistance genes present in extant Bacillus licheniformis and Bacillus paralicheniformis strains are probably intrinsic and part of the ancient resistome
Source: PLoS One. 2019 Jan 15;14(1):e0210363. doi: 10.1371/journal.pone.0210363 (PMC6333372; doi:10.1371/journal.pone.0210363)
Supplement: S9 Fig — Sequence alignments were made with Clustal Omega using default settings. The left column indicates the locus tag of each ermD gene per strain. Identical amino acids are indicated by an asterisk below each column. Residue numbers are indicated at the end of each row. (DOCX) [file pone.0210363.s009.docx]

CHCC20488_2428 MKKKNHKYRGKKLNRGESPNFSGQHLMHNKKLIEEIVDRANISIDDTVLELGAGKGALTT 60

BL09_3044 MKKKNHKYRGKKLNRGESPNFSGQHLMHNKKLIEEIVDRANISIDDTVLELGAGKGALTT 60

CHCC14817_2224 MKKKNHKYRGKKLNRGESPNFSGQHLMHNKKLIEEIVDRANISIDDTVLELGAGKGALTT 60

CHCC5021_0575 MKKKNHKYRGKKLNRGESPNFSGQHLMHNKKLIEEIVDRANISIDDTVLELGAGKGALTT 60

CHCC15332_2404 MKKKNHKYRGKKLNRGESPNFSGQHLMHNKKLIEEIVDRANISIDDTVLELGAGKGALTT 60

CHCC15337_3786 MKKKNHKYRGKKLNRGESPNFSGQHLMHNKKLIEEIVDRANISIDDTVLELGAGKGALTT 60

CHCC20347_3669 MKKKNHKYRGKKLNRGESPNFSGQHLMHNKKLIEEIVDRANISIDDTVLELGAGKGALTT 60

CHCC15136_3010 MKKKNHKYRGKKLNRGESPNFSGQHLMHNKKLIEEIVDRANISIDDTVLELGAGKGALTT 60

ATCC9945A_2991 MKKKNHKYRGKKLNRGESPNFSGQHLMHNKKLIEEIVDRANISIDDTVLELGAGKGALTT 60

CHCC14527_3619 MKKKNHKYRGKKLNRGESPNFSGQHLMHNKKLIEEIVDRANISIDDTVLELGAGKGALTT 60

CHCC14523_2532 MKKKNHKYRGKKLNRGESPNFSGQHLMHNKKLIEEIVDRANISIDDTVLELGAGKGALTT 60

CHCC20492_1445 MKKKNHKYRGKKLNRGESPNFSGQHLMHNKKLIEEIVDRANISIDDTVLELGAGKGALTT 60

CHCC20491_1195 MKKKNHKYRGKKLNRGESPNFSGQHLMHNKKLIEEIVDRANISIDDTVLELGAGKGALTT 60

CHCC20497_3638 MKKKNHKYRGKKLNRGESPNFSGQHLMHNKKLIEEIVDRANISIDDTVLELGAGKGALTT 60

CHCC19468_0855 MKKKNHKYRGKKLNRGESPNFSGQHLMHNKKLIEEIVDRANISIDDTVLELGAGKGALTT 60

CHCC19467_2168 MKKKNHKYRGKKLNRGESPNFSGQHLMHNKKLIEEIVDRANISIDDTVLELGAGKGALTT 60

CHCC12620_3847 MKKKNHKYRGKKLNRGESPNFSGQHLMHNKKLIEEIVDRANISIDDTVLELGAGKGALTT 60

CHCC15381_0096 MKKKNHKYRGKKLNRGESPNFSGQHLMHNKKLIEEIVDRANISIDDTVLELGAGKGALTT 60

CHCC14814_2880 MKKKNHKYRGKKLNRGESPNFSGQHLMHNKKLIEEIVDRANIGIDDTVLELGAGKGALTT 60

CHCC16874_3885 MKKKNHKYRGKKLNRGEYPNFSGQHLMHNKKLIEEIVDRANISIDDTVLELGAGKGALTT 60

CHCC20494_3822 MKKKNHKYRGKKLNRGEYPNFSGQHLMHNKKLIEEIVDRANISIDDTVLELGAGKGALTT 60

CHCC20496_2720 MKKKNHKYRGKKLNRGEYPNFSGQHLMHNKKLIEEIVDRANISIDDTVLELGAGKGALTT 60

CHCC20493_4062 MKKKNHKYRGKKLNRGEYPNFSGQHLMHNKKLIEEIVDRANISIDDTVLELGAGKGALTT 60

CHCC15325_3700 MKKKNHKYRGKKLNRGEYPNFSGQHLMHNKKLIEEIVDRANISIDDTVLELGAGKGALTT 60

CHCC20343_0710 MKKKNHKYRGKKLNRGEYPNFSGQHLMHNKKLIEEIVDRANISIDDTVLELGAGKGALTT 60

CHCC5025_4590 MKKKNHKYRGKKLNRGEYPNFSGQHLMHNKKLIEEIVDRANISIDDTVLELGAGKGALTT 60

CHCC20331_0924 MKKKNHKYRGKKLNRGEYPNFSGQHLMHNKKLIEEIVDRANISIDDTVLELGAGKGALTT 60

CHCC20372_3771 MKKKNHKYRGKKLNRGEYPNFSGQHLMHNKKLIEEIVDRANISIDDTVLELGAGKGALTT 60

CHCC20348_2758 MKKKNHKYRGKKLNRGEYPNFSGQHLMHNKKLIEEIVDRANISIDDTVLELGAGKGALTT 60

CHCC20333_1384 MKKKNHKYRGKKLNRGEYPNFSGQHLMHNKKLIEEIVDRANISIDDTVLELGAGKGALTT 60

CHCC20490_4191 MKKKNHKYRGKKLNRGEYPNFSGQHLMHNKKLIEEIVDRANISIDDTVLELGAGKGALTT 60

CHCC5019_3869 MKKKNHKYRGKKLNRGEYPNFSGQHLMHNKKLIEEIVDRANISIDDTVLELGAGKGALTT 60

CHCC5022_3686 MKKKNHKYRGKKLNRGEYPNFSGQHLMHNKKLIEEIVDRANISIDDTVLELGAGKGALTT 60

CHCC5023_4361 MKKKNHKYRGKKLNRGEYPNFSGQHLMHNKKLIEEIVDRANISIDDTVLELGAGKGALTT 60

CHCC5027_4245 MKKKNHKYRGKKLNRGEYPNFSGQHLMHNKKLIEEIVDRANISIDDTVLELGAGKGALTT 60

CHCC4186_0312 MKKKNHKYRGKKLNRGEYPNFSGQHLMHNKKLIEEIVDRANISIDDTVLELGAGKGALTT 60

CHCC14820_3578 MKKKNHKYRGKKLNRGEYPNFSGQHLMHNKKLIEEIVDRANISIDDTVLELGAGKGALTT 60

CHCC20375_2305 MKKKNHKYRGKKLNRRESPNFSGQHLMHNKKLIEEIVDWANIGIDDTVLELGAGKGALTT 60

CHCC10893_4267 MKKKNLKYRGKKLNRGESPNFSGQHLMHNKKLIEEIVDRANIGINDTVLELGAGKGALTT 60

CHCC14564_0457 MKKKNLKYRGKKLNRGESPNFSGQHLMHNKKLIEEIVDRANIGINDTVLELGAGKGALTT 60

CHCC19466_0697 MKKKNLKYRGKKLNRGESPNFSGQHLMHNKKLIEEIVDRANIGINDTVLELGAGKGALTT 60

CHCC20495_3329 MKKKNLKYRGKKLNRGESPNFSGQHLMHNKKLIEEIVDRANIGINDTVLELGAGKGALTT 60

CHCC20323_0831 MKKKNLKYRGKKLNRGESPNFSGQHLMHNKKLIEEIVDRANIGINDTVLELGAGKGALTT 60

CHCC14525_1264 MKKKNLKYRGKKLNRGESPNFSGQHLMHNKKLIEEIVDRANIGINDTVLELGAGKGALTT 60

CHCC15320_0006 MKKKNLKYRGKKLNRGESPNFSGQHLMHNKKLIEEIVDRANIGINDTVLELGAGKGALTT 60

CHCC14600_3499 MKKKNLKYRGKKLNRGESPNFSGQHLMHNKKLIEEIVDRANIGINDTVLELGAGKGALTT 60

CHCC14813_1065 MKKKNLKYRGKKLNRGESPNFSGQHLMHNKKLIEEIVDRANIGINDTVLELGAGKGALTT 60

CHCC14598_4524 MKKKNLKYRGKKLNRGESPNFSGQHLMHNKKLIEEIVDRANIGINDTVLELGAGKGALTT 60

CHCC15291_3091 MKKKNLKYRGKKLNRGESPNFSGQHLMHNKKLIEEIVDRANIGINDTVLELGAGKGALTT 60

CHCC20373_3131 MKKKNLKYRGKKLNRGESPNFSGQHLMHNKKLIEEIVDRANIGINDTVLELGAGKGALTT 60

CHCC15289_0637 MKKKNLKYRGKKLNRGESPNFSGQHLMHNKKLIEEIVDRANIGINDTVLELGAGKGALTT 60

****. ********* * ******************** ***.*:***************

CHCC20488_2428 VLSQKAGKVLAVENDSKFVDILTRKTAQHSNTKIIHQDIMKIHLPKEKFVVVSNIPYAIT 120

BL09_3044 VLSQKAGKVLAVENDSKFVDILTRKTAQHSNTKIIHQDIMKIHLPKEKFVVVSNIPYAIT 120

CHCC14817_2224 VLSQKAGKVLAVENDSKFVDILTRKTAQHSNTKIIHQDIMKIHLPKEKFVVVSNIPYAIT 120

CHCC5021_0575 VLSQKAGKVLAVENDSKFVDILTRKTAQHSNTKIIHQDIMKIHLPKEKFVVVSNIPYAIT 120

CHCC15332_2404 VLSQKAGKVLAVENDSKFVDILTRKTAQHSNTKIIHQDIMKIHLPKEKFVVVSNIPYAIT 120

CHCC15337_3786 VLSQKAGKVLAVENDSKFVDILTRKTAQHSNTKIIHQDIMKIHLPKEKFVVVSNIPYAIT 120

CHCC20347_3669 VLSQKAGKVLAVENDSKFVDILTRKTAQHSNTKIIHQDIMKIHLPKEKFVVVSNIPYAIT 120

CHCC15136_3010 VLSQKAGKVLAVENDSKFVDILTRKTAQHSNTKIIHQDIMKIHLPKEKFVVVSNIPYAIT 120

ATCC9945A_2991 VLSQKAGKVLAVENDSKFVDILTRKTAQHSNTKIIHQDIMKIHLPKEKFVVVSNIPYAIT 120

CHCC14527_3619 VLSQKAGKVLAVENDSKFVDILTRKTAQHSNTKIIHQDIMKIHLPKEKFVVVSNIPYAIT 120

CHCC14523_2532 VLSQKAGKVLAVENDSKFVDILTRKTAQHSNTKIIHQDIMKIHLPKEKFVVVSNIPYAIT 120

CHCC20492_1445 VLSQKAGKVLAVENDSKFVDILTRKTAQHSNTKIIHQDIMKIHLPKEKFVVVSNIPYAIT 120

CHCC20491_1195 VLSQKAGKVLAVENDSKFVDILTRKTAQHSNTKIIHQDIMKIHLPKEKFVVVSNIPYAIT 120

CHCC20497_3638 VLSQKAGKVLAVENDSKFVDILTRKTAQHSNTKIIHQDIMKIHLPKEKFVVVSNIPYAIT 120

CHCC19468_0855 VLSQKAGKVLAVENDSKFVDILTRKTAQHSNTKIIHQDIMKIHLPKEKFVVVSNIPYAIT 120

CHCC19467_2168 VLSQKAGKVLAVENDSKFVDILTRKTAQHSNTKIIHQDIMKIHLPKEKFVVVSNIPYAIT 120

CHCC12620_3847 VLSQKAGKVLAVENDSKFVDILTRKTAQHSNTKIIHQDIMKIHLPKEKFVVVSNIPYAIT 120

CHCC15381_0096 VLSQKAGKVLAVENDSKFVDILTRKTAQHSNTKIIHQDIMKIHLPKEKFVVVSNIPYAIT 120

CHCC14814_2880 VLCQKAGKVLAVENDSKFIDILTRKTAHHSNTKIIHQDILKIHLPKEKFVVVSNIPYAIT 120

CHCC16874_3885 MLSQKAGKVLAVENDSKFVAILTRKTAQHPNTKIIHQDIMKIHLPKEKFVVVSNIPYAIT 120

CHCC20494_3822 MLSQKAGKVLAVENDSKFVAILTRKTAQHPNTKIIHQDIMKIHLPKEKFVVVSNIPYAIT 120

CHCC20496_2720 MLSQKAGKVLAVENDSKFVAILTRKTAQHPNTKIIHQDIMKIHLPKEKFVVVSNIPYAIT 120

CHCC20493_4062 MLSQKAGKVLAVENDSKFVAILTRKTAQHPNTKIIHQDIMKIHLPKEKFVVVSNIPYAIT 120

CHCC15325_3700 MLSQKAGKVLAVENDSKFVAILTRKTAQHPNTKIIHQDIMKIHLPKEKFVVVSNIPYAIT 120

CHCC20343_0710 MLSQKAGKVLAVENDSKFVAILTRKTAQHPNTKIIHQDIMKIHLPKEKFVVVSNIPYAIT 120

CHCC5025_4590 MLSQKAGKVLAVENDSKFVAILTRKTAQHPNTKIIHQDIMKIHLPKEKFVVVSNIPYAIT 120

CHCC20331_0924 VLSQKAGKVLAVENDSKFVAILTRKTAQHPNTKIIHQDIMKIHLPKEKFVVVSNIPYAIT 120

CHCC20372_3771 VLSQKAGKVLAVENDSKFVAILTRKTAQHPNTKIIHQDIMKIHLPKEKFVVVSNIPYAIT 120

CHCC20348_2758 MLSQKAGKVLAVENDSKFVAILTRKTAQHPNTKIIHQDIMKIHLPKEKFVVVSNIPYAIT 120

CHCC20333_1384 VLSQKAGKVLAVENDSKFVAILTRKTAQHPNTKIIHQDIMKIHLPKEKFVVVSNIPYAIT 120

CHCC20490_4191 VLSQKAGKVLAVENDSKFVDILTRKTAQHSNTKIIHQDIMKIHLPKEKFVVVSNIPYAIT 120

CHCC5019_3869 VLSQKAGKVLAVENDSKFVDILTRKTAQHSNTKIIHQDIMKIHLPKEKFVVVSNIPYAIT 120

CHCC5022_3686 VLSQKAGKVLAVENDSKFVDILTRKTAQHSNTKIIHQDIMKIHLPKEKFVVVSNIPYAIT 120

CHCC5023_4361 VLSQKAGKVLAVENDSKFVDILTRKTAQHSNTKIIHQDIMKIHLPKEKFVVVSNIPYAIT 120

CHCC5027_4245 VLSQKAGKVLAVENDSKFVDILTRKTAQHSNTKIIHQDIMKIHLPKEKFVVVSNIPYAIT 120

CHCC4186_0312 VLSQKAGKVLAVENDSKFVDILTRKTAQHSNTKIIHQDIMKIHLPKEKFVVVSNIPYAIT 120

CHCC14820_3578 VLSQKAGKVLAVENDSKFVDILTRKTAQHSNTKIIHQDIMKIHLPKEKFVVVSNIPYAIT 120

CHCC20375_2305 VLSQKAGKVLAVENDSKFVDILTRKTAHHSNTKIIHQDIMKIHLPKEKFVVVSNIPYAIT 120

CHCC10893_4267 VLSQKAGKVLAVENDSKFVGILTRKTAQHSNAKIIHQDIMKIHLPKEKFVVVSNIPYAIT 120

CHCC14564_0457 VLSQKAGKVLAVENDSKFVGILTRKTAQHSNAKIIHQDIMKIHLPKEKFVVVSNIPYAIT 120

CHCC19466_0697 VLSQKAGKVLAVENDSKFVGILTRKTAQHSNAKIIHQDIMKIHLPKEKFVVVSNIPYAIT 120

CHCC20495_3329 VLSQKAGKVLAVENDSKFVGILTRKTAQHSNAKIIHQDIMKIHLPKEKFVVVSNIPYAIT 120

CHCC20323_0831 VLSQKAGKVLAVENDSKFVGILTRKTAQHSNAKIIHQDIMKIHLPKEKFVVVSNIPYAIT 120

CHCC14525_1264 VLSQKAGKVLAVENDSKFVGILTRKTAQHSNAKIIHQDIMKIHLPKEKFVVVSNIPYAIT 120

CHCC15320_0006 VLSQKAGKVLAVENDSKFVGILTRKTAQHSNAKIIHQDIMKIHLPKEKFVVVSNIPYAIT 120

CHCC14600_3499 VLSQKAGKVLAVENDSKFVGILTRKTAQHSNAKIIHQDIMKIHLPKEKFVVVSNIPYAIT 120

CHCC14813_1065 VLSQKAGKVLAVENDSKFVGILTRKTAQHSNAKIIHQDIMKIHLPKEKFVVVSNIPYAIT 120

CHCC14598_4524 VLSQKAGKVLAVENDSKFVGILTRKTAQHSNAKIIHQDIMKIHLPKEKFVVVSNIPYAIT 120

CHCC15291_3091 VLSQKAGKVLAVENDSKFVGILTRKTAQHSNAKIIHQDIMKIHLPKEKFVVVSNIPYAIT 120

CHCC20373_3131 VLSQKAGKVLAVENDSKFVGILTRKTAQHSNAKIIHQDIMKIHLPKEKFVVVSNIPYAIT 120

CHCC15289_0637 VLSQKAGKVLAVENDSKFVGILTRKTAQHSNAKIIHQDIMKIHLPKEKFVVVSNIPYAIT 120

:*.************ **: *******::.*:*******:********************

CHCC20488_2428 TPIMKMLLNNPASGFQKGIIVMEKGAAKRFTSKFIKNSYVLAWRMWFDIGIVREISKEHF 180

BL09_3044 TPIMKMLLNNPASGFQKGIIVMEKGAAKRFTSKFIKNSYVLAWRMWFDIGIVREISKEHF 180

CHCC14817_2224 TPIMKMLLNNPASGFQKGIIVMEKGAAKRFTSKFIKNSYVLAWRMWFDIGIVREISKEHF 180

CHCC5021_0575 TPIMKMLLNNPASGFQKGIIVMEKGAAKRFTSKFIKNSYVLAWRMWFDIGIVREISKEHF 180

CHCC15332_2404 TPIMKMLLNNPASGFQKGIIVMEKGAAKRFTSKFIKNSYVLAWRMWFDIGIVREISKEHF 180

CHCC15337_3786 TPIMKMLLNNPASGFQKGIIVMEKGAAKRFTSKFIKNSYVLAWRMWFDIGIVREISKEHF 180

CHCC20347_3669 TPIMKMLLNNPASGFQKGIIVMEKGAAKRFTSKFIKNSYVLAWRMWFDIGIVREISKEHF 180

CHCC15136_3010 TPIMKMLLNNPASGFQKGIIVMEKGAAKRFTSKFIKNSYVLAWRMWFDIGIVREISKEHF 180

ATCC9945A_2991 TPIMKMLLNNPASGFQKGIIVMEKGAAKRFTSKFIKNSYVLAWRMWFDIGIVREISKEHF 180

CHCC14527_3619 TPIMKMLLNNPASGFQKGIIVMEKGAAKRFTSKFIKNSYVLAWRMWFDIGIVREISKEHF 180

CHCC14523_2532 TPIMKMLLNNPASGFQKGIIVMEKGAAKRFTSKFIKNSYVLAWRMWFDIGIVREISKEHF 180

CHCC20492_1445 TPIMKMLLNNPASGFQKGIIVMEKGAAKRFTSKFIKNSYVLAWRMWFDIGIVREISKEHF 180

CHCC20491_1195 TPIMKMLLNNPASGFQKGIIVMEKGAAKRFTSKFIKNSYVLAWRMWFDIGIVREISKEHF 180

CHCC20497_3638 TPIMKMLLNNPASGFQKGIIVMEKGAAKRFTSKFIKNSYVLAWRMWFDIGIVREISKEHF 180

CHCC19468_0855 TPIMKMLLNNPASGFQKGIIVMEKGAAKRFTSKFIKNSYVLAWRMWFDIGIVREISKEHF 180

CHCC19467_2168 TPIMKMLLNNPASGFQKGIIVMEKGAAKRFTSKFIKNSYVLAWRMWFDIGIVREISKEHF 180

CHCC12620_3847 TPIMKMLLNNPASGFQKGIIVMEKGAAKRFTSKFIKNSYVLAWRMWFDIGIVREISKEHF 180

CHCC15381_0096 TPIMKMLLNNPASGFQKGIIVMEKGAAKRFTSKFIKNSYVLAWRMWFDIGIVREISKEHF 180

CHCC14814_2880 TPIMKMLLNNPASGFQKGIIVMEKGAAKRFTSKFIKNSYVLAWRMWFNIGIVREISKEHF 180

CHCC16874_3885 TPIMKMLLNNPASGFQKGIIVMEKGAAKRFTSKFIKNSYVLAWRMWFDIGIVREISKEHF 180

CHCC20494_3822 TPIMKMLLNNPASGFQKGIIVMEKGAAKRFTSKFIKNSYVLAWRMWFDIGIVREISKEHF 180

CHCC20496_2720 TPIMKMLLNNPASGFQKGIIVMEKGAAKRFTSKFIKNSYVLAWRMWFDIGIVREISKEHF 180

CHCC20493_4062 TPIMKMLLNNPASGFQKGIIVMEKGAAKRFTSKFIKNSYVLAWRMWFDIGIVREISKEHF 180

CHCC15325_3700 TPIMKMLLNNPASGFQKGIIVMEKGAAKRFTSKFIKNSYVLAWRMWFDIGIVREISKEHF 180

CHCC20343_0710 TPIMKMLLNNPASGFQKGIIVMEKGAAKRFTSKFIKNSYVLAWRMWFDIGIVREISKEHF 180

CHCC5025_4590 TPIMKMLLNNPASGFQKGIIVMEKGAAKRFTSKFIKNSYVLAWRMWFDIGIVREISKEHF 180

CHCC20331_0924 TPIMKMLLNNPASGFQKGIIVMEKGAAKRFTSKFIKNSYVLAWRMWFDIGIVREISKEHF 180

CHCC20372_3771 TPIMKMLLNNPASGFQKGIIVMEKGAAKRFTSKFIKNSYVLAWRMWFDIGIVREISKEHF 180

CHCC20348_2758 TPIMKMLLNNPASGFQKGIIVMEKGAAKRFTSKFIKNSYVLAWRMWFDIGIVREISKEHF 180

CHCC20333_1384 TPIMKMLLNNPASGFQKGIIVMEKGAAKRFTSKFIKNSYVLAWRMWFDIGIVREISKEHF 180

CHCC20490_4191 TPIMKMLLNNPASGFQKGIIVMEKGAAKRFTSKFIKNSYVLAWRMWFDIGIVREISKEHF 180

CHCC5019_3869 TPIMKMLLNNPASGFQKGIIVMEKGAAKRFTSKFIKNSYVLAWRMWFDIGIVREISKEHF 180

CHCC5022_3686 TPIMKMLLNNPASGFQKGIIVMEKGAAKRFTSKFIKNSYVLAWRMWFDIGIVREISKEHF 180

CHCC5023_4361 TPIMKMLLNNPASGFQKGIIVMEKGAAKRFTSKFIKNSYVLAWRMWFDIGIVREISKEHF 180

CHCC5027_4245 TPIMKMLLNNPASGFQKGIIVMEKGAAKRFTSKFIKNSYVLAWRMWFDIGIVREISKEHF 180

CHCC4186_0312 TPIMKMLLNNPASGFQKGIIVMEKGAAKRFTSKFIKNSYVLAWRMWFDIGIVREISKEHF 180

CHCC14820_3578 TPIMKMLLNNPASGFQKGIIVMEKGAAKRFTSKFIKNSYVLAWRMWFDIGIVREISKEHF 180

CHCC20375_2305 TPIMKMLLNNPASGFQKGIIVMEKGAAKRFTSKFMKNSYVLAWRMWFDIGIVREISKEHF 180

CHCC10893_4267 TPIMKMLLNNPASGFQKGIIVMEKGAAKRFTSKFIKNSYVLAWRMWFNIGIVREISKEHF 180

CHCC14564_0457 IPIMKMLLNNPASGFQKGIIVMEKGAAKRFTSKFIKNSYVLAWRMWFNIGIVREISKEHF 180

CHCC19466_0697 TPIMKMLLNNPASGFQKGIIVMEKGAAKRFTSKFIKNSYVLAWRMWFNIGIVREISKEHF 180

CHCC20495_3329 TPIMKMLLNNPASGFQKGIIVMEKGAAKRFTSKFIKNSYVLAWRMWFNIGIVREISKEHF 180

CHCC20323_0831 TPIMKMLLNNPASGFQKGIIVMEKGAAKRFTSKFIKNSYVLAWRMWFNIGIVREISKEHF 180

CHCC14525_1264 TPIMKMLLNNPASGFQKGIIVMEKGAAKRFTSKFIKNSYVLAWRMWFNIGIVREISKEHF 180

CHCC15320_0006 TPIMKMLLNNPASGFQKGIIVMEKGAAKRFTSKFIKNSYVLAWRMWFNIGIVREISKEHF 180

CHCC14600_3499 TPIMKMLLNNPASGFQKGIIVMEKGAAKRFTSKFIKNSYVLAWRMWFNIGIVREISKEHF 180

CHCC14813_1065 TPIMKMLLNNPASGFQKGIIVMEKGAAKRFTSKFIKNSYVLAWRMWFNIGIVREISKEHF 180

CHCC14598_4524 TPIMKMLLNNPASGFQKGIIVMEKGAAKRFTSKFIKNSYVLAWRMWFNIGIVREISKEHF 180

CHCC15291_3091 TPIMKMLLNNPASGFQKGIIVMEKGAAKRFTSKFIKNSYVLAWRMWFNIGIVREISKEHF 180

CHCC20373_3131 TPIMKMLLNNPASGFQKGIIVMEKGAAKRFTSKFIKNSYVLAWRMWFNIGIVREISKEHF 180

CHCC15289_0637 TPIMKMLLNNPASGFQKGIIVMEKGAAKRFTSKFIKNSYVLAWRMWFNIGIVREISKEHF 180

*********************************:************:************

CHCC20488_2428 SPPPKVDSAMVRITRKKDAPLSHKHYIAFRGLAEYALKEPNIPLCVALRGIFTPRQMKHL 240

BL09_3044 SPPPKVDSAMVRITRKKDAPLSHKHYIAFRGLAEYALKEPNIPLCVALRGIFTPRQMKHL 240

CHCC14817_2224 SPPPKVDSAMVRITRKKDAPLSHKHYIAFRGLAEYALKEPNIPLCVALRGIFTPRQMKHL 240

CHCC5021_0575 SPPPKVDSAMVRITRKKDAPLSHKHYIAFRGLAEYALKEPNIPLCVALRGIFTPRQMKHL 240

CHCC15332_2404 SPPPKVDSAMVRITRKKDAPLSHKHYIAFRGLAEYALKEPNIPLCVALRGIFTPRQMKHL 240

CHCC15337_3786 SPPPKVDSAMVRITRKKDAPLSHKHYIAFRGLAEYALKEPNIPLCVALRGIFTPRQMKHL 240

CHCC20347_3669 SPPPKVDSAMVRITRKKDAPLSHKHYIAFRGLAEYALKEPNIPLCVALRGIFTPRQMKHL 240

CHCC15136_3010 SPPPKVDSAMVRITRKKDAPLSHKHYIAFRGLAEYALKEPNIPLCVALRGIFTPRQMKHL 240

ATCC9945A_2991 SPPPKVDSAMVRITRKKDAPLSHKHYIAFRGLAEYALKEPNIPLCVALRGIFTPRQMKHL 240

CHCC14527_3619 SPPPKVDSAMVRITRKKDAPLSHKHYIAFRGLAEYALKEPNIPLCVALRGIFTPRQMKHL 240

CHCC14523_2532 SPPPKVDSAMVRITRKKDAPLSHKHYIAFRGLAEYALKEPNIPLCVALRGIFTPRQMKHL 240

CHCC20492_1445 SPPPKVDSAMVRITRKKDAPLSHKHYIAFRGLAEYALKEPNIPLCVALRGIFTPRQMKHL 240

CHCC20491_1195 SPPPKVDSAMVRITRKKDAPLSHKHYIAFRGLAEYALKEPNIPLCVALRGIFTPRQMKHL 240

CHCC20497_3638 SPPPKVDSAMVRITRKKDAPLSHKHYIAFRGLAEYALKEPNIPLCVALRGIFTPRQMKHL 240

CHCC19468_0855 SPPPKVDSAMVRITRKKDAPLSHKHYIAFRGLAEYALKEPNIPLCVALRGIFTPRQMKHL 240

CHCC19467_2168 SPPPKVDSAMVRITRKKDAPLSHKHYIAFRGLAEYALKEPNIPLCVALRGIFTPRQMKHL 240

CHCC12620_3847 SPPPKVDSAMVRITRKKDAPLSHKHYIAFRGLAEYALKEPNIPLCVALRGIFTPRQMKHL 240

CHCC15381_0096 SPPPKVDSAMVRITRKKDAPLSHKHYIAFRGLAEYALKEPNIPLCVALRGIFTPRQMKHL 240

CHCC14814_2880 SPPPKVDSAMVRITRKKDAPISHKHYIAFRGLAEYALKEPNIPLCVALRGIFTPPQMKHL 240

CHCC16874_3885 SPPPKVDSAMVRITRKKDAPLSHKHYIAFLGLAEYALKEPQAPFCVALRGIFTPRQMKHL 240

CHCC20494_3822 SPPPKVDSAMVRITRKKDAPLSHKHYIAFLGLAEYALKEPQAPFCVALRGIFTPRQMKHL 240

CHCC20496_2720 SPPPKVDSAMVRITRKKDAPLSHKHYIAFLGLAEYALKEPQAPFCVALRGIFTPRQMKHL 240

CHCC20493_4062 SPPPKVDSAMVRITRKKDAPLSHKHYIAFLGLAEYALKEPQAPFCVALRGIFTPRQMKHL 240

CHCC15325_3700 SPPPKVDSAMVRITRKKDAPLSHKHYIAFLGLAEYALKEPQAPFCVALRGIFTPRQMKHL 240

CHCC20343_0710 SPPPKVDSAMVRITRKKDAPLSHKHYIAFLGLAEYALKEPQAPFCVALRGIFTPRQMKHL 240

CHCC5025_4590 SPPPKVDSAMVRITRKKDAPLSHKHYIAFLGLAEYALKEPQAPFCVALRGIFTPRQMKHL 240

CHCC20331_0924 SPPPKVDSAMVRITRKKDAPLSHKHYIAFLGLAEYALKEPQAPLCVALRGIFTPRQMKHL 240

CHCC20372_3771 SPPPKVDSAMVRITRKKDAPLSHKHYIAFLGLAEYALKEPQAPLCVALRGIFTPRQMKHL 240

CHCC20348_2758 SPPPKVDSAMVRITRKKDAPLSHKHYIAFLGLAEYALKEPQAPLCVALRGIFTPRQMKHL 240

CHCC20333_1384 SPPPKVDSAMVRITRKKDAPLSHKHYIAFRGLAEYALKEPNIPLCVALRGIFTPRQMKHL 240

CHCC20490_4191 SPPPKVDSAMVRITRKKEAPLSHKHYIAFRGLAEYALKEPQAPFCVALRGIFTPRQMKHL 240

CHCC5019_3869 SPPPKVDSAMVRITRKKEAPLSHKHYIAFRGLAEYALKEPQAPFCVALRGIFTPRQMKHL 240

CHCC5022_3686 SPPPKVDSAMVRITRKKEAPLSHKHYIAFRGLAEYALKEPQAPFCVALRGIFTPRQMKHL 240

CHCC5023_4361 SPPPKVDSAMVRITRKKEAPLSHKHYIAFRGLAEYALKEPQAPFCVALRGIFTPRQMKHL 240

CHCC5027_4245 SPPPKVDSAMVRITRKKEAPLSHKHYIAFRGLAEYALKEPQAPFCVALRGIFTPRQMKHL 240

CHCC4186_0312 SPPPKVDSAMVRITRKKEAPLSHKHYIAFRGLAEYALKEPQAPFCVALRGIFTPRQMKHL 240

CHCC14820_3578 SPPPKVDSAMVRITRKKEAPLSHKHYIAFRGLAEYALKEPQAPFCVALRGIFTPRQMKHL 240

CHCC20375_2305 SPPPKVDSAMVRITRKKEAPISHKHYIAFLGLAEYALKEPQAPFCVALRGIFTPRQMKHL 240

CHCC10893_4267 SPPPKVDSAMVSITRKKEAPIPHKHYIAFLVLAEYALKEPHAPFCVALRGIFTPRQMKHL 240

CHCC14564_0457 SPPPKVDSAMVSITRKKEAPIPHKHYIAFLVLAEYALKEPHAPFCVALRGIFTPRQMKHL 240

CHCC19466_0697 SPPPKVDSAMVSITRKKEAPIPHKHYIAFLVLAEYALKEPHAPFCVALRGIFTPRQMKHL 240

CHCC20495_3329 SPPPKVDSAMVSITRKKEAPIPHKHYIAFLVLAEYALKEPHAPFCVALRGIFTPRQMKHL 240

CHCC20323_0831 SPPPKVDSAMVSITRKKEAPIPHKHYIAFLVLAEYALKEPHAPFCVALRGIFTPRQMKHL 240

CHCC14525_1264 SPPPKVDSAMVSITRKKEAPIPHKHYIAFLVLAEYALKEPHAPFCVALRGIFTPRQMKHL 240

CHCC15320_0006 SPPPKVDSAMVSITRKKEAPIPHKHYIAFLVLAEYALKEPHAPFCVALRGIFTPRQMKHL 240

CHCC14600_3499 SPPPKVDSAMVSITRKKEAPIPHKHYIAFLVLAEYALKEPHAPFCVALRGIFTPRQMKHL 240

CHCC14813_1065 SPPPKVDSAMVSITRKKEAPIPHKHYIAFLVLAEYALKEPHAPFCVALRGIFTPRQMKHL 240

CHCC14598_4524 SPPPKVDSAMVSITRKKEAPIPHKHYIAFLVLAEYALKEPHAPFCVALRGIFTPRQMKHL 240

CHCC15291_3091 SPPPKVDSAMVSITRKKEAPIPHKHYIAFLVLAEYALKEPHAPFCVALRGIFTPRQMKHL 240

CHCC20373_3131 SPPPKVDSAMVSITRKKEAPIPHKHYIAFLVLAEYALKEPHAPFCVALRGIFTPRQMKHL 240

CHCC15289_0637 SPPPKVDSAMVSITRKKEAPIPHKHYIAFLVLAEYALKEPHAPFCVALRGIFTPRQMKHL 240

*********** *****:**:.******* *********: *:**** ***** *****

CHCC20488_2428 RKSLKINNEKTVGTLTENQWAVIFNTMTQYVMHHKWPRANKRKPGEI 287

BL09_3044 RKSLKINNEKTVGTLTENQWAVIFNTMTQYVMHHKWPRANKRKPGEI 287

CHCC14817_2224 RKSLKINNEKTVGTLTENQWAVIFNTMTQYVMHHKWPRANKRKPGEI 287

CHCC5021_0575 RKSLKINNEKTVGTLTENQWAVIFNTMTQYVMHHKWPRANKRKPGEI 287

CHCC15332_2404 RKSLKINNEKTVGTLTENQWAVIFNTMTQYVMHHKWPRANKRKPGEI 287

CHCC15337_3786 RKSLKINNEKTVGTLTENQWAVIFNTMTQYVMHHKWPRANKRKPGEI 287

CHCC20347_3669 RKSLKINNEKTVGTLTENQWAVIFNTMTQYVMHHKWPRANKRKPGEI 287

CHCC15136_3010 RKSLKINNEKTVGTLTENQWAVIFNTMTQYVMHHKWPRANKRKPGEI 287

ATCC9945A_2991 RKSLKINNEKTVGTLTENQWAVIFNTMTQYVMHHKWPRANKRKPGEI 287

CHCC14527_3619 RKSLKINNEKTVGTLTENQWAVIFNTMTQYVMHHKWPRANKRKPGEI 287

CHCC14523_2532 RKSLKINNEKTVGTLTENQWAVIFNTMTQYVMHHKWPRANKRKPGEI 287

CHCC20492_1445 RKSLKINNEKTVGTLTENQWAVIFNTMTQYVMHHKWPRANKRKPGEI 287

CHCC20491_1195 RKSLKINNEKTVGTLTENQWAVIFNTMTQYVMHHKWPRANKRKPGEI 287

CHCC20497_3638 RKSLKINNEKTVGTLTENQWAVIFNTMTQYVMHHKWPRANKRKPGEI 287

CHCC19468_0855 RKSLKINNEKTVGTLTENQWAVIFNTMTQYVMHHKWPRANKRKPGEI 287

CHCC19467_2168 RKSLKINNEKTVGTLTENQWAVIFNTMTQYVMHHKWPRANKRKPGEI 287

CHCC12620_3847 RKSLKINNEKTVGTLTENQWAVIFNTMTQYVMHHKWPRANKRKPGEI 287

CHCC15381_0096 RKSLKINNEKTVGTLTENQWAVIFNTMTQYVMHHKWPRANKRKPGEI 287

CHCC14814_2880 RKSLKINNEKTVGTLTENQWAVIFNTMTQYVMHHKWPRANKRKPGEI 287

CHCC16874_3885 RKSLKINNEKTVGTLTENQWAVIFNTMTQYVMHHKWPRANKRKPGEI 287

CHCC20494_3822 RKSLKINNEKTVGTLTENQWAVIFNTMTQYVMHHKWPRANKRKPGEI 287

CHCC20496_2720 RKSLKINNEKTVGTLTENQWAVIFNTMTQYVMHHKWPRANKRKPGEI 287

CHCC20493_4062 RKSLKINNEKTVGTLTENQWAVIFNTMTQYVMHHKWPRANKRKPGEI 287

CHCC15325_3700 RKSLKINNEKTVGTLTENQWAVIFNTMTQYVMHHKWPRANKRKPGEI 287

CHCC20343_0710 RKSLKINNEKTVGTLTENQWAVIFNTMTQYVMHHKWPRANKRKPGEI 287

CHCC5025_4590 RKSLKINNEKTVGTLTENQWAVIFNTMTQYVMHHKWPRANKRKPGEI 287

CHCC20331_0924 RKSLKINNEKTVGTLTENQWAVIFNTMTQYVMHHKWPRANKRKSGEI 287

CHCC20372_3771 RKSLKINNEKTVGTLTENQWAVIFNTMTQYVMHHKWPRANKRKSGEI 287

CHCC20348_2758 RKSLKINNEKTVGTLTENQWAVIFNTMTQYVMHHKWPRANKRKSGEI 287

CHCC20333_1384 RKSLKINNEKTVGTLTENQWAVIFNTMTQYVMHHKWPRANKRKSGEI 287

CHCC20490_4191 RKSLKINNEKTVGTLTENQWAVIFYTMTQYVMHHKWPRANKRKPGEI 287

CHCC5019_3869 RKSLKINNEKTVGTLTENQWAVIFYTMTQYVMHHKWPRANKRKPGEI 287

CHCC5022_3686 RKSLKINNEKTVGTLTENQWAVIFYTMTQYVMHHKWPRANKRKPGEI 287

CHCC5023_4361 RKSLKINNEKTVGTLTENQWAVIFYTMTQYVMHHKWPRANKRKPGEI 287

CHCC5027_4245 RKSLKINNEKTVGTLTENQWAVIFYTMTQYVMHHKWPRANKRKPGEI 287

CHCC4186_0312 RKSLKINNEKTVGTLTENQWAVIFYTMTQYVMHHKWPRANKRKPGEI 287

CHCC14820_3578 RKSLKINNEKTVGTLTENQWAVIFNTMTQYVMHHKWPRANKRKPGEI 287

CHCC20375_2305 RKSLKINNEKTVGTLTENQWAVIFNTMTQYVMHHKWPRANKRKPGEI 287

CHCC10893_4267 RKSLKINNEKTVGTLTENQWAIIFKTMTQYVMHHKWPRANKRKPGE- 286

CHCC14564_0457 RKSLKINNEKTVGTLTENQWAIIFKTMTQYVMHHKWPRANKRKPGE- 286

CHCC19466_0697 RKSLKINNEKTVGTLTENQWAIIFKTMTQYVMHHKWPRANKRKPGE- 286

CHCC20495_3329 RKSLKINNEKTVGTLTENQWAIIFKTMTQYVMHHKWPRANKRKPGE- 286

CHCC20323_0831 RKSLKINNEKTVGTLTENQWAIIFKTMTQYVMHHKWPRANKRKPGE- 286

CHCC14525_1264 RKSLKINNEKTVGTLTENQWAIIFKTMTQYVMHHKWPRANKRKPGE- 286

CHCC15320_0006 RKSLKINNEKTVGTLTENQWAIIFKTMTQYVMHHKWPRANKRKPGE- 286

CHCC14600_3499 RKSLKINNEKTVGTLTENQWAIIFKTMTQYVMHHKWPRANKRKPGE- 286

CHCC14813_1065 RKSLKINNEKTVGTLTENQWAIIFKTMTQYVMHHKWPRANKRKPGE- 286

CHCC14598_4524 RKSLKINNEKTVGTLTENQWAIIFKTMTQYVMHHKWPRANKRKPGE- 286

CHCC15291_3091 RKSLKINNEKTVGTLTENQWAIIFKTMTQYVMHHKWPRANKRKPGE- 286

CHCC20373_3131 RKSLKINNEKTVGTLTENQWAIIFKTMTQYVMHHKWPRANKRKPGE- 286

CHCC15289_0637 RKSLKINNEKTVGTLTENQWAIIFKTMTQYVMHHKWPRANKRKPGE- 286

**.******************:** ******************.**
