# Supplementary material for: Putative antibiotic resistance genes present in extant Bacillus licheniformis and Bacillus paralicheniformis strains are probably intrinsic and part of the ancient resistome
Source: PLoS One. 2019 Jan 15;14(1):e0210363. doi: 10.1371/journal.pone.0210363 (PMC6333372; doi:10.1371/journal.pone.0210363)
Supplement: S11 Fig — Sequence alignments were made with Clustal 2.1 using default settings. Two examples of each ermD sequence variant are shown, including about 400 upstream nucleotides (upstream numbering is shown at the left). Identical nucleotides are indicated by an asterisk below each column. The start codon of the ermD gene is shown in green. The two putative encoded leader peptides LP1 and LP2 are shown above the nucleotide sequences. (DOCX) [file pone.0210363.s011.docx]

CHCC15289_ermD_variant_2 ---------------------------------------------CAGCCGTTGACGTTT

CHCC20495_ermD_variant_2 -------------------------------------GAAAATTCCAGCCGTTGACGTTT

CHCC20375_ermD_variant_outlier --------------------------------------------CCAGCCGTTGACGTTT

CHCC20348_ermD_variant_1.2.1 --------TGGCCTCCTGCGATATTCGTAAGGGACGAGAAAATTCCAGCCGTTGACGTTT

CHCC5025_ermD_variant_1.2.1 TTGCCTGATGGCCTCCTGCGATATTCGTAAGGGACGAGAAAATTCCAGCTGTTGACGTTT

CHCC5021_ermD_variant_1.1 ---------------------------TAAG-GAGAAGAAAATTCCAGCCGTTGACGTTT

CHCC20491_ermD_variant_1.1 --------------------------------------------------------GTTT

CHCC4186_ermD_variant_1.2.2 --------------------------------------AAAATTCCAGACGTTGACGTTT

CHCC5019_ermD_variant_1.2.2 ------------------------TCGTAAG-GAGAAGAAAATTCCAGACGTTGACGTTT

****

CHCC15289_ermD_variant_2 TCCAAAGAATGCCCTACAATGAGATCGTAACTCTAACTTTTCAGGAGGATTATTAAAAAA

CHCC20495_ermD_variant_2 TCCAAAGAATGCCCTACAATGAGATCGTAACTCTAACTTTTCAGGAGGATTATTAAAAAA

CHCC20375_ermD_variant_outlier TCCAAAGAATACCCTACAATGAGATCGTAACTTTAACTTTTCAGGAGGATTATTAAAAAA

CHCC20348_ermD_variant_1.2.1 TCCAAAGAATGCCCTACAATGAGATCGTAACTTTAACTTTTCAGGAGGATTATTAAAAAA

CHCC5025_ermD_variant_1.2.1 TCCAAAGAATGCCCTACAATGAGATCGTAACTTTAACTTTTCAGGAGGATTATTAAAAAA

CHCC5021_ermD_variant_1.1 TCCAAAGAATGCCCTACAATGAGATCGTAACTTTAACTTTTCAGGAGGATTATTAAAAAA

CHCC20491_ermD_variant_1.1 TCCAAAGAATGCCCTACAATGAGATCGTAACTTTAACTTTTCAGGAGGATTATTAAAAAA

CHCC4186_ermD_variant_1.2.2 TCCAAAGAATGCCCTACAATGAGATCGTAACTTTAACTTTTCAGGAGGATTATTAAAAAA

CHCC5019_ermD_variant_1.2.2 TCCAAAGAATGCCCTACAATGAGATCGTAACTTTAACTTTTCAGGAGGATTATTAAAAAA

**********.********************* ***************************

**LP1**

-322MetThrHisSerMetArgLeuArgPheProThrLeuAsnGln***

CHCC15289_ermD_variant_2 TGACACACTCAATGAGACTTCGTTTCCCAACTTTGAACCAGTAATTAAATACGTTCAAAG

CHCC20495_ermD_variant_2 TGACACACTCAATGAGACTTCGTTTCCCAACTTTGAACCAGTAATTAAATACGTTCAAAG

CHCC20375_ermD_variant_outlier TGACACACTCAATGAGACTGCGTTTCCCAACTTTGAACCAGTAATTAAATACGTTCAAAG

CHCC20348_ermD_variant_1.2.1 TGACACACTCAATGAGACTGCGTTTCCCAACTTTGAACCAGTAATTAAATACGTTCAAAG

CHCC5025_ermD_variant_1.2.1 TGACACACTCAATGAGACTGCGTTTCCCAACTTTGAACCAGTAATTAAATACGTTCAAAG

CHCC5021_ermD_variant_1.1 TGACACACTCAATGAGACTTCGTTTCCCAACTTTGAACCAGTAATTAAATACGTTCAAAG

CHCC20491_ermD_variant_1.1 TGACACACTCAATGAGACTTCGTTTCCCAACTTTGAACCAGTAATTAAATACGTTCAAAG

CHCC4186_ermD_variant_1.2.2 TGACACACTCAATGAGACTTCGTTTCCCAACTTTGAACCAGTAATTAAATACGTTCAAAG

CHCC5019_ermD_variant_1.2.2 TGACACACTCAATGAGACTTCGTTTCCCAACTTTGAACCAGTAATTAAATACGTTCAAAG

******************* ****************************************

**LP2**

-262 MetCysMetGlnSerLysArgAspGlnSerValLeuPhe***

CHCC15289_ermD_variant_2 GCTCTGTTTGTGTATGCAGAGTAAACGGGATCAGTCTGTCCTTTTTTAATAATCTTCATT

CHCC20495_ermD_variant_2 GCTCTGTTTGTGTATGCAGAGTAAACGGGATCAGTCTGTCCTTTTTTAATAATCTTCATT

CHCC20375_ermD_variant_outlier GCTCTGTTTGTGTATGCAGAGTAAACGGGATCAGTCTGTCCTTTTTTAATAATCTTCATT

CHCC20348_ermD_variant_1.2.1 GCTCTGTTTGTGTATGCAGAGTAAACGGGATCAGTCTGTCCTTTTTTAATAATCTTCAAT

CHCC5025_ermD_variant_1.2.1 GCTCTGTTTGTGTATGCAGAGTAAACGGGATCAGTCTGTCCTTTTTTAATAATCTTCAAT

CHCC5021_ermD_variant_1.1 GCTCTGTTTGTGTATGCAGAGTAAACGGGATCAGTCTGTCCTTTTTTAATAATCTTCAAT

CHCC20491_ermD_variant_1.1 GCTCTGTTTGTGTATGCAGAGTAAACGGGATCAGTCTGTCCTTTTTTAATAATCTTCAAT

CHCC4186_ermD_variant_1.2.2 GCTCTGTTTGTGTATGCAGAGTAAACGGGATCAGTCTGTCCTTTTTTAATAATCTTCAAT

CHCC5019_ermD_variant_1.2.2 GCTCTGTTTGTGTATGCAGAGTAAACGGGATCAGTCTGTCCTTTTTTAATAATCTTCAAT

**********************************************************:*

-202

CHCC15289_ermD_variant_2 TTGCATATAAAATTTGTAACTTGTAGAAAGGCGGATCGTTCTGCCTTTCTTTTTTGTTTA

CHCC20495_ermD_variant_2 TTGCATATAAAATTTGTAACTTGTAGAAAGGCGGATCGTTCTGCCTTTCTTTTTTGTTTA

CHCC20375_ermD_variant_outlier TTGCATATAAAATTTGTAACTTGGAGAAAGGCGGATCGTTCTGCCTTTCTTTTTTGTTTA

CHCC20348_ermD_variant_1.2.1 TTGCATATAAAATTTGTAACTTGTAGAAAGGCGGATCGTTCTGCCTTTCTTTTTTGTTTA

CHCC5025_ermD_variant_1.2.1 TTGCATATAAAATTTGTAACTTGTAGAAAGGCGGATCGTTCTGCCTTTCTTTTTTGTTTA

CHCC5021_ermD_variant_1.1 TTGCATATAAAATTTGTAATTTGTAGAAAGGCGGATCGTTCTGCCTTTCTTTTTTGTTTA

CHCC20491_ermD_variant_1.1 TTGCATATAAAATTTGTAATTTGTAGAAAGGCGGATCGTTCTGCCTTTCTTTTTTGTTTA

CHCC4186_ermD_variant_1.2.2 TTGCATATAAAATTTGTAATTTGTAGAAAGGCGGATCGTTCTGCCTTTCTTTTTTGTTTA

CHCC5019_ermD_variant_1.2.2 TTGCATATAAAATTTGTAATTTGTAGAAAGGCGGATCGTTCTGCCTTTCTTTTTTGTTTA

******************* *** ************************************

-142

CHCC15289_ermD_variant_2 ATCTCCTTTTTTTCCAACTGTTGAGAAAGGCTTATTTGGTTATGAAAACACCGCGGATTC

CHCC20495_ermD_variant_2 ATCTCCTTTTTTTCCAACTGTTGAGAAAGGCTTATTTGGTTATGAAAACACCGCGGATTC

CHCC20375_ermD_variant_outlier A--TCCTTTTTTTCTAACGGCTGAGAAAGGCTTATTTGGTTATGGAAATACCGCGGCTTC

CHCC20348_ermD_variant_1.2.1 A--TCCTT-TTTTCTAACGGCTGAGAAAGGCTTATTTGGTTATGGAAATACCGCGGATTC

CHCC5025_ermD_variant_1.2.1 A--TCCTTTTTTTCTAACGGCTGAGAAAGGCTTATTTGGTTATGGAAATACCGCGGATTC

CHCC5021_ermD_variant_1.1 A--TCCTTTTTTTCTAACGGCTGAGAAAGGCTTATTTGGTTATGGAAATACCGCGGCTTC

CHCC20491_ermD_variant_1.1 A--TCCTTTTTTTCTAACGGCTGAGAAAGGCTTATTTGGTTATGGAAATACCGCGGCTTC

CHCC4186_ermD_variant_1.2.2 A--TCCTTTTTTTCTAACGGCTGAGAAAGGCTTATTTGGTTATGGAAATACCGCGGCTTC

CHCC5019_ermD_variant_1.2.2 A--TCCTTTTTTTCTAACGGCTGAGAAAGGCTTATTTGGTTATGGAAATACCGCGGCTTC

* ***** ***** *** * ***********************.*** *******.***

-82

CHCC15289_ermD_variant_2 ATCCTTAAAGGATGGCTCTTCCCTTTACTCTGAATCACAGGCAGACCGCCTGTGATTTTT

CHCC20495_ermD_variant_2 ATCCTTAAAGGATGGCTCTTCCCTTTACTCTGAATCACAGGCAGACCGCCTGTGATTTTT

CHCC20375_ermD_variant_outlier ATCCTTAAAGGATGGCTCTTCCCTTTACTCTGAATCACAGGCAGACCGCCTGTGATTTTT

CHCC20348_ermD_variant_1.2.1 ATCCTTAAAGGATGGCTCTTCCCTTTACTCTGAATCACAGGCAGACCGCCTGTGATTTTT

CHCC5025_ermD_variant_1.2.1 ATCCTTAAAGGATGGCTCTTCCCTTTACTCTGAATCACAGGCAGACCGCCTGTGATTTTT

CHCC5021_ermD_variant_1.1 ATCCTTAAAGGATGGCTCTTCCCTTTACTCTGAATCACAGGCAGACCGCCTGTGATTTTT

CHCC20491_ermD_variant_1.1 ATCCTTAAAGGATGGCTCTTCCCTTTACTCTGAATCACAGGCAGACCGCCTGTGATTTTT

CHCC4186_ermD_variant_1.2.2 ATCCTTAAAGGATGGCTCTTCCCTTTACTCTGAATCACAGGCAGACCGCCTGTGATTTTT

CHCC5019_ermD_variant_1.2.2 ATCCTTAAAGGATGGCTCTTCCCTTTACTCTGAATCACAGGCAGACCGCCTGTGATTTTT

************************************************************

-22 *ermD* methylase

CHCC15289_ermD_variant_2 TATGATGAGAGGAAGAGGAAACGTGAAGAAAAAAAATCTTAAGTACAGAGGAAAAAAGTT

CHCC20495_ermD_variant_2 TATGATGAGAGGAAGAGGAAACGTGAAGAAAAAAAATCTTAAGTACAGAGGAAAAAAGTT

CHCC20375_ermD_variant_outlier TATGATGAGAGGAAGAGGAAACGTGAAGAAAAAAAATCATAAGTACAGAGGAAAAAAGCT

CHCC20348_ermD_variant_1.2.1 TATGATGAGAGGAAGAGGAAACATGAAGAAAAAAAATCATAAGTACAGAGGAAAAAAGTT

CHCC5025_ermD_variant_1.2.1 TATGATGAGAGGAAGAGGAAACATGAAGAAAAAAAATCATAAGTACAGAGGAAAAAAGTT

CHCC5021_ermD_variant_1.1 TATGATGAGAGGAAGAGGAAACATGAAGAAAAAAAATCATAAGTACAGAGGAAAAAAGTT

CHCC20491_ermD_variant_1.1 TATGATGAGAGGAAGAGGAAACATGAAGAAAAAAAATCATAAGTACAGAGGAAAAAAGTT

CHCC4186_ermD_variant_1.2.2 TATGATGAGAGGAAGAGGAAACATGAAGAAAAAAAATCATAAGTACAGAGGAAAAAAGTT

CHCC5019_ermD_variant_1.2.2 TATGATGAGAGGAAGAGGAAACATGAAGAAAAAAAATCATAAGTACAGAGGAAAAAAGTT

**********************.***************:******************* *

CHCC15289_ermD_variant_2 AAACCGCGGGGAATCTCCGAATTTTTCCGGACAGCATTTGATGCATAATAAAAAATTAAT

CHCC20495_ermD_variant_2 AAACCGCGGGGAATCTCCGAATTTTTCCGGACAGCATTTGATGCATAATAAAAAATTAAT

CHCC20375_ermD_variant_outlier AAATCGCAGGGAATCTCCGAATTTTTCCGGACAGCATTTGATGCATAATAAAAAATTAAT

CHCC20348_ermD_variant_1.2.1 AAACCGCGGGGAATATCCGAATTTTTCCGGACAGCATTTGATGCATAATAAAAAATTAAT

CHCC5025_ermD_variant_1.2.1 AAACCGCGGGGAATATCCGAATTTTTCCGGACAGCATTTGATGCATAATAAAAAATTAAT

CHCC5021_ermD_variant_1.1 AAACCGCGGGGAATCTCCGAATTTTTCCGGACAGCATTTGATGCATAATAAAAAATTAAT

CHCC20491_ermD_variant_1.1 AAACCGCGGGGAATCTCCGAATTTTTCCGGACAGCATTTGATGCATAATAAAAAATTAAT

CHCC4186_ermD_variant_1.2.2 AAACCGCGGGGAATATCCGAATTTTTCCGGACAGCATTTGATGCATAATAAAAAATTAAT

CHCC5019_ermD_variant_1.2.2 AAACCGCGGGGAATATCCGAATTTTTCCGGACAGCATTTGATGCATAATAAAAAATTAAT

*** ***.******.*********************************************

CHCC15289_ermD_variant_2 TGAAGAAATTGTAGATCGGGCGAATATTGGCATTAACGATACAGTTTTAGAGTTAGGGGC

CHCC20495_ermD_variant_2 TGAAGAAATTGTAGATCGGGCGAATATTGGCATTAACGATACAGTTTTAGAGTTAGGGGC

CHCC20375_ermD_variant_outlier TGAAGAAATTGTGGATTGGGCAAATATTGGCATAGACGATACAGTTTTAGAGTTAGGGGC

CHCC20348_ermD_variant_1.2.1 TGAAGAAATTGTGGATCGAGCAAATATTAGCATAGACGATACGGTTTTAGAGTTAGGAGC

CHCC5025_ermD_variant_1.2.1 TGAAGAAATTGTGGATCGGGCAAATATTAGCATAGACGATACGGTTTTAGAGTTAGGAGC

CHCC5021_ermD_variant_1.1 TGAAGAAATTGTGGATCGGGCAAATATTAGCATAGACGATACGGTTTTAGAGTTAGGAGC

CHCC20491_ermD_variant_1.1 TGAAGAAATTGTGGATCGGGCAAATATTAGCATAGACGATACGGTTTTAGAGTTAGGAGC

CHCC4186_ermD_variant_1.2.2 TGAAGAAATTGTGGATCGGGCAAATATTAGCATAGACGATACGGTTTTAGAGTTAGGAGC

CHCC5019_ermD_variant_1.2.2 TGAAGAAATTGTGGATCGGGCAAATATTAGCATAGACGATACGGTTTTAGAGTTAGGAGC

************.*** *.**.******.****:.*******.**************.**

CHCC15289_ermD_variant_2 TGGAAAAGGTGCTTTGACAACTGTGCTAAGTCAAAAAGCCGGTAAGGTATTGGCAGTGGA

CHCC20495_ermD_variant_2 TGGAAAAGGTGCTTTGACAACTGTGCTAAGTCAAAAAGCCGGTAAGGTATTGGCAGTGGA

CHCC20375_ermD_variant_outlier TGGAAAAGGTGCTTTGACAACTGTGCTAAGTCAAAAAGCCGGTAAGGTATTGGCAGTGGA

CHCC20348_ermD_variant_1.2.1 GGGAAAAGGTGCTTTGACAACTATGCTAAGTCAAAAAGCCGGTAAGGTATTGGCAGTGGA

CHCC5025_ermD_variant_1.2.1 GGGAAAAGGTGCTTTGACAACTATGCTAAGTCAAAAAGCCGGTAAGGTATTGGCAGTGGA

CHCC5021_ermD_variant_1.1 GGGAAAAGGGGCTTTGACAACTGTGCTAAGTCAAAAAGCCGGTAAGGTATTGGCAGTGGA

CHCC20491_ermD_variant_1.1 GGGAAAAGGGGCTTTGACAACTGTGCTAAGTCAAAAAGCCGGTAAGGTATTGGCAGTGGA

CHCC4186_ermD_variant_1.2.2 GGGAAAAGGGGCTTTGACAACTGTGCTAAGTCAAAAAGCCGGTAAGGTATTGGCAGTGGA

CHCC5019_ermD_variant_1.2.2 GGGAAAAGGGGCTTTGACAACTGTGCTAAGTCAAAAAGCCGGTAAGGTATTGGCAGTGGA

******** ************.*************************************

CHCC15289_ermD_variant_2 AAACGATTCTAAATTCGTTGGTATACTCACACGTAAAACAGCACAGCACTCAAATGCGAA

CHCC20495_ermD_variant_2 AAACGATTCTAAATTCGTTGGTATACTCACACGTAAAACAGCACAGCACTCAAATGCGAA

CHCC20375_ermD_variant_outlier AAACGATTCTAAATTCGTTGATATACTCACACGTAAAACAGCACATCACTCAAATACGAA

CHCC20348_ermD_variant_1.2.1 AAACGATTCTAAATTCGTTGCTATACTCACACGTAAAACAGCACAGCATCCAAATACGAA

CHCC5025_ermD_variant_1.2.1 AAACGATTCTAAATTCGTTGCTATACTCACACGTAAAACAGCACAGCATCCAAATACGAA

CHCC5021_ermD_variant_1.1 AAACGATTCTAAATTCGTTGATATACTCACACGTAAAACGGCACAGCATTCAAATACGAA

CHCC20491_ermD_variant_1.1 AAACGATTCTAAATTCGTTGATATACTCACACGTAAAACGGCACAGCATTCAAATACGAA

CHCC4186_ermD_variant_1.2.2 AAACGATTCTAAATTCGTTGATATACTCACACGTAAAACAGCACAGCATTCAAATACGAA

CHCC5019_ermD_variant_1.2.2 AAACGATTCTAAATTCGTTGATATACTCACACGTAAAACGGCACAGCATTCAAATACGAA

******************** ******************.***** ** *****.****

CHCC15289_ermD_variant_2 AATTATTCATCAAGATATCATGAAGATTCATTTACCAAAAGAAAAGTTTGTGGTGGTCTC

CHCC20495_ermD_variant_2 AATTATTCATCAAGATATCATGAAGATTCATTTACCAAAAGAAAAGTTTGTGGTGGTCTC

CHCC20375_ermD_variant_outlier AATTATTCATCAAGATATCATGAAGATTCATTTACCAAAAGAAAAGTTTGTGGTGGTCTC

CHCC20348_ermD_variant_1.2.1 AATTATTCATCAAGATATCATGAAGATTCATTTACCAAAAGAAAAGTTTGTGGTGGTCTC

CHCC5025_ermD_variant_1.2.1 AATTATTCATCAAGATATCATGAAGATTCATTTACCAAAAGAAAAGTTTGTGGTGGTCTC

CHCC5021_ermD_variant_1.1 AATTATTCATCAAGATATCATGAAGATTCATTTACCAAAAGAAAAGTTTGTGGTGGTCTC

CHCC20491_ermD_variant_1.1 AATTATTCATCAAGATATCATGAAGATTCATTTACCAAAAGAAAAGTTTGTGGTGGTCTC

CHCC4186_ermD_variant_1.2.2 AATTATTCATCAAGATATCATGAAGATTCATTTACCAAAAGAAAAGTTTGTGGTGGTCTC

CHCC5019_ermD_variant_1.2.2 AATTATTCATCAAGATATCATGAAGATTCATTTACCAAAAGAAAAGTTTGTGGTGGTCTC

************************************************************

CHCC15289_ermD_variant_2 TAATATTCCTTATGCCATCACAACCCCCATTATGAAAATGCTTTTGAACAATCCTGCAAG

CHCC20495_ermD_variant_2 TAATATTCCTTATGCCATCACAACCCCCATTATGAAAATGCTTTTGAACAATCCTGCAAG

CHCC20375_ermD_variant_outlier TAATATTCCCTATGCCATCACAACCCCCATCATGAAAATGCTTTTGAACAATCCTGCAAG

CHCC20348_ermD_variant_1.2.1 TAATATTCCCTATGCCATCACAACTCCCATCATGAAAATGCTCTTGAACAATCCTGCAAG

CHCC5025_ermD_variant_1.2.1 TAATATTCCCTATGCCATCACAACTCCCATCATGAAAATGCTCTTGAACAATCCTGCAAG

CHCC5021_ermD_variant_1.1 TAATATTCCCTATGCCATCACAACCCCCATCATGAAAATGCTTTTGAACAATCCTGCAAG

CHCC20491_ermD_variant_1.1 TAATATTCCCTATGCCATCACAACCCCCATCATGAAAATGCTTTTGAACAATCCTGCAAG

CHCC4186_ermD_variant_1.2.2 TAATATTCCCTATGCCATCACAACCCCCATCATGAAAATGCTTTTGAACAATCCTGCAAG

CHCC5019_ermD_variant_1.2.2 TAATATTCCCTATGCCATCACAACCCCCATCATGAAAATGCTTTTGAACAATCCTGCAAG

********* ************** ***** *********** *****************

CHCC15289_ermD_variant_2 CGGATTTCAAAAAGGCATCATCGTAATGGAAAAAGGGGCTGCTAAACGATTCACATCAAA

CHCC20495_ermD_variant_2 CGGATTTCAAAAAGGCATCATCGTAATGGAAAAAGGGGCTGCTAAACGATTCACATCAAA

CHCC20375_ermD_variant_outlier CGGATTTCAAAAAGGCATCATCGTAATGGAAAAAGGGGCTGCTAAACGATTCACATCAAA

CHCC20348_ermD_variant_1.2.1 CGGATTTCAAAAAGGGATCATCGTAATGGAAAAAGGGGCTGCTAAACGTTTCACATCAAA

CHCC5025_ermD_variant_1.2.1 CGGATTTCAAAAAGGGATCATCGTAATGGAAAAAGGGGCTGCTAAACGTTTCACATCAAA

CHCC5021_ermD_variant_1.1 CGGATTTCAAAAAGGGATCATCGTAATGGAAAAAGGGGCTGCTAAACGTTTCACATCAAA

CHCC20491_ermD_variant_1.1 CGGATTTCAAAAAGGGATCATCGTAATGGAAAAAGGGGCTGCTAAACGTTTCACATCAAA

CHCC4186_ermD_variant_1.2.2 CGGATTTCAAAAAGGGATCATCGTAATGGAAAAAGGGGCTGCTAAACGTTTCACATCAAA

CHCC5019_ermD_variant_1.2.2 CGGATTTCAAAAAGGGATCATCGTAATGGAAAAAGGGGCTGCTAAACGTTTCACATCAAA

*************** ********************************:***********

CHCC15289_ermD_variant_2 ATTCATTAAAAACTCCTATGTTTTAGCTTGGAGAATGTGGTTTAATATTGGCATTGTCAG

CHCC20495_ermD_variant_2 ATTCATTAAAAACTCCTATGTTTTAGCTTGGAGAATGTGGTTTAATATTGGCATTGTCAG

CHCC20375_ermD_variant_outlier ATTCATGAAAAATTCCTATGTTTTAGCTTGGAGAATGTGGTTTGATATTGGCATTGTCAG

CHCC20348_ermD_variant_1.2.1 ATTCATTAAAAATTCCTATGTTTTAGCTTGGAGAATGTGGTTTGATATTGGCATTGTCAG

CHCC5025_ermD_variant_1.2.1 ATTCATTAAAAATTCCTATGTTTTAGCTTGGAGAATGTGGTTTGATATTGGCATTGTCAG

CHCC5021_ermD_variant_1.1 ATTCATTAAAAATTCCTATGTTTTAGCTTGGAGAATGTGGTTTGATATTGGCATTGTCAG

CHCC20491_ermD_variant_1.1 ATTCATTAAAAATTCCTATGTTTTAGCTTGGAGAATGTGGTTTGATATTGGCATTGTCAG

CHCC4186_ermD_variant_1.2.2 ATTCATTAAAAATTCCTATGTTTTAGCTTGGAGAATGTGGTTTGATATTGGCATTGTCAG

CHCC5019_ermD_variant_1.2.2 ATTCATTAAAAATTCCTATGTTTTAGCTTGGAGAATGTGGTTTGATATTGGCATTGTCAG

****** ***** ******************************.****************

CHCC15289_ermD_variant_2 AGAAATATCGAAAGAACATTTTTCTCCCCCTCCAAAAGTGGACTCGGCAATGGTCAGCAT

CHCC20495_ermD_variant_2 AGAAATATCGAAAGAACATTTTTCTCCCCCTCCAAAAGTGGACTCGGCAATGGTCAGCAT

CHCC20375_ermD_variant_outlier AGAAATATCGAAAGAACATTTTTCTCCCCCTCCAAAAGTGGACTCGGCAATGGTTAGAAT

CHCC20348_ermD_variant_1.2.1 AGAAATATCGAAAGAGCATTTTTCTCCCCCTCCAAAAGTGGACTCGGCAATGGTCAGAAT

CHCC5025_ermD_variant_1.2.1 AGAAATATCGAAAGAGCATTTTTCTCCCCCTCCAAAAGTGGACTCGGCAATGGTCAGAAT

CHCC5021_ermD_variant_1.1 AGAAATATCGAAAGAGCATTTTTCTCCCCCTCCAAAAGTGGACTCGGCAATGGTCAGAAT

CHCC20491_ermD_variant_1.1 AGAAATATCGAAAGAGCATTTTTCTCCCCCTCCAAAAGTGGACTCGGCAATGGTCAGAAT

CHCC4186_ermD_variant_1.2.2 AGAAATATCGAAAGAGCATTTTTCTCCCCCTCCAAAAGTGGACTCGGCAATGGTCAGAAT

CHCC5019_ermD_variant_1.2.2 AGAAATATCGAAAGAGCATTTTTCTCCCCCTCCAAAAGTGGACTCGGCAATGGTCAGAAT

***************.************************************** **.**

CHCC15289_ermD_variant_2 AACACGAAAAAAAGAAGCGCCTATACCACATAAACATTACATTGCATTTCTTGTGCTTGC

CHCC20495_ermD_variant_2 AACACGAAAAAAAGAAGCGCCTATACCACATAAACATTACATTGCATTTCTTGTGCTTGC

CHCC20375_ermD_variant_outlier AACACGGAAAAAAGAAGCGCCTATATCACATAAACATTACATTGCATTTCTTGGGCTTGC

CHCC20348_ermD_variant_1.2.1 AACACGAAAAAAAGACGCGCCTCTATCACATAAACATTACATTGCGTTTCTTGGGCTTGC

CHCC5025_ermD_variant_1.2.1 AACACGAAAAAAAGACGCGCCTCTATCACATAAACATTACATTGCGTTTCTTGGGCTTGC

CHCC5021_ermD_variant_1.1 AACACGAAAAAAAGACGCGCCTCTATCACATAAACATTATATTGCGTTTCGGGGACTTGC

CHCC20491_ermD_variant_1.1 AACACGAAAAAAAGACGCGCCTCTATCACATAAACATTATATTGCGTTTCGGGGACTTGC

CHCC4186_ermD_variant_1.2.2 AACACGAAAAAAAGAAGCGCCTCTATCACATAAACATTACATTGCGTTTCGGGGGCTTGC

CHCC5019_ermD_variant_1.2.2 AACACGAAAAAAAGAAGCGCCTCTATCACATAAACATTACATTGCGTTTCGGGGGCTTGC

******.********.******.** ************* *****.**** * .*****

CHCC15289_ermD_variant_2 CGAATATGCGCTAAAGGAGCCGCATGCCCCTTTCTGTGTTGCTTTACGCGGGATTTTTAC

CHCC20495_ermD_variant_2 CGAATATGCGCTAAAGGAGCCGCATGCCCCTTTCTGTGTTGCTTTACGCGGGATTTTTAC

CHCC20375_ermD_variant_outlier TGAATATGCGCTCAAGGAGCCGCAAGCCCCTTTCTGTGTTGCTTTACGCGGAATTTTTAC

CHCC20348_ermD_variant_1.2.1 CGAATATGCGCTAAAGGAGCCGCAAGCCCCTCTCTGTGTTGCTTTACGCGGAATTTTTAC

CHCC5025_ermD_variant_1.2.1 CGAATATGCGCTAAAGGAGCCGCAAGCCCCTTTCTGTGTTGCTTTACGCGGAATTTTTAC

CHCC5021_ermD_variant_1.1 CGAATACGCGCTAAAGGAGCCGAATATCCCTCTCTGTGTTGCTTTACGCGGAATTTTTAC

CHCC20491_ermD_variant_1.1 CGAATACGCGCTAAAGGAGCCGAATATCCCTCTCTGTGTTGCTTTACGCGGAATTTTTAC

CHCC4186_ermD_variant_1.2.2 CGAATATGCGCTAAAGGAGCCGCAAGCCCCTTTCTGTGTTGCTTTACGCGGAATTTTTAC

CHCC5019_ermD_variant_1.2.2 CGAATATGCGCTAAAGGAGCCGCAAGCCCCTTTCTGTGTTGCTTTACGCGGAATTTTTAC

***** *****.*********.*:. **** *******************.********

CHCC15289_ermD_variant_2 TCCGCGTCAAATGAAACACTTAAGAAAAAGTCTAAAAATCAACAACGAAAAAACCGTTGG

CHCC20495_ermD_variant_2 TCCGCGTCAAATGAAACACTTAAGAAAAAGTCTAAAAATCAACAACGAAAAAACCGTTGG

CHCC20375_ermD_variant_outlier TCCGCGTCAAATGAAGCACTTAAGAAAAAGTCTAAAAATCAACAATGAAAAAACCGTTGG

CHCC20348_ermD_variant_1.2.1 CCCGCGTCAAATGAAACACTTAAGAAAAAGTCTAAAAATCAACAATGAAAAAACCGTTGG

CHCC5025_ermD_variant_1.2.1 TCCGCGTCAAATGAAACACTTAAGAAAAAGTCTAAAAATTAACAATGAAAAAACCGTTGG

CHCC5021_ermD_variant_1.1 CCCGCGTCAAATGAAACACTTAAGAAAAAGTCTAAAAATCAACAATGAAAAAACCGTTGG

CHCC20491_ermD_variant_1.1 CCCGCGTCAAATGAAACACTTAAGAAAAAGTCTAAAAATCAACAATGAAAAAACCGTTGG

CHCC4186_ermD_variant_1.2.2 CCCGCGTCAAATGAAACACTTAAGAAAAAGTCTAAAAATCAACAATGAAAAAACCGTTGG

CHCC5019_ermD_variant_1.2.2 CCCGCGTCAAATGAAACACTTAAGAAAAAGTCTAAAAATCAACAATGAAAAAACCGTTGG

**************.*********************** ***** **************

CHCC15289_ermD_variant_2 AACGCTCACCGAAAACCAATGGGCGATTATTTTTAAGACGATGACTCAATATGTGATGCA

CHCC20495_ermD_variant_2 AACGCTCACCGAAAACCAATGGGCGATTATTTTTAAGACGATGACTCAATATGTGATGCA

CHCC20375_ermD_variant_outlier AACGCTCACCGAAAACCAATGGGCGGTTATTTTTAACACGATGACTCAATATGTGATGCA

CHCC20348_ermD_variant_1.2.1 AACGCTCACCGAAAACCAATGGGCGGTTATTTTTAACACGATGACTCAATATGTGATGCA

CHCC5025_ermD_variant_1.2.1 AACGCTCACCGAAAACCAATGGGCGGTTATTTTTAACACGATGACTCAATATGTGATGCA

CHCC5021_ermD_variant_1.1 AACGCTCACCGAAAACCAATGGGCGGTTATTTTTAACACGATGACTCAGTATGTAATGCA

CHCC20491_ermD_variant_1.1 AACGCTCACCGAAAACCAATGGGCGGTTATTTTTAACACGATGACTCAGTATGTAATGCA

CHCC4186_ermD_variant_1.2.2 AACGCTCACCGAAAACCAATGGGCGGTTATTTTTTACACGATGACTCAGTATGTAATGCA

CHCC5019_ermD_variant_1.2.2 AACGCTCACCGAAAACCAATGGGCGGTTATTTTTTACACGATGACTCAGTATGTAATGCA

*************************.********:* ***********.*****.*****

CHCC15289_ermD_variant_2 CCACAAATGGCCAAGAGCAAATAAGAGAAAACCCGGAGAA---TAAAGAA-AAAAGCTGC

CHCC20495_ermD_variant_2 CCACAAATGGCCAAGAGCAAATAAGAGAAAACCCGGAGAA---TAAAGAA-AAAAGCTGC

CHCC20375_ermD_variant_outlier CCACAAATGGCCAAGAGCAAATAAGAGAAAACCCGGAGAAATATAAAGAA-AAAAGCTGC

CHCC20348_ermD_variant_1.2.1 CCACAAATGGCCAAGAGCAAATAAGCGAAAATCCGGAGAAATATAAAGAAAAAAAGCTGC

CHCC5025_ermD_variant_1.2.1 CCACAAATGGCCAAGAGCAAATAAGCGAAAACCCGGAGAAATATAAAGAAAAAAAGCTGC

CHCC5021_ermD_variant_1.1 TCACAAATGGCCAAGAGCAAATAAGCGAAAACCCGGAGAAATATAAAGAA-AAAAGCTGC

CHCC20491_ermD_variant_1.1 TCACAAATGGCCAAGAGCAAATAAGCGAAAACCCGGAGAAATATAAAGAA-AAAAGCTGC

CHCC4186_ermD_variant_1.2.2 TCACAAATGGCCAAGAGCAAATAAGCGAAAACCCGGAGAAATATAAAGAA-AAAAGCTGC

CHCC5019_ermD_variant_1.2.2 TCACAAATGGCCAAGAGCAAATAAGCGAAAACCCGGAGAAATATAAAGAA-AAAAGCTGC

************************.***** ********::******** *********

CHCC15289_ermD_variant_2 TGGCATCTCGTCAGCAGCTTAAGCT-TTTCCGG-GGGATTCAGATGTCCCTGTCAACATT

CHCC20495_ermD_variant_2 TGGCATCTCGTCAGCAGCTTAAGCT-TTTCCGG-GGGATTCAGATGTCCCTGTCAACATT

CHCC20375_ermD_variant_outlier TGACGTCTCGTCAGCAGCTTAAGCT-TTTCTGGAGGGATTCAGATGTCCCTGTCAACATT

CHCC20348_ermD_variant_1.2.1 TGACGTCTCGTCAGCAGCTTAAGCT-TTTCCGGAGGGATTCAGATGTCCCTGTCAACATT

CHCC5025_ermD_variant_1.2.1 TGACGTCTCGTCAGCAGCTTAAGCT-TTTCTGGAGGGATTCAGATGTCCCTGTCAACATT

CHCC5021_ermD_variant_1.1 TGACTTCCCGTCAGCAGCTTTAGCTATTTCTGGAGGGATTCAGATGTCCCTGTCAACATT

CHCC20491_ermD_variant_1.1 TGACTTCCCGTCAGCAGCTTTAGCTATTTCTGGAGGGATTCAGATGTCCCTGTCAACATT

CHCC4186_ermD_variant_1.2.2 TGACTTCCCGTCAGCAGCTTTAGCTATTTCTGGAGGGATTCAGATGTCCCTGTCAACATT

CHCC5019_ermD_variant_1.2.2 TGACTTCCCGTCAGCAGCTTTAGCTATTTCTGGAGGGATTCAGATGTCCCTGTCAACATT

**.* ** ************:**** **** **.**************************

CHCC15289_ermD_variant_2 CCTTTTGCCCGCTTACAGCTTTCCAGTTAAAAACTGCGCCTCGCCGAGGCC---------

CHCC20495_ermD_variant_2 CCTTTTGCCCGCTTACAGCTTTCCAGTTAAAAACTGCGCCTCG-----------------

CHCC20375_ermD_variant_outlier CCTTTTGCCCGCTTACAGCTTTCCAGTTAAAAACTGCGCCTCGCCGAG------------

CHCC20348_ermD_variant_1.2.1 CCTTTTGCCCGCTTACAGCTTTCCAGTTAAAAACTGCGCCTCGCCGAGGCCGAAACTCCA

CHCC5025_ermD_variant_1.2.1 CCTTTTGCCCGCTTACAGCTTTCCAGTTAAAAACTGCGCCTCGCCGAGGCCGAAACTCCA

CHCC5021_ermD_variant_1.1 CCTTTTGTCCGCTTACAGCTTTCCAGTTAAAAACTGCGCCTCGCCGAGGCCGAAACTCCA

CHCC20491_ermD_variant_1.1 CCTTTTGTCCGCTTACAGCTTTCCAGTTAAAAACTGCGCCTCGCCGAGGCCGAAACTCC-

CHCC4186_ermD_variant_1.2.2 CCTTTTGTCCGCTTACAGCTTTCCAGTTAAAAACTGCGCCT-------------------

CHCC5019_ermD_variant_1.2.2 CCTTTTGTCCGCTTACAGCTTTCCAGTT--------------------------------

******* ********************
